# Samotolisib Attenuates Acute Liver Injury Through Inhibiting Caspase-11-Mediated Pyroptosis Via Regulating E3 Ubiquitin Ligase Nedd4

**DOI:** 10.3389/fphar.2021.726198

**Published:** 2021-08-13

**Authors:** Yang-Yang Zhao, Dong-Ming Wu, Miao He, Feng Zhang, Ting Zhang, Teng Liu, Jin Li, Li Li, Ying Xu

**Affiliations:** ^1^ Chengdu Medical College, Chengdu, China; ^2^ The First Affiliated Hospital of Chengdu Medical College, Chengdu, China

**Keywords:** samotolisib, acute liver injury, lipopolysaccharide, pyroptosis, caspase-11

## Abstract

Acute liver injury (ALI) is associated with poor survival in patients with sepsis. During sepsis, the liver is the main site of bacterial endotoxin-induced inflammation. Lipopolysaccharide (LPS) promotes caspase-4/5/11 activation, leading to pyroptosis, a major sepsis driver. This study aimed to identify novel drugs that can control hepatocyte caspase-4/5/11 activation during sepsis. We performed LPS-induced caspase-11 activation and pyroptosis in RAW 264.7 cells and established an LPS-induced ALI mouse model. We identified samotolisib (ST), a novel dual phosphoinositide 3-kinase (PI3K) and mammalian target of rapamycin (mTOR) inhibitor, by screening a library of 441 pyroptosis compounds with known targets, which dose-dependently inhibited caspase-11 activation and N-terminal fragment of gasdermin D (GSDMD-NT) generation, reducing RAW 264.7 cell pyroptosis. In mice, ST preconditioning improved survival, attenuated LPS-induced serum alanine aminotransferase and aspartate aminotransferase activity, and inhibited severe liver inflammation and damage. Importantly, ST treatment activated Nedd4, which directly interacts with and mediates caspase-11 ubiquitination and degradation. This was largely abrogated by insulin-like growth factor 1. ST ameliorated LPS-induced hepatotoxicity by inhibiting caspase-11/GSDMD-NT pyroptosis signaling via regulating PI3K/AKT/mTOR/Nedd4 signaling. Hence, ST may play a key role in the prevention of liver injury in patients with sepsis.

## Introduction

Acute liver injury (ALI) occurs at any stage of sepsis and is an independent risk factor for sepsis-induced death (Sun et al., 2020). Sepsis is characterized by life-threatening organ dysfunction caused by a dysregulated host response to infection (Singer et al., 2016). It is a common cause of death in intensive care units (Bauer et al., 2020). Inflammation is the main feature of sepsis, and the liver, as a lymphatic organ, plays an important role in inflammation. Clinical and experimental data suggest that liver dysfunction is an early sign of sepsis, and early hepatic dysfunction in patients with sepsis is a specific and independent risk factor for poor outcomes (Kramer et al., 2007). Attenuating liver injury and restoring the balance of pro- and anti-inflammatory responses in the liver will lower sepsis morbidity and mortality rates by regulating systemic immune responses and protecting organs from injury (Yan, Li, and Li 2014). However, there is no specific therapy for sepsis-induced liver injury.

A study showed that Gram-negative bacterial infections were more common than Gram-positive bacterial infections among patients with sepsis in the United States of America (Cecconi et al., 2018). Pathogen-associated molecular patterns (PAMPs) are recognized by the immune system via pattern recognition receptors. Lipopolysaccharide (LPS), the key inducer of inflammation in Gram-negative bacterial infections, is also mainly cleared by the liver (Deng et al., 2013). Simultaneously, LPS can be a key component of sepsis by overactivating the innate immune system (Pfalzgraff and Weindl 2019). Previous studies have suggested that LPS-induced endotoxemia is only mediated by the activation of the cell surface LPS receptor Toll-like receptor (TLR) 4 (Pfalzgraff and Weindl 2019). However, the disappointing results of TLR4 inhibitors as anti-sepsis drugs in clinical trials indicate that an LPS-induced injury mechanism that is not related to TLR4 may play a key role (Cheng et al., 2017; Rathinam, Zhao, and Shao 2019).

Recent studies have shown that cytosolic LPS-mediated pyroptosis is the main driver of endotoxic shock (Kayagaki et al., 2011; Cheng et al., 2017; Rathinam, Zhao, and Shao 2019). Pyroptosis is a new type of programmed cell death that has been discovered and confirmed in recent years. It is characterized by its dependence on caspase and pore-forming protein (GSDMD) and is mediated by a large number of pro-inflammatory factors (Man, Karki, and Kanneganti 2017; Lu et al., 2020). Pathways activated by pyrolysis include the canonical pathway, which depends on caspase-1, and the non-canonical pathway, which depends on caspases-4/5/11 (Ding et al., 2016; Lu et al., 2020). The loss of caspase-11, but not of caspase-1, protects mice from LPS-induced cell death, whereas intracellular LPS triggers caspase-11-dependent inflammasome activation in the cytoplasm independently of TLR4 (Kayagaki et al., 2011; Abu Khweek and Amer 2020). Caspase-11 is the direct sensor of intracellular LPS and can be directly activated by binding to LPS (Shi et al., 2014). However, activated caspase-11 can mediate the activation of the non-canonical NOD-, LRR- and pyrin domain-containing protein 3 (NLRP3) inflammasome, leading to caspase-1 activation and interleukin (IL)-1β maturation, which is likely mediated via potassium efflux through GSDMD-N-terminal (NT) pores (Rühl and Broz 2015). The human orthologues of murine caspase-11 are caspases-4 and 5, which can also be similarly activated by intracellular LPS, culminating in GSDMD-NT-mediated pyroptosis (Kayagaki et al., 2015). Thus, caspases-11/4/5 play an essential role in defending against intracellular bacterial infection upstream of the canonical NLRP3 inflammasome. However, currently, few known drugs can control caspase-11 activation. Inhibition of caspase-11 may be a new way to prevent sepsis caused by Gram-negative bacteria.

Ubiquitination is one of the most prevalent protein post-translational modifications and plays a crucial regulatory role in inflammatory cells (Zinngrebe et al., 2014). Nedd4 is a HECT E3 ubiquitin ligase that is widely expressed in mammalian tissues (Ingham, Gish, and Pawson 2004). Nedd4 is well known to play an important role in development and physiological growth (Cao et al., 2008). Moreover, Nedd4 regulates immune cell functions and plays important regulatory roles in adaptive immunity (Liu et al., 2017; Gao et al., 2021). In natural immunity, Nedd4 has an inhibitory effect on non-classical inflammasomes. It can directly bind to caspase-11 to induce its ubiquitination and degradation. The survival rate of Nedd4 ± mice after LPS challenge is significantly reduced, and serum IL-1β is significantly increased. Here, the heart and liver were determined to suffer serious inflammatory damage (Liu et al., 2019). At the same time, Nedd4 can catalyze the polyubiquitination of pro-IL-1β (Bulek et al., 2020).

Previous reports have confirmed that RAW 264.7 cells lack apoptosis-associated speck-like protein containing a CARD domain (ASC) expression (Lin et al., 2016). ASC is essential for the activation of caspase-1 in the classic NLRP3 inflammasome pathway. Thus, RAW 264.7 cells constitute an ideal cell model for studying non-classical pyroptosis, which does not depend on ASC/caspase-1. Through rigorous and systemic analysis, we were able to screen out samotolisib (ST) from 441 potential candidates in the FDA-approved pyroptosis compound library. We demonstrated that ST effectively suppressed LPS-induced caspase-11 activation and pyroptosis in RAW264.7 cells. Subsequently, we investigated how ST alleviated inflammatory infiltration and caspase-11 activation in liver tissue from ALI mice. Further molecular analysis showed that ST hepatoprotection was mediated by inhibiting LPS-induced hepatocyte pyroptosis via phosphoinositide 3-kinase (PI3K)/AKT/mammalian target of rapamycin (mTOR) signaling-mediated Nedd4 expression.

## Materials and Methods

### Reagents and Antibodies

The primary antibodies used in this study were as follows: anti-NLRP3 (ab4207), anti-IL-18 (ab68435), anti-IL-1β (ab2105), anti-ASC (ab127537), anti-caspase-1 (ab179515), and anti-GSDMD-N (ab215203) purchased from Abcam (Cambridge, United Kingdom); anti-Caspase-11 (sc-56038) purchased from Santa Cruz Biotechnology (Dallas, United States of America); anti-ubiquitin (10201-2-AP), anti-Nedd4 (21698-1-AP), anti-GAPDH (60004-1-Ig), and horseradish peroxidase (HRP)-conjugated secondary antibodies (SA00001-2) purchased from Proteintech (Wuhan, China); and anti-Neutrophil (bs-19701R) purchased from Biosynthesis Biotechnology Co. (Beijing, China). Cy3 goat anti-mouse IgG (H + L) (A0521) and FITC goat anti-rabbit IgG (H + L) (A0562) used for immunofluorescence analysis were purchased from Beyotime (Shanghai, China). FuGENE-HD transfection reagent (E2311) was purchased from Promega (Madison, United States of America). ELISA kits for IL-1β and IL-18 were purchased from Shanghai Enzyme-linked Biotechnology (Shanghai, China). The LDH activity detection kit (C0016) was purchased from Beyotime Institute of Biotechnology (Shanghai, China). Pyroptosis Compound Library (L7400), ST (S8322) were purchased from Selleckchem (Houston, TX). *Escherichia coli* LPS (L2630) and FITC-conjugated LPS from *E. coli* 0111:B4 (F3665) were purchased from Sigma-Aldrich (St. Louis, MO).

### Establishment of the Acute Liver Injury Mouse Model

Six-to-eight-week-old male C57BL/6 mice, weighing 18–21 g, were purchased from Chengdu Dashuo Experimental Animal Company (Chengdu, China). All mice were raised in specific pathogen-free animal housing and provided with bacteria-free food and water. Mice were randomly divided into four groups (group 1: vehicle group; group 2: 10 mg/kg LPS; group 3: 5 mg/kg ST; group 4: 5 mg/kg ST + 10 mg/kg LPS, *n* = 6 in each group). Then, the mice were sacrificed, and tissues and blood were collected. Sera were isolated from blood using centrifugation at 4000 rpm at room temperature and stored at −80°C for biochemical analysis. Tissues were fixed in a 4% formalin solution and embedded in paraffin for histological analysis. All animal procedures were approved by the Animal Policy and Welfare Committee of Chengdu Medical College (CDYXY-2019036).

### Cell Lines and Cultures

RAW264.7 cells were maintained in DMEM supplemented with 10% fetal bovine serum at 37°C with 5% CO_2_. All media and supplements were purchased from Invitrogen (Carlsbad, CA, United States).

### Flow Cytometry

Flow cytometry was used to measure pyroptosis using an Annexin V-PE/7-AAD Detection Kit (KeyGEN, Jiangsu, China), according to the manufacturer’s instructions. RAW264.7 cells were pre-treated with or without ST at 2.5, 5, and 10 μM, and then harvested, washed with phosphate-buffered saline (PBS), and stained with 7-AAD for 15 min. After the reaction, 450 μl binding buffer was added, followed by incubation with 1 μl Annexin V-PE at 37°C in the dark for 15 min. Finally, the cells were analyzed using a flow cytometer (FACSCalibur, Becton-Dickinson, Franklin Lakes, NJ, United States).

### Cell Death Assessment

Cells were seeded in 96-well plates and primed with 1 mg/ml Pam3CSK4 in Opti-MEM for 4 h. Subsequently, the cells were treated with the different compounds for specific time periods and then transfected with 1 ug/mL LPS plus FuGENE-HD (2.5%, v/v) in Opti-MEM for 16 h (Ye et al., 2021). Lytic cell death was determined using a lactate dehydrogenase (LDH) activity detection kit.

### Liver Indices

Liver tissues were weighed, and liver indices calculated as the ratio of liver weight to body weight.

### Hematoxylin and Eosin, Periodic Acid–Schiff , and TUNEL Staining

Mice were anaesthetized using 0.6% pentobarbital sodium (40 mg/kg), and liver tissues were collected and fixed with 4% paraformaldehyde (in PBS) for 48 h at room temperature, dehydrated using an ethanol gradient, cleared with xylene, embedded in paraffin, and cut into 5 μm sections.

To evaluate the degree of inflammatory cell infiltration, liver sections were stained using an H&E staining kit (Beyotime Biotechnology, Shanghai, China). The sections were dewaxed, dehydrated, washed with PBS, and then stained with hematoxylin for 6 min and eosin for 2 min. We evaluated the distribution of liver glycogen using a PAS staining kit (Solarbio, Beijing, China). DNA fragmentation was detected by a TUNEL apoptosis detection kit (KeyGen, Nanjing, China).

### Immunohistochemistry Analysis

IHC was performed using an SP link detection kit (ZSGB-BIO Technology, Beijing, China). Tissue sections were dewaxed, dehydrated, and washed with PBS. Subsequently, the samples were boiled in citrate buffer (pH 6.0) for antigen retrieval and blocked with 5% normal goat serum at 37°C for 1 h. The sections were then incubated overnight at 4°C with the primary antibodies (1:200). After washing with PBS, the sections were incubated with the corresponding secondary antibody for 30 min. Finally, diaminobenzidine was used as the chromogen to visualize the immunocomplexes, and the sections were counterstained with hematoxylin. Images of random liver sections were captured at ×40 and ×20 magnifications using a microscope (XI 71 Olympus, Tokyo, Japan).

### Immunofluorescence Staining

The liver tissues were embedded in optimal cutting temperature compound and cut into 10 μm sections for IF staining. After washing with PBS, tissue sections were blocked with 5% BSA for 30 min at 37 C and then incubated overnight at 4 C with primary antibodies. After washing, the sections were incubated with secondary antibodies, Cy3 goat anti-rabbit/mouse IgG (H + L) or Alexa Fluor 488 goat anti-mouse/rabbit IgG (H + L) (1:200) for 1 h at 37°C, and nuclei were stained with 4,6-diamidino-2-phenylindole (DAPI). Fluorescent images were captured at a ×40 magnification using a fluorescence microscope.

### Survival Analysis

After LPS injection, the survival status of the mice was monitored at least every 6 h for two consecutive days. Survival curves were constructed using the GraphPad Prism 7.0 software.

### Immunoprecipitation

Liver samples were lysed on ice with immunoprecipitation lysis buffer (Beyotime) containing a protease inhibitor cocktail (Beyotime). Samples were centrifuged at 15,000 × *g* for 15 min, and 200 μl supernatant was transferred to a new tube as the input. Approximately 500 to 1,000 μg of protein was incubated with 1 μg of antibody overnight at 4 C. In total, 30 μl protein A/G-agarose beads (Beyotime) were added to each sample and incubated for another 2 h at 4 C the next day. The beads were then washed thrice with immunoprecipitation lysis buffer. After the final centrifugation, the beads were boiled for 5 min with 60 μl SDS loading buffer (Beyotime), and samples were subjected to western blot analysis using standard protocols.

### Quantitative Reverse-Transcription Polymerase Chain Reaction

Total RNA was extracted from spinal cord tissue using a total RNA extraction kit (Solarbio, Beijing, China) according to the manufacturer’s instructions. Next, cDNA was synthesized using an iScript cDNA synthesis kit (Bio-Rad, Hercules, CA, United States). The mRNA levels of Il18, Il1β, Il6, Il10, tumor necrosis factor (TNF)-α, monocyte chemoattractant protein-1 (MCP-1), and Nedd4 were analyzed using qRT-PCR and the SYBR Green Supermix (Bio-Rad, Hercules, CA, United States). The primers were synthesized by Shanghai Shenggong. The 2^−ΔΔCT^ method was used to calculate the relative mRNA levels.

### Western Blot Assay

Liver tissues and cells were lysed in ice-cold RIPA lysis buffer (Beyotime Biotechnology, Shanghai, China). Protein concentrations were determined using a BCA reagent kit (Beyotime Biotechnology, Shanghai, China). Total protein (30 μg) was separated using 10% sodium dodecyl sulphate-polyacrylamide gel electrophoresis and transferred onto polyvinylidene fluoride membranes (Millipore, Billerica, MA, United States). The membranes were blocked in Tris-buffered saline with 5% non-fat milk and 0.5% bovine serum albumin for 2 h at room temperature and then incubated overnight at 4 C with primary antibodies (1:1,000). After washing, the membranes were incubated with secondary antibodies (1:5,000) for 1 h at 37 C. Blots were visualized with the chemiluminescent HRP substrate (Millipore) and quantified using the Quantity 5.2 software System (Bio-Rad).

### Cell Counting Kit 8 Assay

Cells were cultured in 96-well plates at 10,000 cells/well, and 24 h after irradiation and/or drug treatment, CCK-8 (C0038, Beyotime, Shanghai, China) was used to detect cell viability according to the manufacturer’s instructions.

### Statistical Analyses

All data are expressed as the mean ± SD. Statistical analysis was performed using the GraphPad Prism 7.0 software (GraphPad, San Diego, CA, United States) with one-way ANOVA, followed by post-hoc multiple comparisons with Tukey’s test. *p* < 0.05 was considered statistically significant.

## Results

### Identification of Compounds That can Suppress Caspase-11 Activation and Pyroptosis Through High-Throughput Screening

**FIGURE 1:**
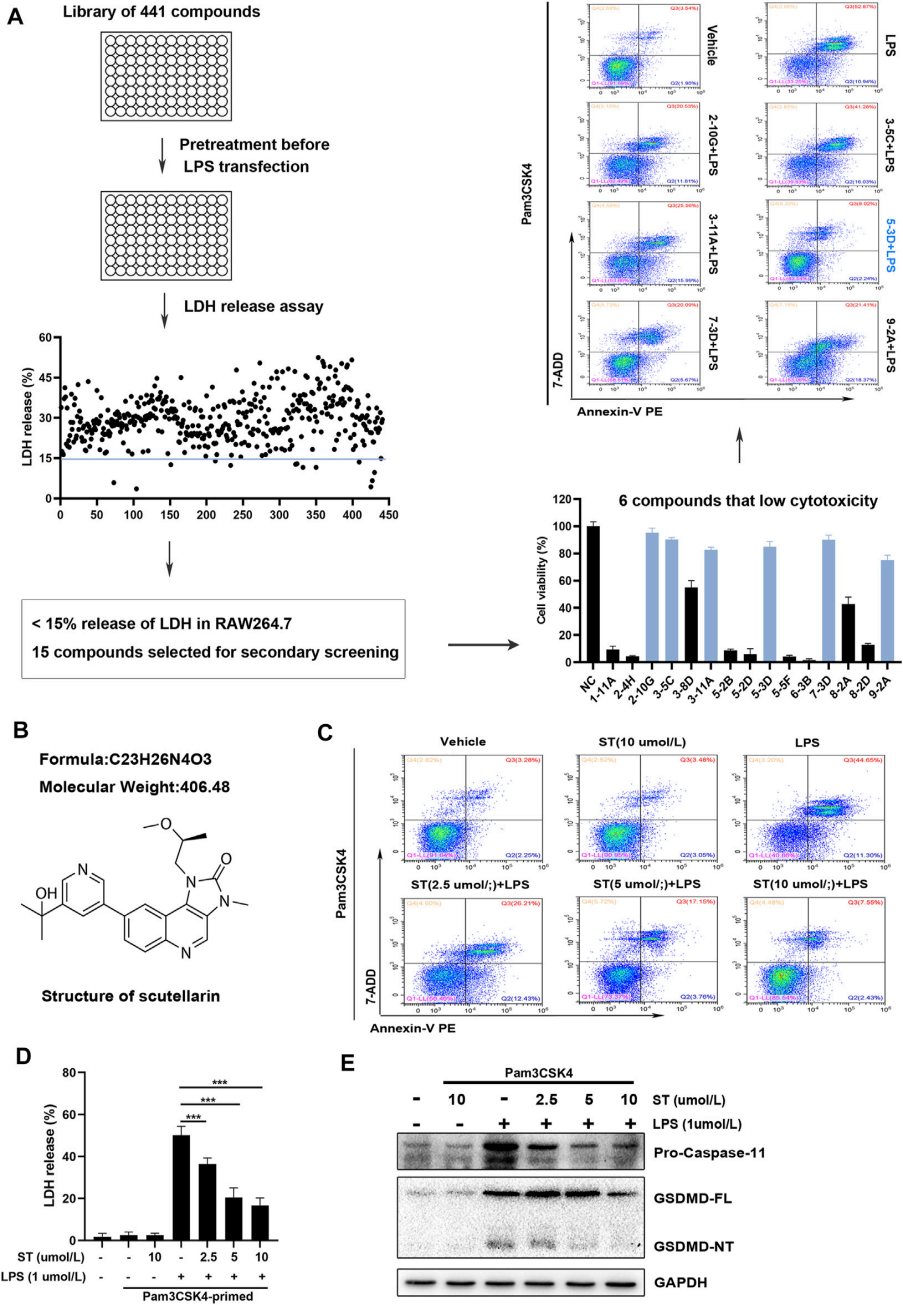
Screening for compounds with the ability to inhibit caspase-11 activation and lytic cell death in RAW264.7 cells. **(A)** Flow chart for primary/secondary screening and identification of compounds. **(B)** Chemical structure of samotolisib. **(C)** Percentage of Annexin V PE and 7-AAD double-positive RAW264.7 cells detected using flow cytometry. **(D)** Percentage of LDH release in the culture supernatants. **(E)** Western blot assay showing the levels of caspase-11 and GSDMD-NT in cell lysates. Glyceraldehyde 3-phosphate dehydrogenase (GAPDH) was adopted as a loading control.

To sufficiently activate caspase-11, appropriate PAMP priming is needed to stimulate the expression of pro-caspase-11 protein prior to transfection or delivery of LPS into the cytosol (Hagar et al., 2013). We initially performed this in RAW264.7 macrophages using Pam3CSK4 (a synthetic ligand for Toll-like receptor TLR2/1 as the priming reagent (Kayagaki et al., 2015). LPS transfection markedly increased caspase-11 activation, GSDMD-NT generation, and LDH release (Supplementary Figure S1). To identify compounds with the ability to inhibit caspase-11 activation, we screened a library of 441 compounds (Selleckchem compounds library) in RAW264.7 cells. Compounds were screened for their ability to inhibit caspase-11 activation and LPS-stimulated pyroptosis at a concentration of 10 μM using different detection methods (Figure 1A). Primary screening with LDH release revealed the ability of the compounds to inhibit lytic cell death after LPS transfection (Figure 1A). A total of 15 compounds that induced LDH release of less than 15% were selected for further evaluation in the secondary screening (Figure 1A). Subsequently, we confirmed the toxicity of these compounds using the CCK-8 assay. The six compounds confirmed in the secondary screening inhibited RAW264.7 cell growth by less than 25% at a concentration of 10 μM (Figure 1A). Flow cytometry assays further detected Annexin V-PE and 7-AAD double-positive cells in RAW264.7 cells pre-treated with these six compounds. The results showed that ST (C_23_H_26_N_4_O_3_, Figure 1B) induced the most significant inhibition of intracellular LPS-induced lytic cell death (Figure 1A). Next, we sought to verify the ability of ST to inhibit caspase-11 activation and pyroptosis at the concentrations of 2.5, 5, and 10 μM. Pre-treatment with ST decreased the number of Annexin V-PE and 7-AAD double-positive cells. ST alone did not increase the number of Annexin V-PE and 7-AAD double-positive cells (Figure 1C). Similarly, western blot and LDH release assay analyses showed that ST dose-dependently inhibited intracellular LPS-induced GSDMD-NT generation, caspase-11 activation, and LDH release. ST alone did not induce any such processes (Figures 1D,E). Together, these data indicate that ST is able to inhibit caspase-11 activation and pyroptosis in RAW264.7 cells.

### ST Pre-treatment Relieves Lipopolysaccharide-Induced Acute Liver Injury in Mouse Models

**FIGURE 2:**
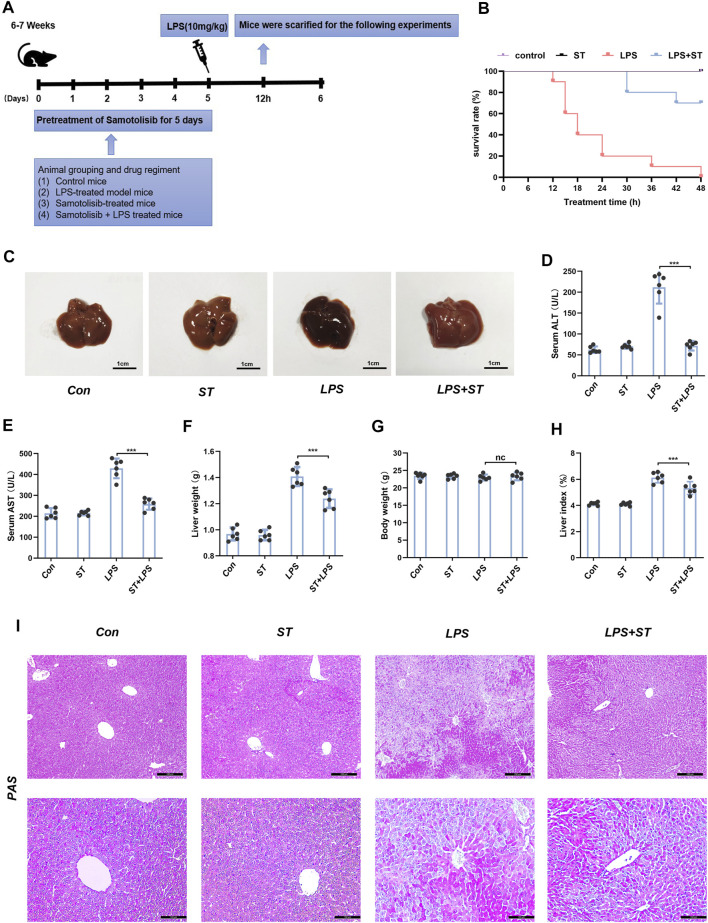
Samotolisib improved survival and liver injury induced by LPS. **(A)** Mice (*n* = 6 per group) were pre-treated with vehicle phosphate-buffered saline (PBS) or ST (5 mg/kg, respectively) 1 h prior to the LPS (10 mg/kg) challenge, respectively. **(B)** Survival curves. **(C)** Representative images of liver tissues. **(D)** Plasma levels of AST and **(E)** ALT. **(F)** Liver weight. **(G)** Body weight. **(H)** Liver index. **(I)** PAS staining of liver section (scale bars = 200 and 100 μm). Data are presented as the mean ± SD; *n* = 10 (for survival rate analysis) or 6; *p < 0.05, **p < 0.01, ***p < 0.005.

To investigate the protective role of ST in sepsis-induced liver damage, we challenged mice with a lethal dose of LPS. The result confirmed that ST pre-treatment dose-dependently improved the clinical symptoms in the ALI mice (Supplementary Figure S2). The appropriate dose of ST to attenuate the symptoms of ALI was 5 mg/kg, which was used for subsequent experiments. Subsequently, mice were randomly divided into seven groups (*n* = 6 per group). The experimental groups are shown in Figure 2A. After the establishment of the ALI model, the 48 h survival rate of mice in the control and ST groups was 100%. Mice began to die 12 h after LPS injection, and the survival rate was zero at 48 h. In the ST + LPS group, mice began to die 30 h after LPS injection. ST pre-treatment significantly prolonged the survival time in the ST group compared to that in the LPS group (Figure 2B). Pathological examinations were performed to determine the effects of ST on hepatic injury (Figure 2C). The activities of serum alanine aminotransferase (ALT) and aspartate aminotransferase (AST) significantly increased in the LPS group compared to those in the control group. ST pre-treatment significantly inhibited ALT and AST activities in the ST group compared to those in the LPS group (Figures 2D,E). The results of liver weight, body weight, and liver index showed that pre-treatment with ST markedly attenuated LPS-induced liver weight and liver index upregulation (Figures 2F–H). PAS staining of liver sections was performed to evaluate the effect of ST pre-treatment on the restoration of functional hepatocytes in the LPS-damaged liver. PAS staining demonstrated that mice treated with ST showed an uneven distribution of liver glycogen caused by LPS (Figure 2I). These results illustrate that ST plays a protective role against LPS-induced liver injury.

### ST Reduces LipopolysaccharideLPS-Induced Hepatic Inflammation in Mouse Models

**FIGURE 3:**
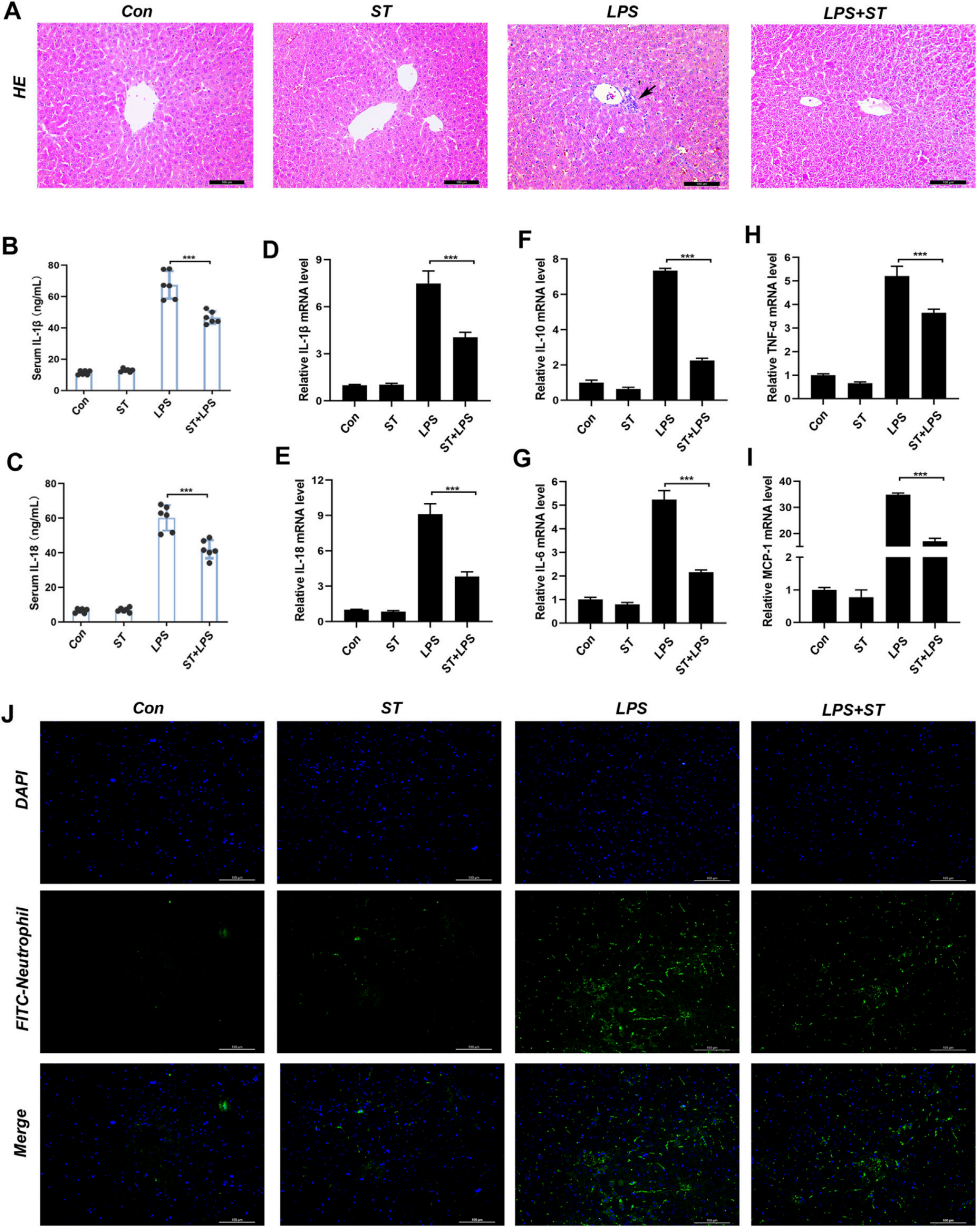
Samotolisib reduces the LPS-induced inflammatory response. Liver tissues were collected from the mice 12 h after LPS administration. The following analyses were performed: **(A)** H&E staining was performed on paraffin-embedded mouse liver sections. Inflammatory foci were observed using a microscope (Scale bar = 100 μm). **(B, C)** Serum IL-1β and IL-18 levels measured using ELISA. **(D–I)** mRNA expression of the inflammatory cytokines IL-18, IL-1β, IL-6, IL-10, MCP-1, and TNF-α, quantitated using qRT-PCR. **(J)** Immunofluorescence assays. Immunofluorescence was performed on frozen mouse liver sections. 4′,6-diamidino-2-phenylindole (DAPI) counter-staining for visualizing nuclei. Stained sections were observed and quantified using a fluorescence microscope (scale bars = 100 μm). Data are presented as the mean ± SD; *p < 0.05, **p < 0.01, ***p < 0.005.

Inflammation is one of the main causes of liver damage during sepsis (Feng et al., 2017). Previous reports have shown that the LPS-induced inflammatory response in the liver may be due to pyroptosis (Hu et al., 2020). H&E staining was used to analyze the inflammatory infiltration of the liver. In the control group, liver tissue revealed a normal hepatic architecture (Figure 3A). However, in the LPS group, the inflammatory infiltration of the liver was significantly increased. Mice pre-treated with ST presented less inflammatory infiltration (Figure 3A). The serum levels of IL-1β and IL-18 were significantly increased in the LPS group compared to those in the control group, and these increases were inhibited by ST pre-treatment (Figures 3B,C). We investigated the mRNA levels of inflammatory cytokines that mediate the development of liver injury. RT-qPCR analysis revealed that LPS injection also dramatically upregulated the expression of pro-inflammatory factors in the liver, including IL-18, IL-6, IL-10, MCP-1, and TNF-α. As expected, ST significantly reduced the expression of these inflammatory mediators (Figures 3D–I). ST inhibited the LPS-induced recruitment of neutrophils (Figure 3J). These results indicate that ST could inhibit the hepatic inflammatory response during ALI in mice.

### ST Relieves Lipopolysaccharide-Induced Caspase-11 Activation and Pyroptosis in the Liver

**FIGURE 4:**
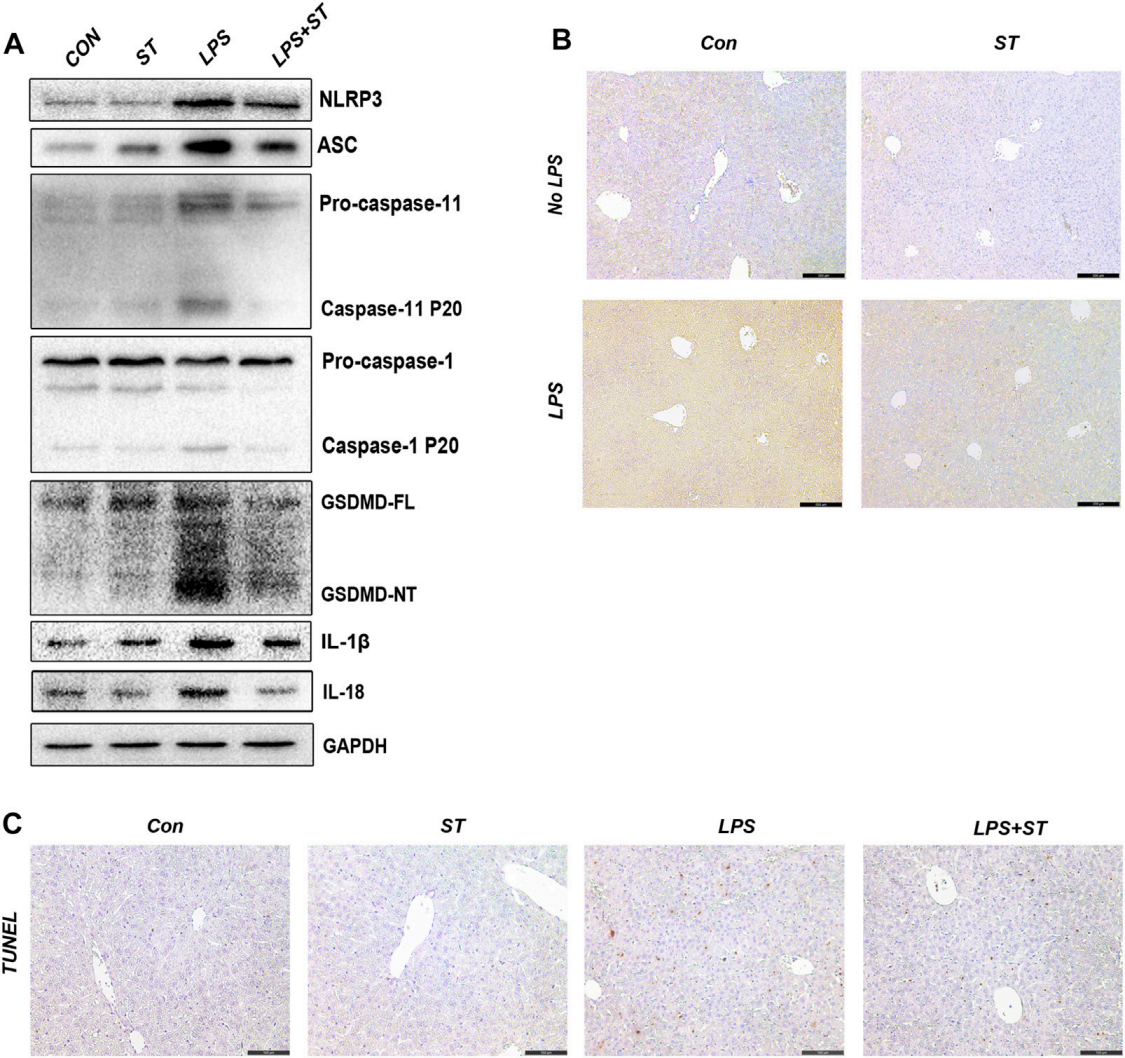
Samotolisib suppresses the activation of caspase-11/pyroptosis in LPS-induced liver injury. **(A)** The protein levels of pyroptosis markers assessed using western blotting. **(B)** Representative immunohistochemistry (IHC) images of caspase-11 in liver sections (scale bars = 200 μm). **(C)** TUNEL staining of liver sections (scale bars = 100 μm). Data are presented as the mean ± SD; *p < 0.05, **p < 0.01, ***p < 0.005.

To clarify whether ST suppresses ALI by inhibiting caspase-11 activation and pyroptosis, we evaluated the expression levels of pyroptosis-related proteins in the liver using western blotting. The results showed that the levels of caspase-11, GSDMD-NT, NLRP3, ASC, caspase-1, IL-1β, and IL-18 were increased in sepsis mice and that ST pre-treatment suppressed this effect (Figure 4A). Caspase-11 was highly expressed in the liver of sepsis mice, and ST pre-treatment remarkably inhibited caspase-11 expression (Figure 4B). Furthermore, compared with the control group, LPS remarkably increased the number of TUNEL-positive cells in the liver, whereas ST pre-treatment significantly reduced these numbers (Figure 4C).

### ST Promoted Caspase-11 Ubiquitination by Inhibiting PI3K/AKT/mTOR Signaling

**FIGURE 5:**
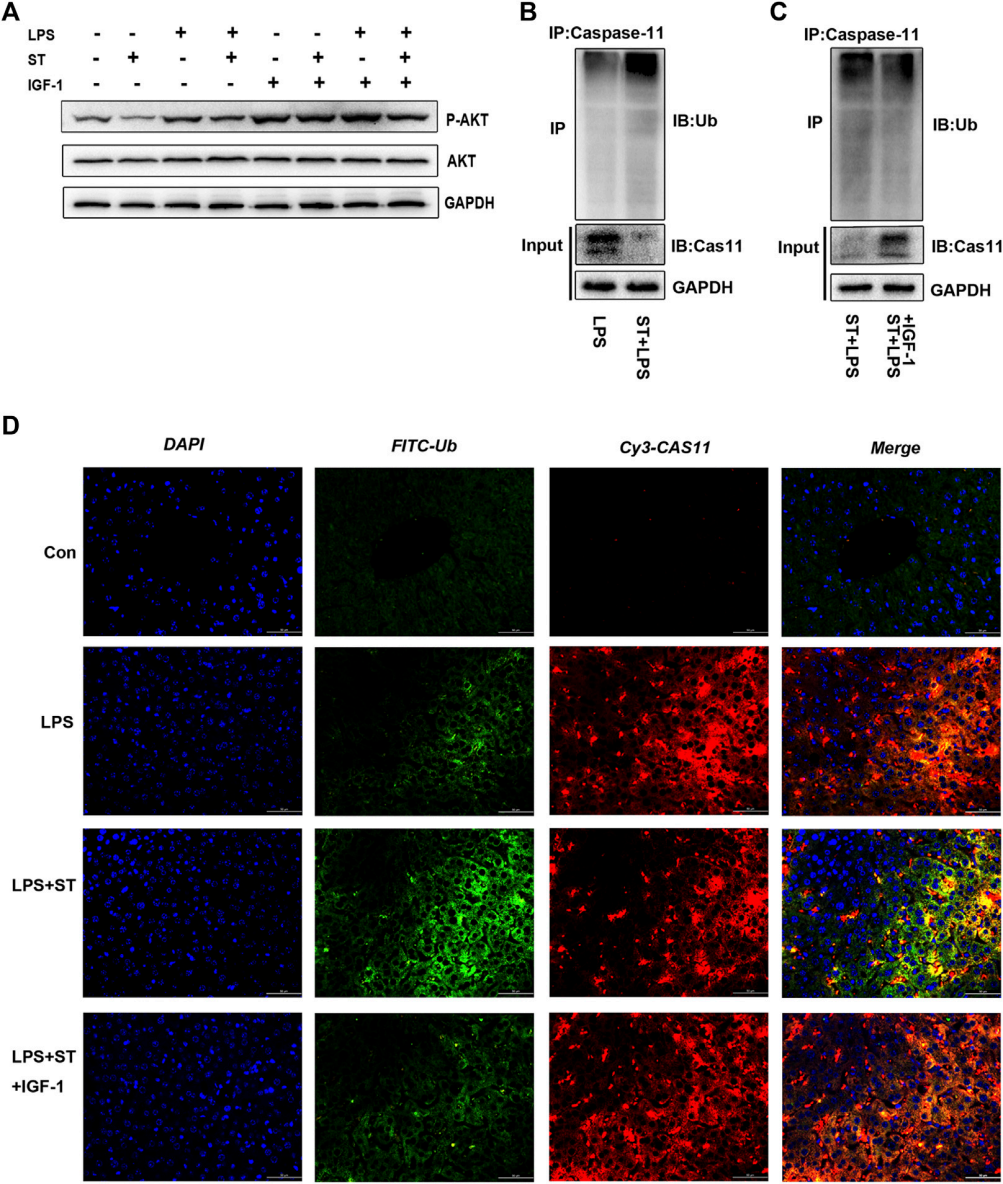
Samotolisib promoted caspase-11 ubiquitination by inhibiting PI3K/AKT/mTOR signaling. **(A)** Western blotting showing the protein levels of P-AKT and AKT. **(B, C)** Lysates of liver tissue immunoprecipitated with an anti-caspase-11 antibody and western blotting with the indicated antibody. **(D)** Immunofluorescence assays. Ubiquitin (Ub) was detected using an anti-Ub primary antibody and FITC goat anti-rabbit antibody; caspase-11 was detected using an anti-caspase-11 primary antibody and Cy3-labeled goat anti-mouse antibody; DAPI counter-staining was used to visualize nuclei (scale bar = 50 μm). Data are presented as the mean ± SD; *p < 0.05, **p < 0.01, ***p < 0.005.

ST is an inhibitor of class I PI3K isoforms and mTOR kinase (Smith et al., 2016). ST can inhibit PI3K/AKT/mTOR signaling, which can be reversed by IGF-1 (Figure 5A). The PI3K/AKT/mTOR pathway has attracted extensive attention as a modulator of autophagy (Xu et al., 2020). We first explored whether ST inhibited pyroptosis by inducing autophagy. Interestingly, the western blotting results showed that ST pre-treatment did not increase the expression of autophagy-related proteins (Supplementary Figure S3A), indicating that autophagy does not participate in the inhibitory effect of ST. Studies have shown that protein ubiquitination has a regulatory effect on the activation of inflammasomes and non-classical inflammasomes. To further elucidate the underlying mechanisms, we verified the level of caspase-11 ubiquitination in liver tissues. The ubiquitination levels of caspase-11 were highly increased and were sustained at high levels in the LPS + ST group compared with those in the LPS group (Figure 5B). Hence, ST may be involved in the regulation of related signaling pathways and of ubiquitin-mediated pyroptosis. Further, IGF-1 inhibited the high ubiquitination levels of caspase-11 in the LPS + ST group (Figure 5C). To this end, we performed double IF staining with caspase-11 and ubiquitin antibodies in the liver. Double IF analysis revealed the co-localization of caspase-11 with ubiquitin in the liver (Figure 5D). The above studies demonstrate that ST promotes the ubiquitination of caspase-11 through PI3K/AKT/mTOR signaling.

### ST Activates the Binding of Ubiquitinating Protein Nedd4 to Caspase-11

**FIGURE 6:**
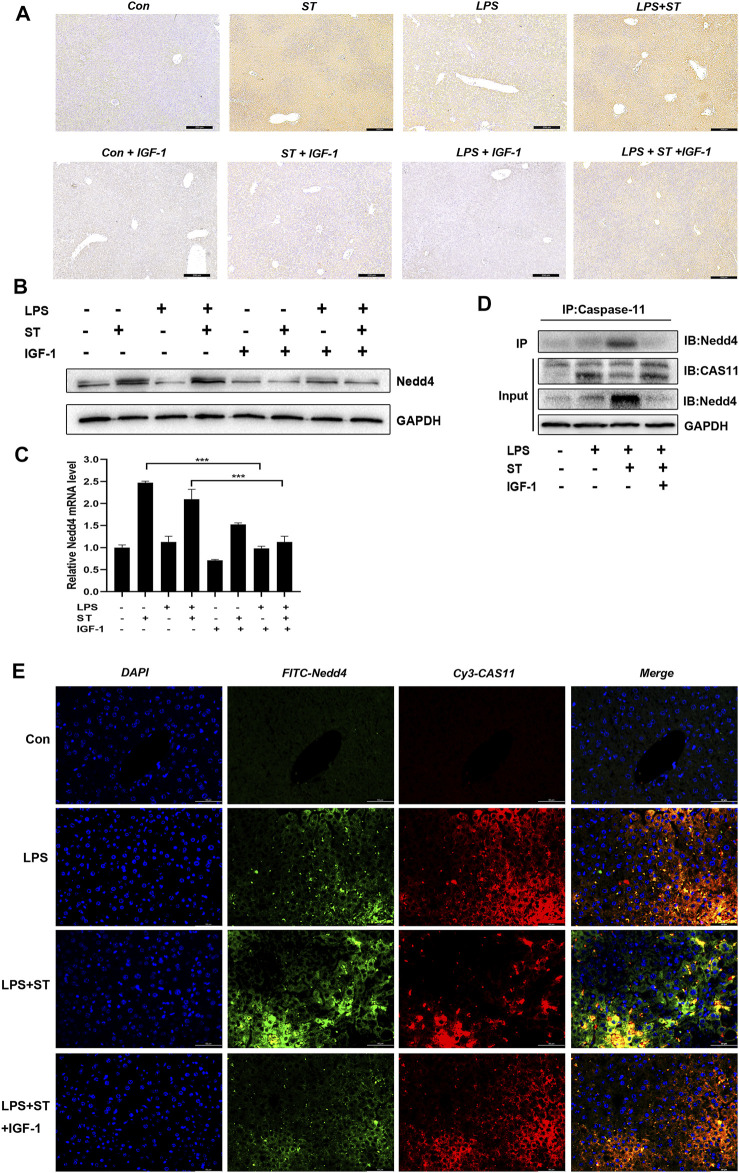
Nedd4 interacts with caspase-11 and targets it for degradation. **(A)** Representative immunohistochemical images of Nedd4 in liver sections (scale bars = 100 μm). **(B)** Western blotting showing the protein levels of Nedd4 and **(C)** qRT-PCR results. **(D)** Lysates of liver tissue immunoprecipitated with an anti-caspase-11 antibody and western blotting with the indicated antibody. **(E)** Immunofluorescence assays. Nedd4 was detected using an anti-Nedd4 primary antibody and FITC goat anti-rabbit antibody; caspase-11 was detected using an anti-caspase-11 primary antibody and Cy3-labeled goat anti-mouse antibody (scale bar = 50 μm). Data are presented as the mean ± SD; *p < 0.05, **p < 0.01, ***p < 0.005.

Nedd4 plays important regulatory roles in adaptive immunity. In particular, it was previously found that Nedd4 controls non-canonical inflammasome activation through polyubiquitination and subsequent degradation of caspase-11 (Liu et al., 2019). IHC, western blot, and RT-qPCR analysis demonstrated that the expression of Nedd4 was upregulated in the LPS + ST group compared with that in the LPS group and that IGF-1 inhibited the increase of Nedd4 (Figures 6A–C). Thus, we hypothesized that caspase-11 ubiquitination by Nedd4 might inhibit non-canonical inflammasome activation. To test this hypothesis, we used co-immunoprecipitation experiments, and double IF staining revealed that Nedd4 interacted with caspase-11 in the liver (Figures 6D,E).

## Discussion

Liver injury is strongly associated with poor outcomes in sepsis patients. Inhibition of caspase-11 activation in the liver is considered a potential therapeutic target for the treatment of sepsis (Liu et al., 2020). However, little is known about the therapeutic drugs that can target and inhibit caspase-11 (Ye et al., 2021). The present study revealed that ST inhibits caspase-11 activation, thereby reducing the impact of pyroptosis in hepatocytes. This ST effect is mediated through increased ubiquitination and subsequent degradation of caspase-11 via PI3K/AKT/mTOR signaling in hepatocytes.

In the current study, we screened 441 small molecule compounds with known targets in RAW264.7 cells. Primary screening resulted in the identification of 15 compounds that equally inhibited the release of LDH. We found some novel anti-inflammatory targets of the 15 compounds, including Hsp70, IL-5 receptor, and sirtuin 1. For example, sirtuin 1 has a significant active role, making it a new drug screening target, with a great developmental potential (James 2017b; Li et al., 2017; Li et al., 2018). LPS is a critical repressor of sirtuin 1 (James 2017c), which was recently found to be involved in non-alcoholic fatty liver disease and various organ diseases (James 2016; James 2017a). Of the 15 compounds, six compounds with inhibitory effects were identified following the secondary screenings, of which ST had the best effect on pyroptosis suppression. Subsequently, *in vivo* experiments confirmed that ST showed significant liver protection against LPS-induced acute liver damage. In addition, the liver plays a central role in inflammation during sepsis (Yan, Li, and Li 2014). ST pre-treatment can inhibit the expression and release of inflammatory cytokines and inflammatory infiltration in liver tissue.

Previous studies have demonstrated that some drugs can play an important role in alleviating LPS-induced liver injury by inhibiting the PI3K/AKT/mTOR signaling pathway (Liu et al., 2020; Shen et al., 2020). At the same time, activating autophagy through the PI3K/AKT/mTOR pathway is a common mechanism for treating infectious diseases (Campbell et al., 2018; Liu et al., 2020; Shen et al., 2020). In this study, we found that the expression of autophagy-related proteins did not increase after ST pre-treatment. Therefore, we considered the involvement of other mechanisms. Polyubiquitination is a post-translational modification that affects the activity and function of innate sensors and downstream signaling molecules through the covalent attachment of ubiquitin to lysine residues in the target protein (Liu, Qian, and Cao 2016). In canonical inflammasomes, protein ubiquitination has a regulatory effect on inflammasome activation. Studies have reported that the linear ubiquitination of ASC is necessary for the assembly of NLRP3 inflammasomes (Rodgers et al., 2014). The ubiquitination of DEAH-Box helicase 33 (DHX33) by tripartite motif-containing 33 is lysine 63-specific and is required for the formation of the DHX33-NLRP3 inflammasome complex (Weng et al., 2014). The ubiquitin-editing enzyme A20 downregulates the expression levels of NLRP3, pro-IL-1β, and IL-18 by blocking nuclear factor-κB (Duong et al., 2015). In addition, ubiquitination can respond to different levels of infection and danger signals and regulate non-canonical inflammasome activity. The results showed that caspase-11 was bound to ubiquitinated proteins in hepatocytes after LPS injection and that it was significantly upregulated during ST pre-treatment. However, IGF-1 reduced the ubiquitination levels of caspase-11 and prevented the inhibitory effect of ST on caspase-11 activation. In this study, we made two key discoveries. First, LPS induced pyroptosis in both the mouse liver and RAW264.7 cells lacking ASC, as evidenced by caspase-11 activation and GSDMD-NT generation (markers of pyroptosis). Second, caspase-11-mediated pyroptosis in the liver was alleviated by ST by increasing the ubiquitination level of caspase-11, which was attenuated by IGF-1. These findings indicate that LPS can activate non-canonical inflammasomes in the liver, and that this is inhibited by ST through the PI3K/AKT/mTOR pathway. Considering that ST could alleviate the increased liver damage and inflammation in the LPS group, our findings indicate that ST protects the liver through a ubiquitination-dependent mechanism and improves the systemic effects of LPS.

To further explore the mechanism by which ST inhibits caspase-11, we aimed to identify the protein responsible for regulating caspase-11 ubiquitination. In innate immunity, Nedd4l catalyzes the K29-linked ubiquitination of cysteine residues in TNF receptor-associated factor 3, which is key to innate antiviral immunity (Gao et al., 2021). Recent research found that Nedd4 is an important negative regulatory component of the non-canonical inflammasome pathway and that it directly interacts with caspase-11 and mediates the polyubiquitination of K48-linked caspase-11 at multiple K residues and subsequent degradation by the 26S proteasome. Simultaneously, caspase-11 mutually regulates Nedd4 through cleavage (Liu et al., 2019). Considering this finding and the fact that ST inhibits caspase-11 by enhancing ubiquitination, we hypothesized that ST may promote the expression of Nedd4 and its binding to caspase-11, leading to caspase-11 inhibition. We detected Nedd4 and its binding to caspase-11. The direct interaction between caspase-11 and Nedd4 was detected using immunoprecipitation-immunoblotting analysis. IF revealed that Nedd4 forms a complex with caspase-11 in liver cells, which is inhibited by IGF-1. In summary, these results indicate that ST interacts with and ubiquitinates caspase-11 through Nedd4, thereby inhibiting LPS-induced caspase-11 activation in hepatocytes, ultimately leading to caspase-11-mediated pyroptosis inhibition in hepatocytes, which is manifested by the reduction of liver damage and improvement of the general condition during sepsis. However, the type of Nedd4 ubiquitination involved and the location of caspase-11 require further study.

In conclusion, we have shown that ST protects the mouse liver from LPS-induced experimental sepsis. This action of ST is achieved through the inhibition of intracellular LPS-induced pyroptosis, which is dependent on PI3K/AKT/mTOR signaling-mediated Nedd4 expression. These findings suggest that ST can be used as a potential drug for the prevention of liver injury in patients with sepsis.

## Data Availability

The datasets presented in this article are not readily available because All data generated or analysed during this study are included in this published article. Requests to access the datasets should be directed to zyy631700543@163.com.

## Supplementary Material

Supplementary Figure 1 LPS activated non-canonical pyroptosis in RAW264.7 cells. **(A)** Western blotting showing the protein levels of caspase-11 and GSDMD-NT in cell lysates. **(B)** Percentage of LDH release in the culture supernatants. **(C)** LPS in cytoplasm of RAW264.7 cells. After transfected with FITC-LPS for 16 h, the cells were counter stained with DAPI. The staining was observed using a fluorescence microscope. **(D)** LPS abundance in RAW264.7 cells was indicated by the FITC fluorescence intensity that measured by a fluorescence microplate reader at excitation/emission wavelengths of 490/530 nm; *P < 0.05, **P < 0.01, ***P < 0.005.

Supplementary Figure 2 Different concentrations of samotolisib improved pathological liver injury induced by LPS. **(A)** Plasma levels of AST and. **(B)** and ALT.

Supplementary Figure 3 Samotolisib cannot regulate autophagy and Sirtuin 1 in acute liver injury. **(A)** Western blotting showing the protein levels of Beclin-1, ATG5, P62, LC3. **(B)** Western blotting showing the protein levels of Sirtuin 1.

## Abbreviations

ALI, acute liver injury; ALT, alanine aminotransferase; AST, aspartate aminotransferase; DAPI, 4′,6-diamidino-2-phenylindole; H&E, hematoxylin and eosin; IL, interleukin; LPS, lipopolysaccharide; TNF-α, tumor necrosis factor-α; ST, samotolisib; GAPDH, glyceraldehyde phosphate dehydrogenase; PI3K, phosphoinositide 3-kinase; mTOR, mammalian target of rapamycin; GSDMD-NT, N-terminal fragment of gasdermin D; IGF-1, insulin-like growth factor 1; MCP-1, monocyte chemoattractant protein-1; IHC, immunohistochemistry; IF, immunofluorescence; CCK-8, cell counting kit 8; DAPI, 4,6-diamidino-2-phenylindole.
