# Supplementary figures and images for: Samotolisib Attenuates Acute Liver Injury Through Inhibiting Caspase-11-Mediated Pyroptosis Via Regulating E3 Ubiquitin Ligase Nedd4 (part 1 of 2)
Source: Front Pharmacol. 2021 Aug 13;12:726198. doi: 10.3389/fphar.2021.726198 (PMC8414251; doi:10.3389/fphar.2021.726198)

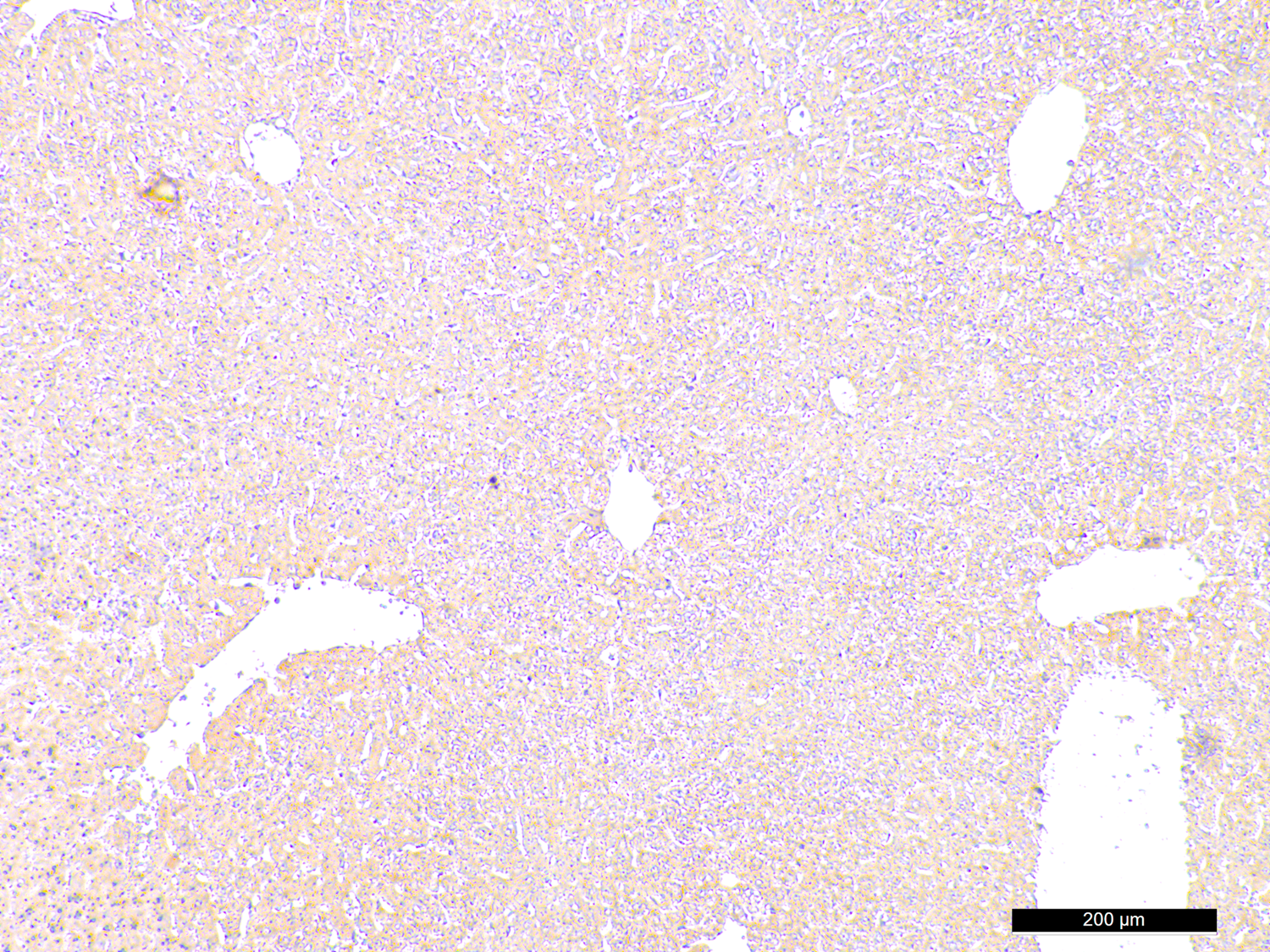

Supplement: Supplementary file 1 [file DataSheet3.ZIP › CN+IGF.tif]

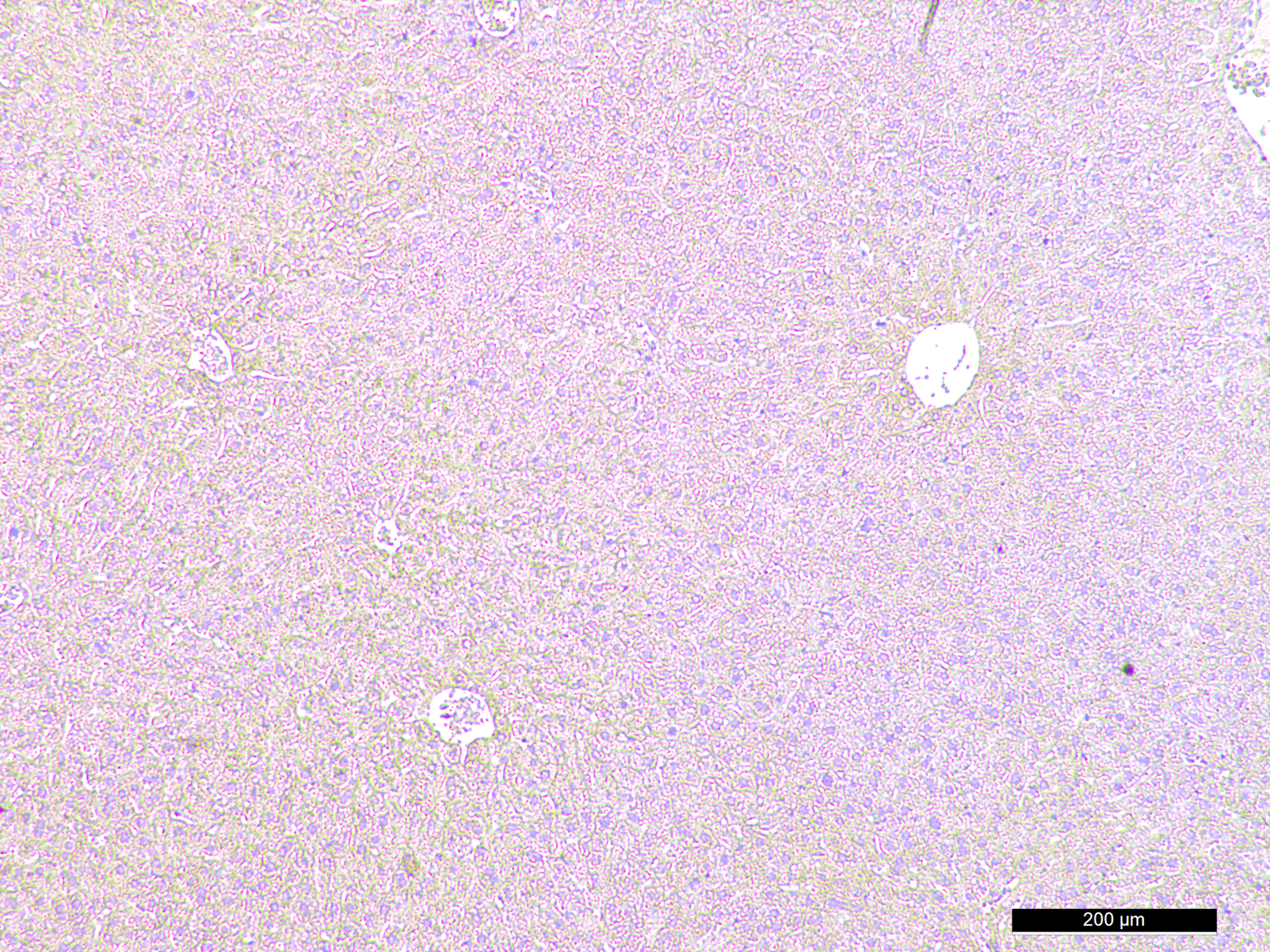

Supplement: Supplementary file 1 [file DataSheet3.ZIP › CN.tif]

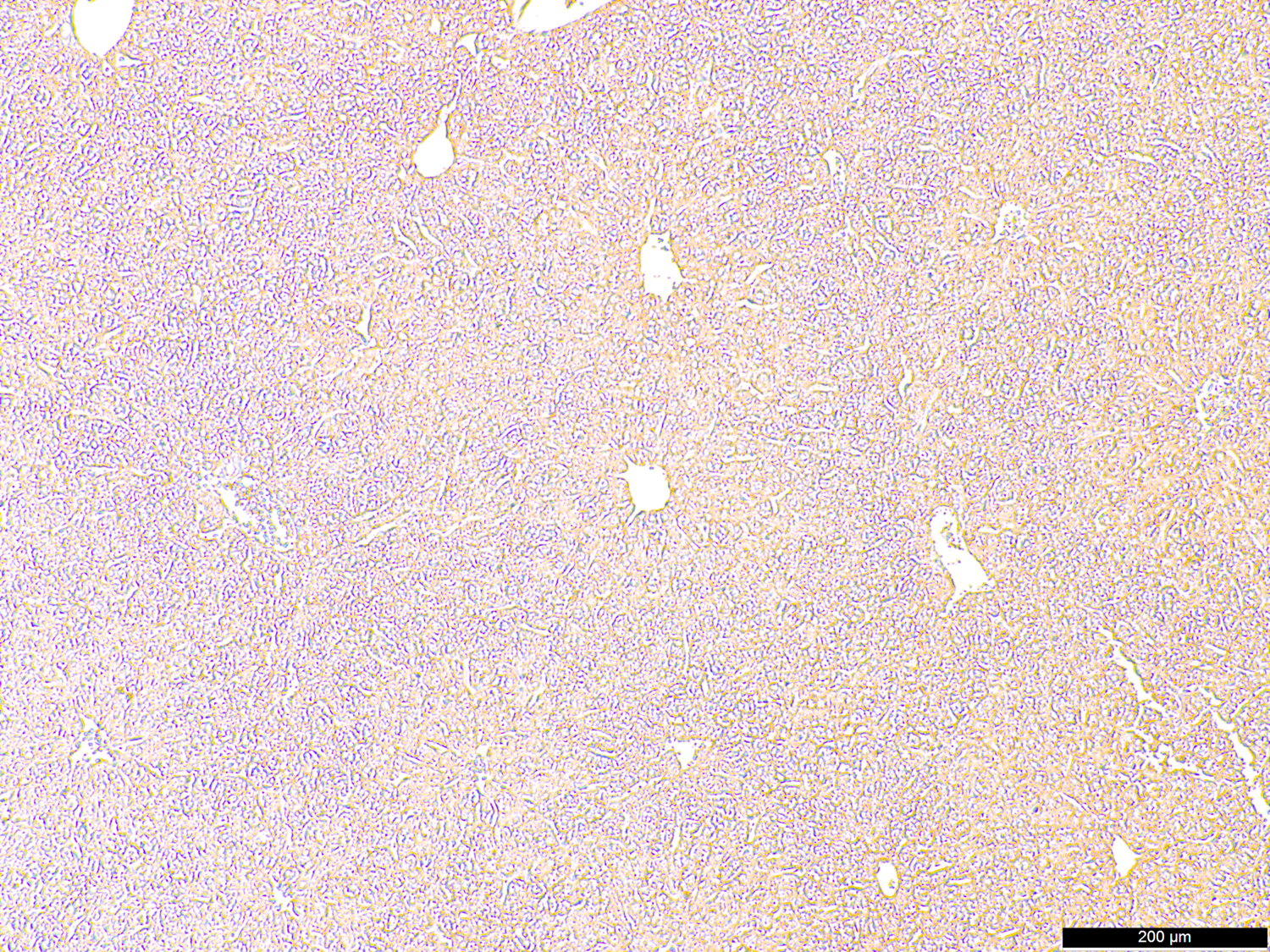

Supplement: Supplementary file 1 [file DataSheet3.ZIP › L+S+G.tif]

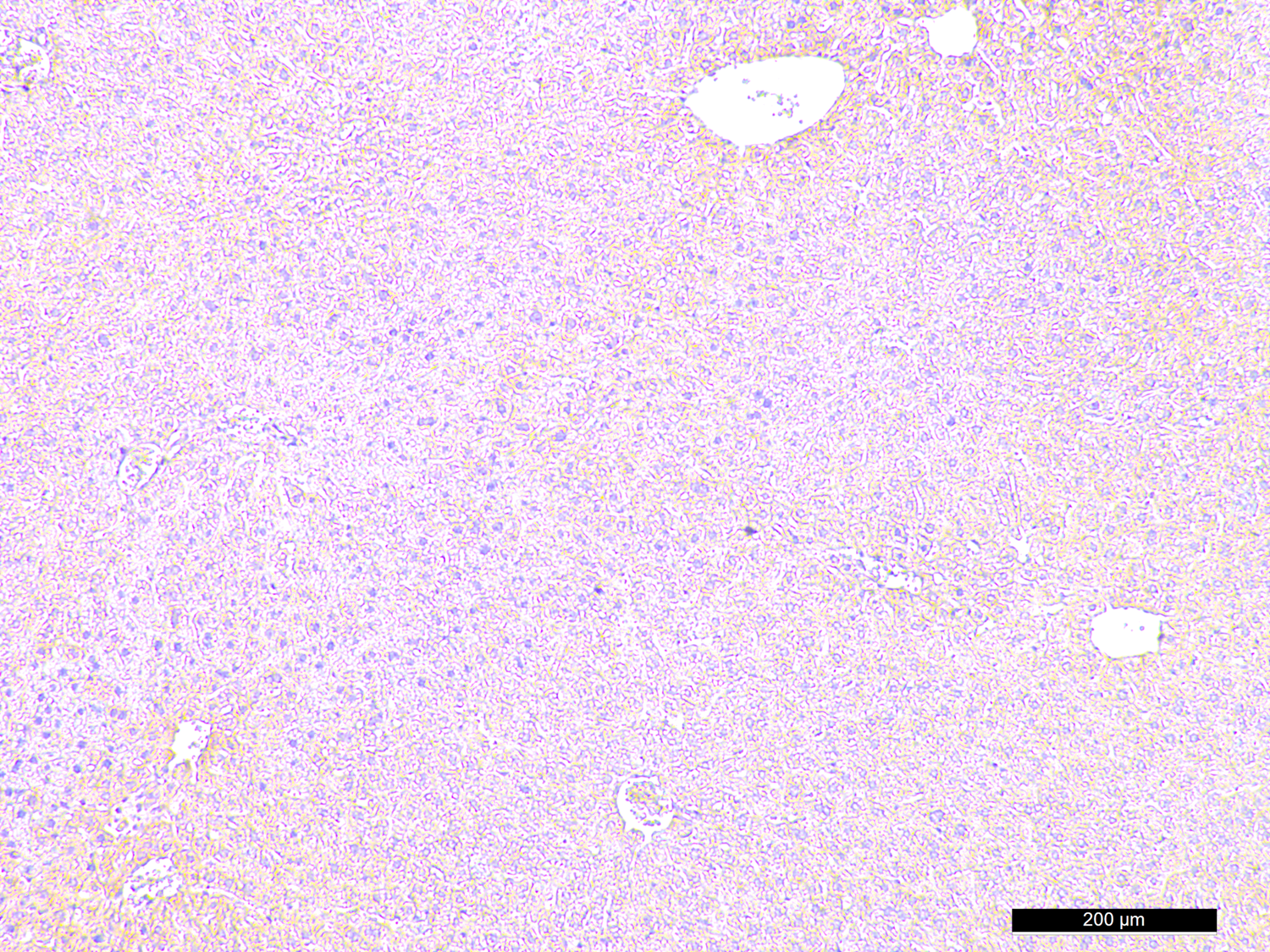

Supplement: Supplementary file 1 [file DataSheet3.ZIP › LPS+IGF.tif]

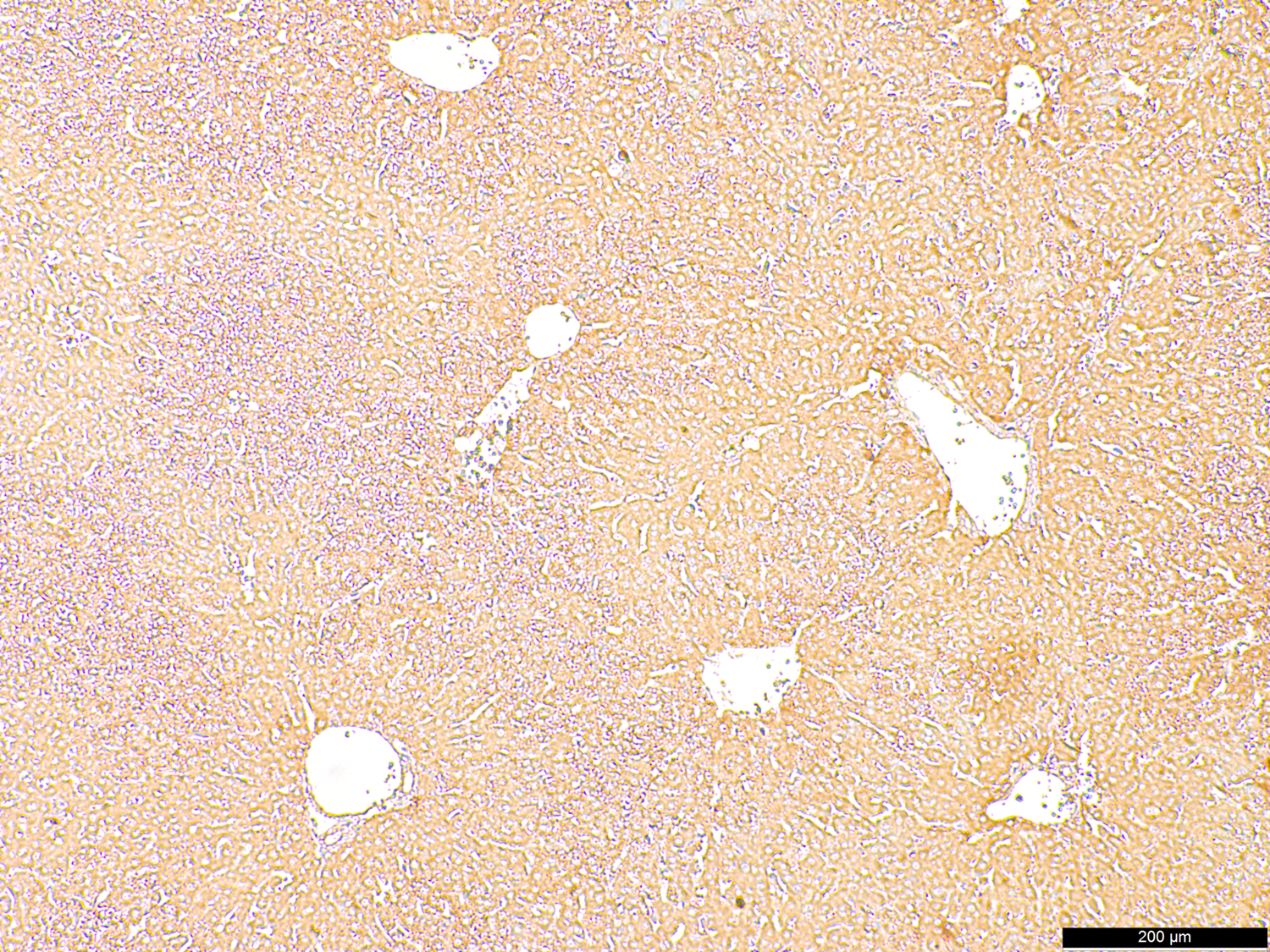

Supplement: Supplementary file 1 [file DataSheet3.ZIP › LPS+ST.tif]

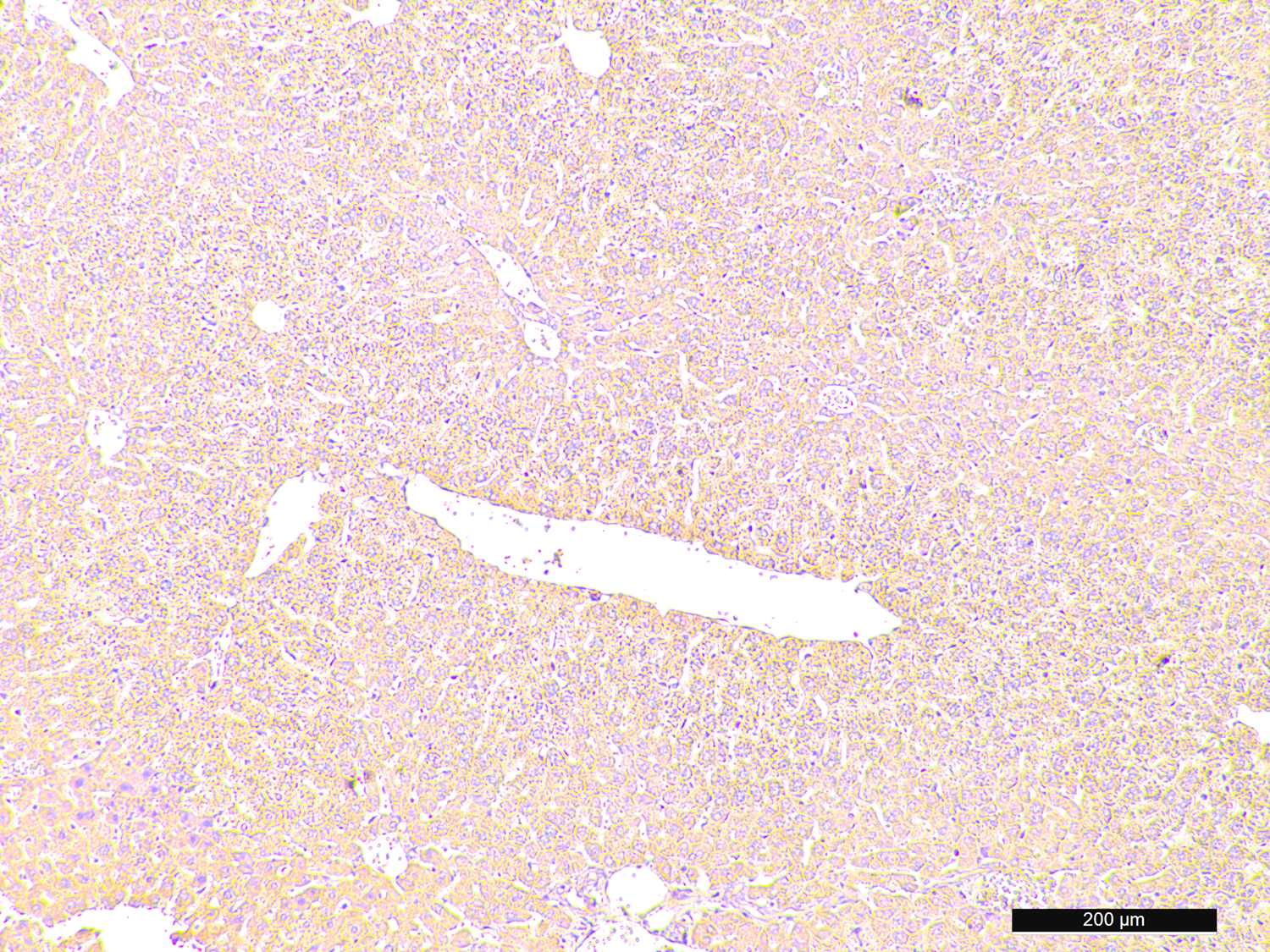

Supplement: Supplementary file 1 [file DataSheet3.ZIP › LPS.tif]

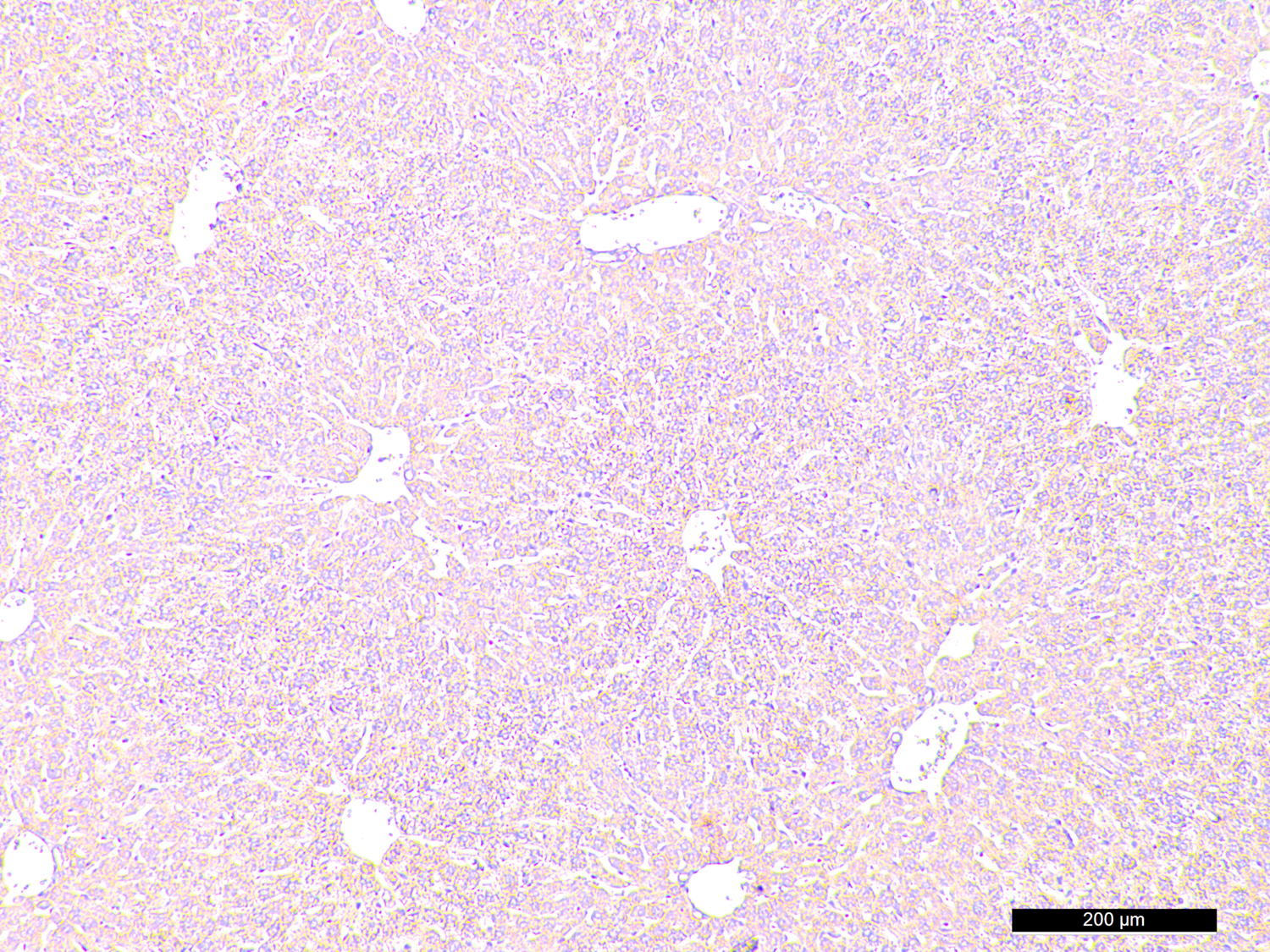

Supplement: Supplementary file 1 [file DataSheet3.ZIP › ST+IGF.tif]

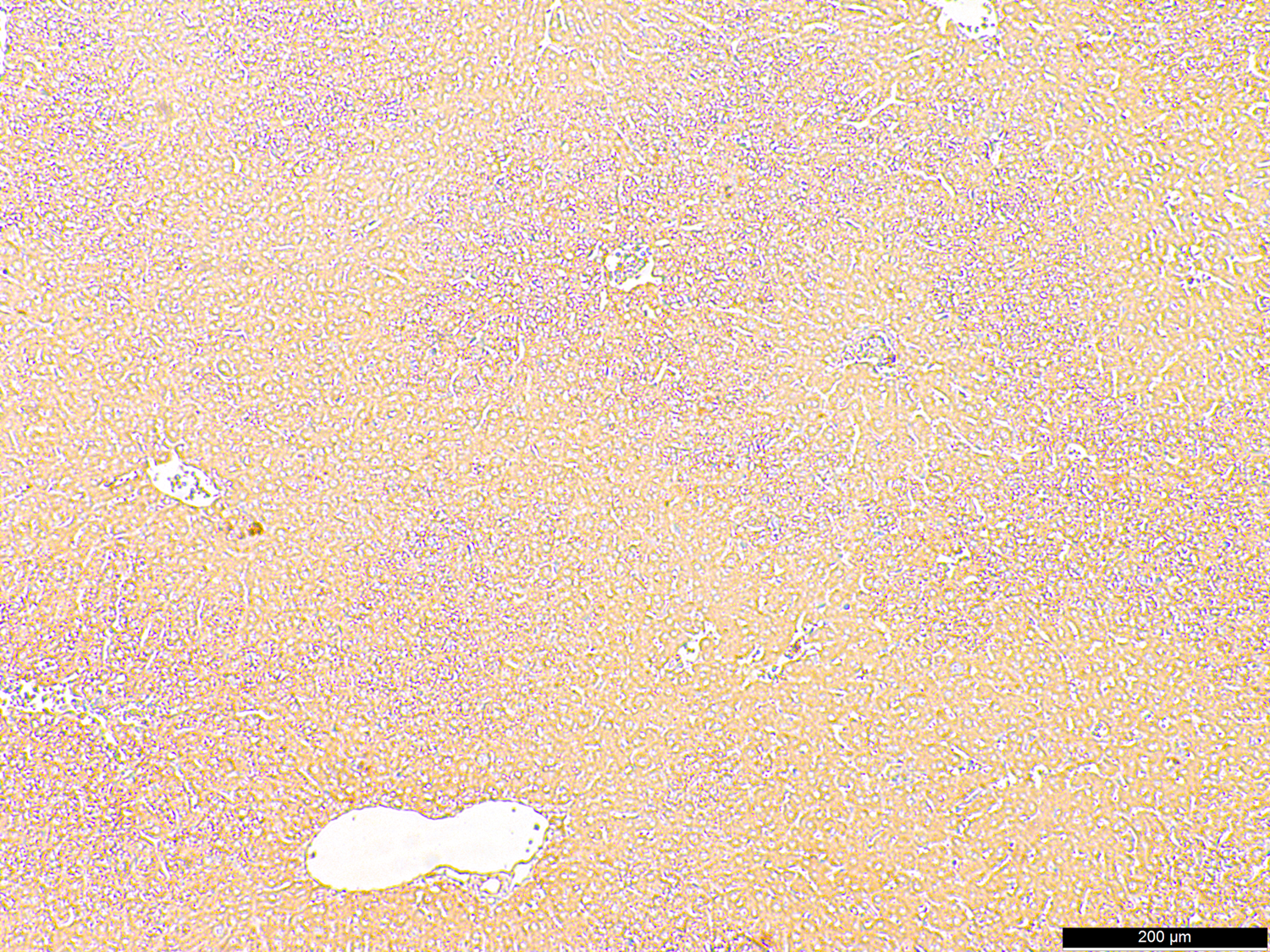

Supplement: Supplementary file 1 [file DataSheet3.ZIP › ST.tif]

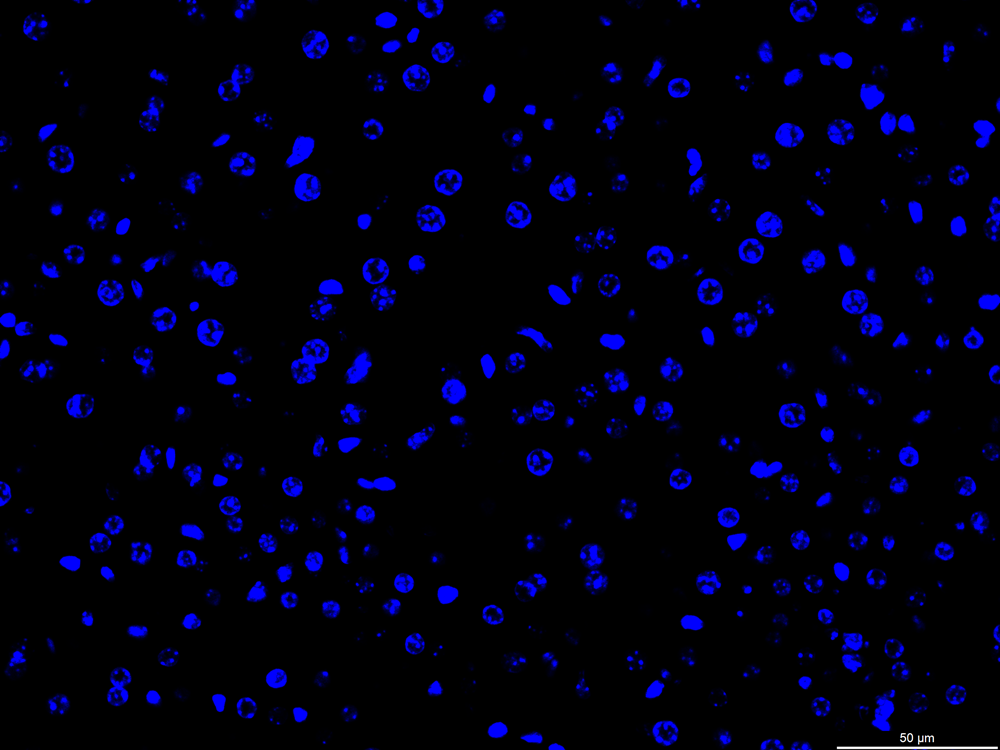

Supplement: Supplementary file 2 [file DataSheet11.ZIP › G.tif]

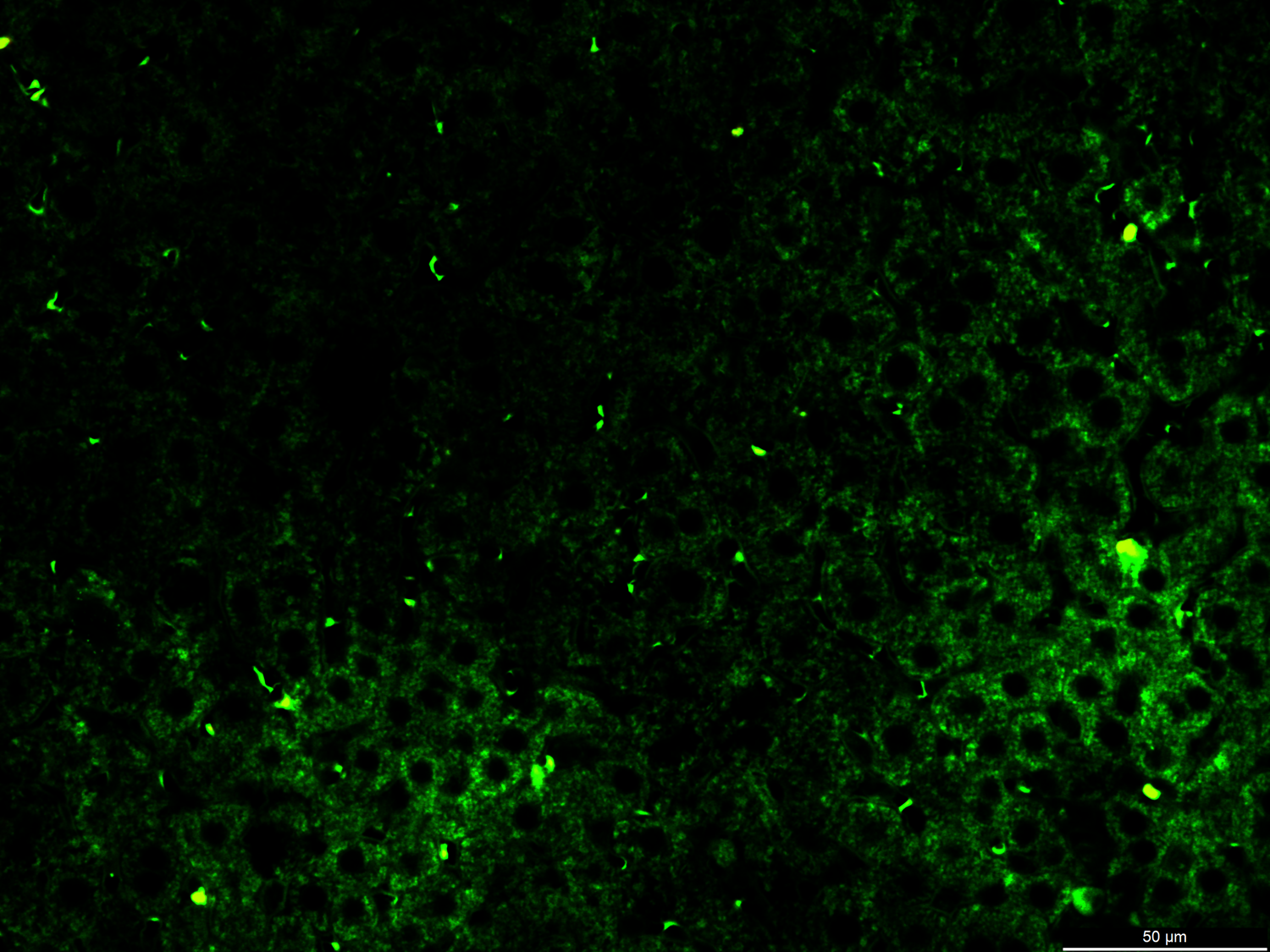

Supplement: Supplementary file 2 [file DataSheet11.ZIP › GL.tif]

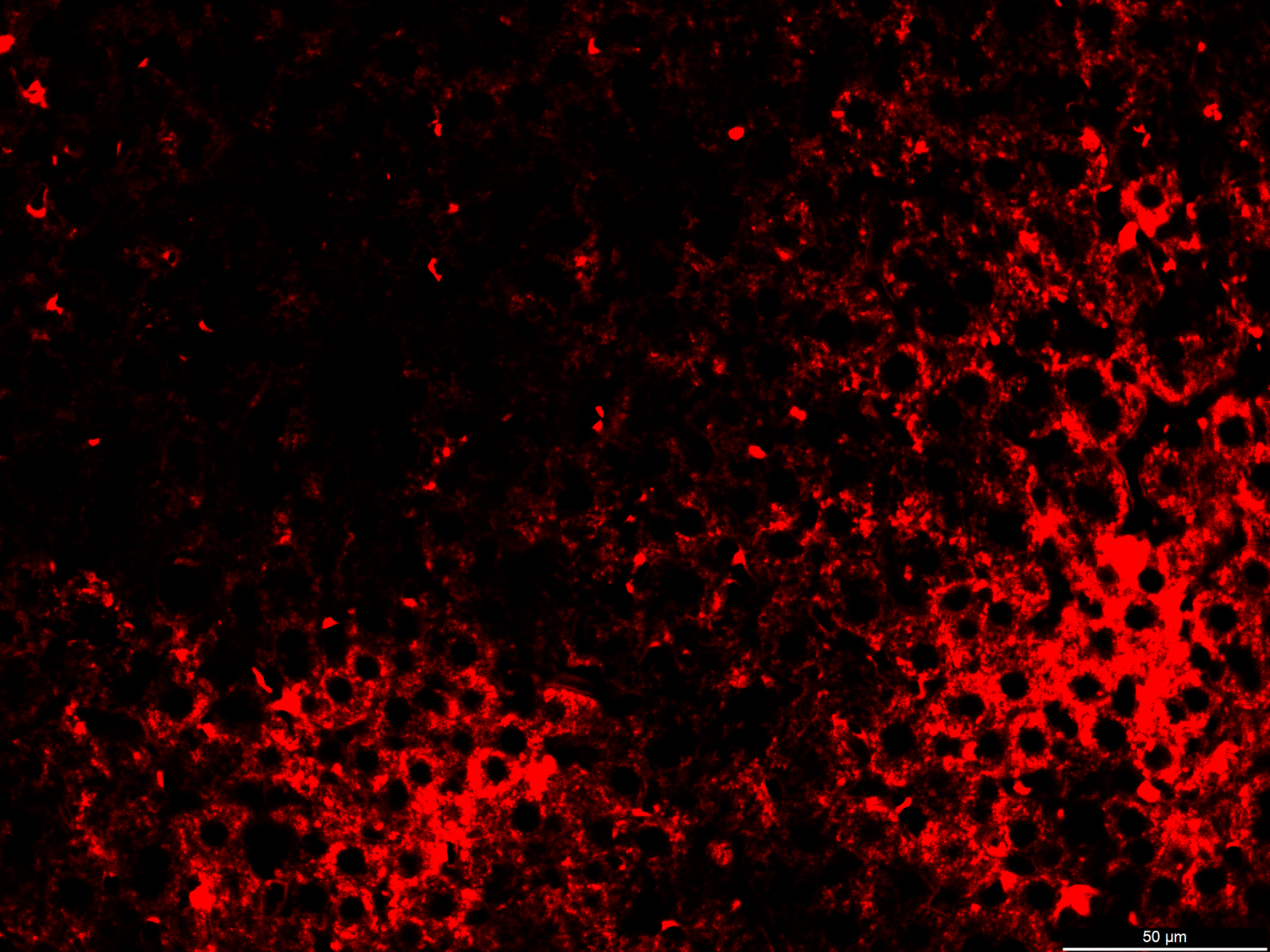

Supplement: Supplementary file 2 [file DataSheet11.ZIP › GLS.tif]

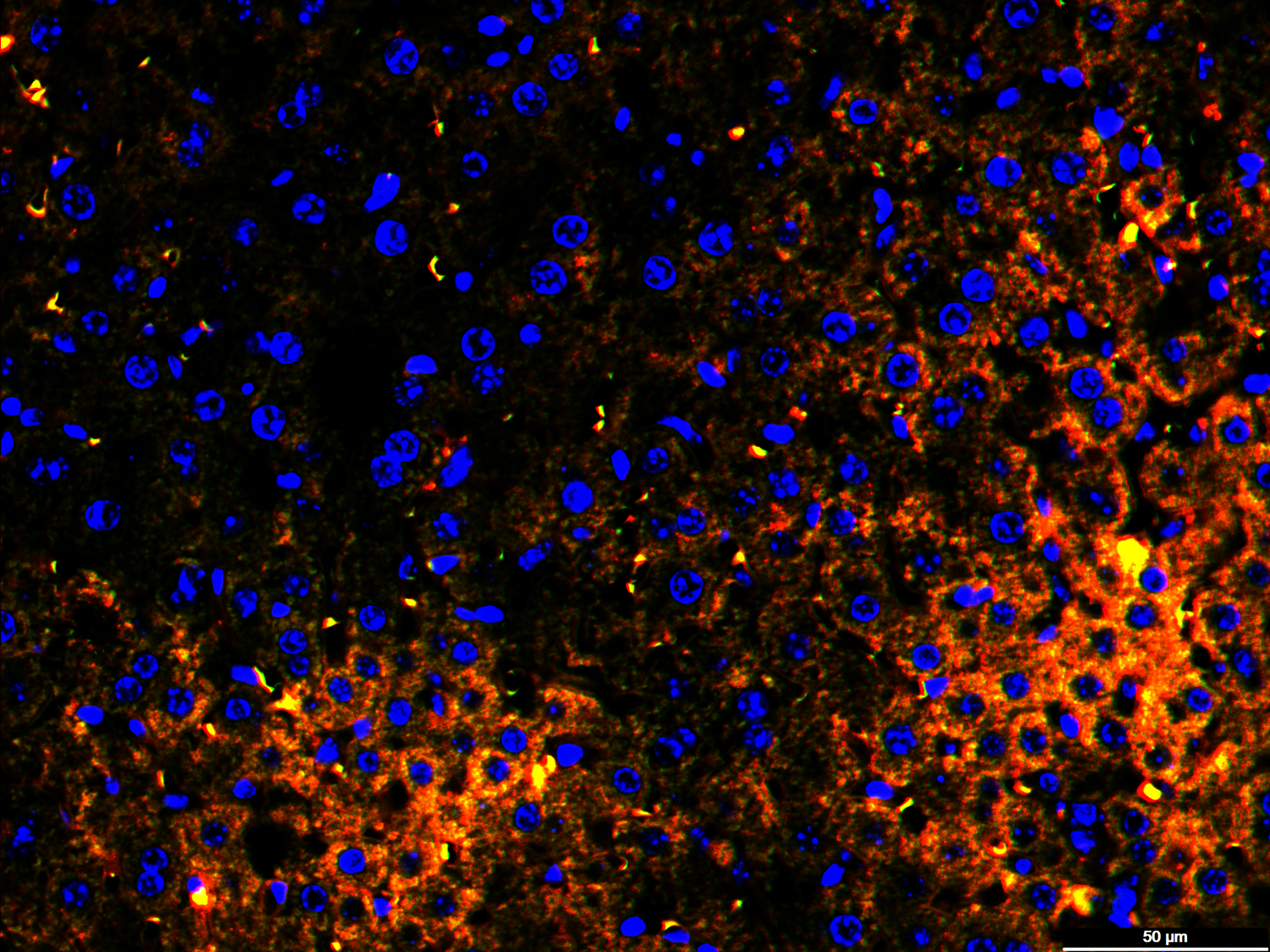

Supplement: Supplementary file 2 [file DataSheet11.ZIP › GSL.tif]

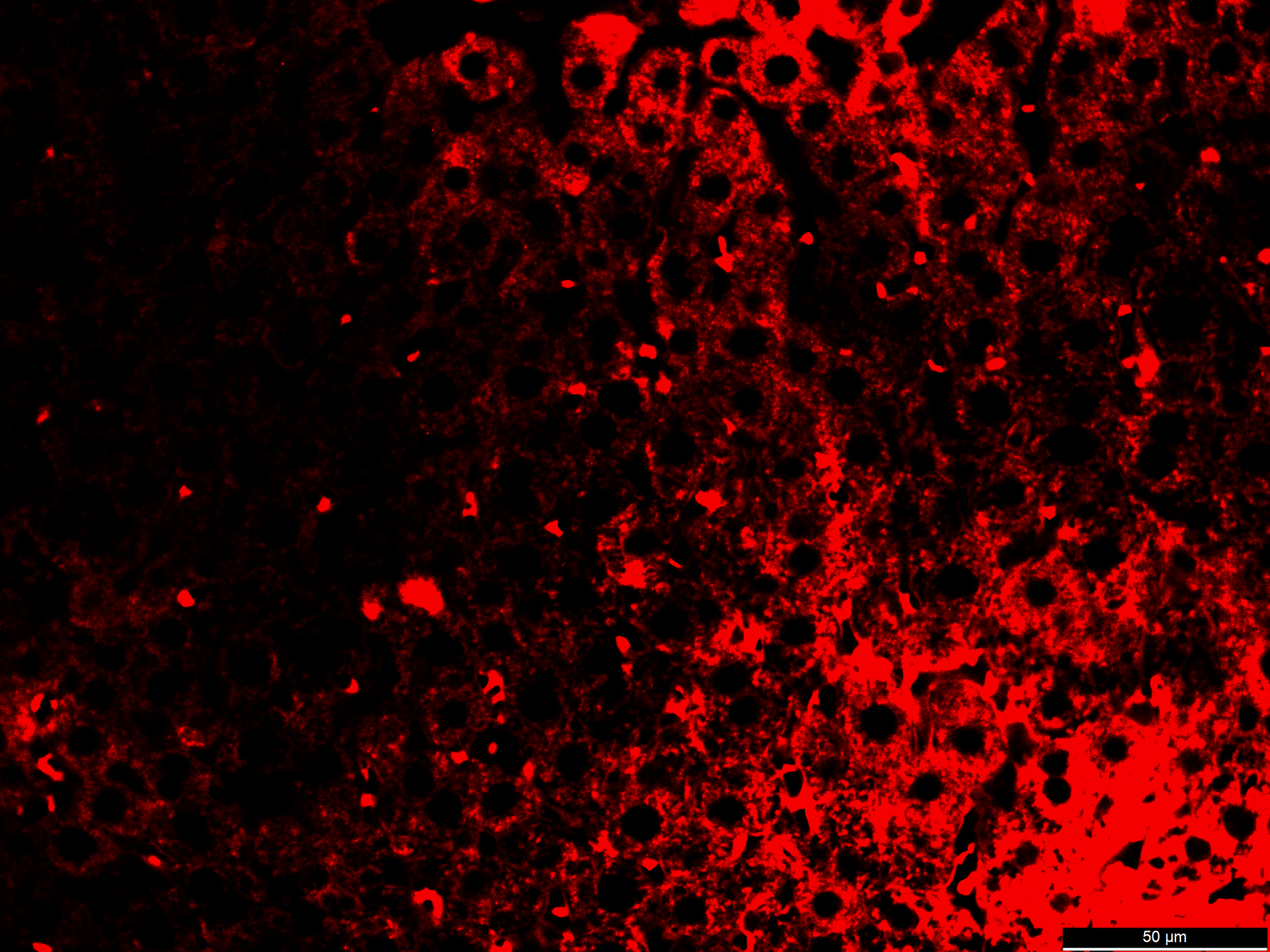

Supplement: Supplementary file 2 [file DataSheet11.ZIP › LLPS.tif]

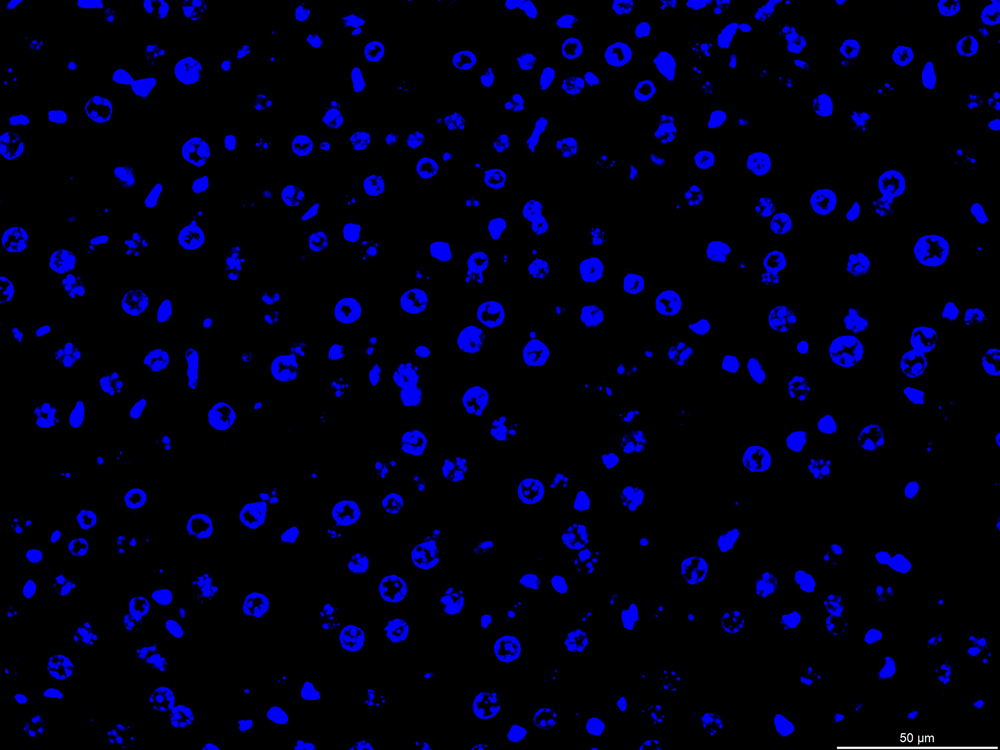

Supplement: Supplementary file 2 [file DataSheet11.ZIP › LPS.tif]

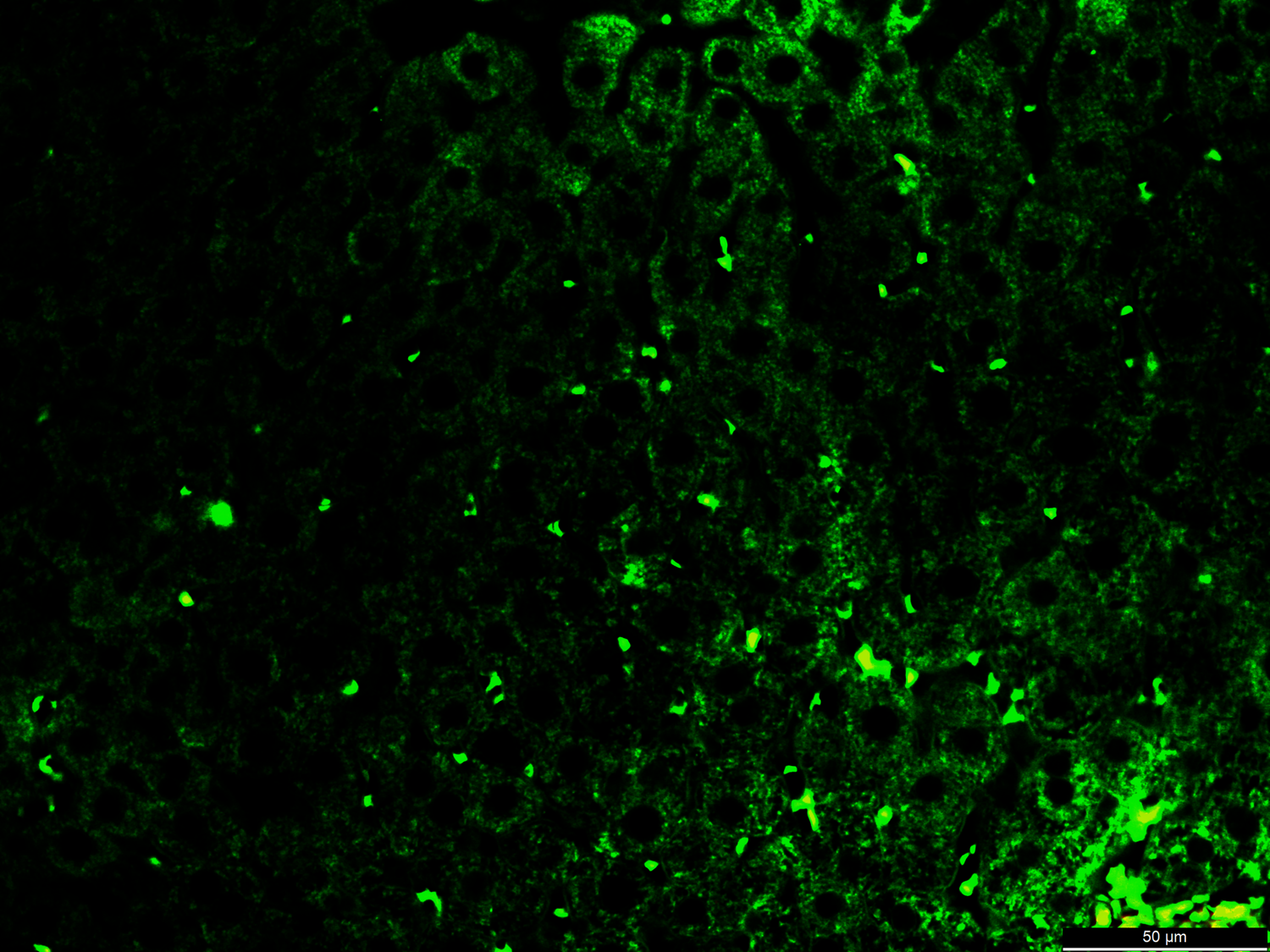

Supplement: Supplementary file 2 [file DataSheet11.ZIP › LPSS.tif]

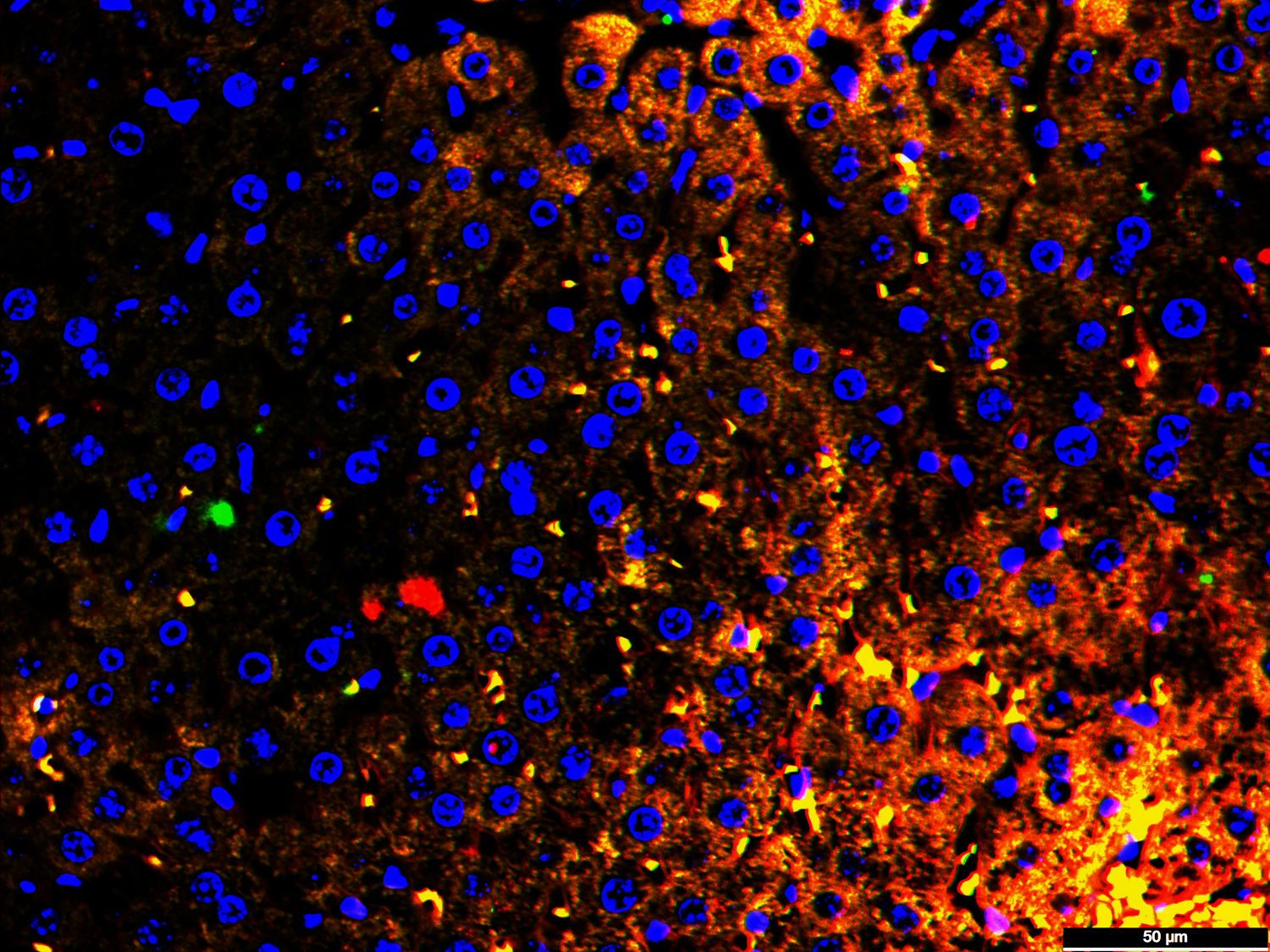

Supplement: Supplementary file 2 [file DataSheet11.ZIP › LPSSS.tif]

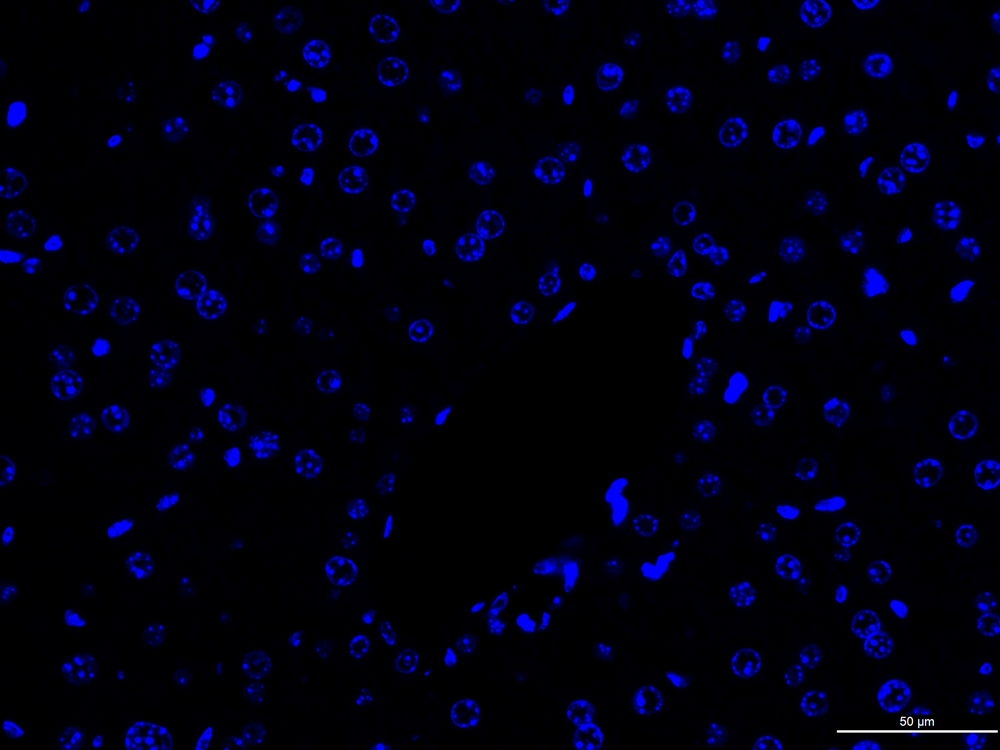

Supplement: Supplementary file 2 [file DataSheet11.ZIP › nc.tif]

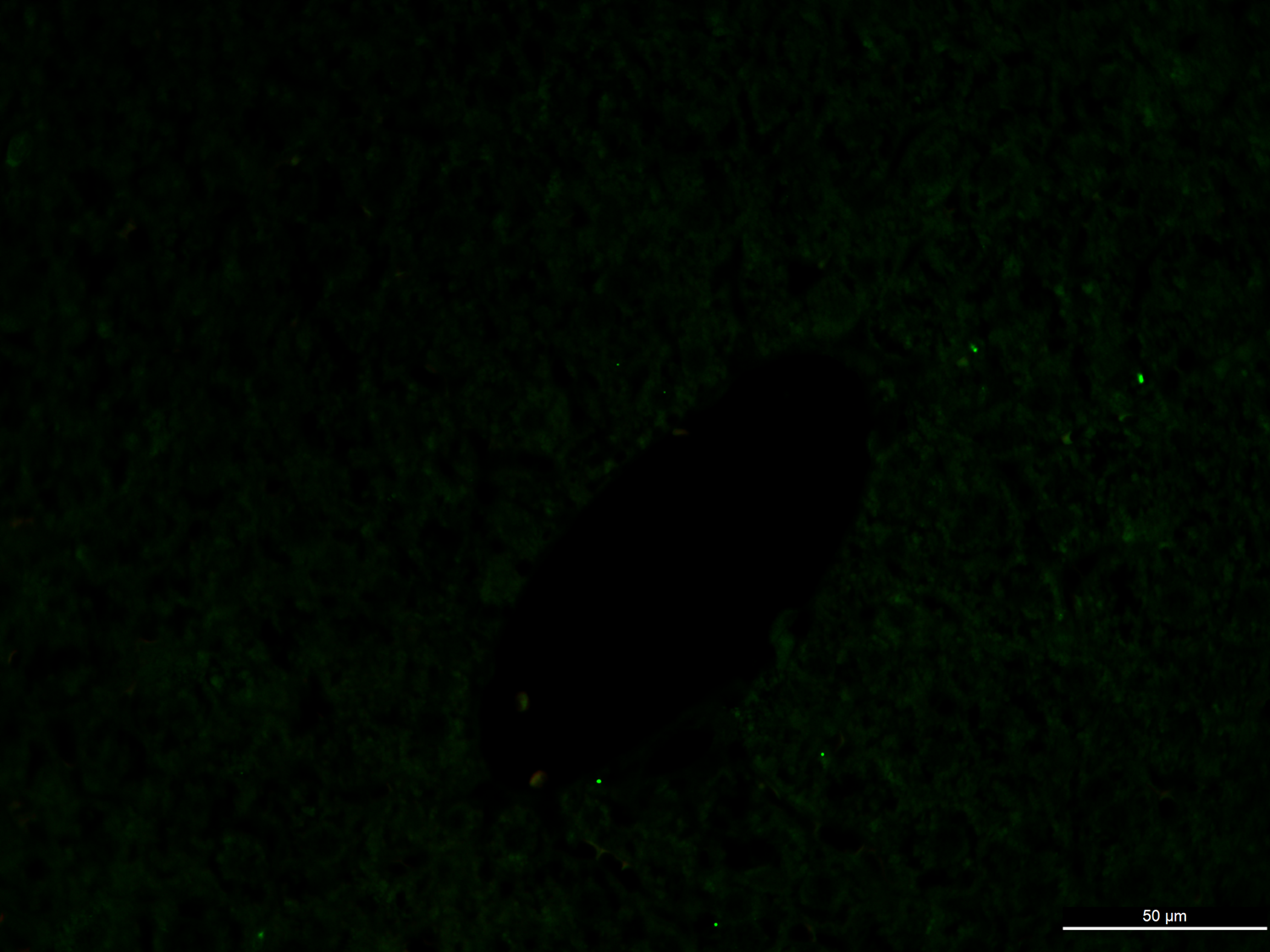

Supplement: Supplementary file 2 [file DataSheet11.ZIP › ncc.tif]

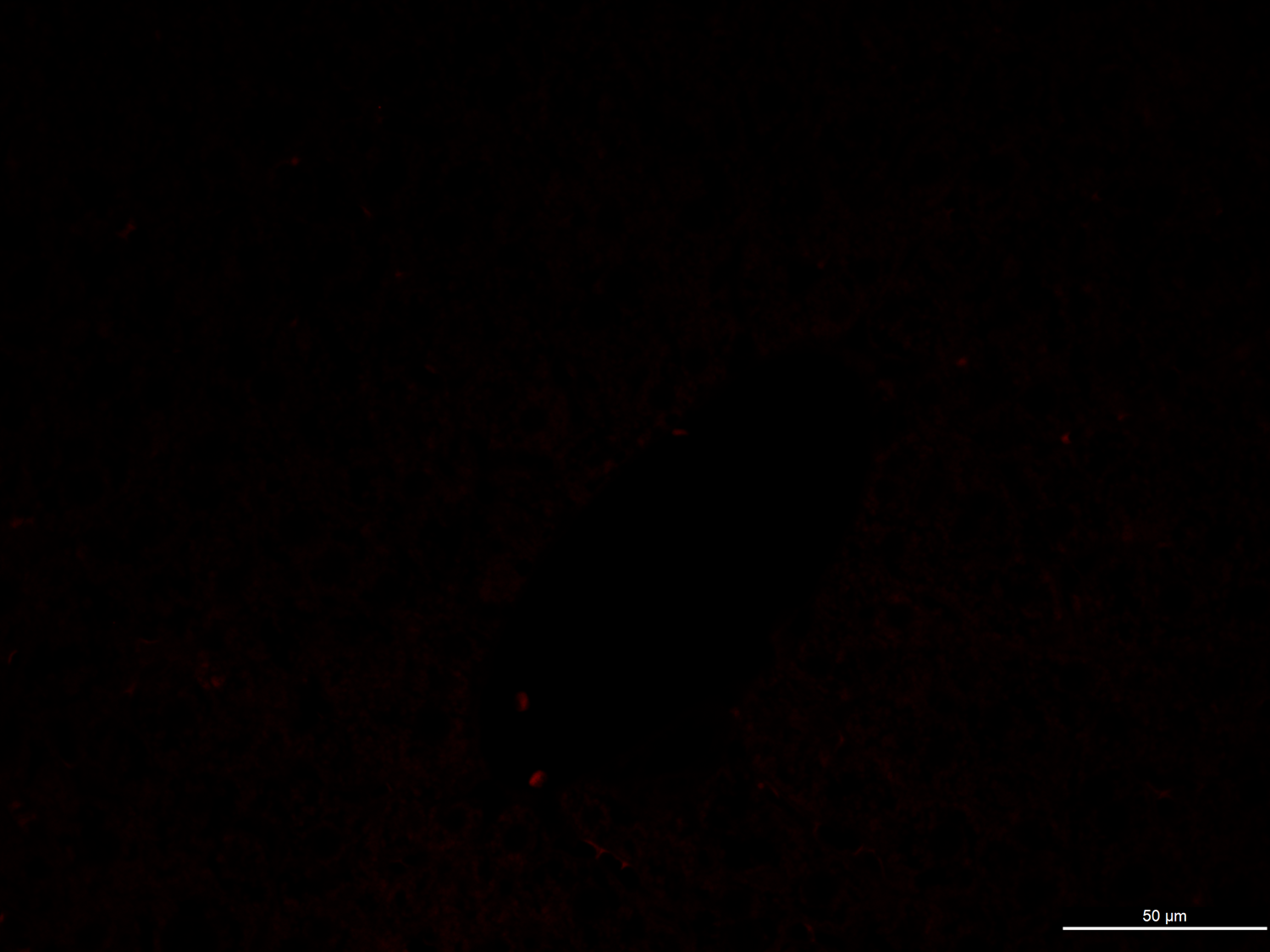

Supplement: Supplementary file 2 [file DataSheet11.ZIP › nncc.tif]

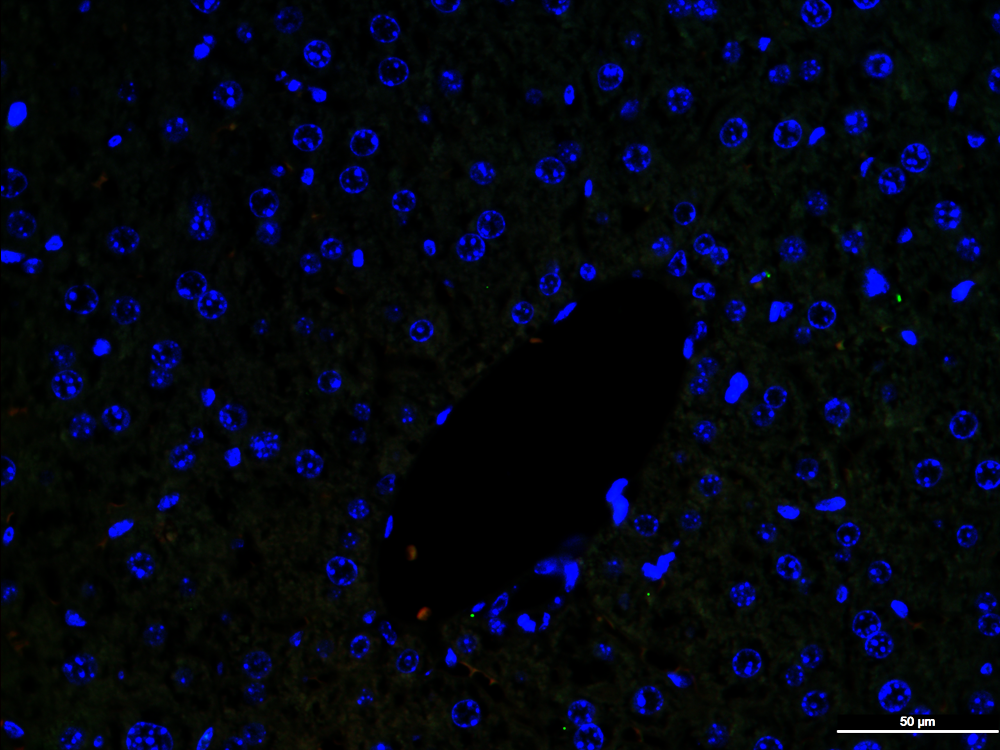

Supplement: Supplementary file 2 [file DataSheet11.ZIP › NNNC.tif]

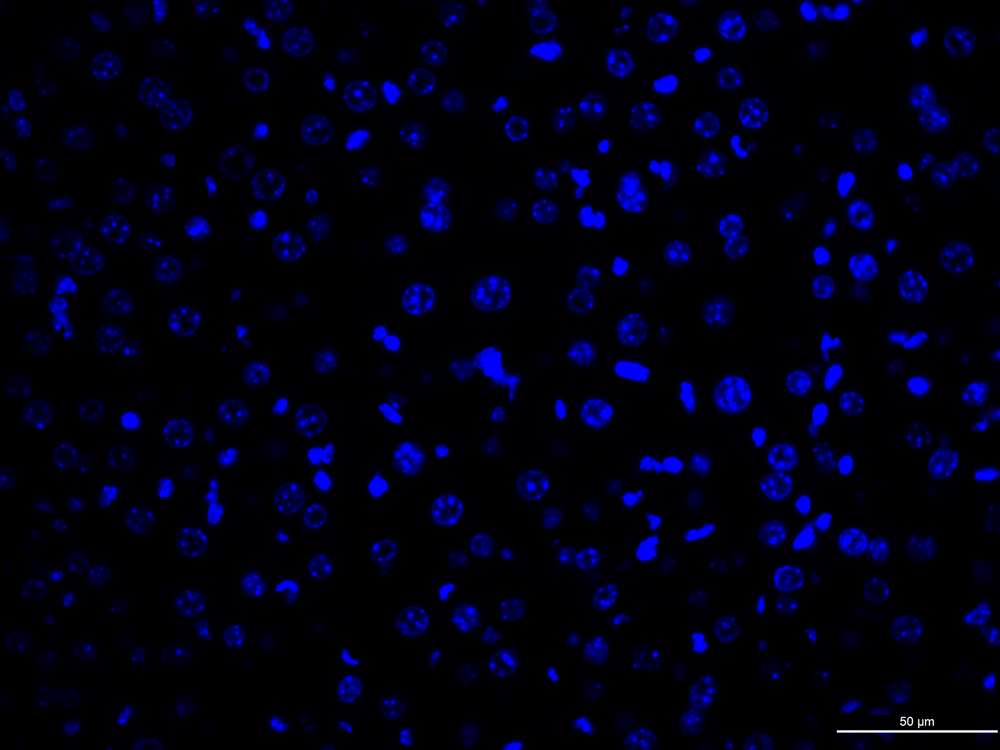

Supplement: Supplementary file 2 [file DataSheet11.ZIP › SL.tif]

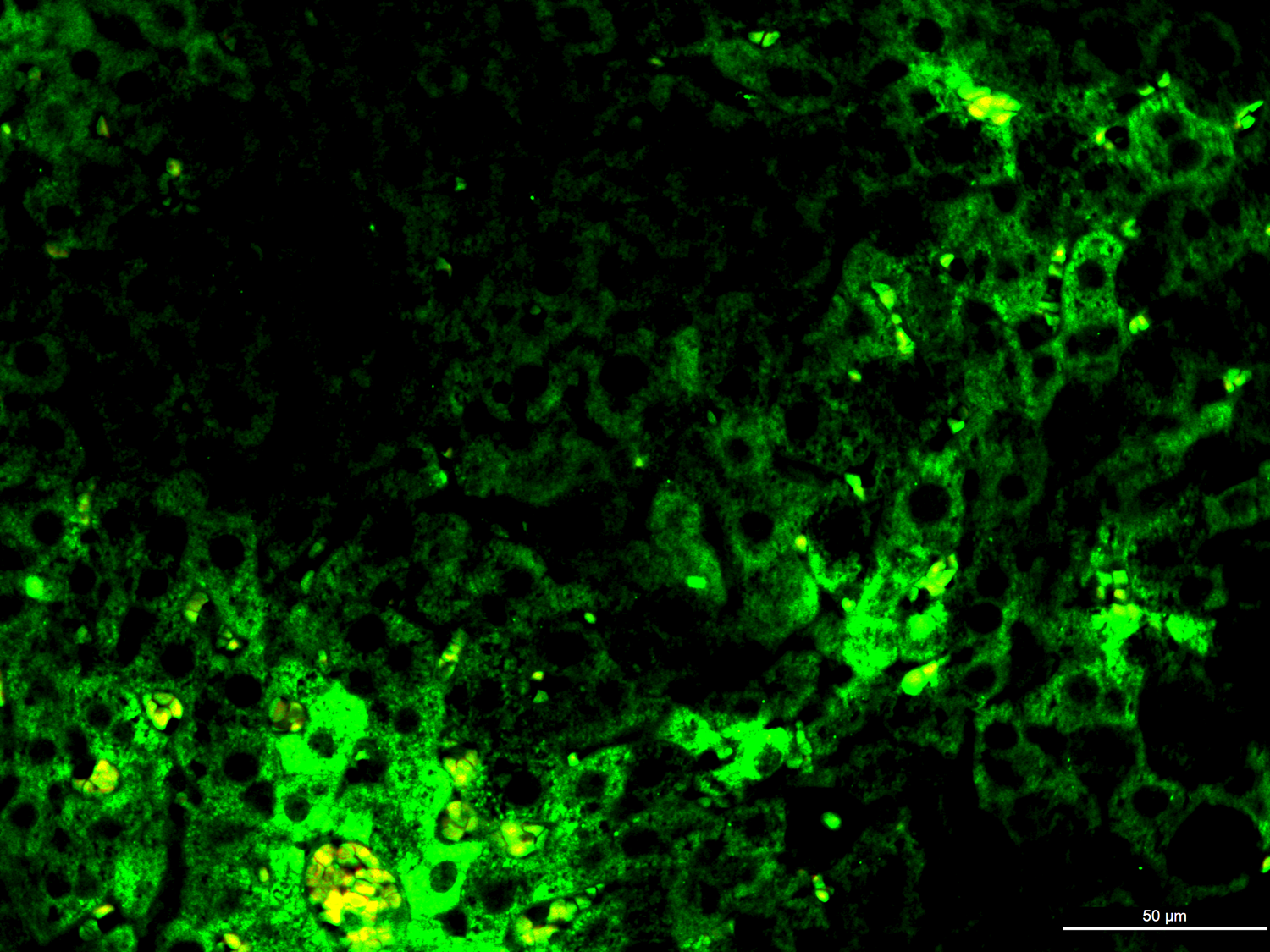

Supplement: Supplementary file 2 [file DataSheet11.ZIP › SLL (2).tif]

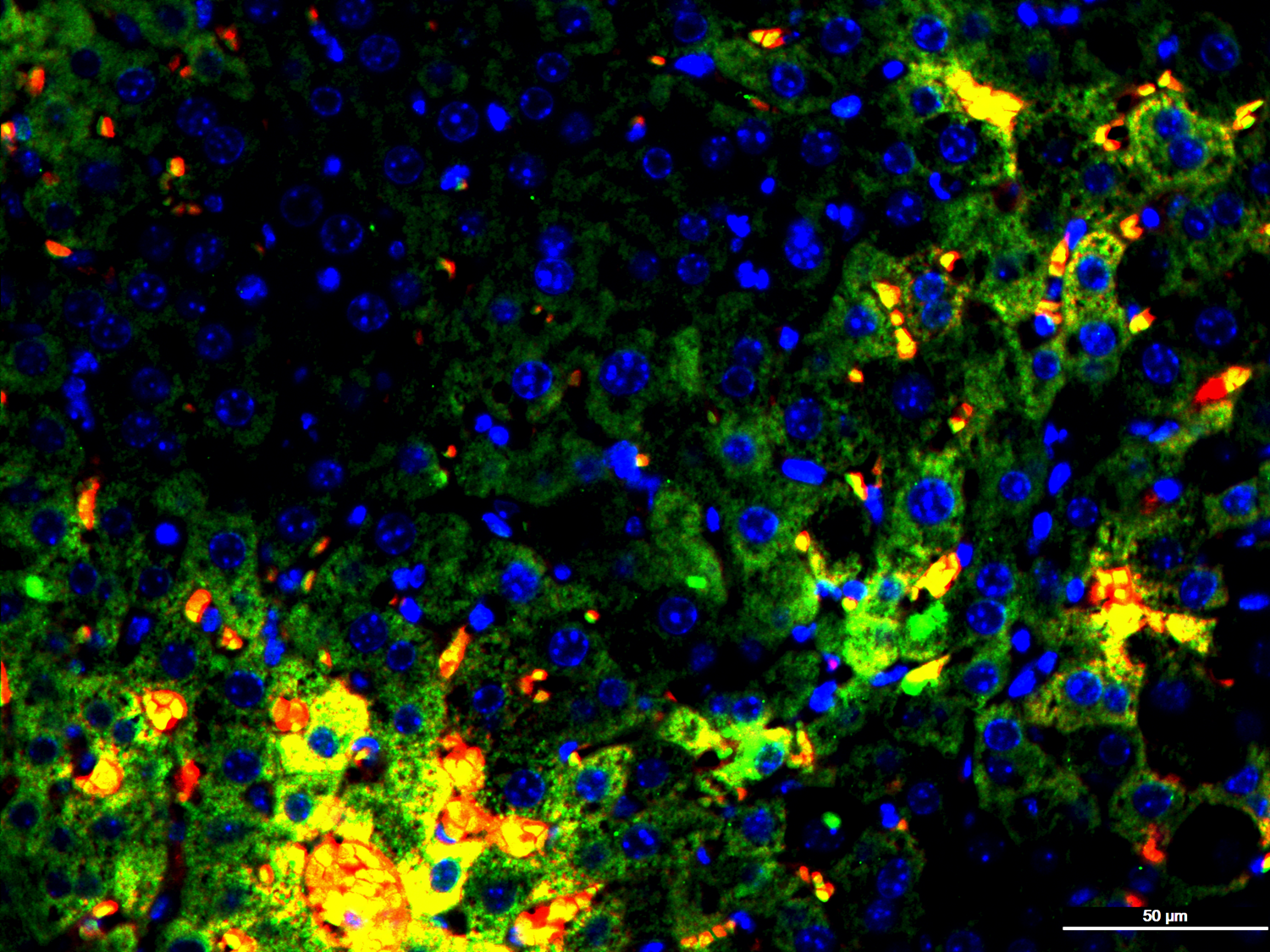

Supplement: Supplementary file 2 [file DataSheet11.ZIP › SLL.tif]

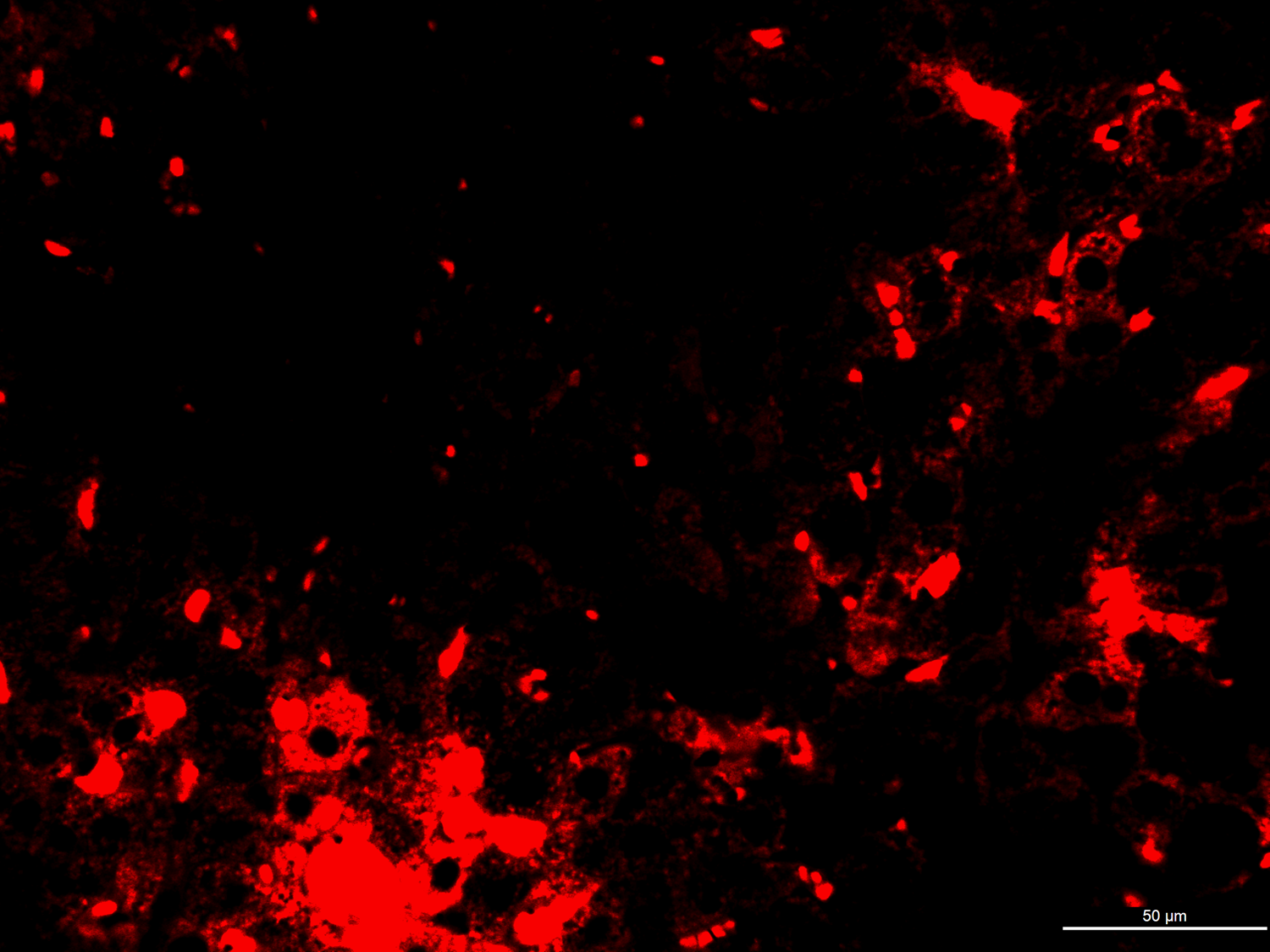

Supplement: Supplementary file 2 [file DataSheet11.ZIP › SSL.tif]

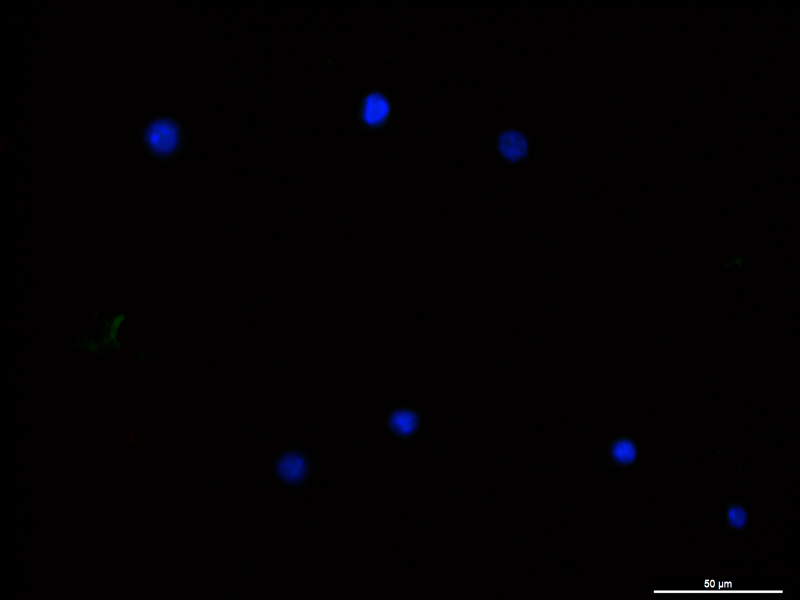

Supplement: Supplementary file 2 [file DataSheet11.ZIP › Supplementary Figure 1 Con.tif]

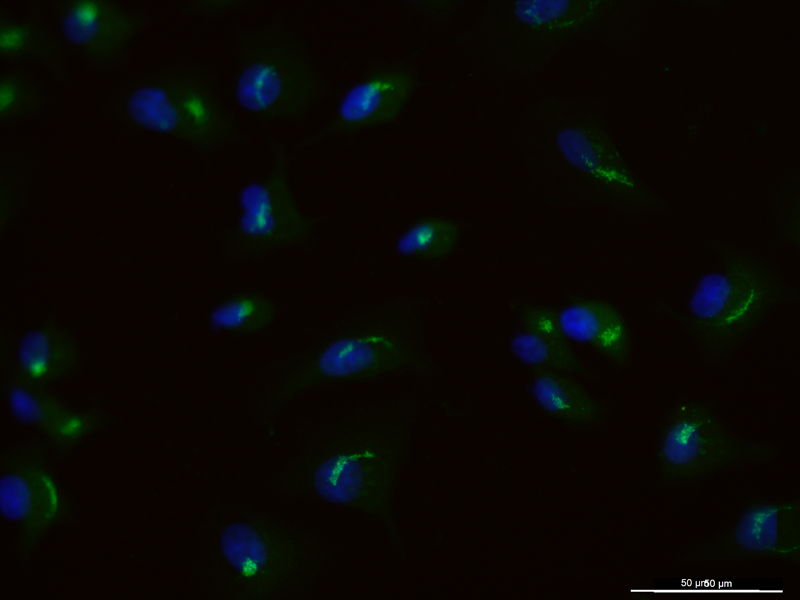

Supplement: Supplementary file 2 [file DataSheet11.ZIP › Supplementary Figure 1 LPS.tif]

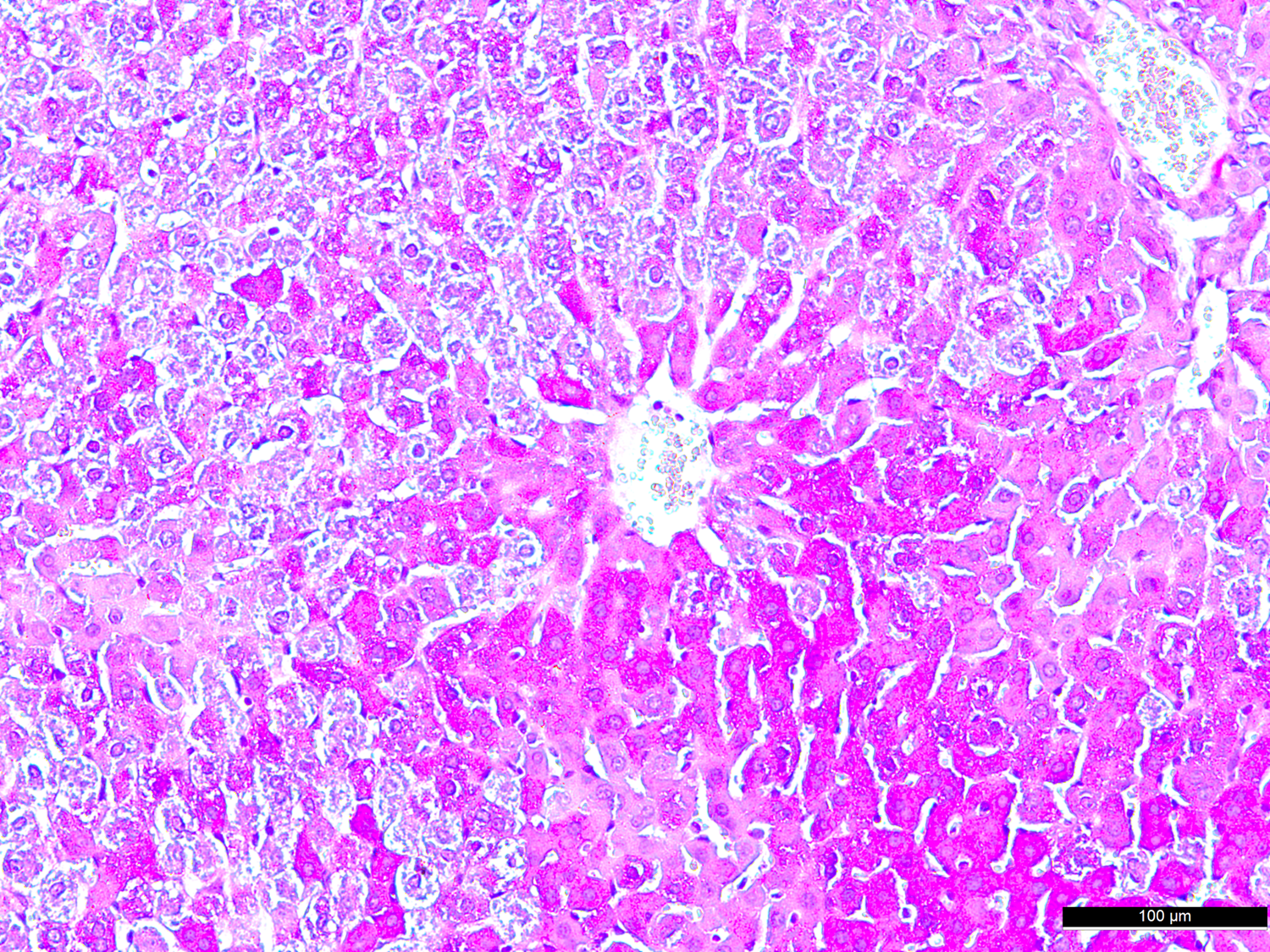

Supplement: Supplementary file 4 [file DataSheet9.ZIP › 100um PAS/LPS (2).tif]

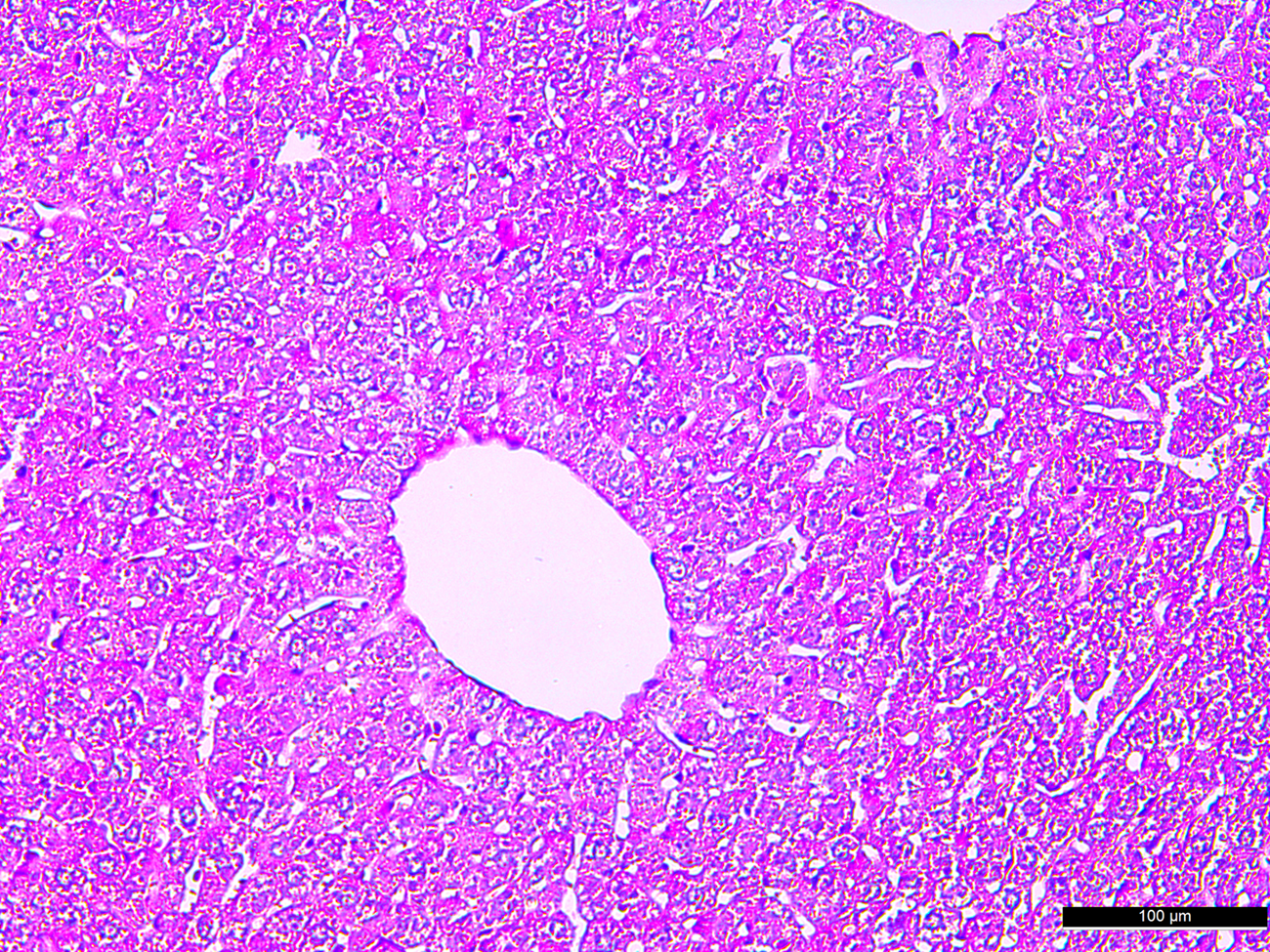

Supplement: Supplementary file 4 [file DataSheet9.ZIP › 100um PAS/NC.tif]

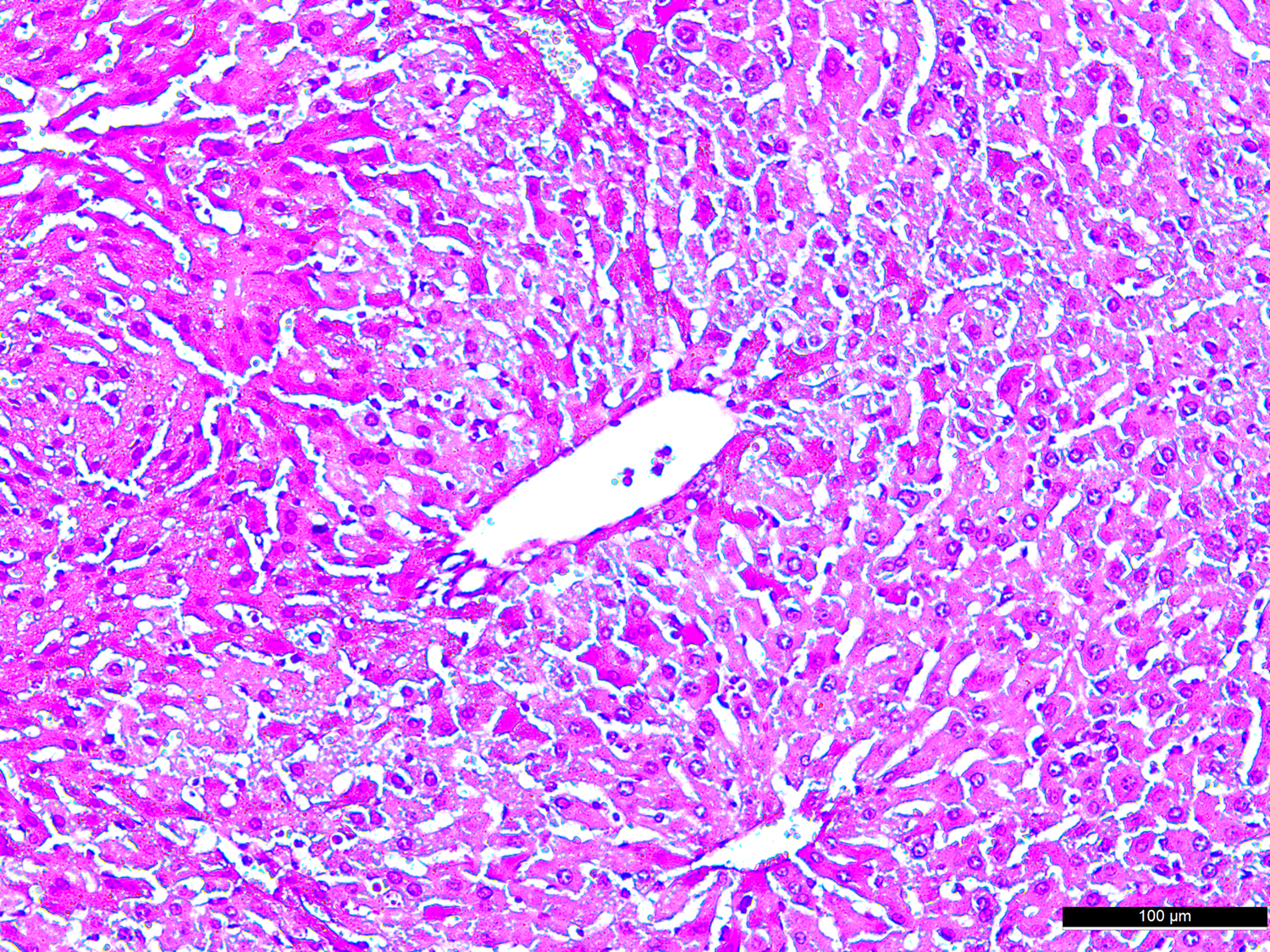

Supplement: Supplementary file 4 [file DataSheet9.ZIP › 100um PAS/ST+LPS.tif]

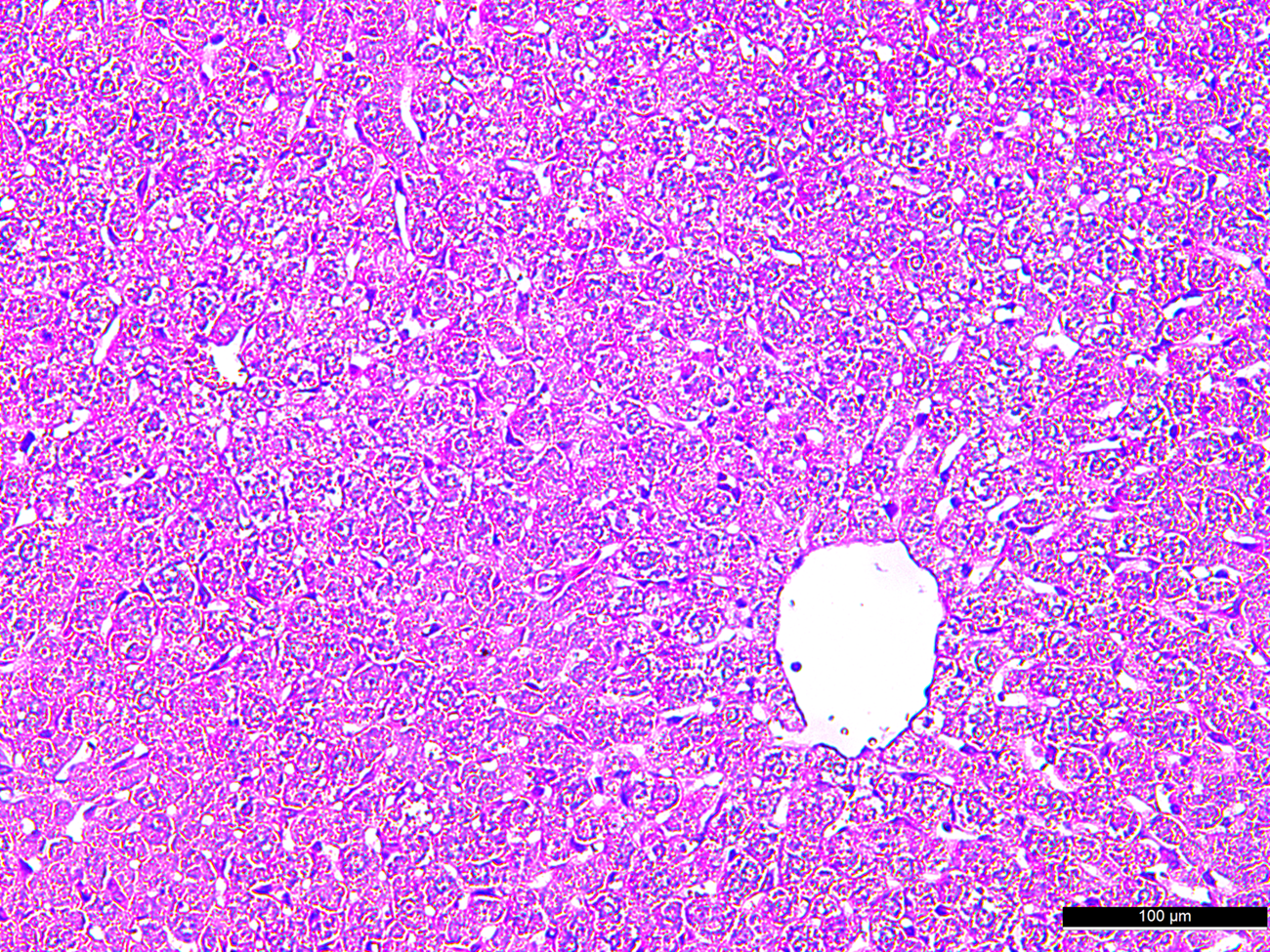

Supplement: Supplementary file 4 [file DataSheet9.ZIP › 100um PAS/ST.tif]

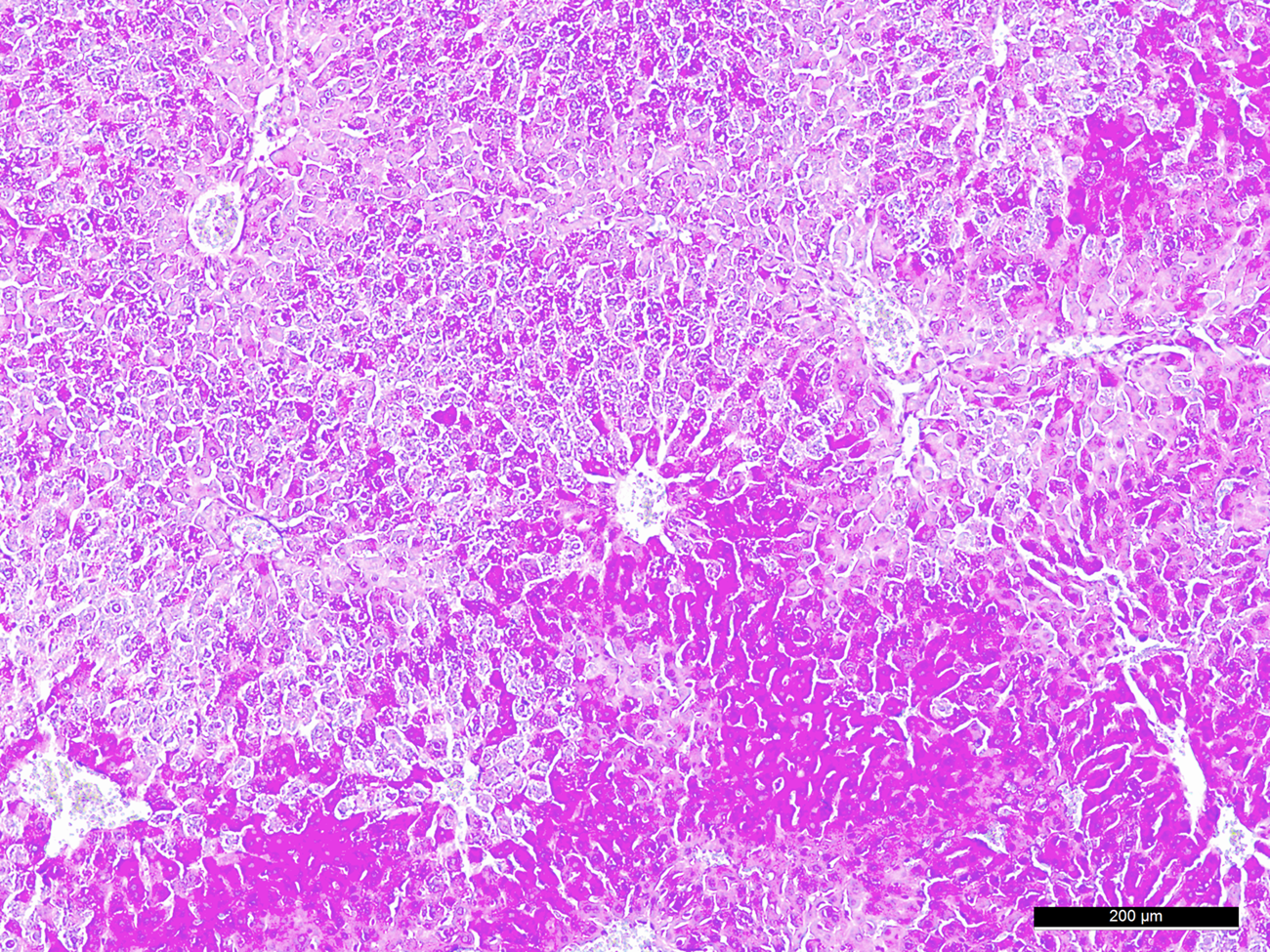

Supplement: Supplementary file 4 [file DataSheet9.ZIP › 200um PAS/LPS.tif]

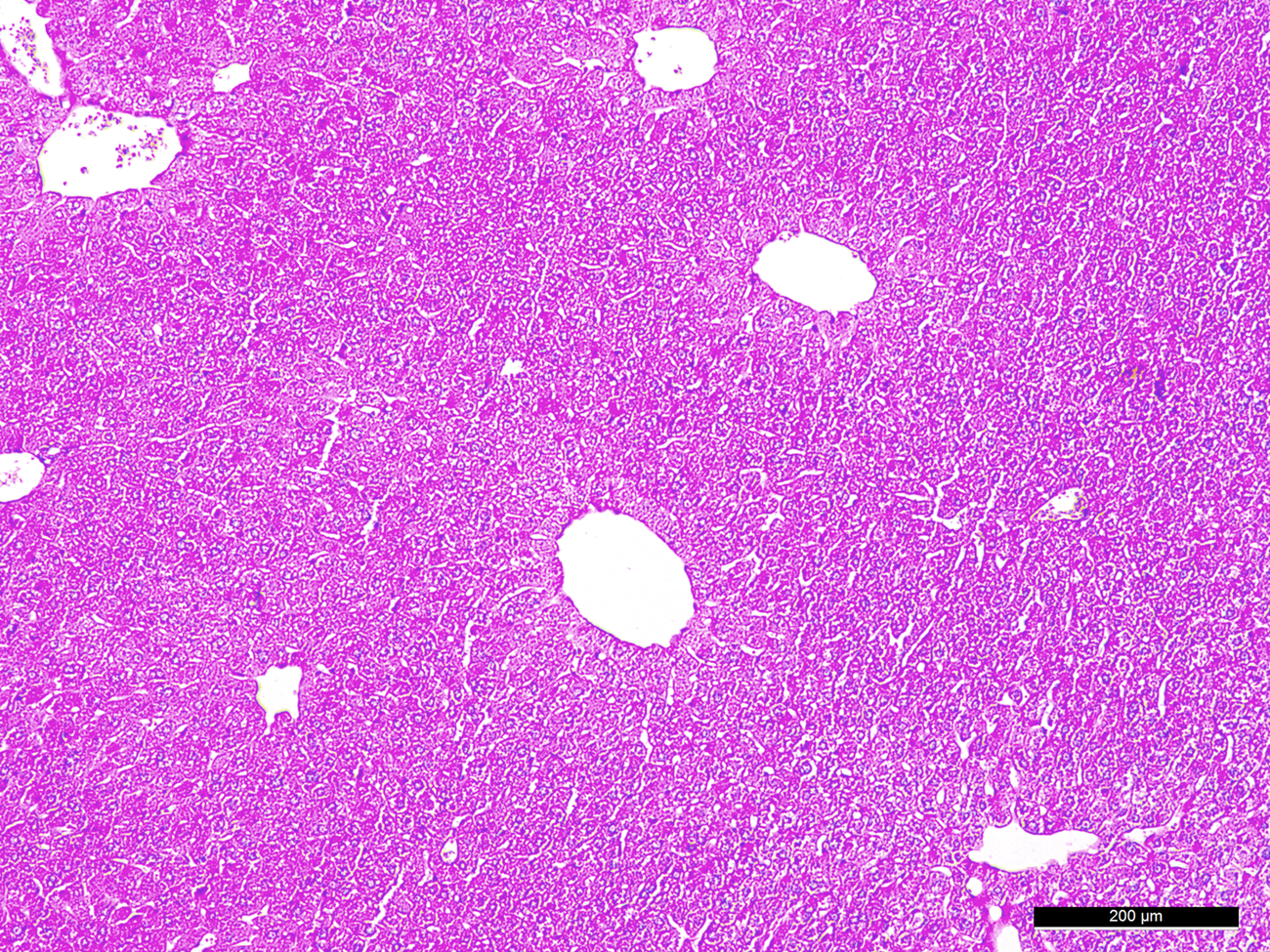

Supplement: Supplementary file 4 [file DataSheet9.ZIP › 200um PAS/NC (2).tif]

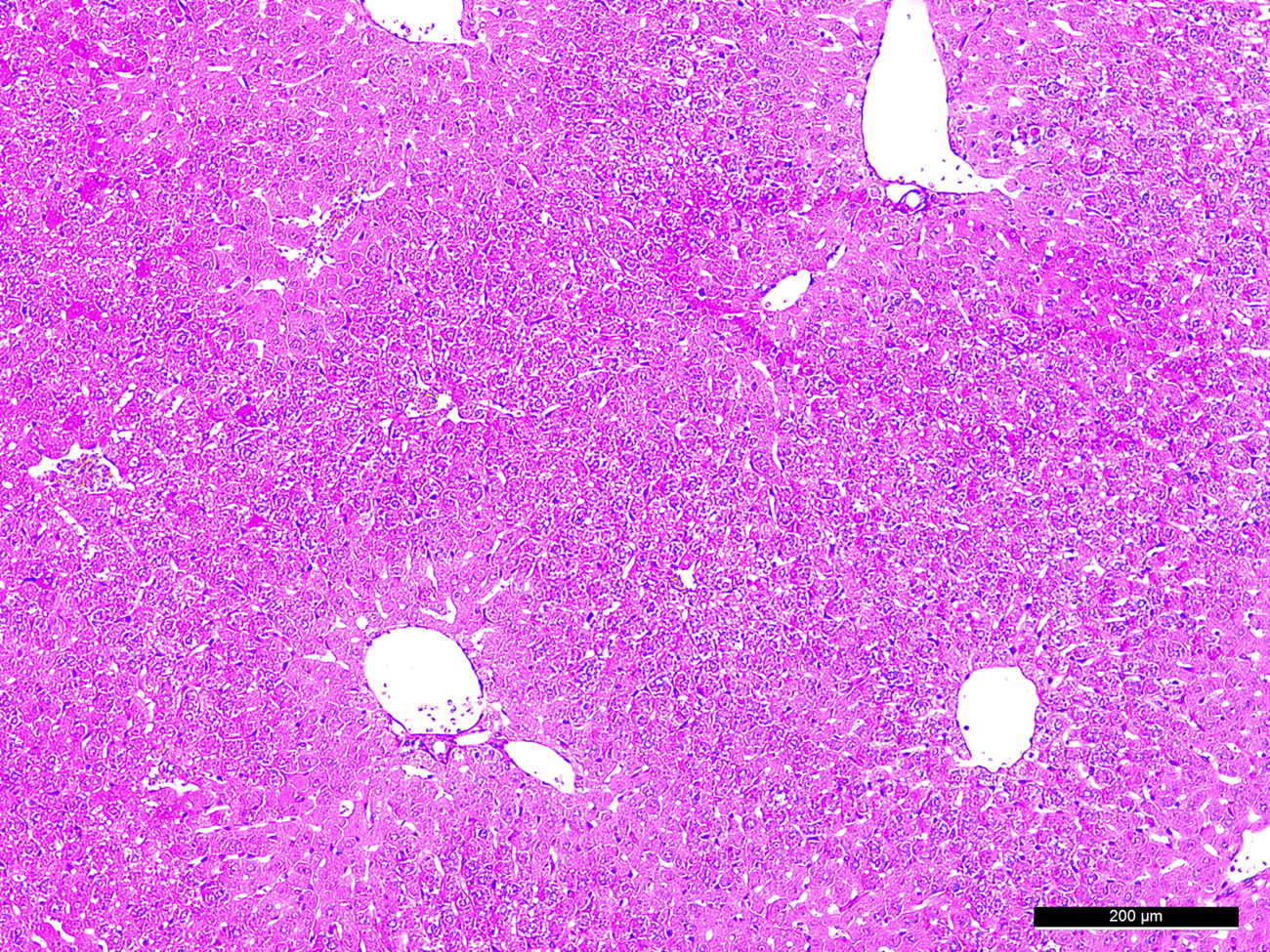

Supplement: Supplementary file 4 [file DataSheet9.ZIP › 200um PAS/ST (2).tif]

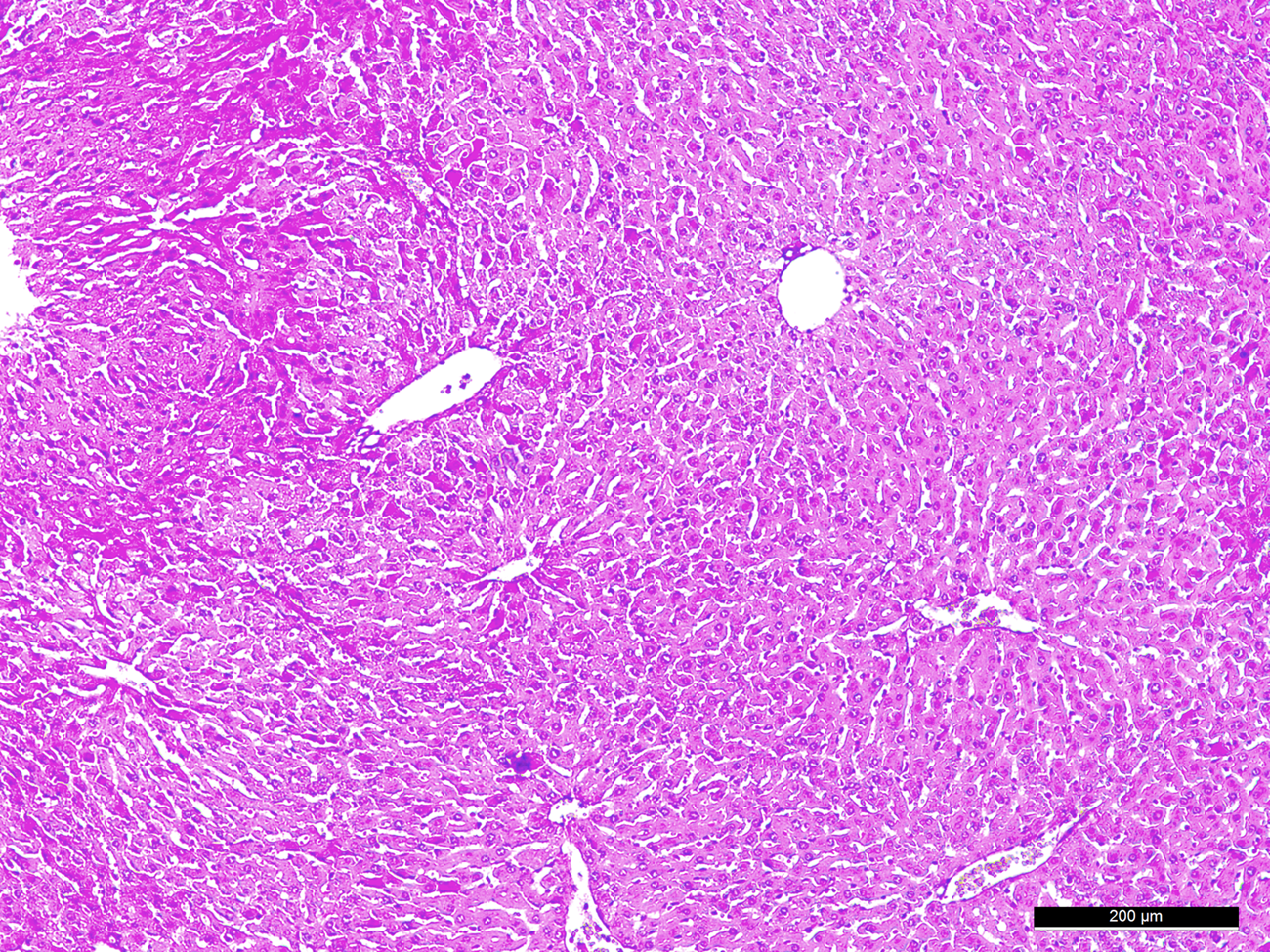

Supplement: Supplementary file 4 [file DataSheet9.ZIP › 200um PAS/ST+LPS (2).tif]

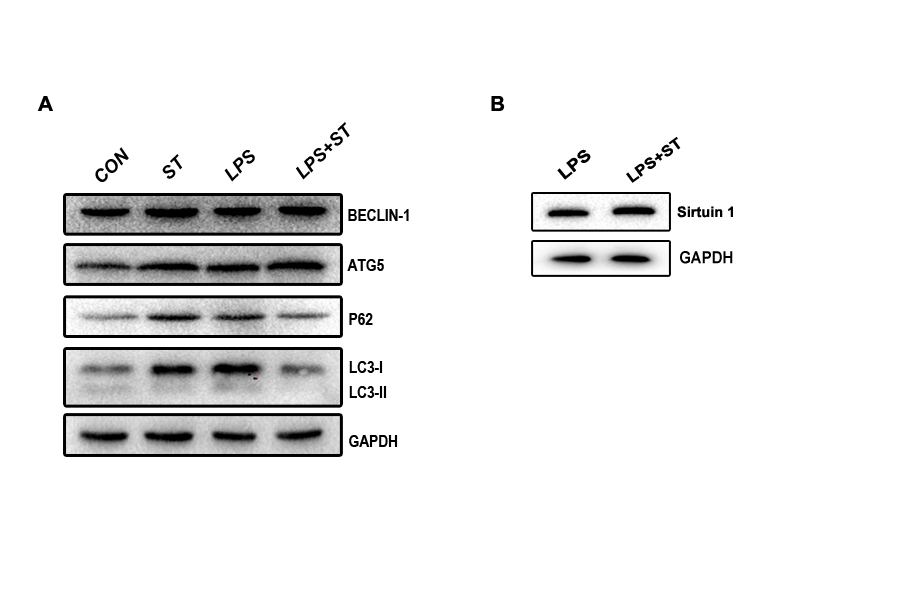

Supplement: Supplementary file 5 [file Image3.TIF]

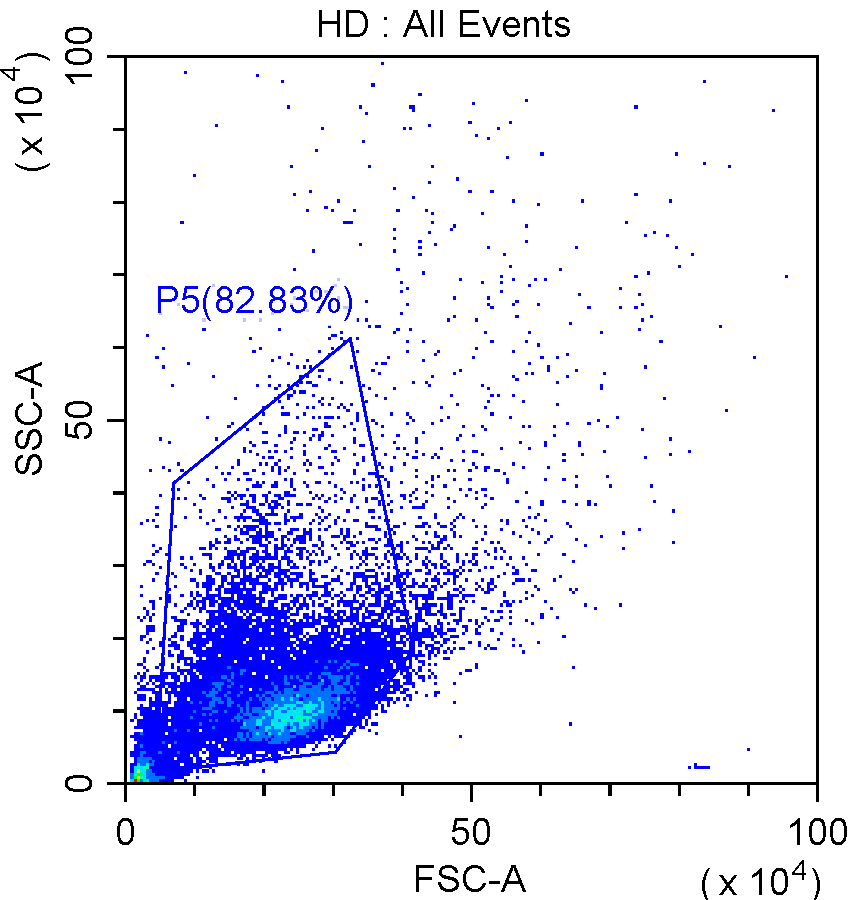

Supplement: Supplementary file 6 [file DataSheet4.ZIP › A/2-10G+LPS/HD _Plot1.bmp]

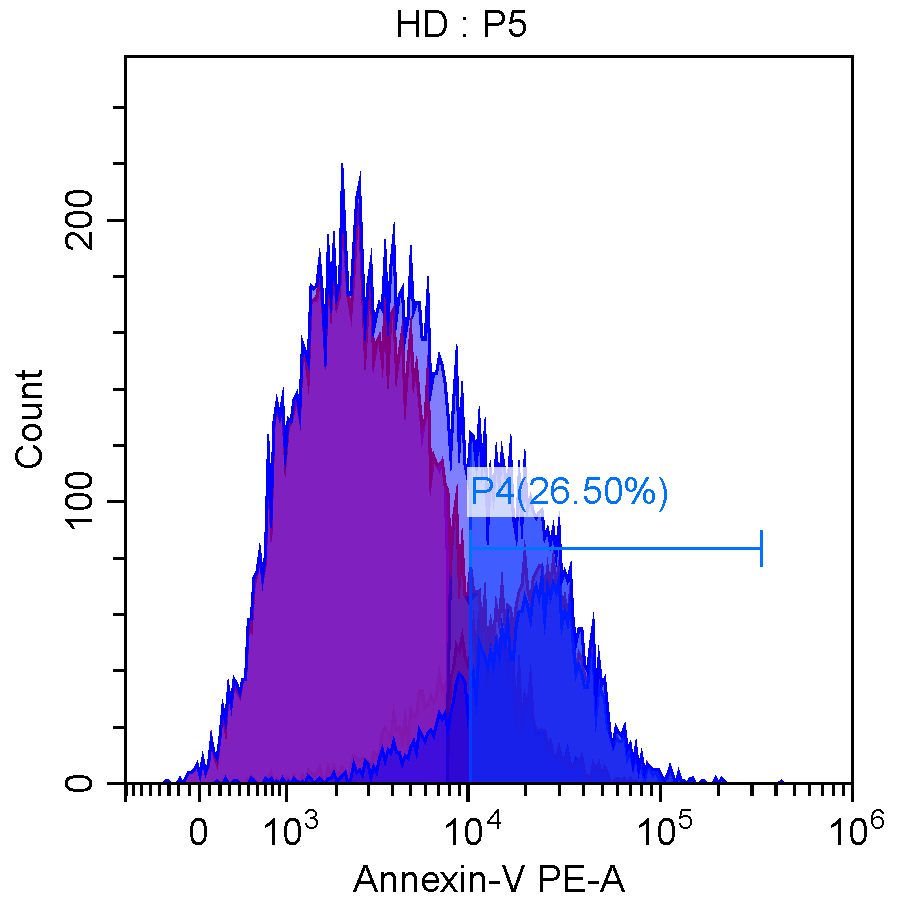

Supplement: Supplementary file 6 [file DataSheet4.ZIP › A/2-10G+LPS/HD_ Plot.bmp]

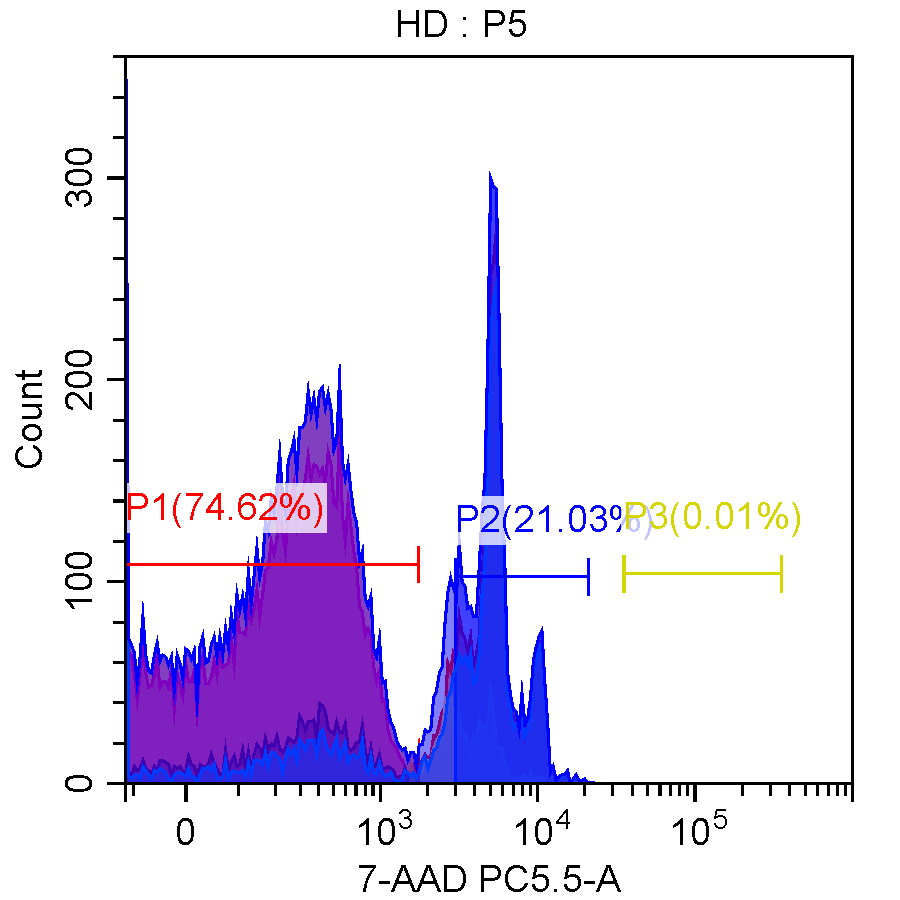

Supplement: Supplementary file 6 [file DataSheet4.ZIP › A/2-10G+LPS/HD_Plot.bmp]

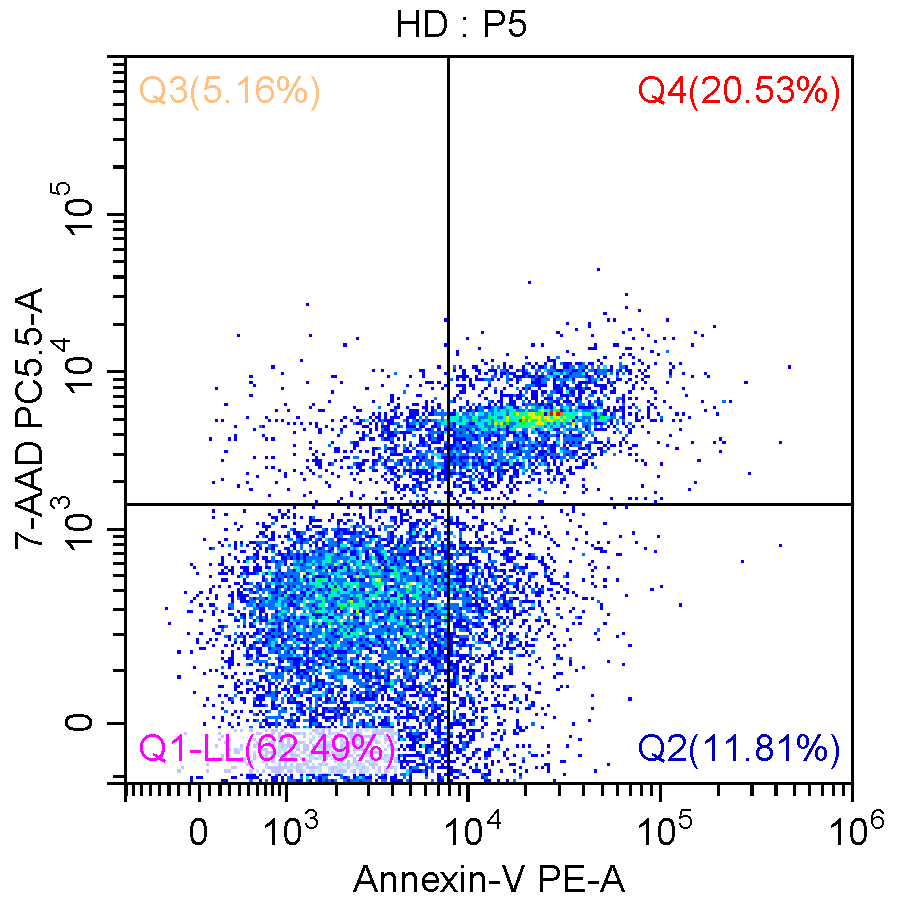

Supplement: Supplementary file 6 [file DataSheet4.ZIP › A/2-10G+LPS/HD_Plot1.bmp]

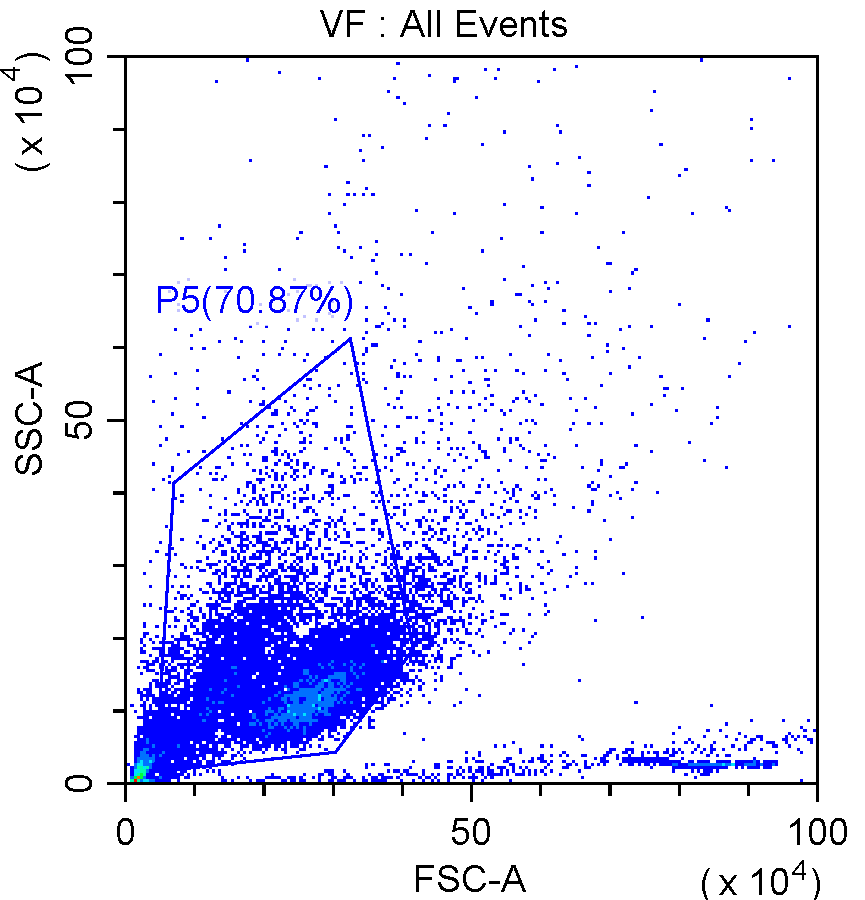

Supplement: Supplementary file 6 [file DataSheet4.ZIP › A/3-11A+LPS/VF_ Plot.bmp]

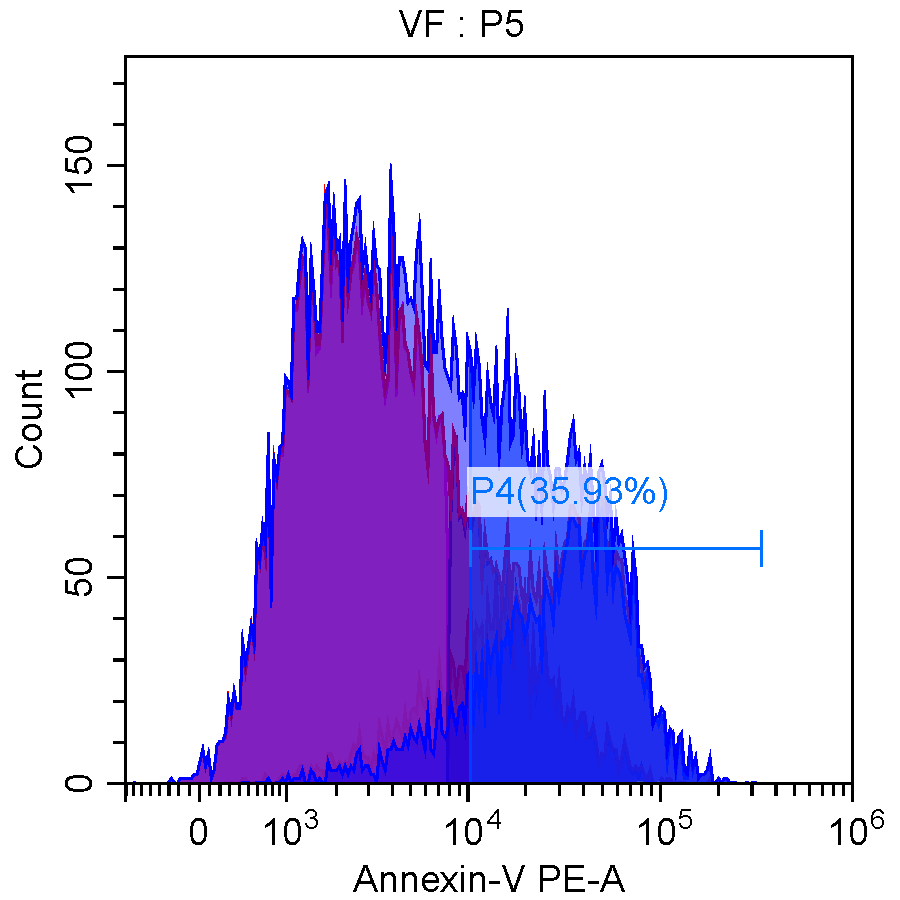

Supplement: Supplementary file 6 [file DataSheet4.ZIP › A/3-11A+LPS/VF_ Plot1.bmp]

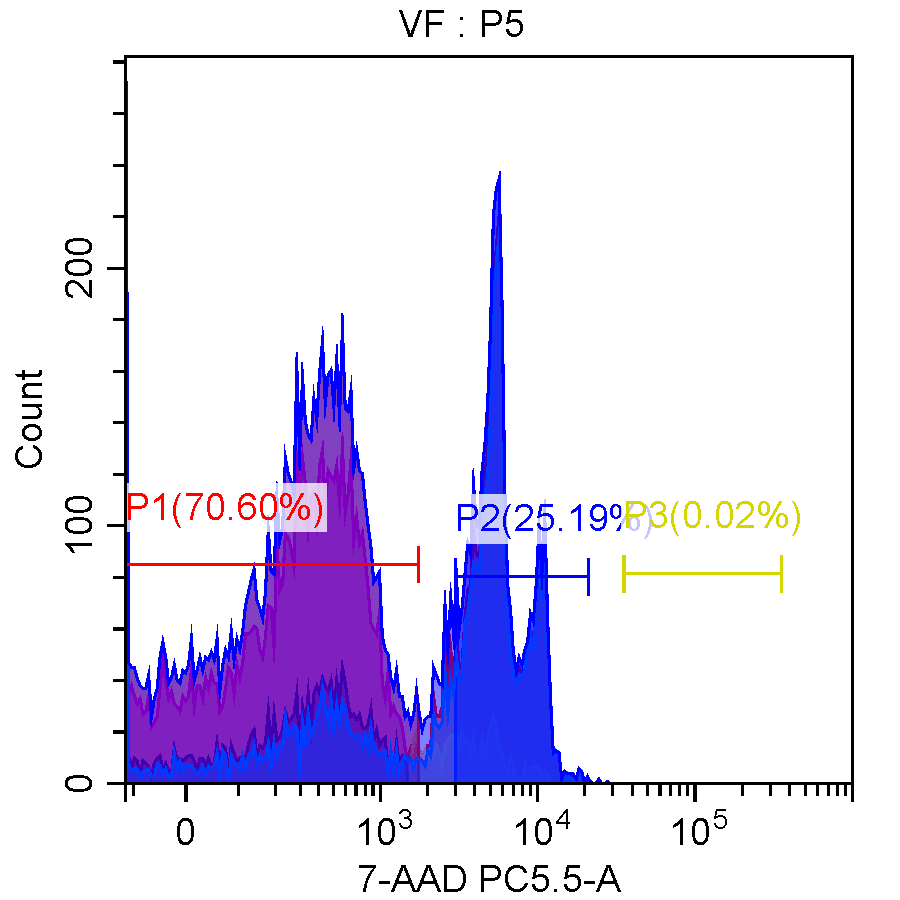

Supplement: Supplementary file 6 [file DataSheet4.ZIP › A/3-11A+LPS/VF_Plot.bmp]

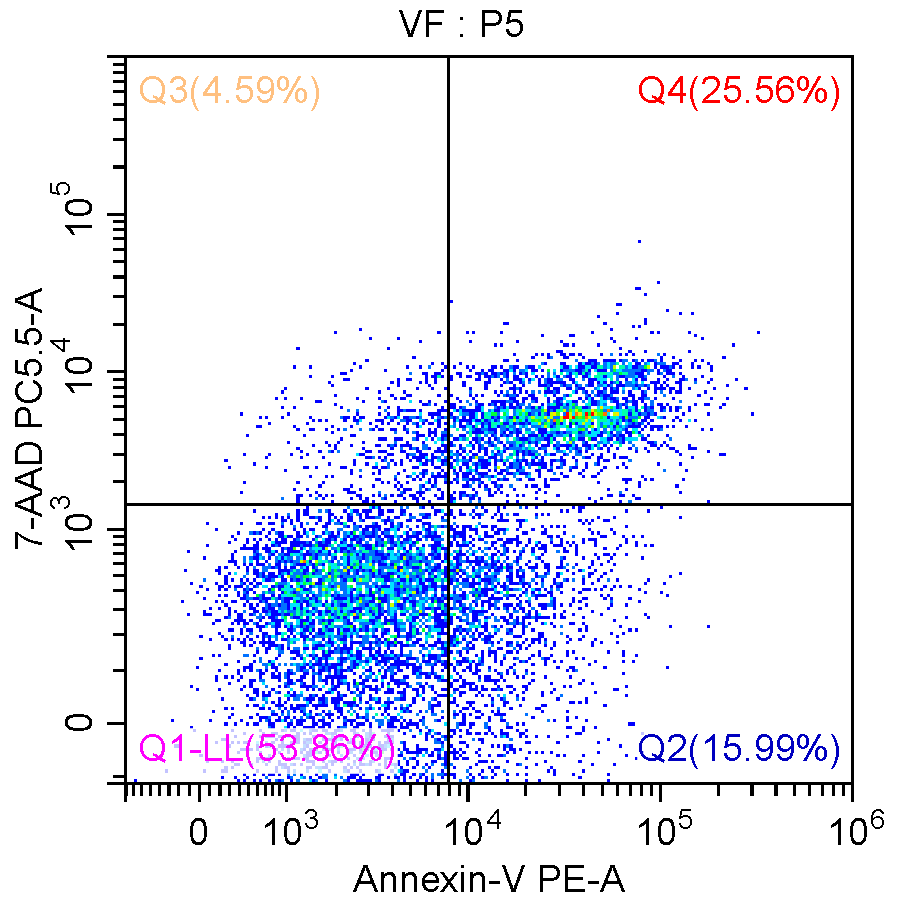

Supplement: Supplementary file 6 [file DataSheet4.ZIP › A/3-11A+LPS/VF_Plot1.bmp]

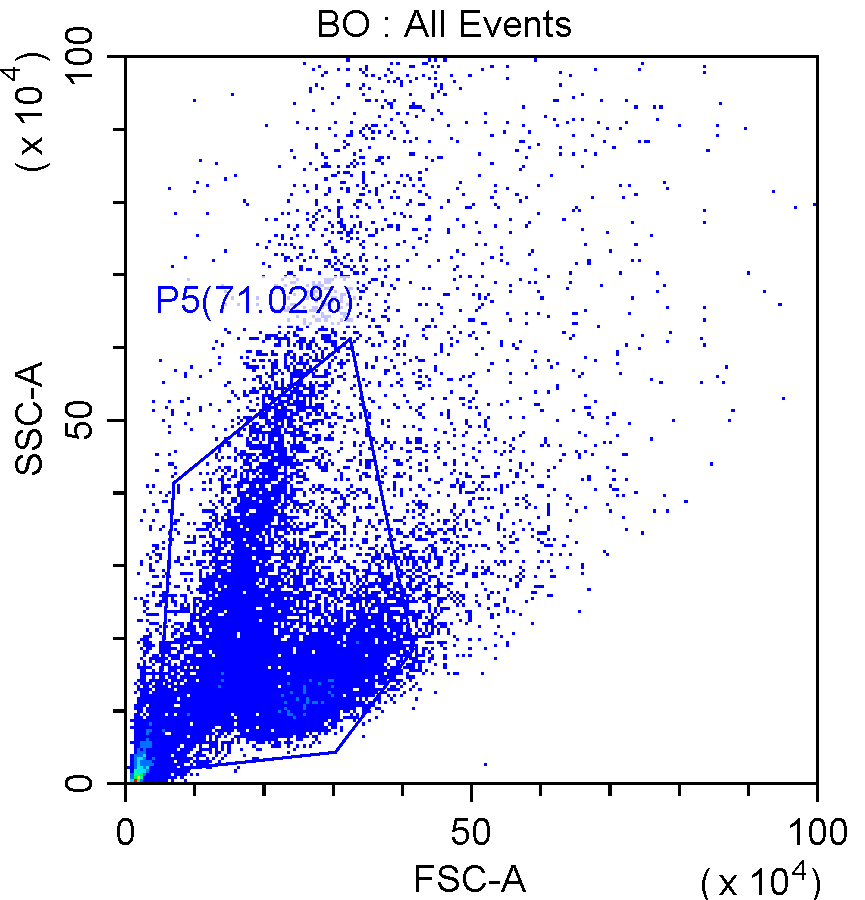

Supplement: Supplementary file 6 [file DataSheet4.ZIP › A/3-5C+LPS/BO _ Plot1.bmp]

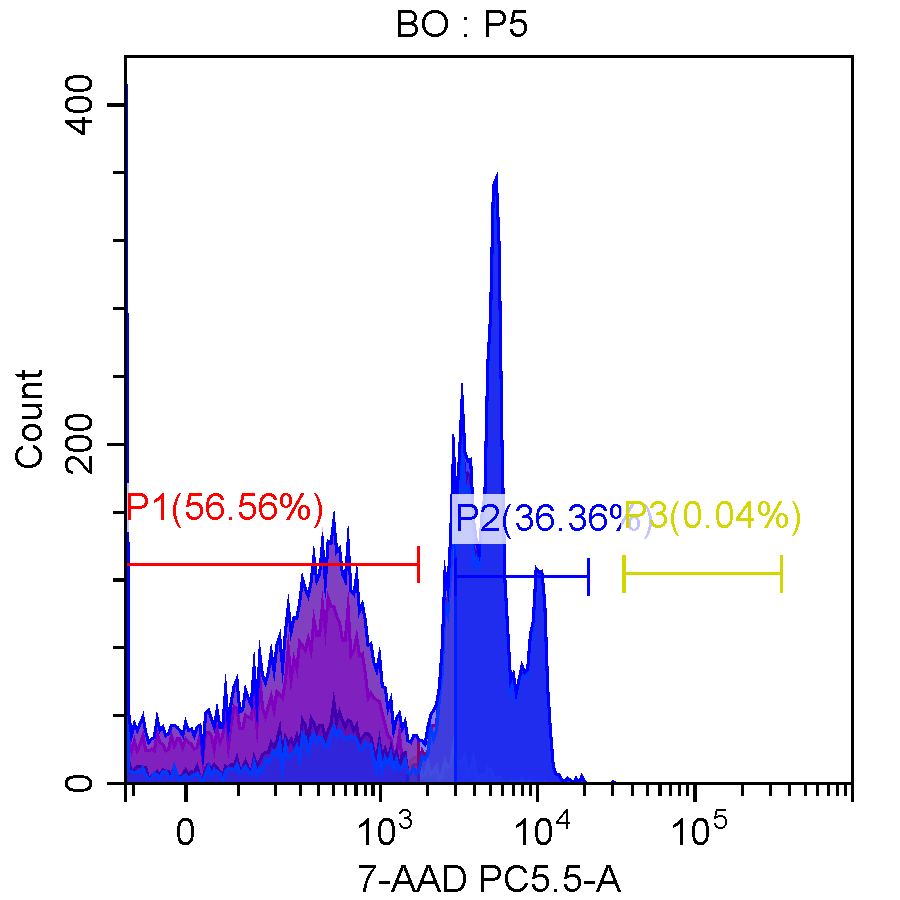

Supplement: Supplementary file 6 [file DataSheet4.ZIP › A/3-5C+LPS/BO _Plot1.bmp]

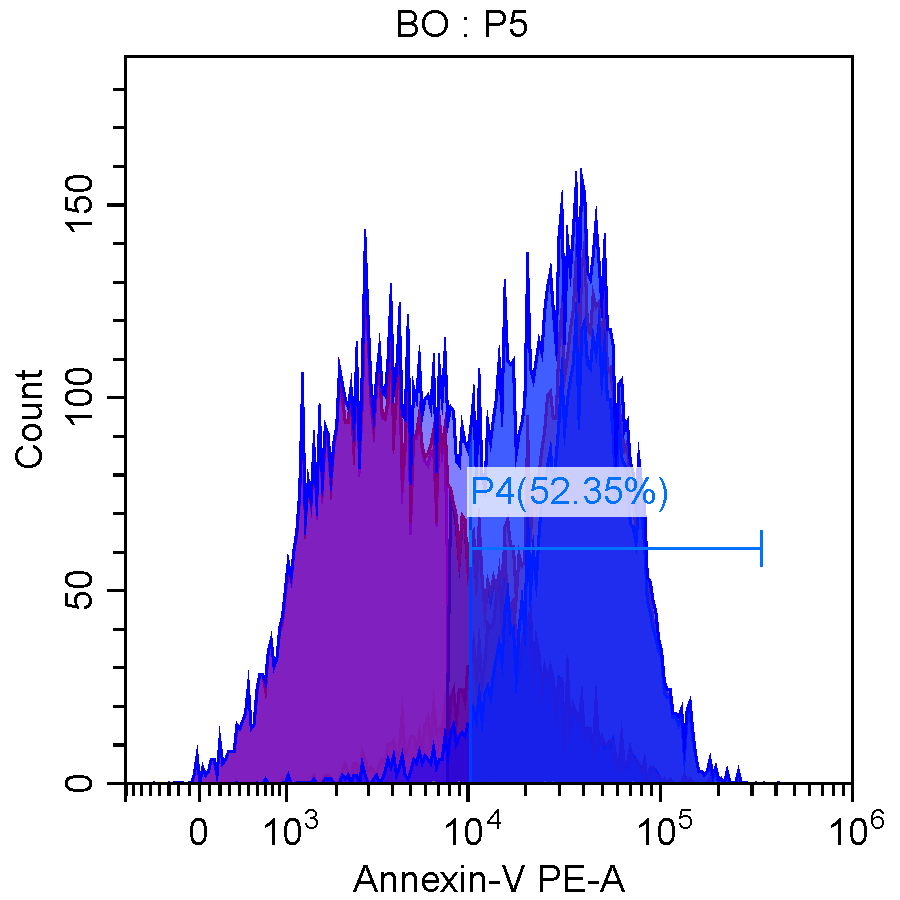

Supplement: Supplementary file 6 [file DataSheet4.ZIP › A/3-5C+LPS/BO_Plot.bmp]

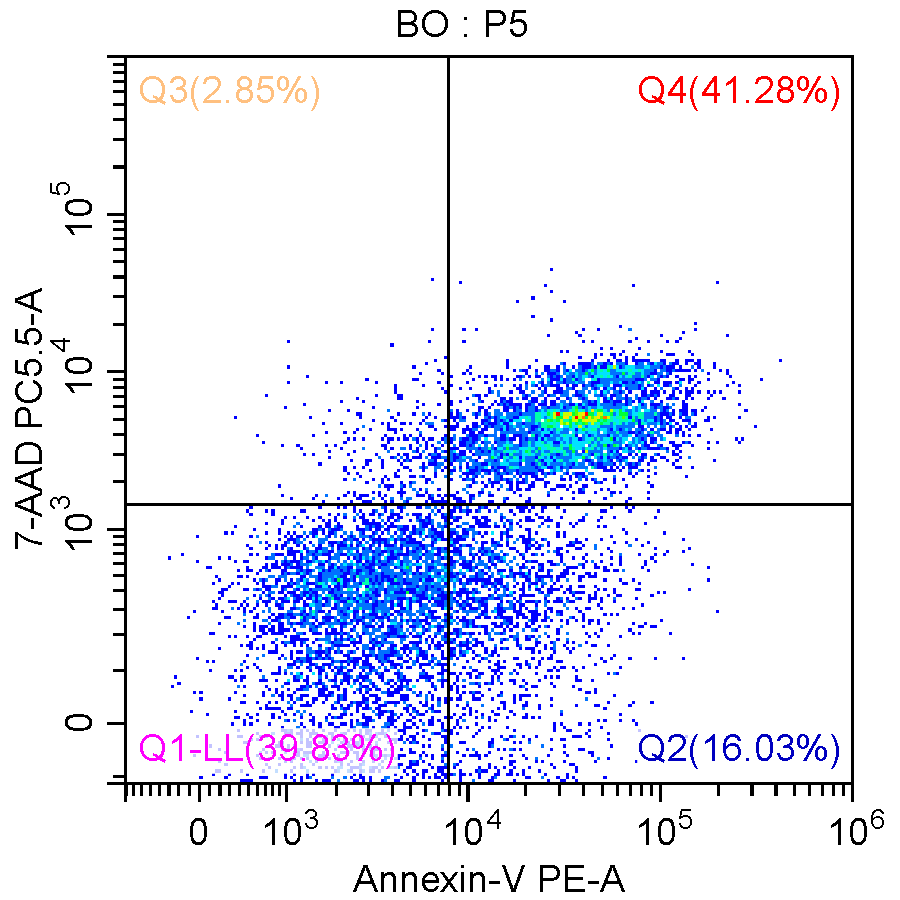

Supplement: Supplementary file 6 [file DataSheet4.ZIP › A/3-5C+LPS/BO_Plot1.bmp]

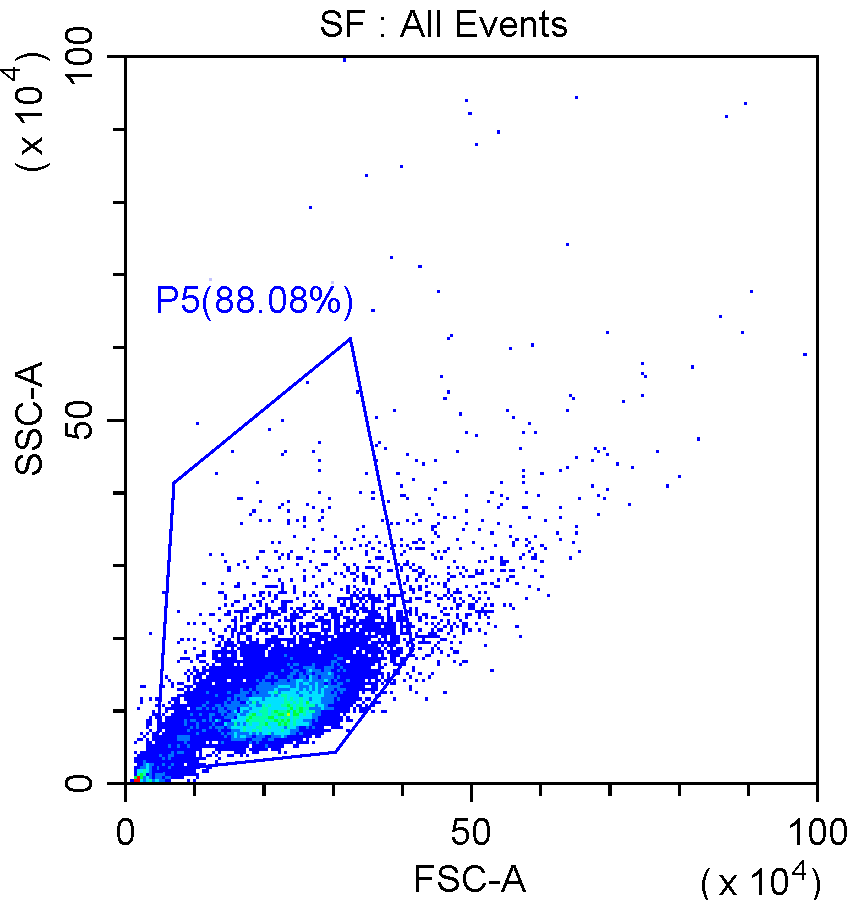

Supplement: Supplementary file 6 [file DataSheet4.ZIP › A/5-3D+LPS/SF _Plot1.bmp]

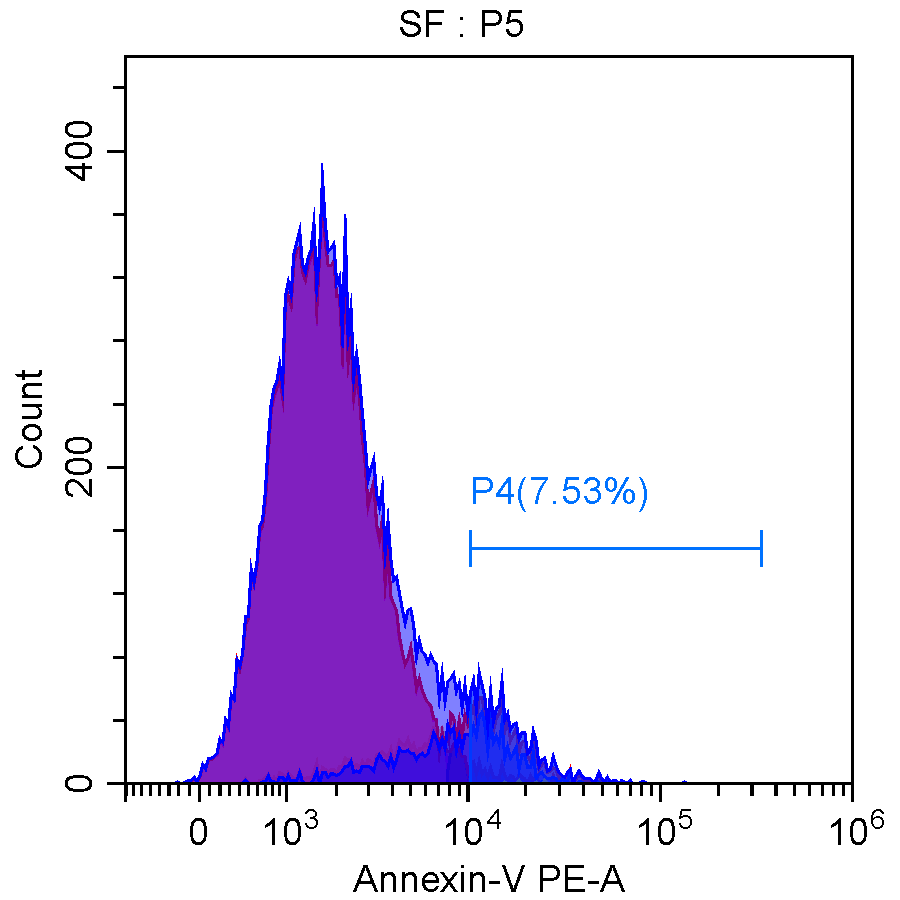

Supplement: Supplementary file 6 [file DataSheet4.ZIP › A/5-3D+LPS/SF_ Plot1.bmp]

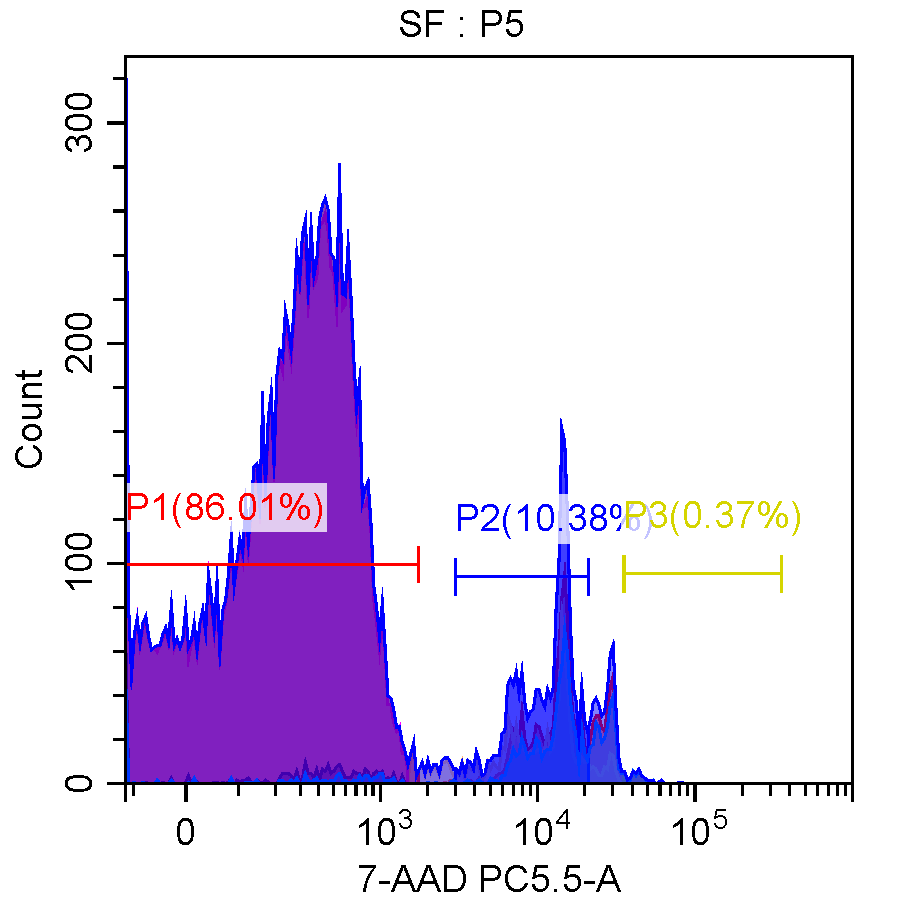

Supplement: Supplementary file 6 [file DataSheet4.ZIP › A/5-3D+LPS/SF_Plot.bmp]

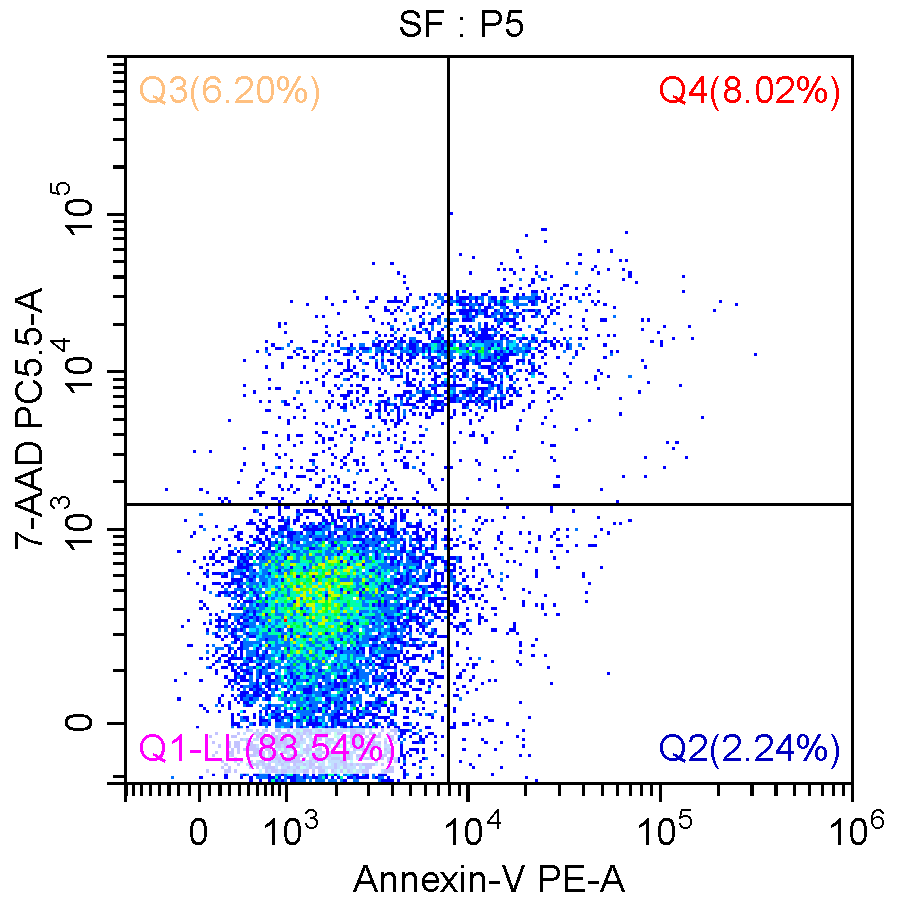

Supplement: Supplementary file 6 [file DataSheet4.ZIP › A/5-3D+LPS/SF_Plot1.bmp]

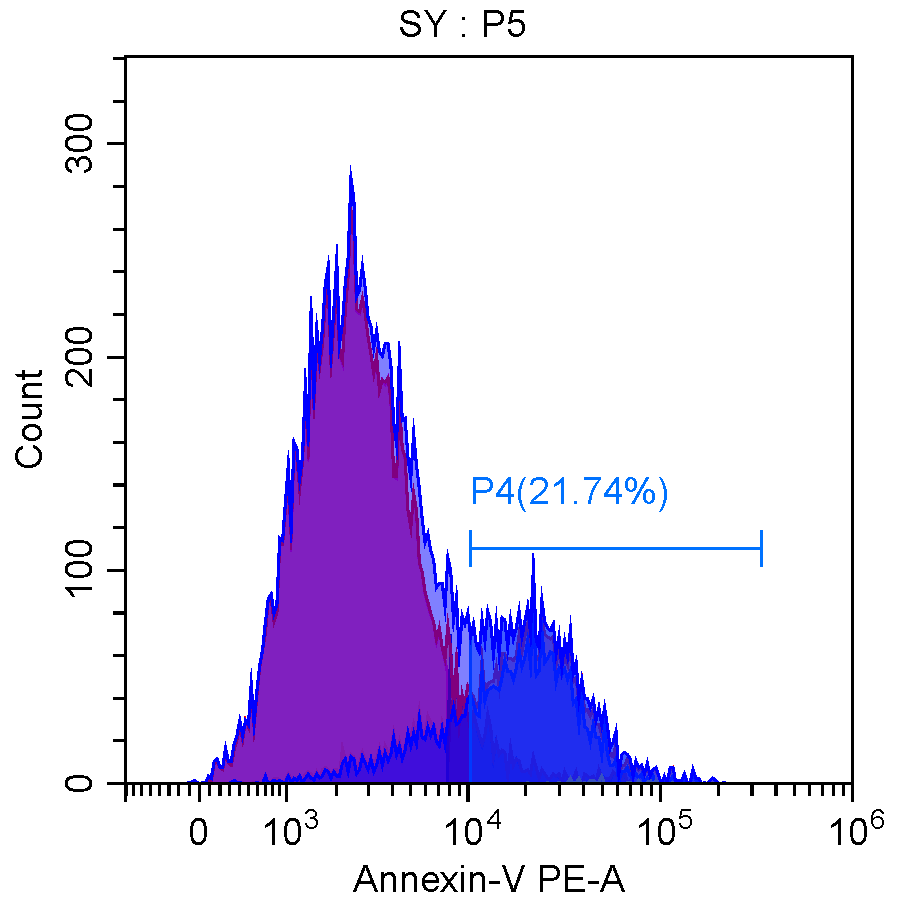

Supplement: Supplementary file 6 [file DataSheet4.ZIP › A/7-3D+LPS/SY _Plot1.bmp]

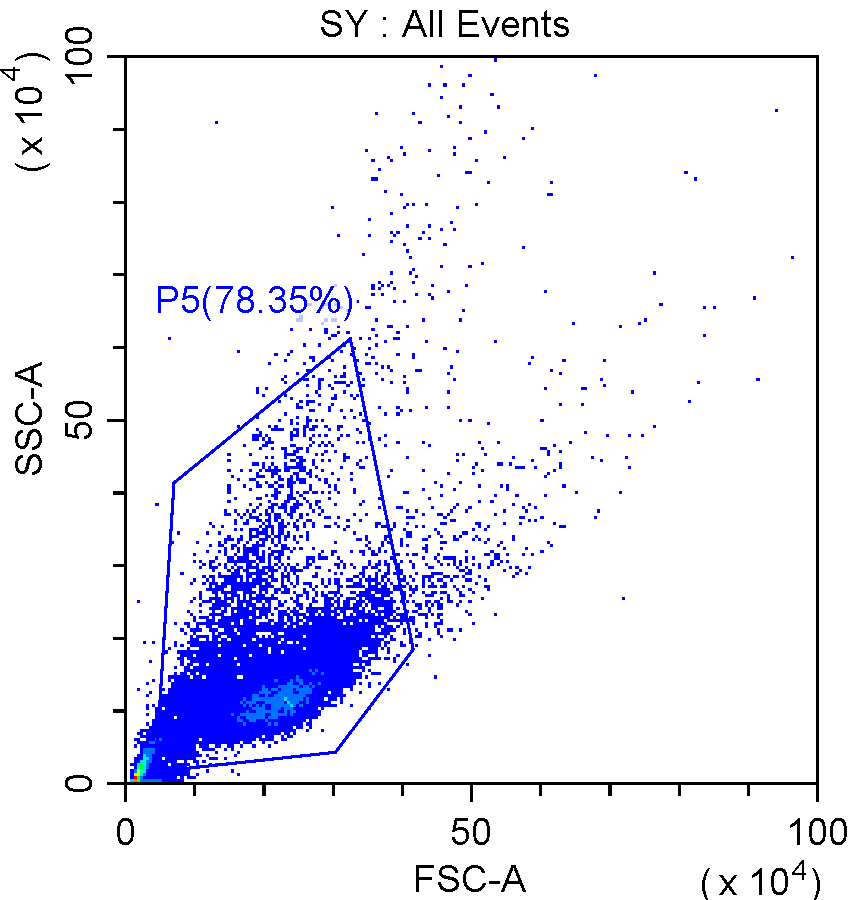

Supplement: Supplementary file 6 [file DataSheet4.ZIP › A/7-3D+LPS/SY_ Plot1.bmp]

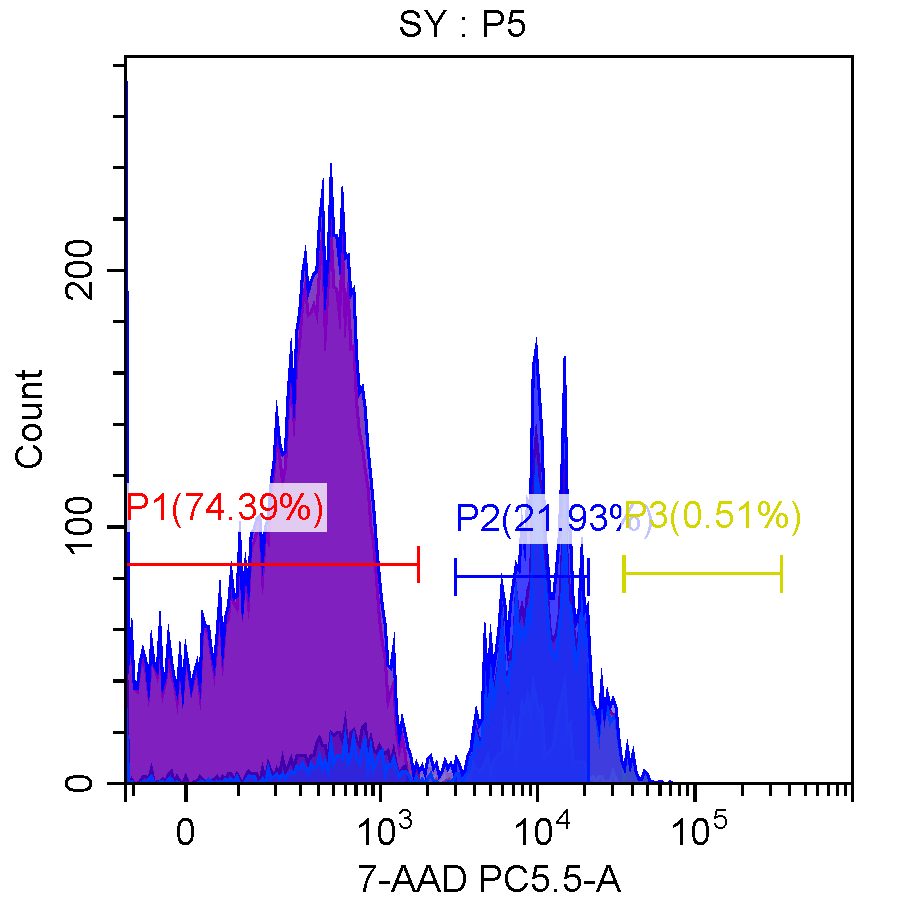

Supplement: Supplementary file 6 [file DataSheet4.ZIP › A/7-3D+LPS/SY_Plot.bmp]

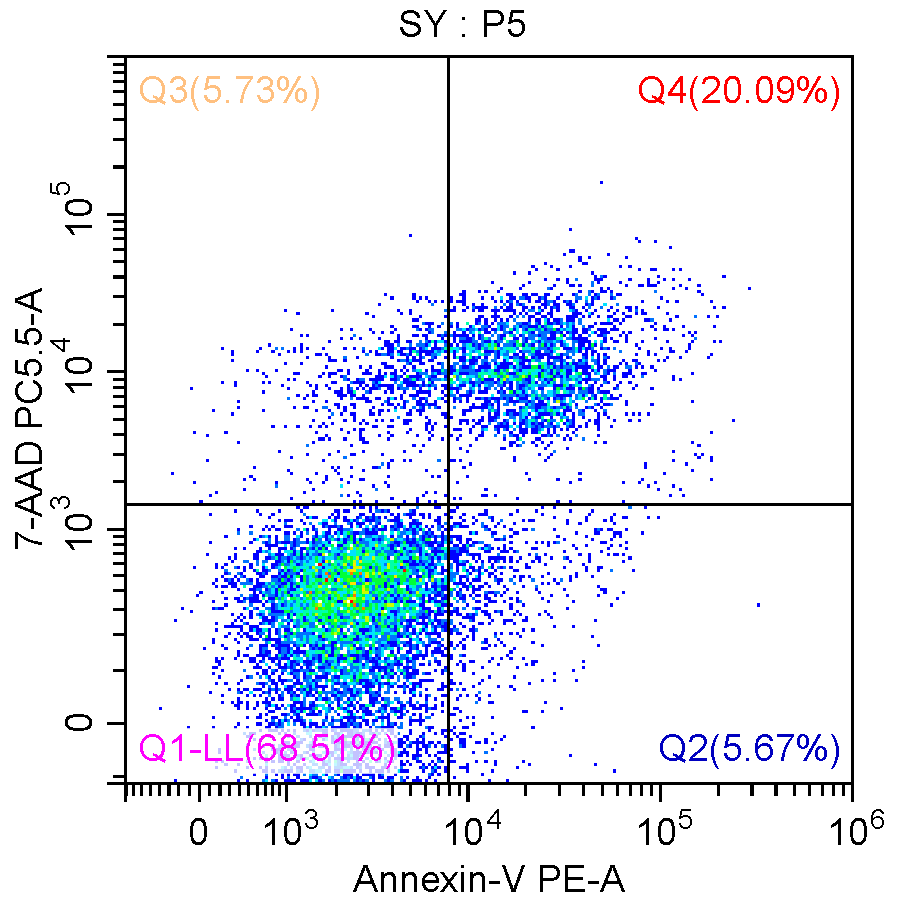

Supplement: Supplementary file 6 [file DataSheet4.ZIP › A/7-3D+LPS/SY_Plot1.bmp]

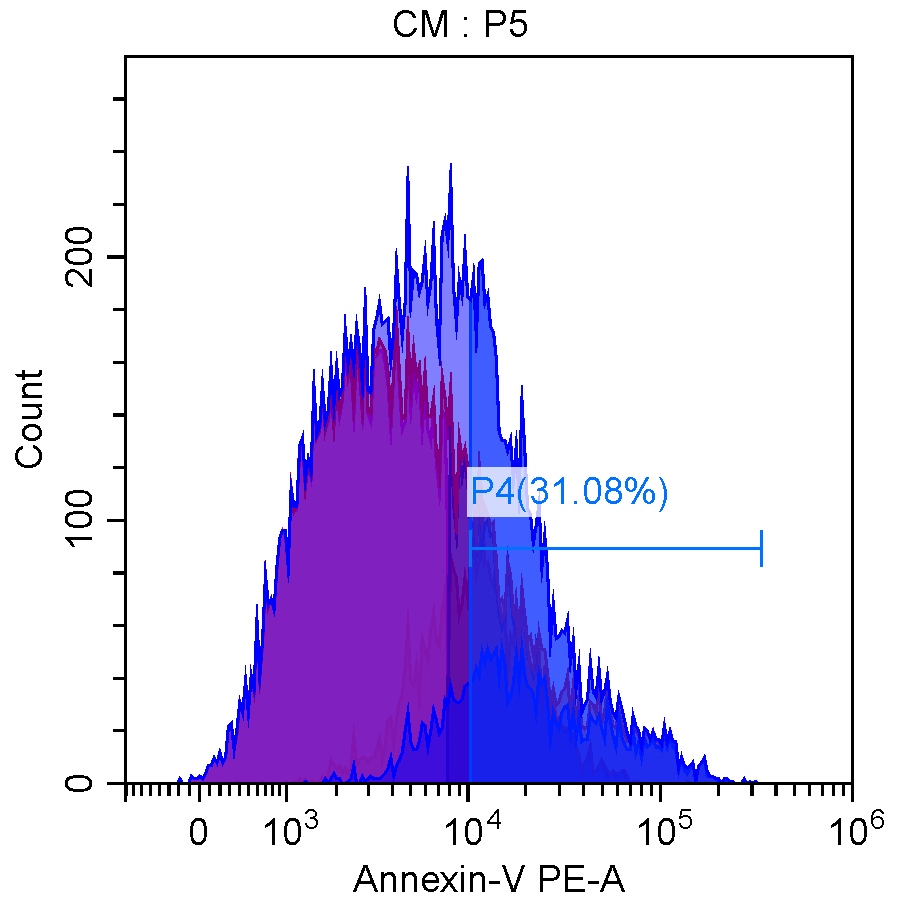

Supplement: Supplementary file 6 [file DataSheet4.ZIP › A/9-2A+LPS/CM _Plot1.bmp]

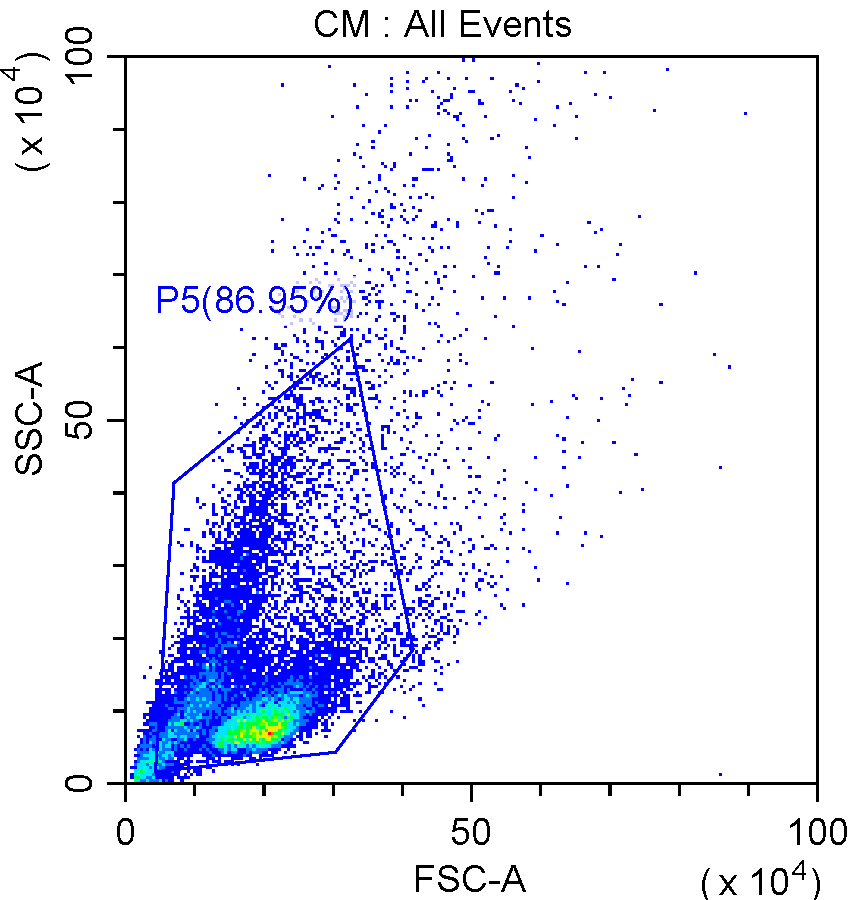

Supplement: Supplementary file 6 [file DataSheet4.ZIP › A/9-2A+LPS/CM_ Plot1.bmp]

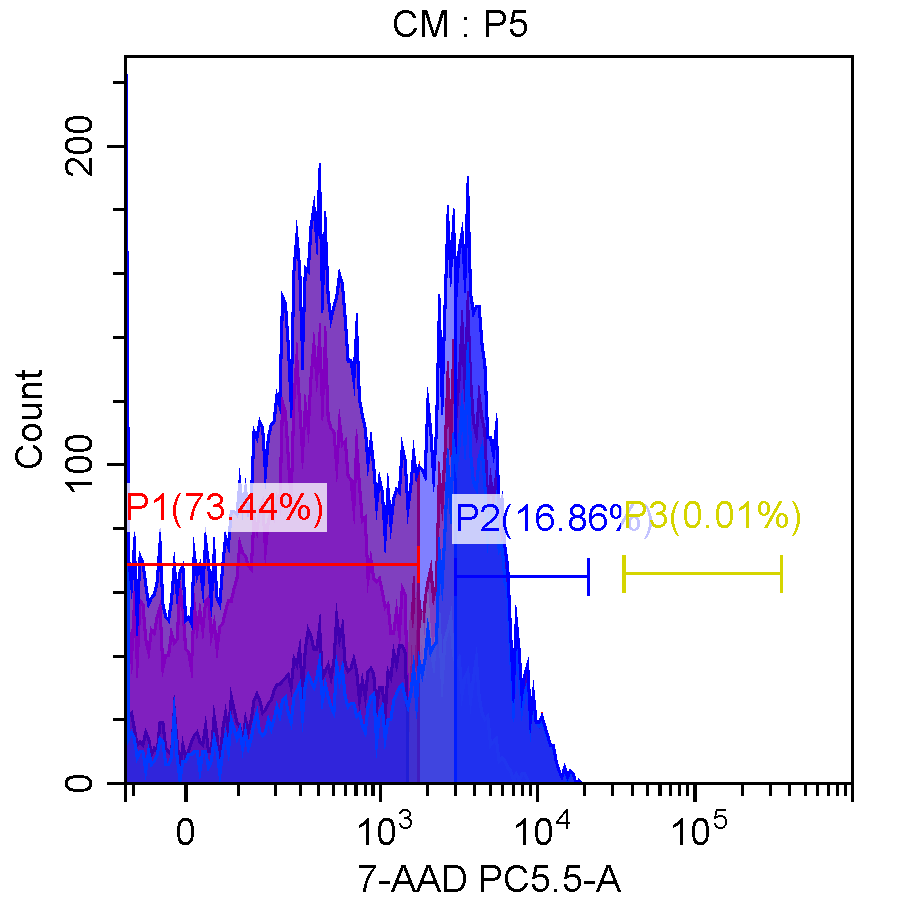

Supplement: Supplementary file 6 [file DataSheet4.ZIP › A/9-2A+LPS/CM_Plot.bmp]

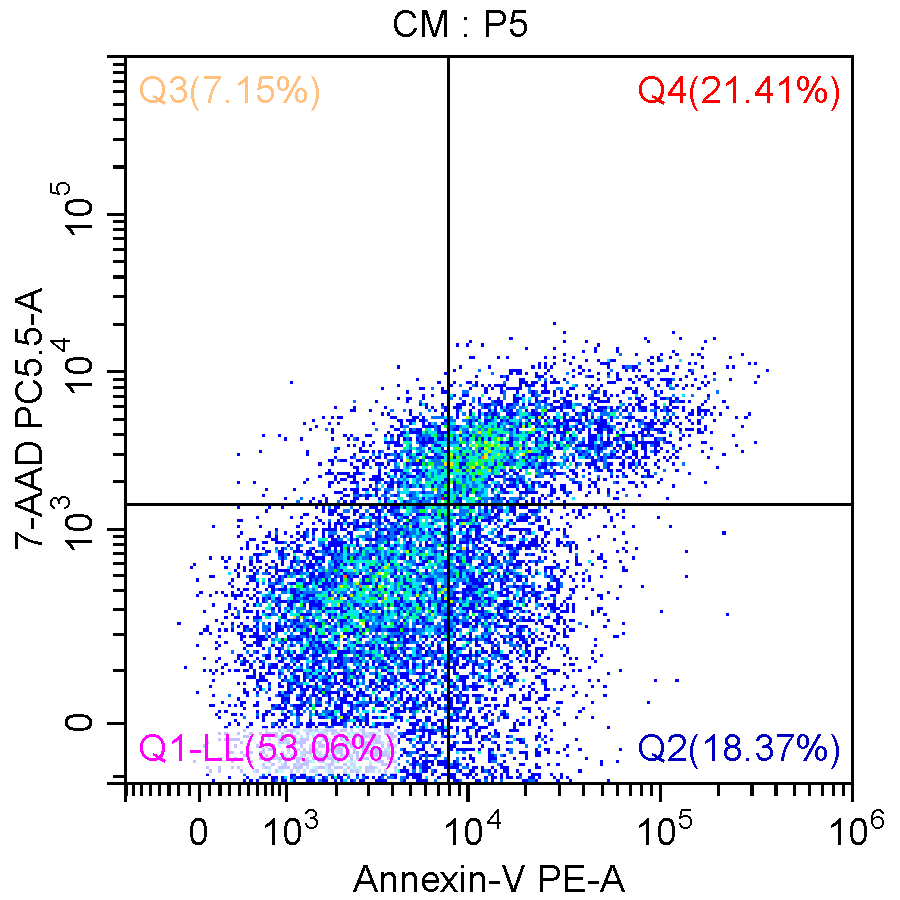

Supplement: Supplementary file 6 [file DataSheet4.ZIP › A/9-2A+LPS/CM_Plot1.bmp]

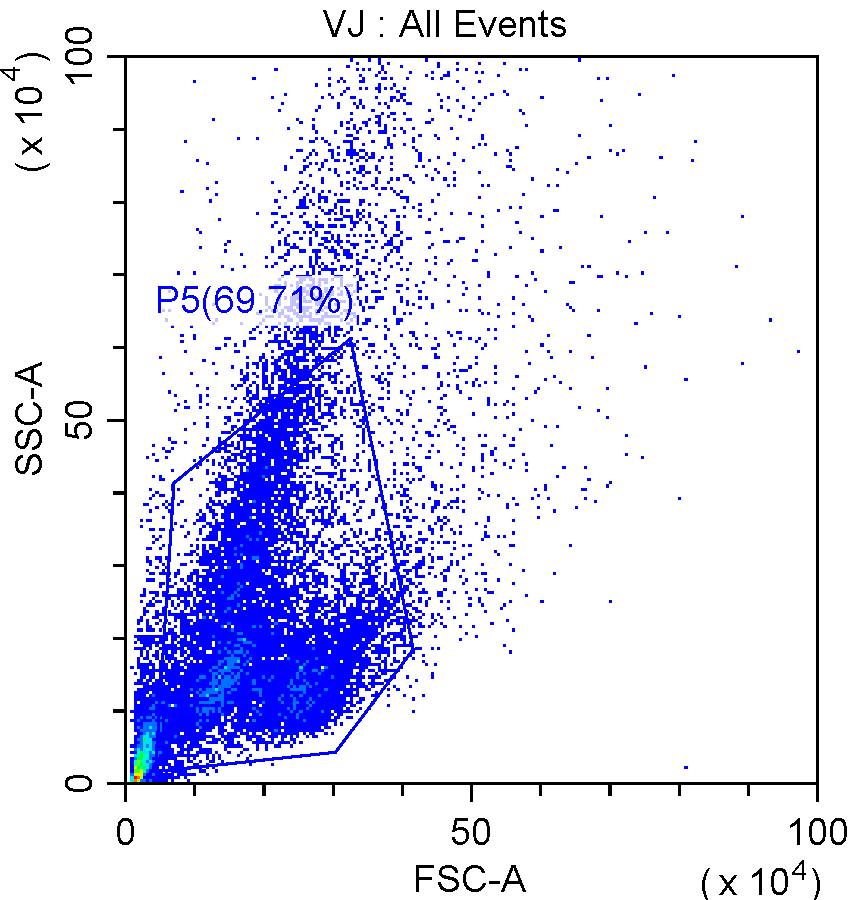

Supplement: Supplementary file 6 [file DataSheet4.ZIP › A/LPS/VJ _Plot1.bmp]

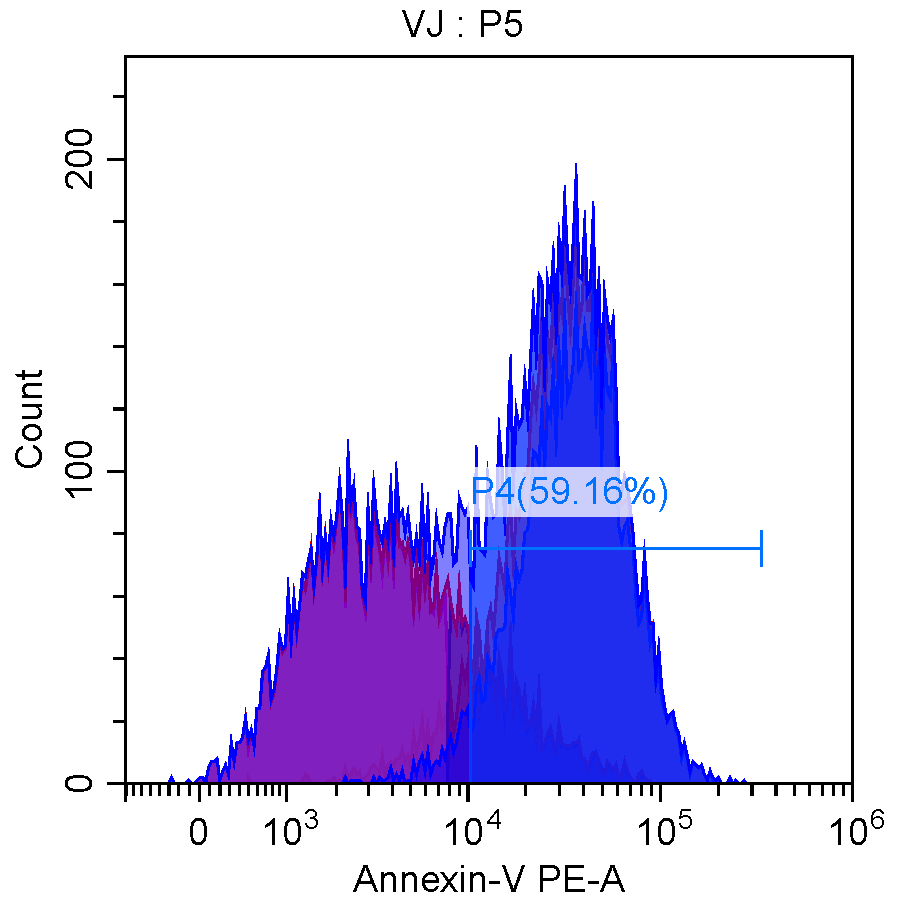

Supplement: Supplementary file 6 [file DataSheet4.ZIP › A/LPS/VJ_ Plot.bmp]

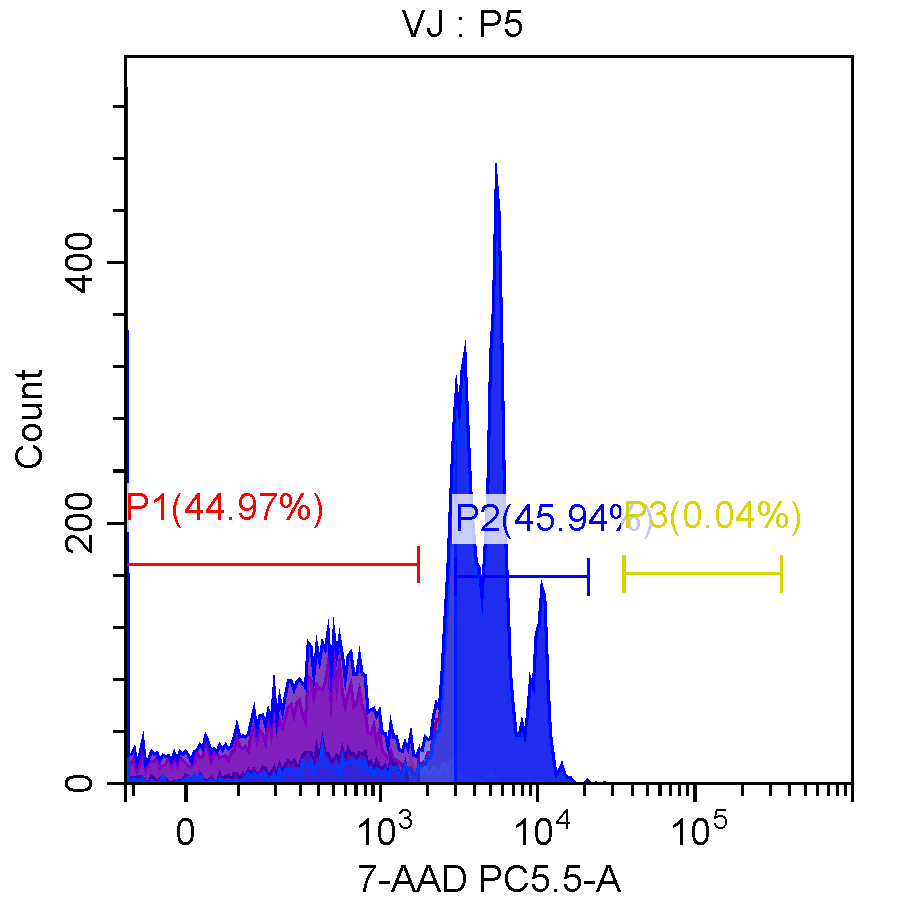

Supplement: Supplementary file 6 [file DataSheet4.ZIP › A/LPS/VJ_Plot.bmp]

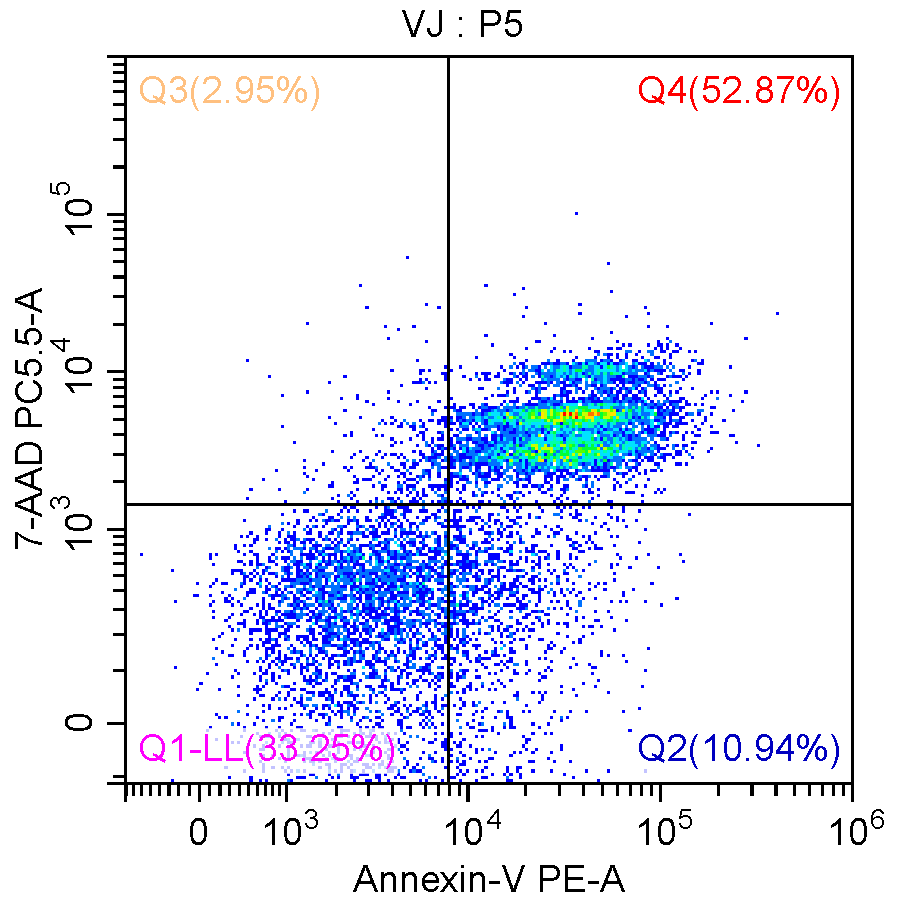

Supplement: Supplementary file 6 [file DataSheet4.ZIP › A/LPS/VJ_Plot1.bmp]

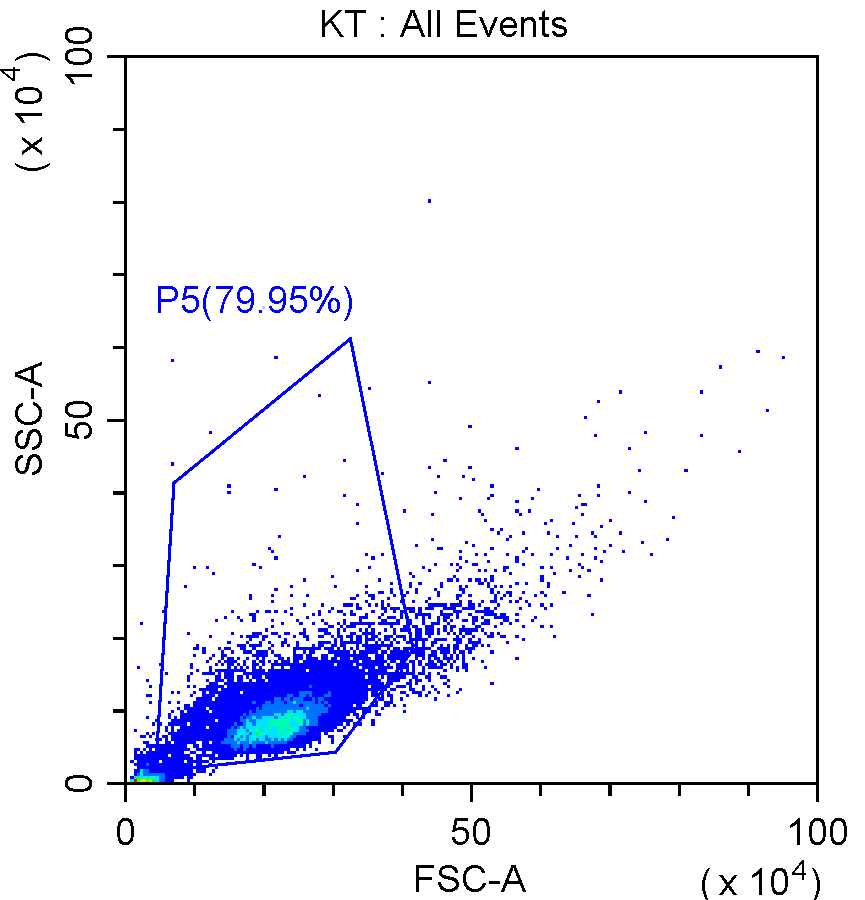

Supplement: Supplementary file 6 [file DataSheet4.ZIP › A/VEHICLE/KT _Plot.bmp]

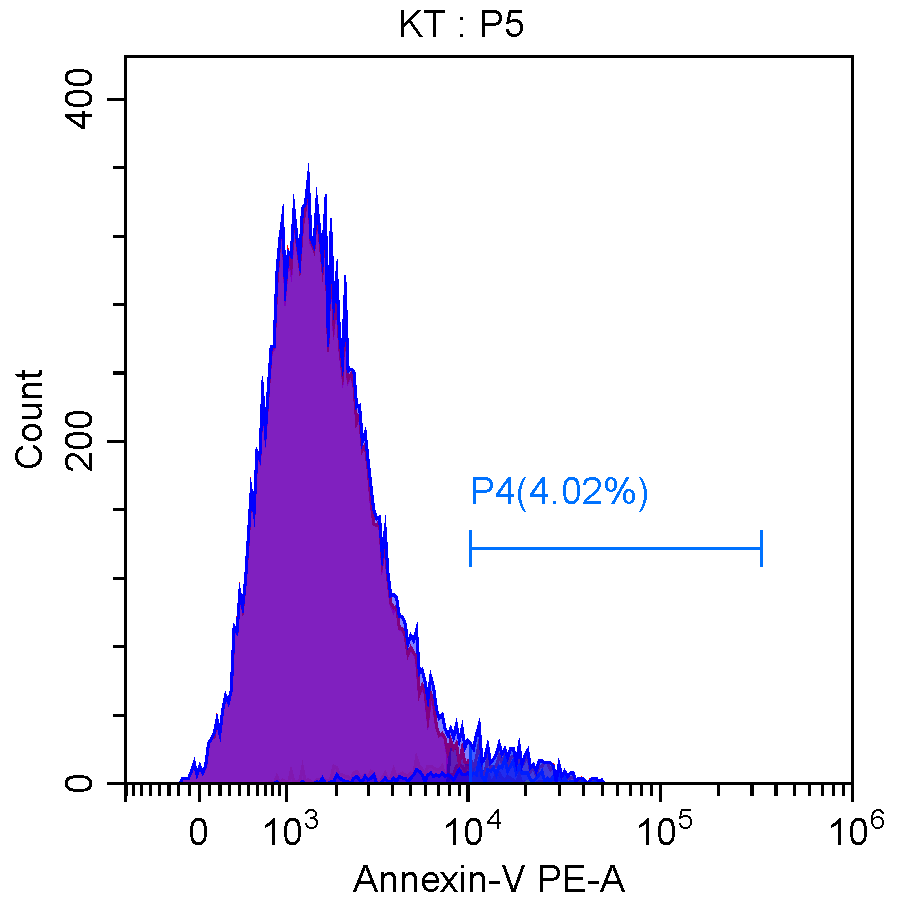

Supplement: Supplementary file 6 [file DataSheet4.ZIP › A/VEHICLE/KT_ Plot.bmp]

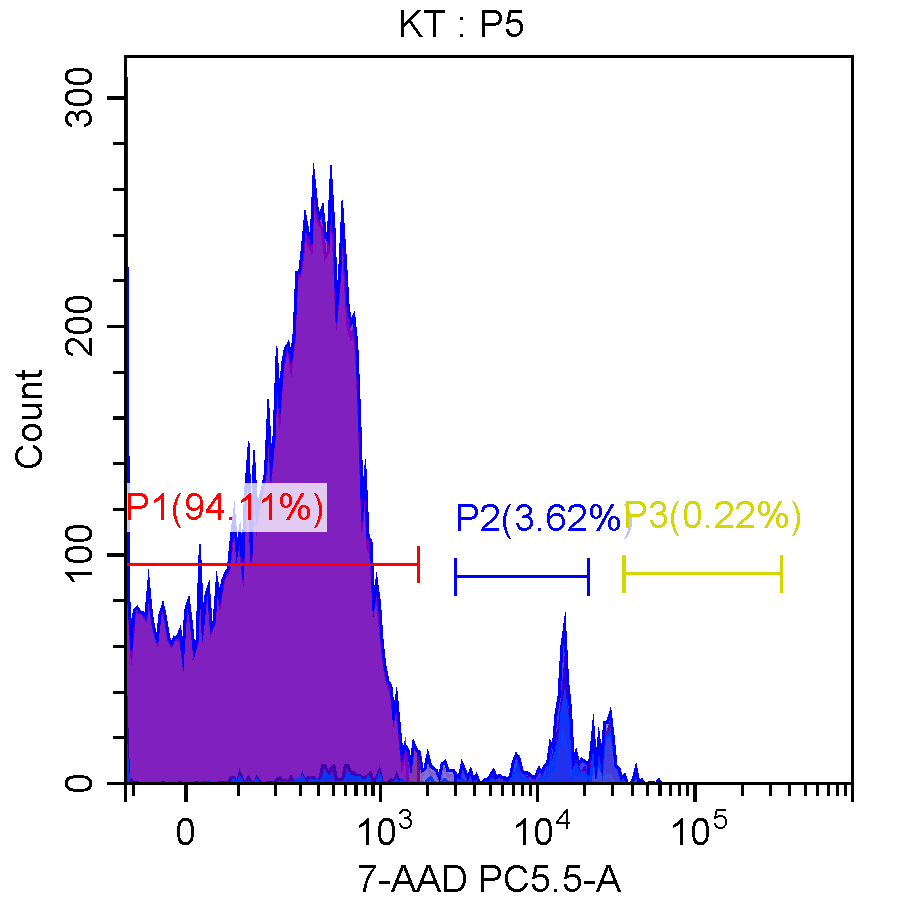

Supplement: Supplementary file 6 [file DataSheet4.ZIP › A/VEHICLE/KT_Plot.bmp]

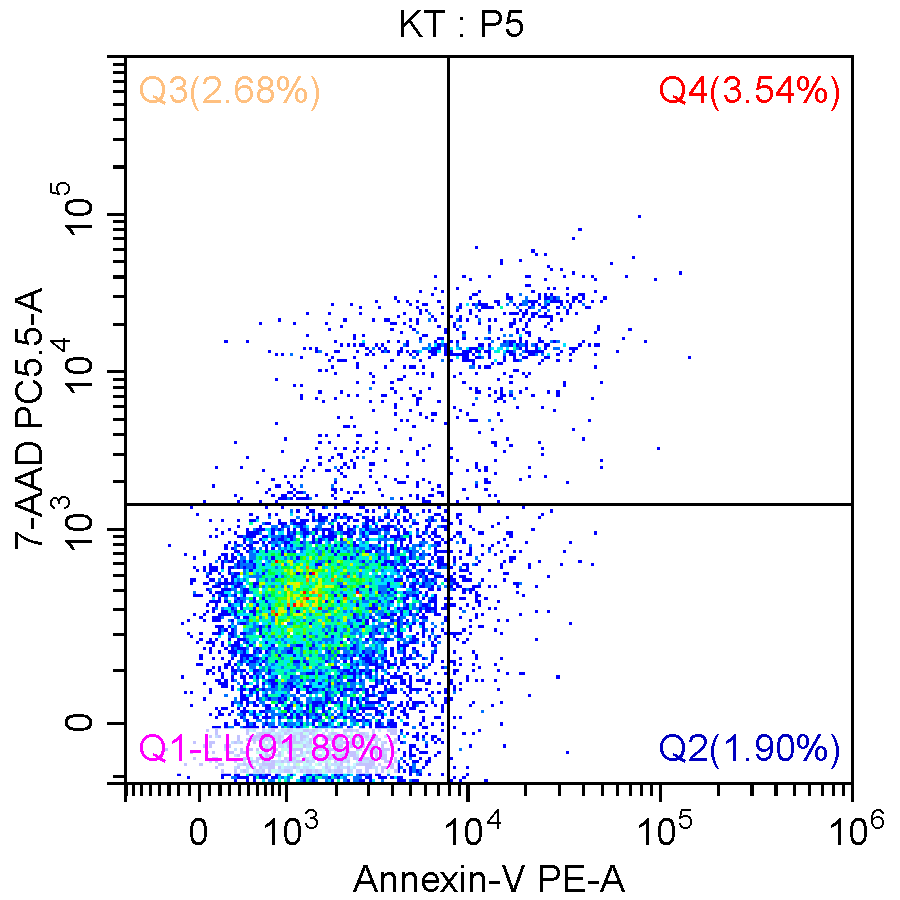

Supplement: Supplementary file 6 [file DataSheet4.ZIP › A/VEHICLE/KT_Plot1.bmp]

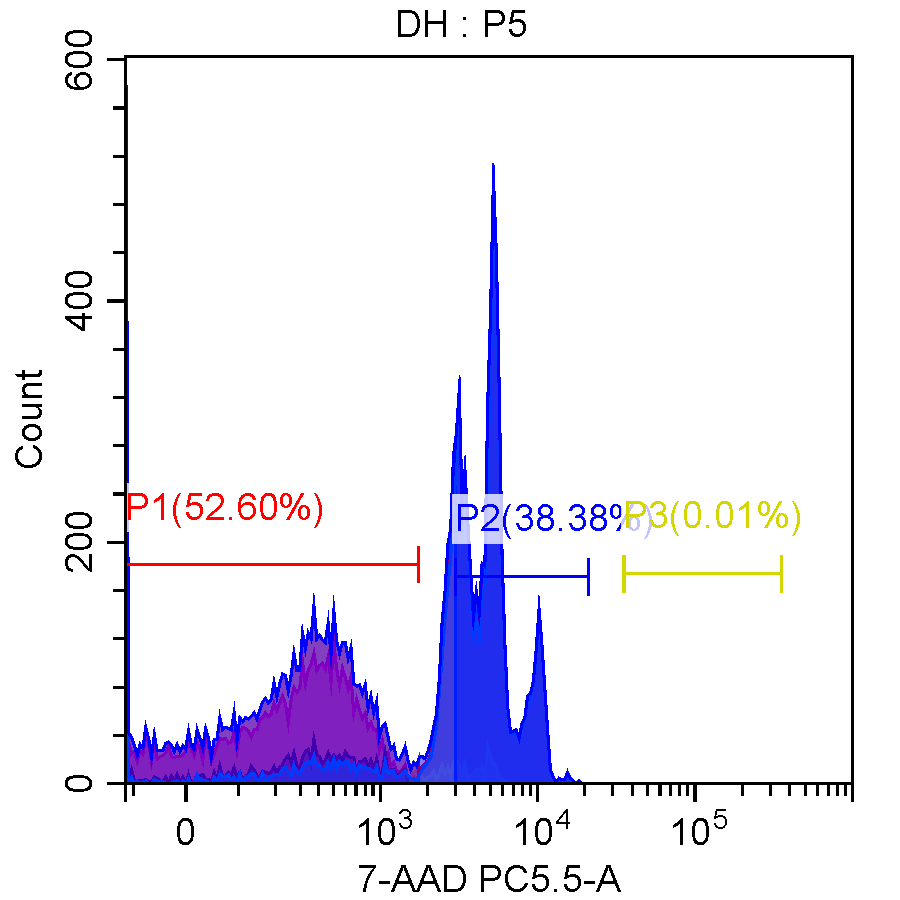

Supplement: Supplementary file 6 [file DataSheet4.ZIP › C/LP S/DH _Plot1.bmp]

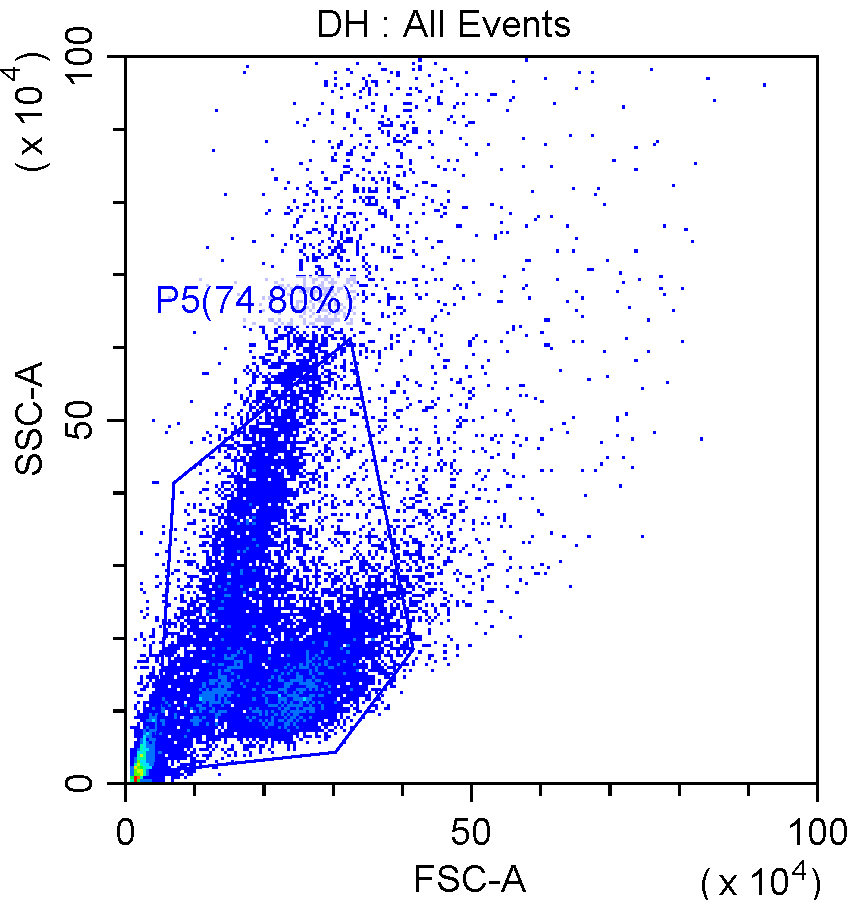

Supplement: Supplementary file 6 [file DataSheet4.ZIP › C/LP S/DH_ Plot1.bmp]

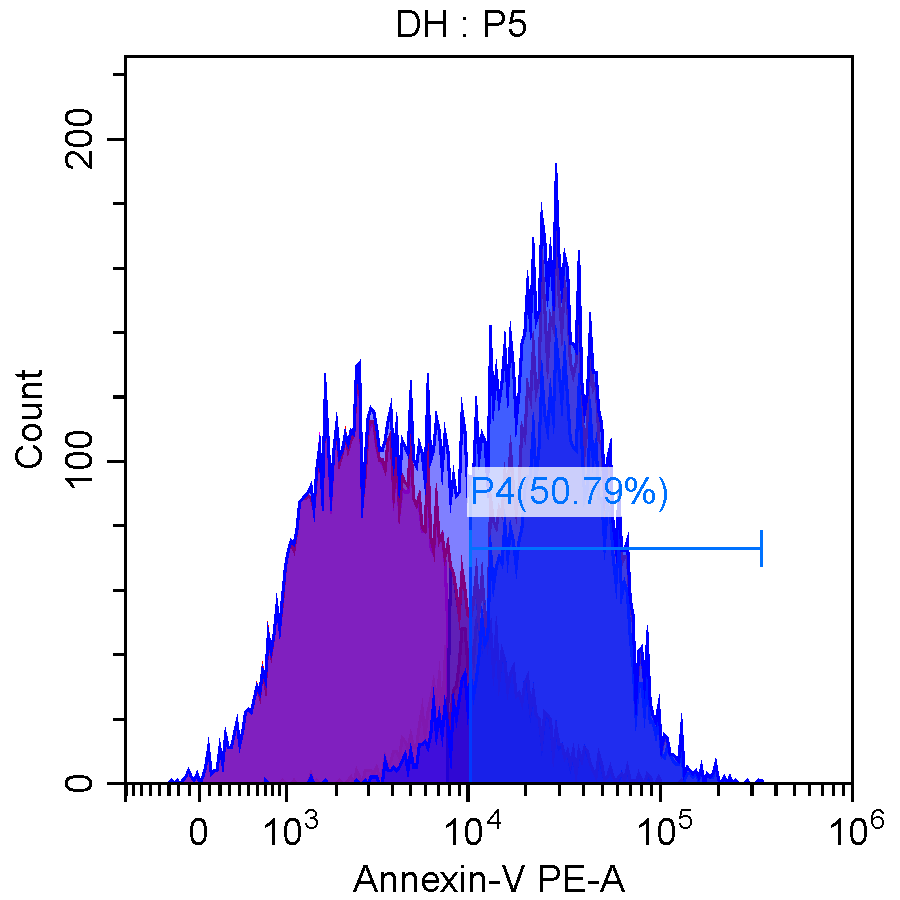

Supplement: Supplementary file 6 [file DataSheet4.ZIP › C/LP S/DH_Plot.bmp]

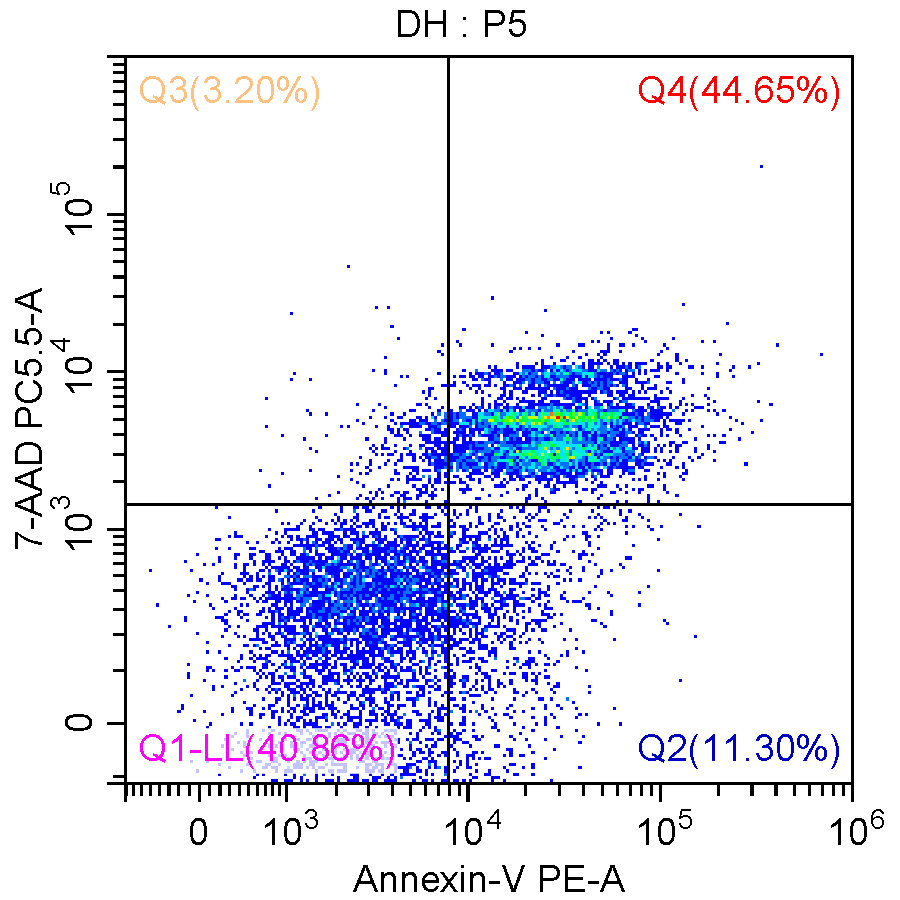

Supplement: Supplementary file 6 [file DataSheet4.ZIP › C/LP S/DH_Plot1.bmp]

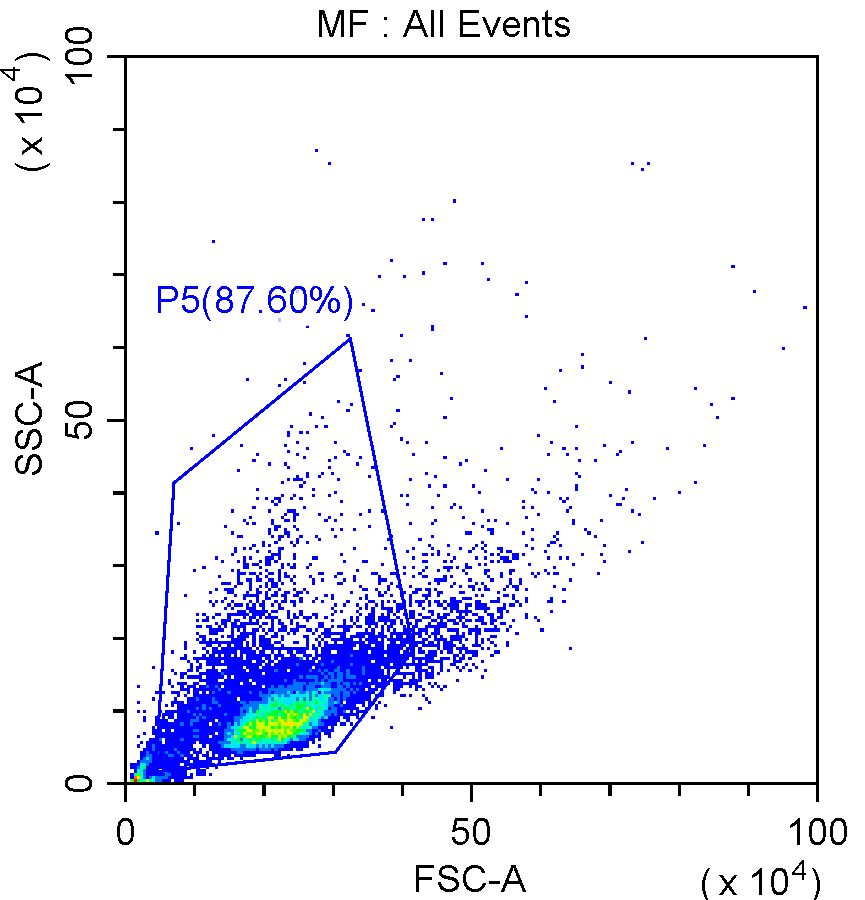

Supplement: Supplementary file 6 [file DataSheet4.ZIP › C/ST10+LPS/MF _Plot1.bmp]

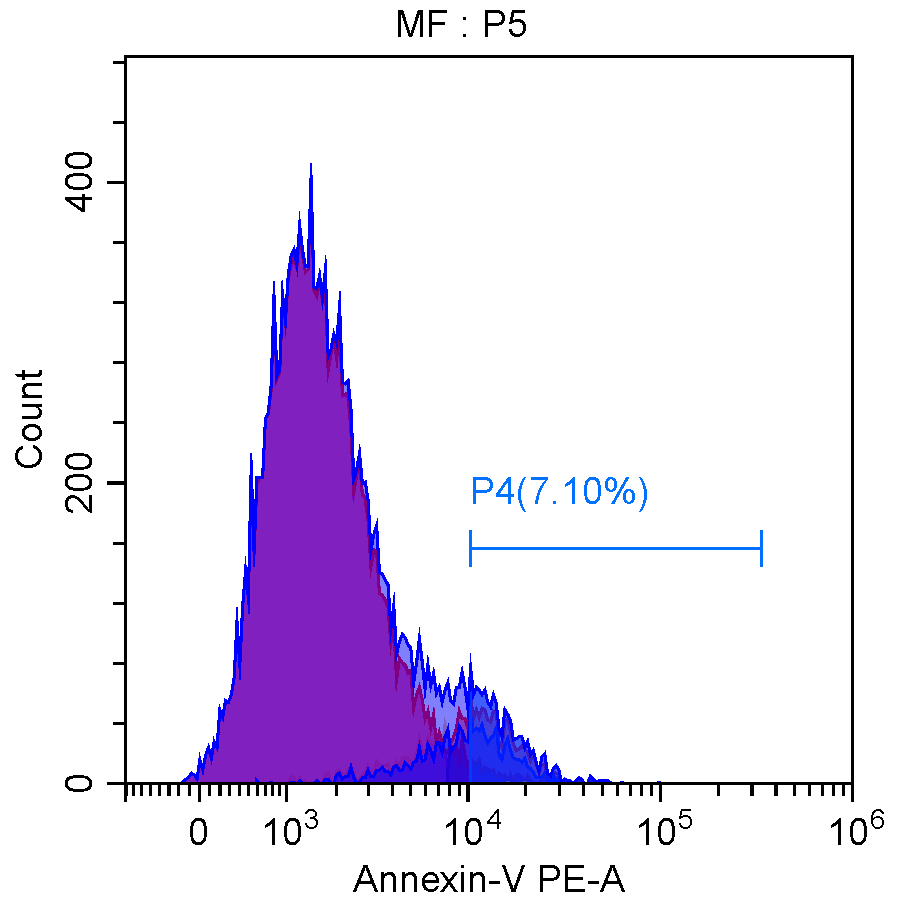

Supplement: Supplementary file 6 [file DataSheet4.ZIP › C/ST10+LPS/MF_ Plot1.bmp]

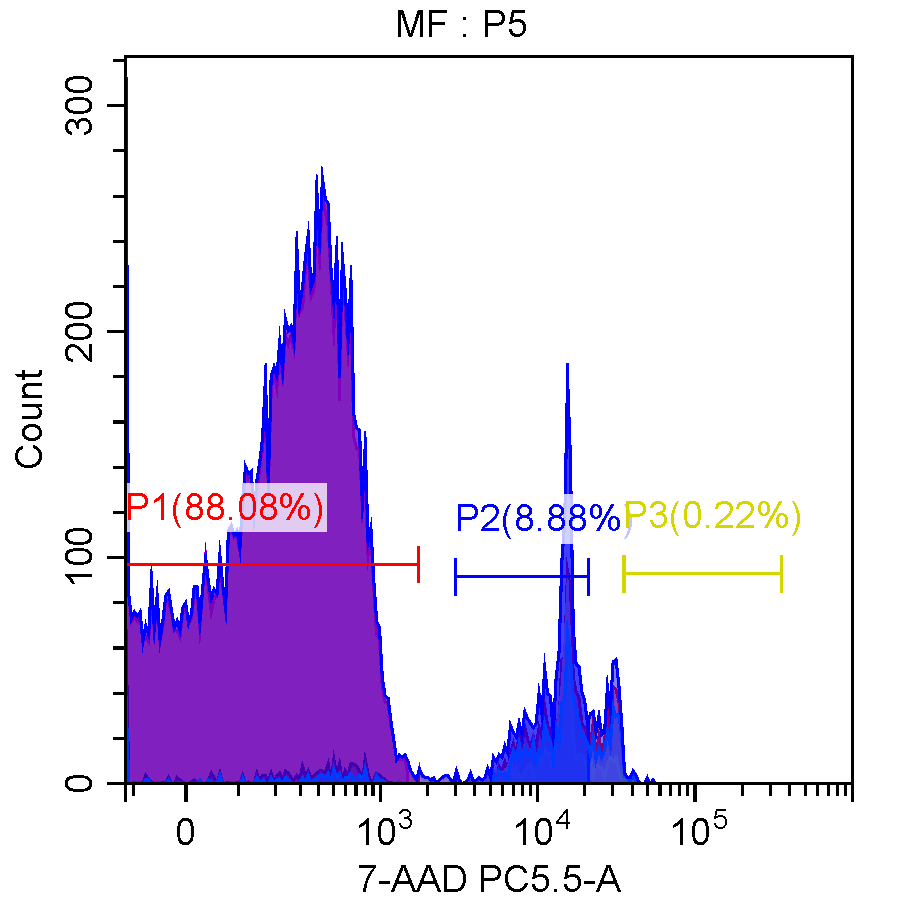

Supplement: Supplementary file 6 [file DataSheet4.ZIP › C/ST10+LPS/MF_Plot.bmp]

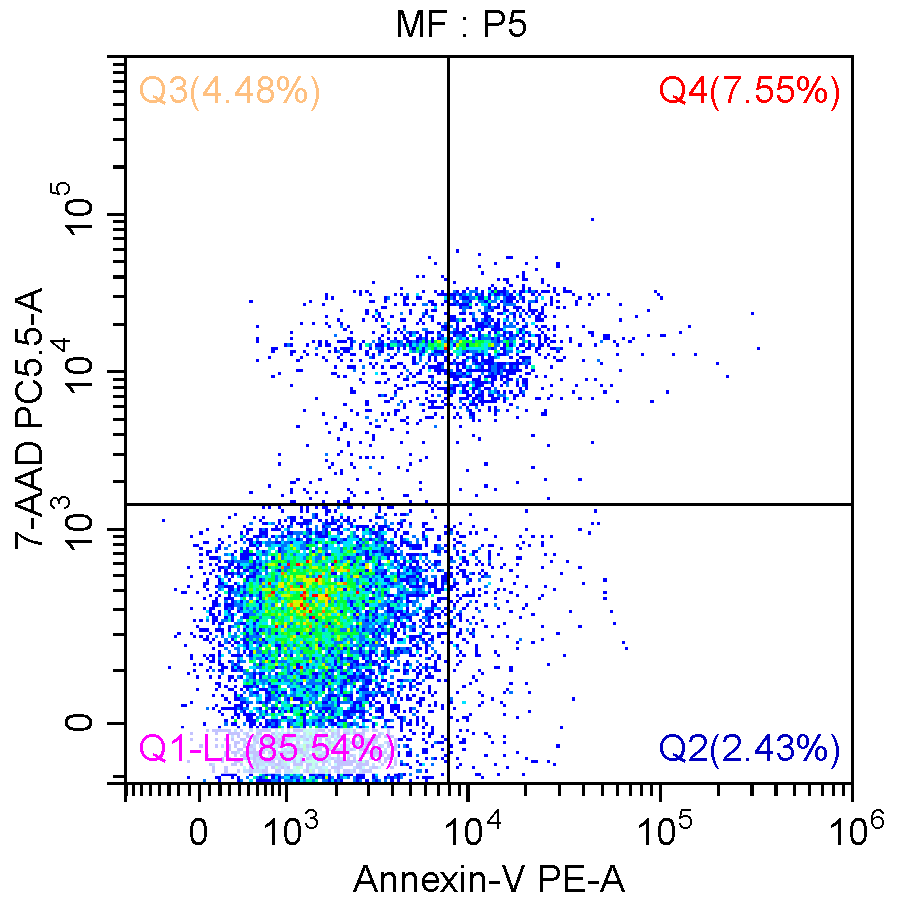

Supplement: Supplementary file 6 [file DataSheet4.ZIP › C/ST10+LPS/MF_Plot1.bmp]

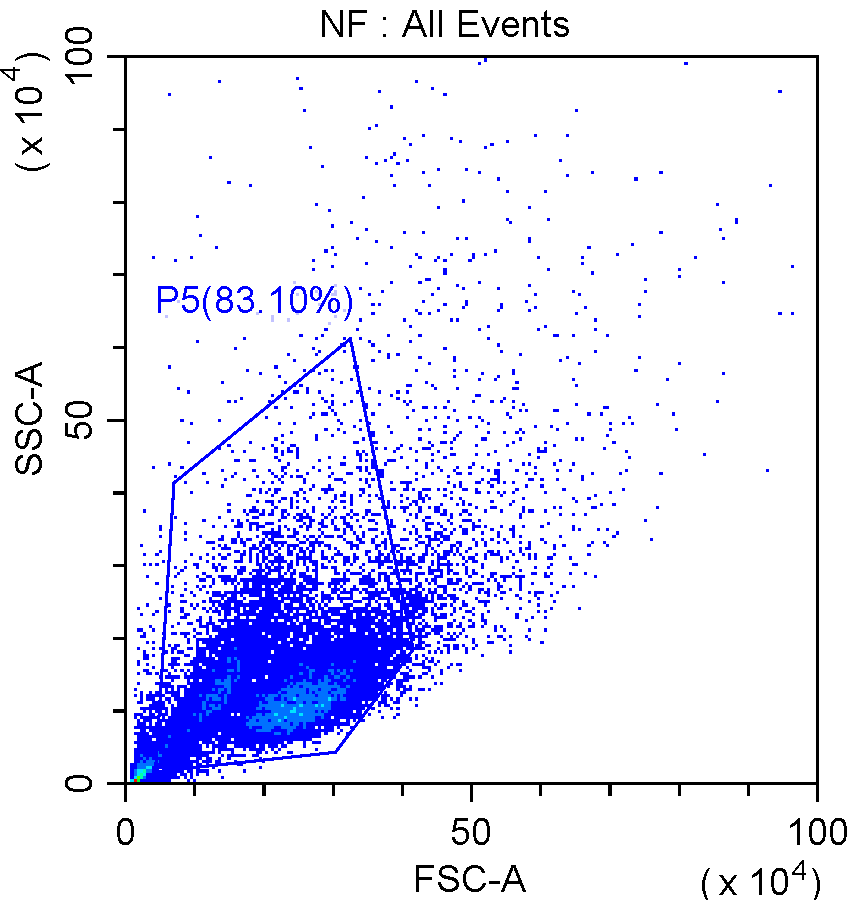

Supplement: Supplementary file 6 [file DataSheet4.ZIP › C/ST2.5+LPS/NF _Plot1.bmp]

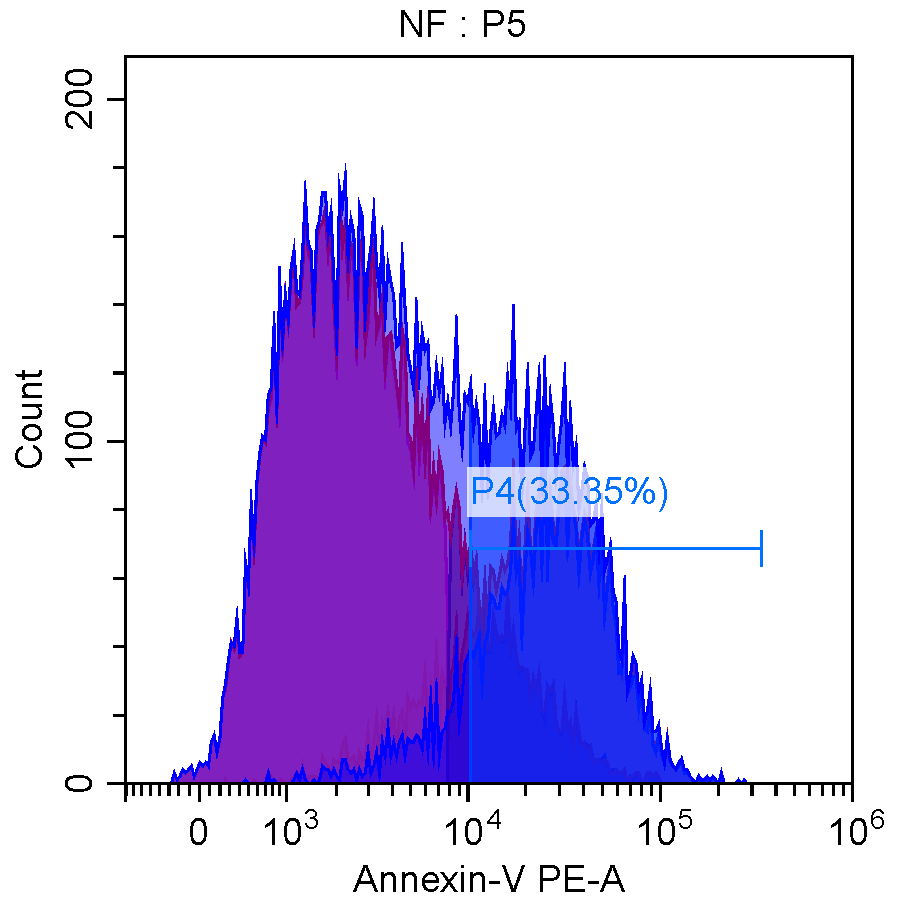

Supplement: Supplementary file 6 [file DataSheet4.ZIP › C/ST2.5+LPS/NF_ Plot1.bmp]

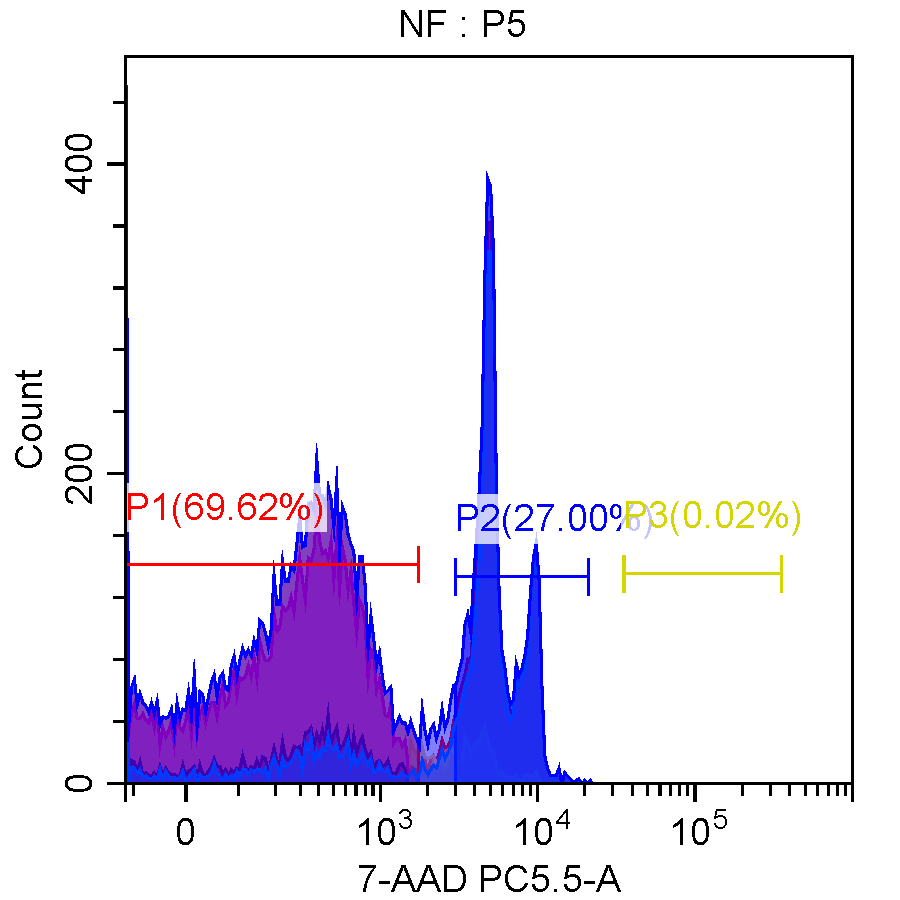

Supplement: Supplementary file 6 [file DataSheet4.ZIP › C/ST2.5+LPS/NF_Plot.bmp]

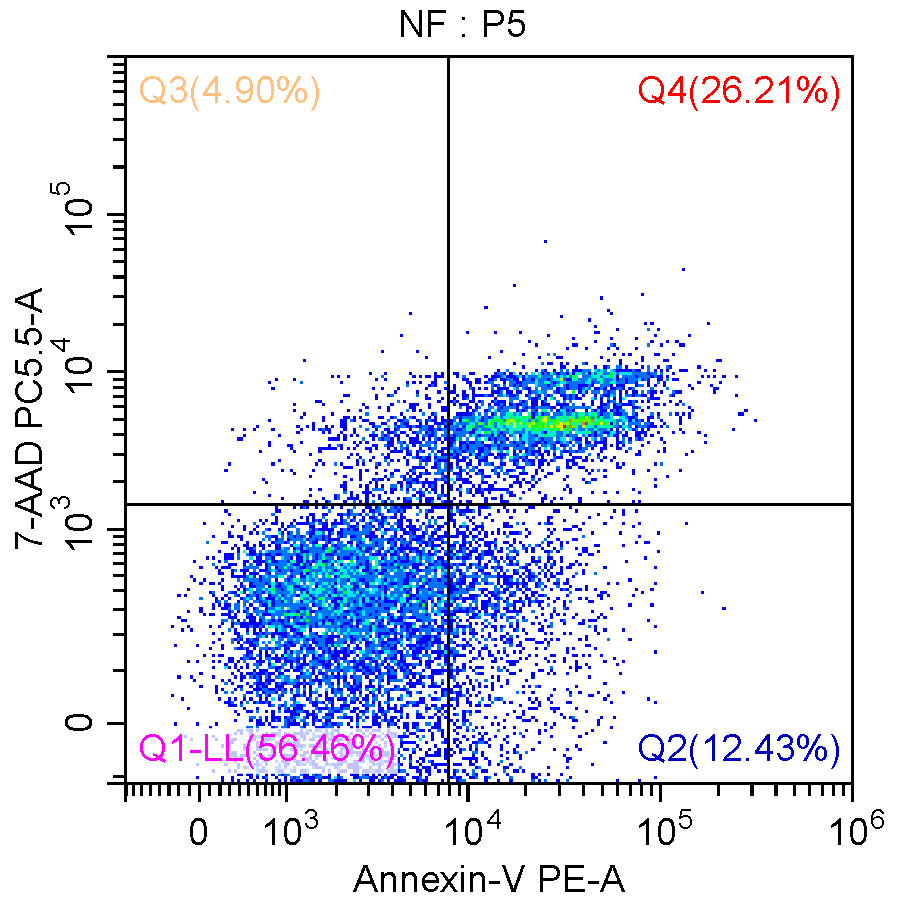

Supplement: Supplementary file 6 [file DataSheet4.ZIP › C/ST2.5+LPS/NF_Plot1.bmp]

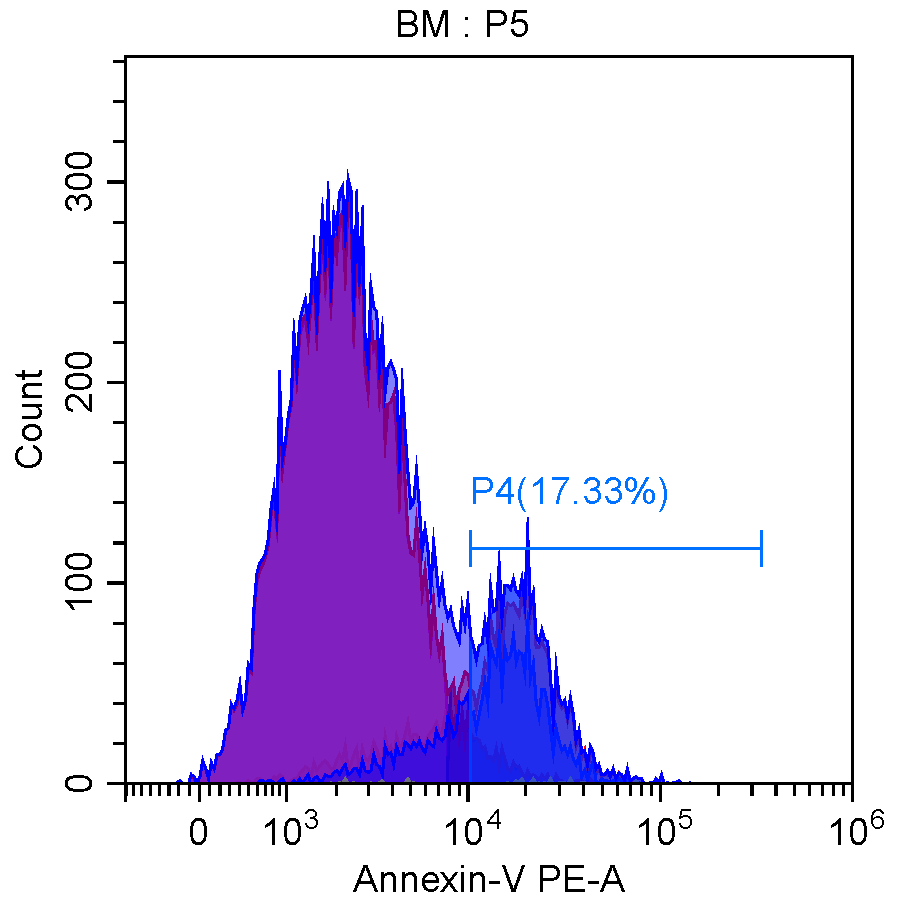

Supplement: Supplementary file 6 [file DataSheet4.ZIP › C/ST5+LPS/BM _Plot1.bmp]

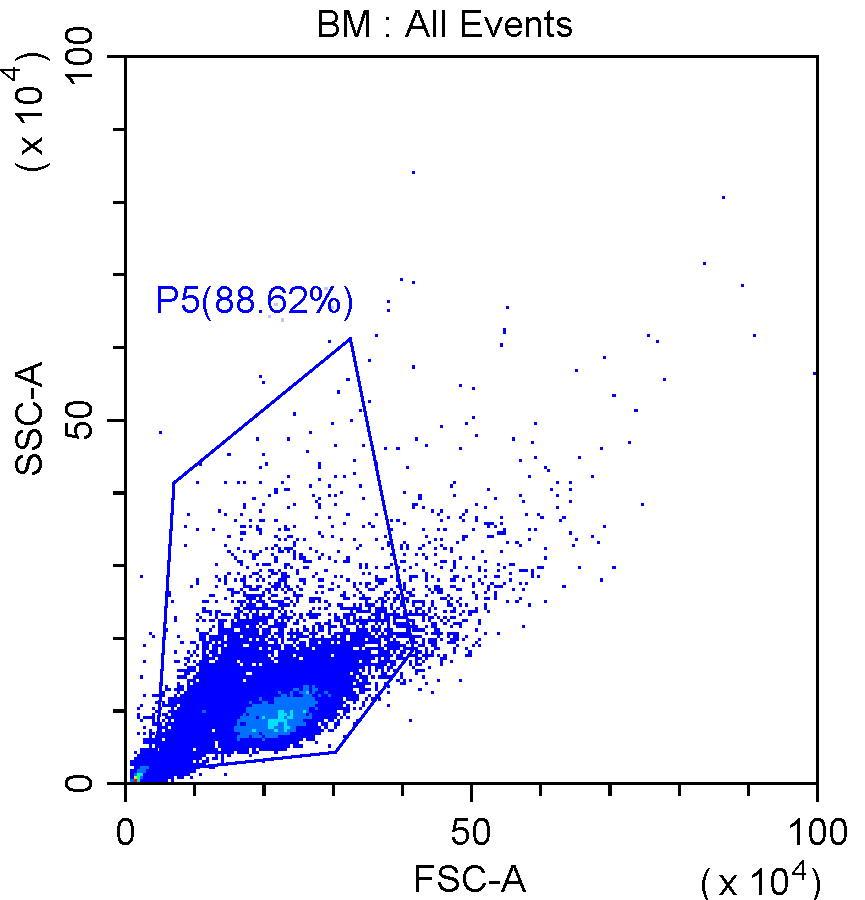

Supplement: Supplementary file 6 [file DataSheet4.ZIP › C/ST5+LPS/BM_ Plot1.bmp]

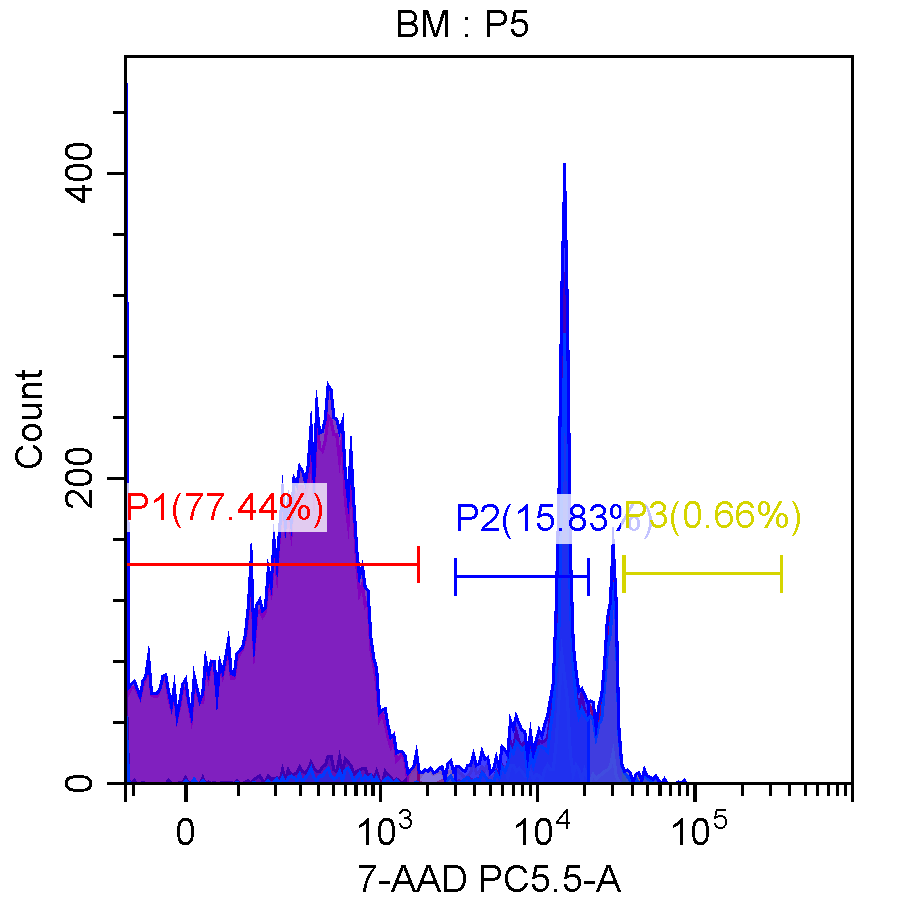

Supplement: Supplementary file 6 [file DataSheet4.ZIP › C/ST5+LPS/BM_Plot.bmp]

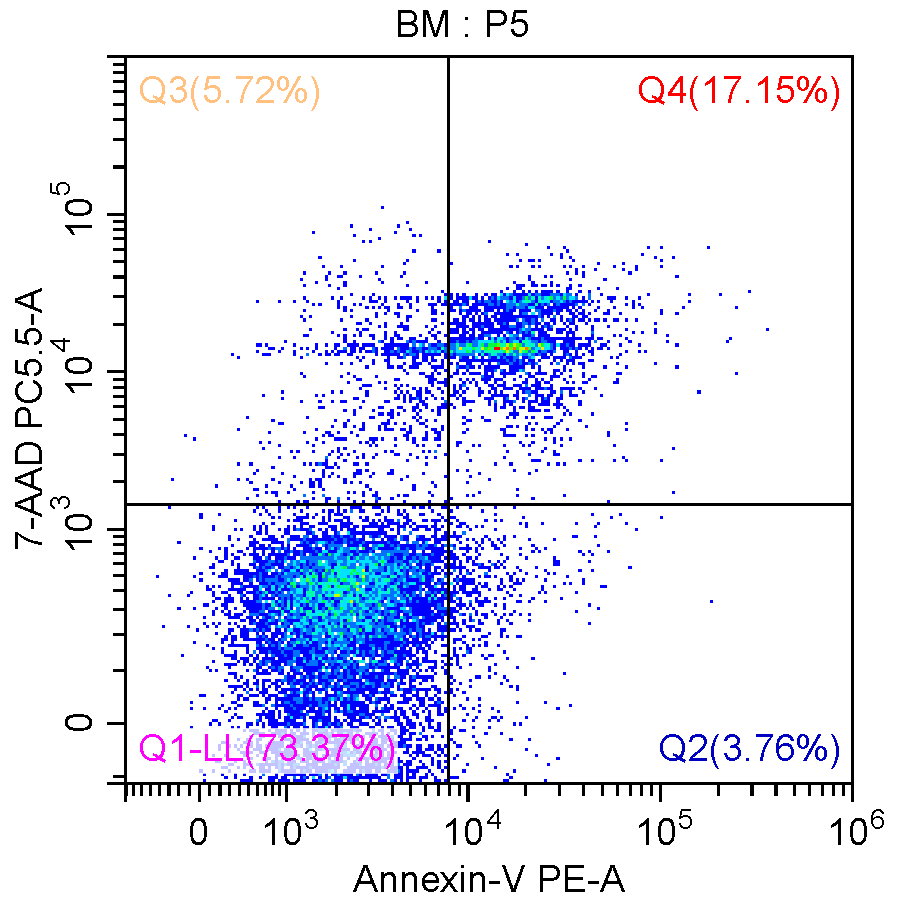

Supplement: Supplementary file 6 [file DataSheet4.ZIP › C/ST5+LPS/BM_Plot1.bmp]

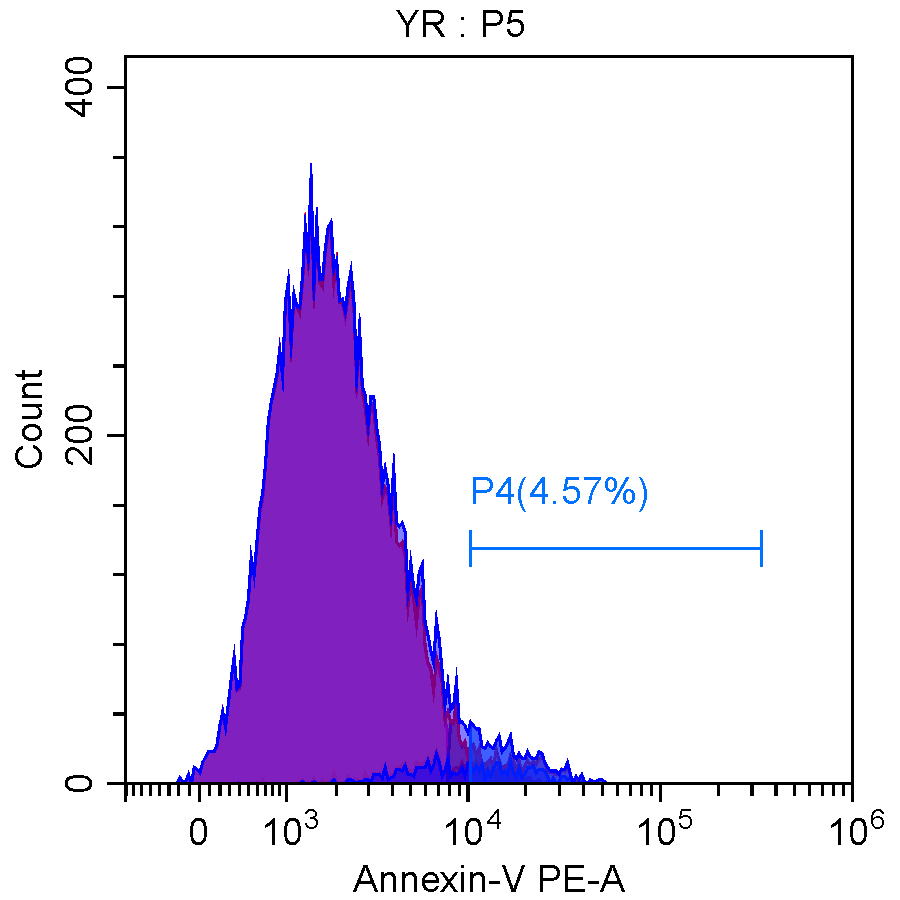

Supplement: Supplementary file 6 [file DataSheet4.ZIP › C/ST/YR _ Plot1.bmp]

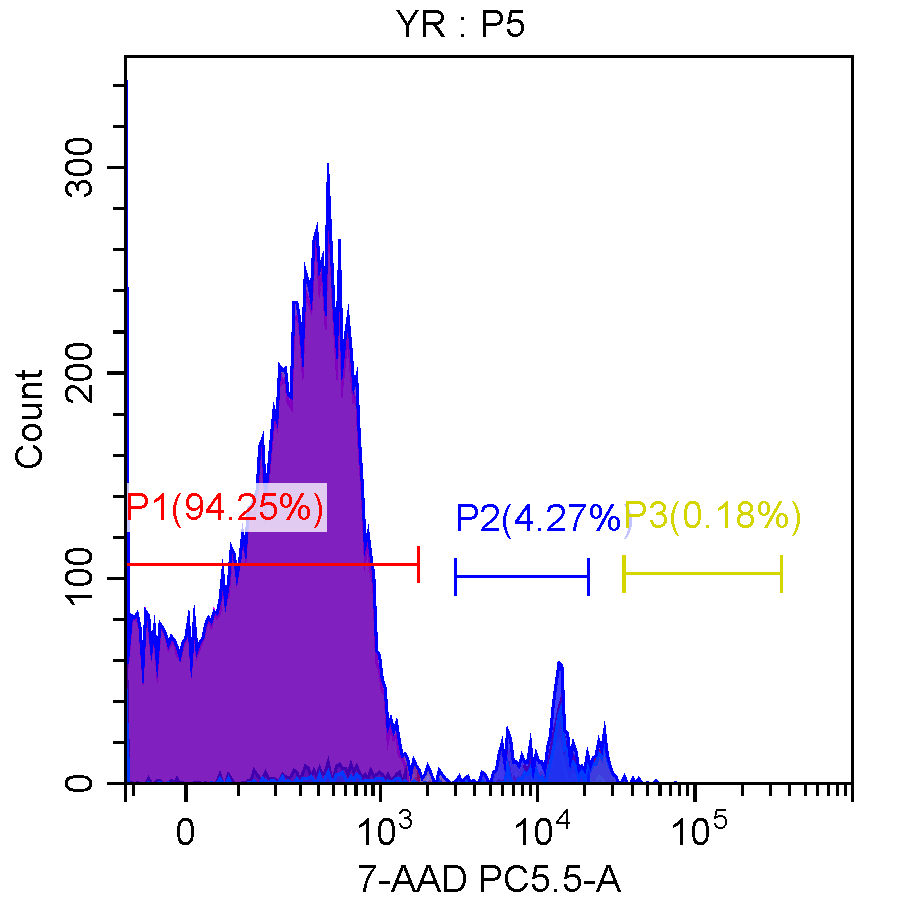

Supplement: Supplementary file 6 [file DataSheet4.ZIP › C/ST/YR_Plot.bmp]

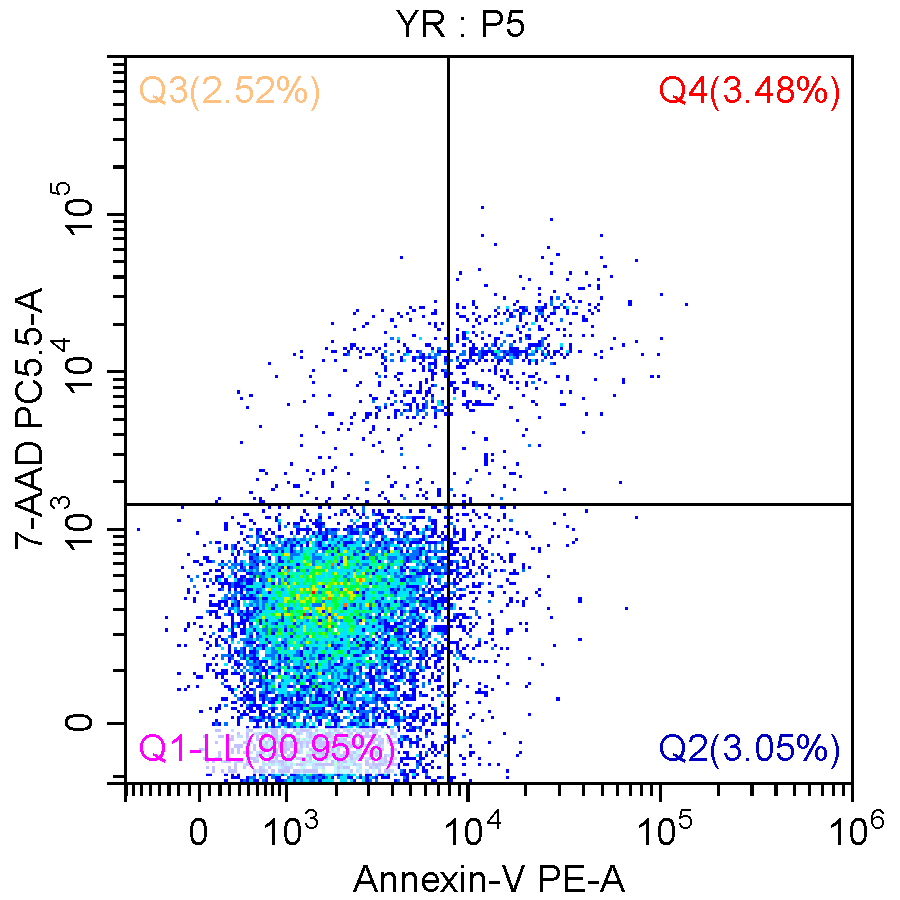

Supplement: Supplementary file 6 [file DataSheet4.ZIP › C/ST/YR_Plot1.bmp]

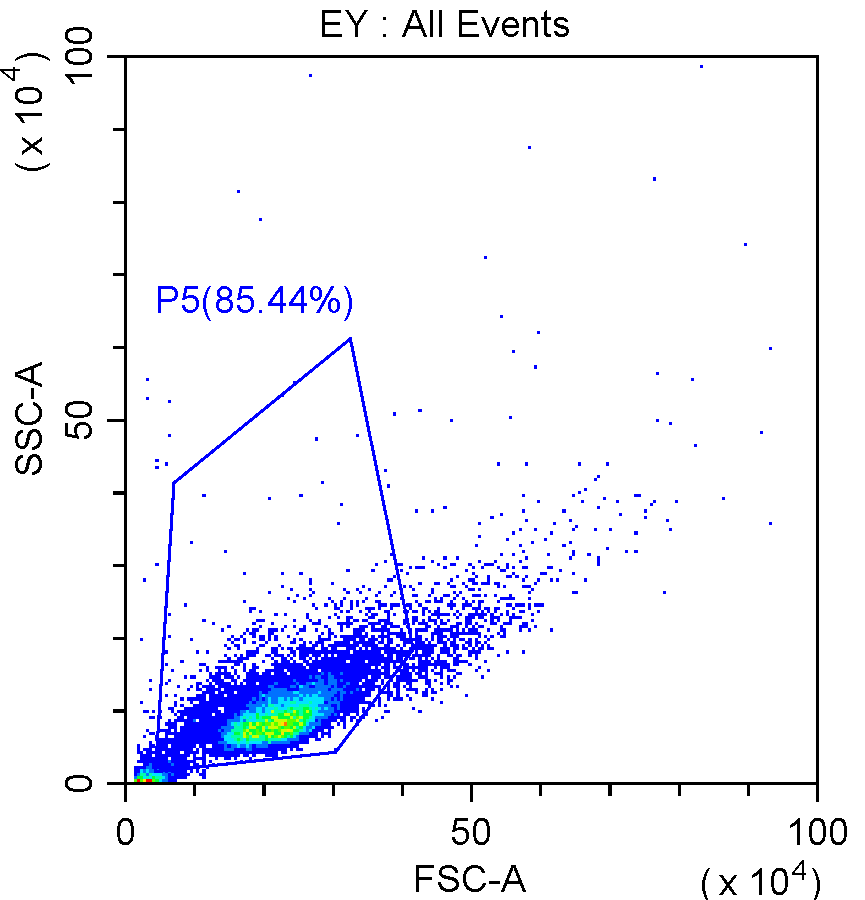

Supplement: Supplementary file 6 [file DataSheet4.ZIP › C/VEHICLE/EY _Plot1.bmp]

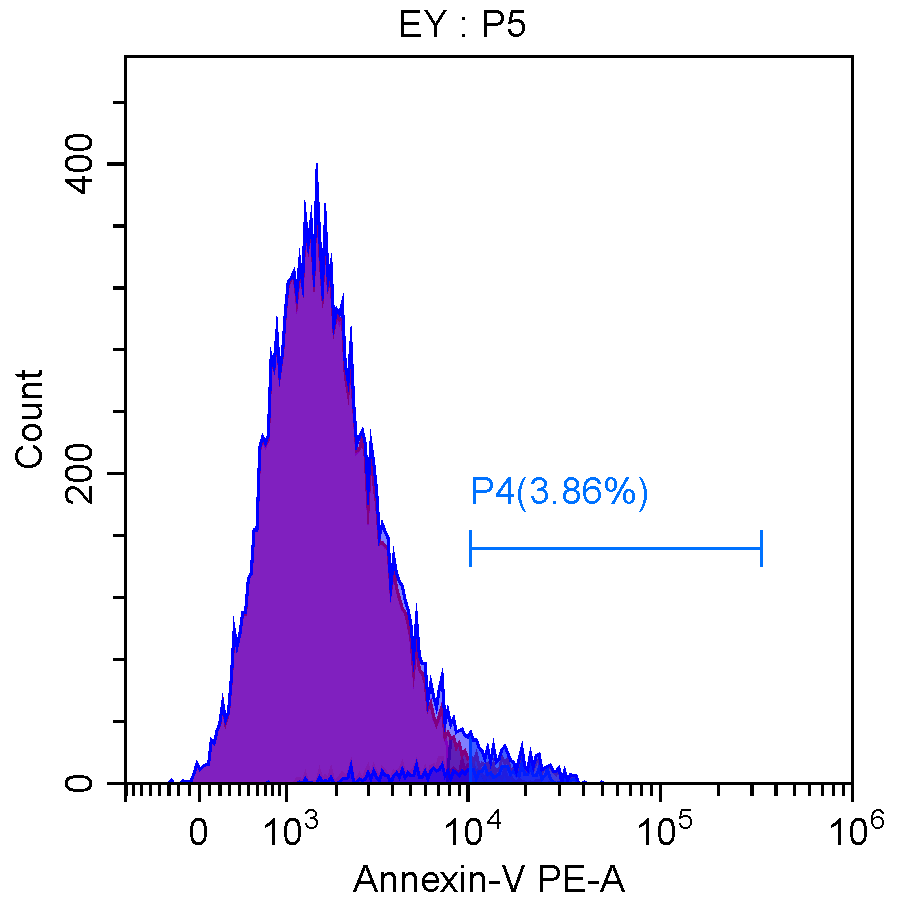

Supplement: Supplementary file 6 [file DataSheet4.ZIP › C/VEHICLE/EY_ Plot1.bmp]

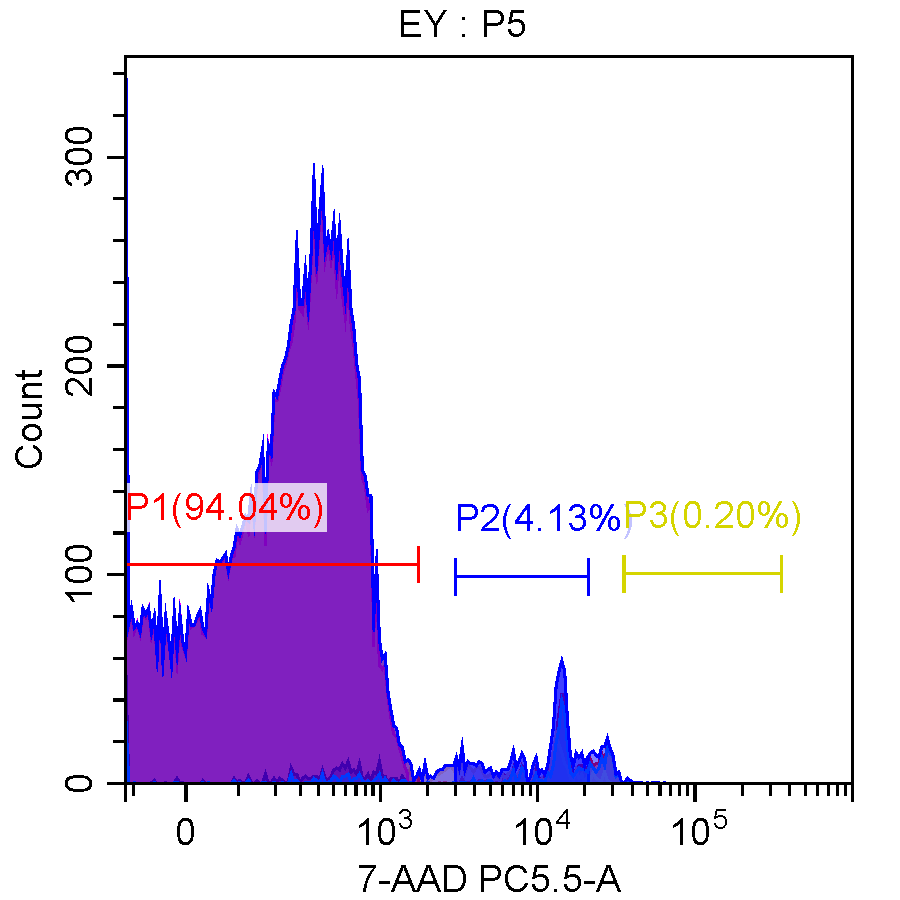

Supplement: Supplementary file 6 [file DataSheet4.ZIP › C/VEHICLE/EY_Plot.bmp]

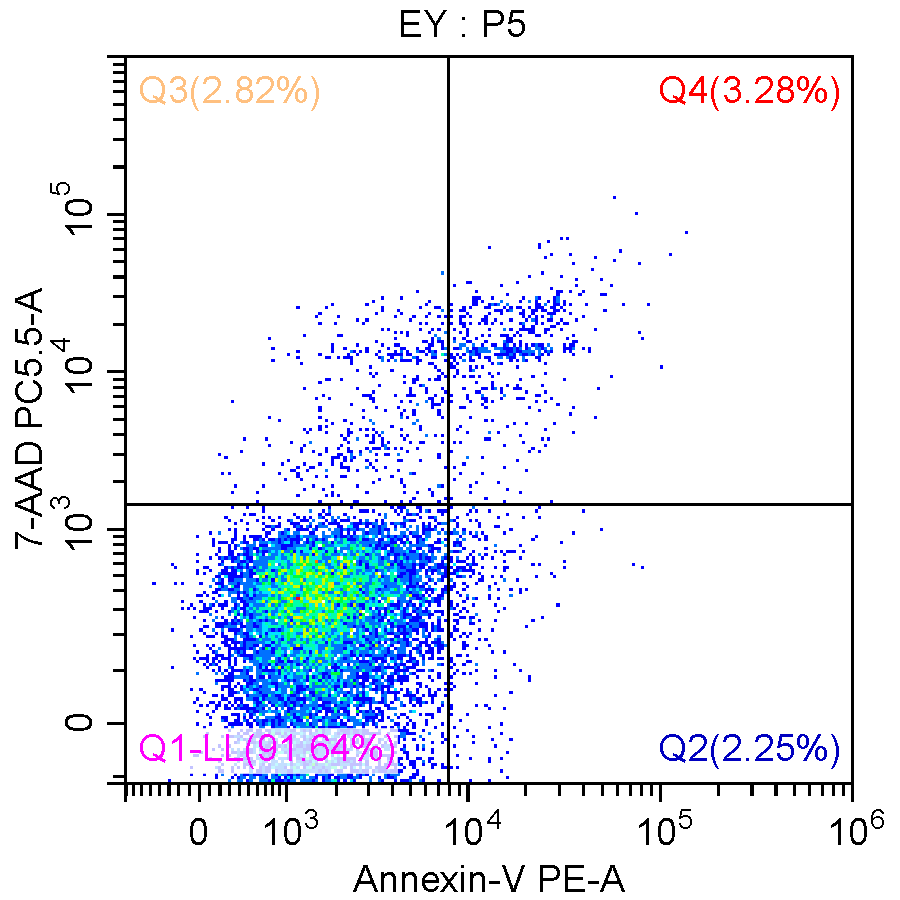

Supplement: Supplementary file 6 [file DataSheet4.ZIP › C/VEHICLE/EY_Plot1.bmp]

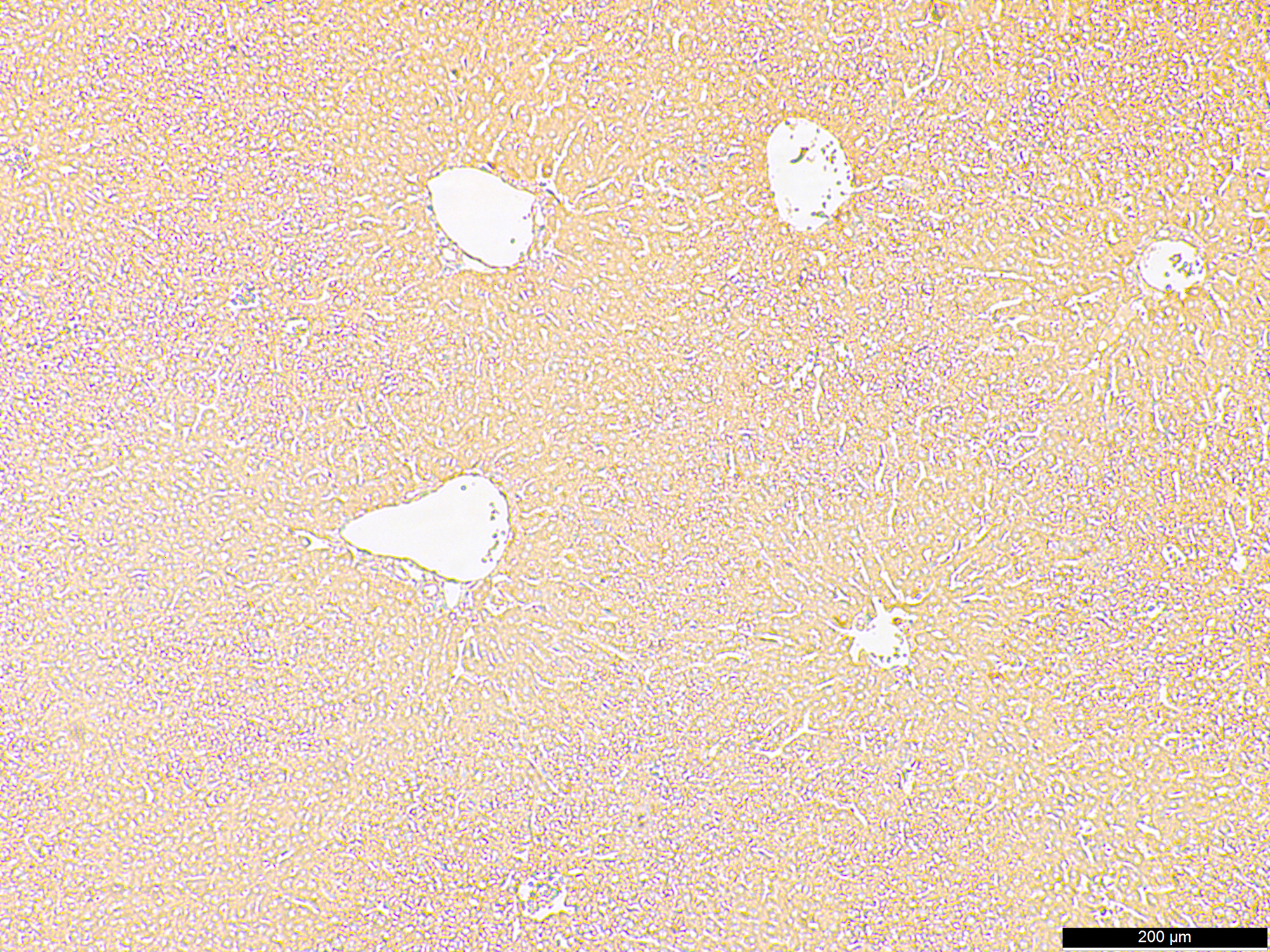

Supplement: Supplementary file 7 [file DataSheet1.ZIP › LPS.tif]

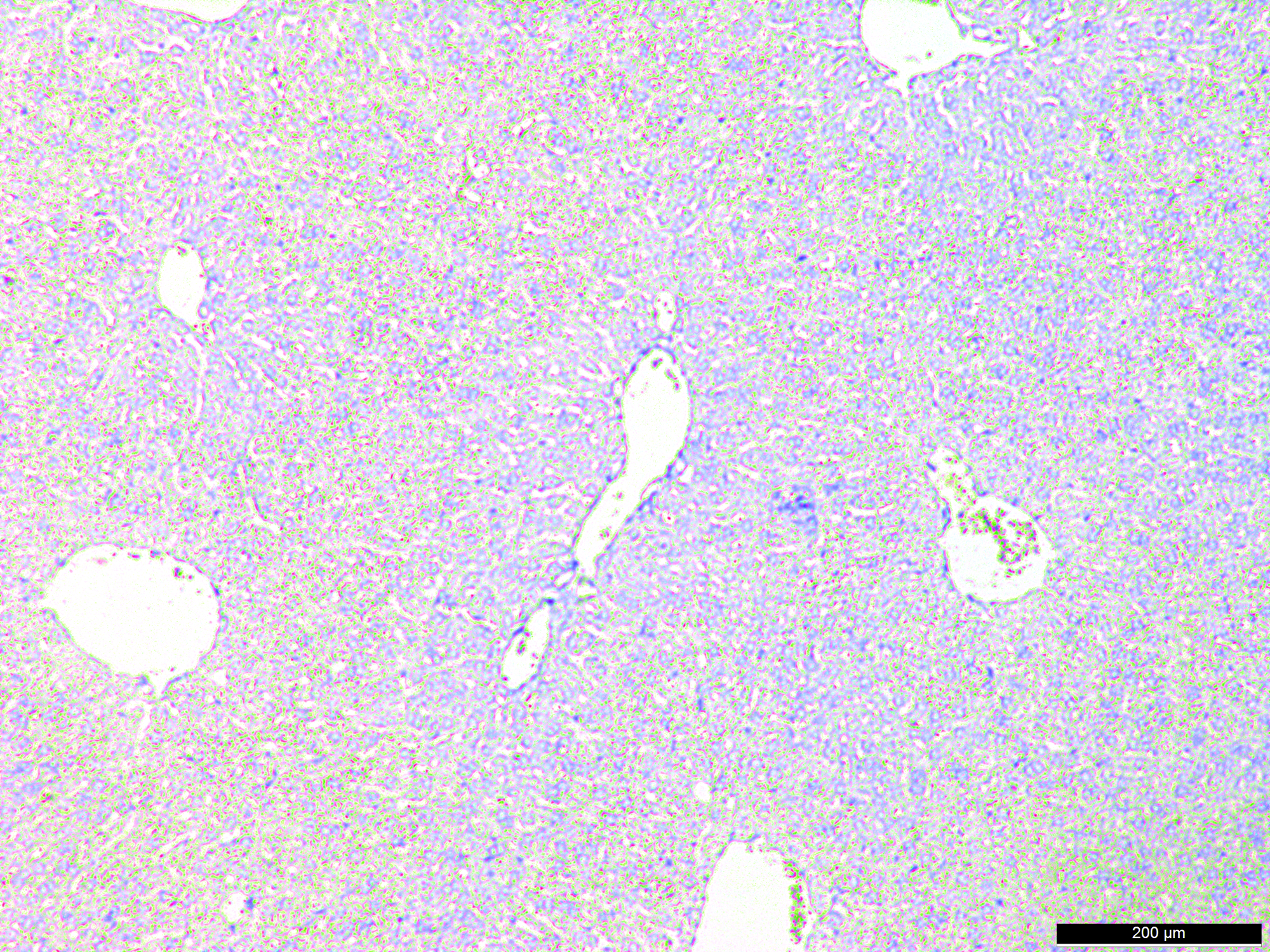

Supplement: Supplementary file 7 [file DataSheet1.ZIP › NC.tif]

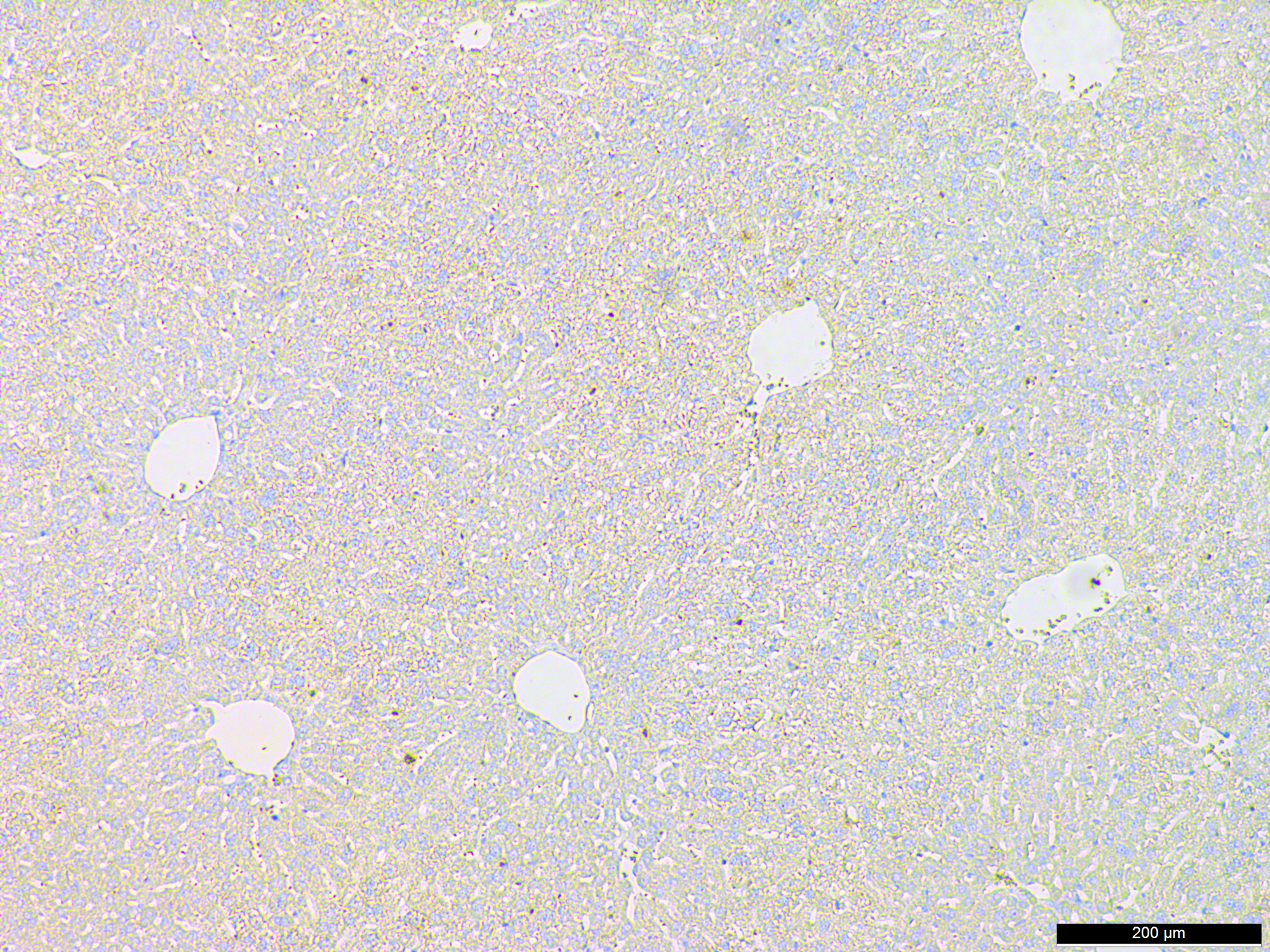

Supplement: Supplementary file 7 [file DataSheet1.ZIP › ST+LPS.tif]

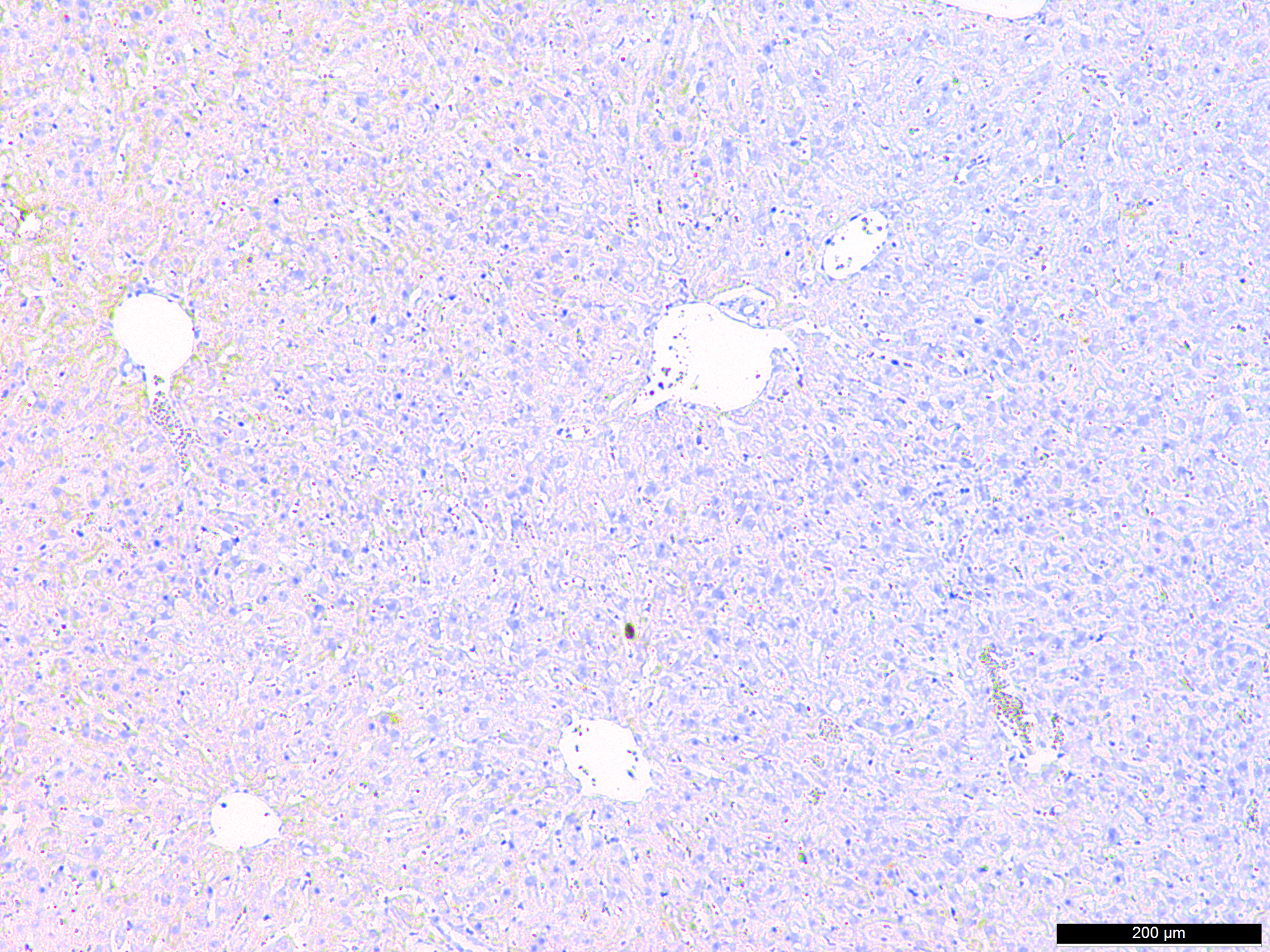

Supplement: Supplementary file 7 [file DataSheet1.ZIP › ST.tif]

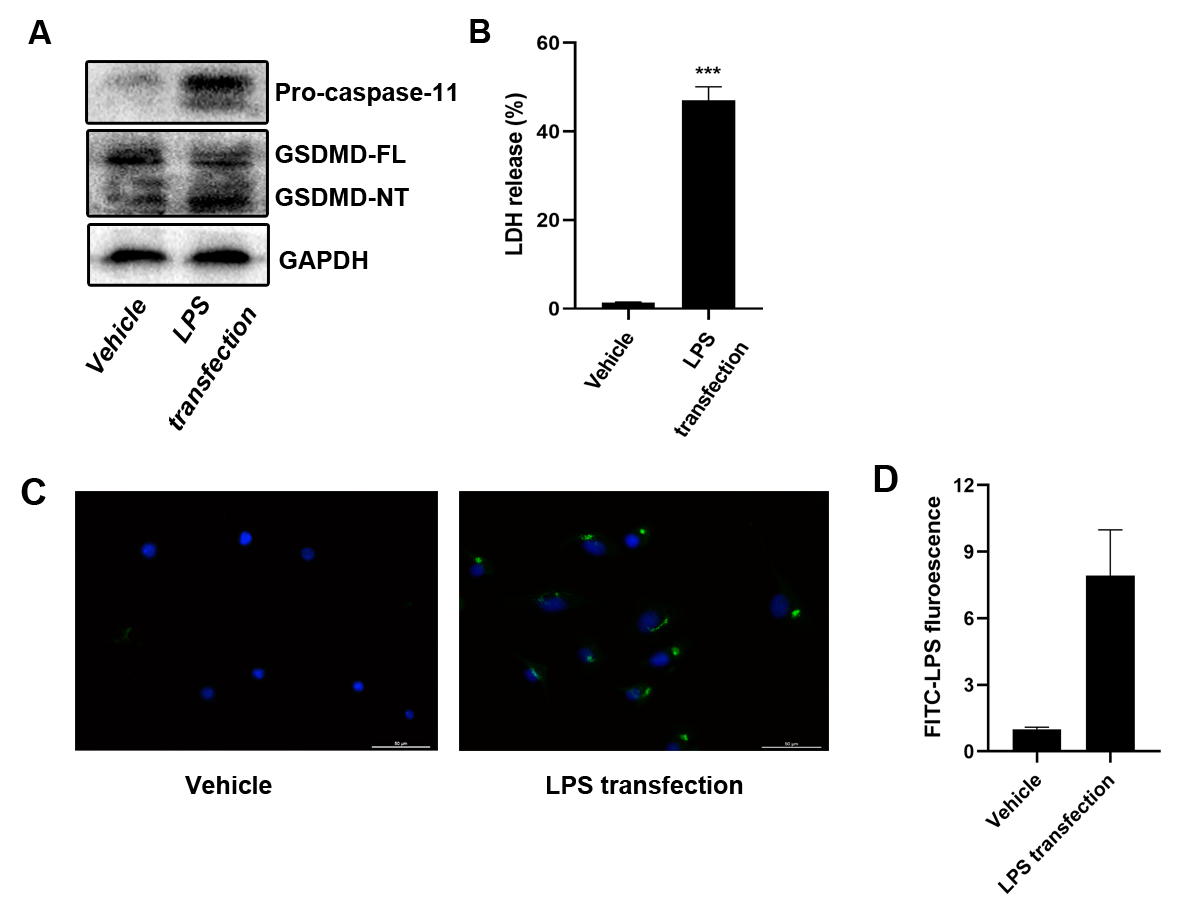

Supplement: Supplementary file 8 [file Image2.TIF]

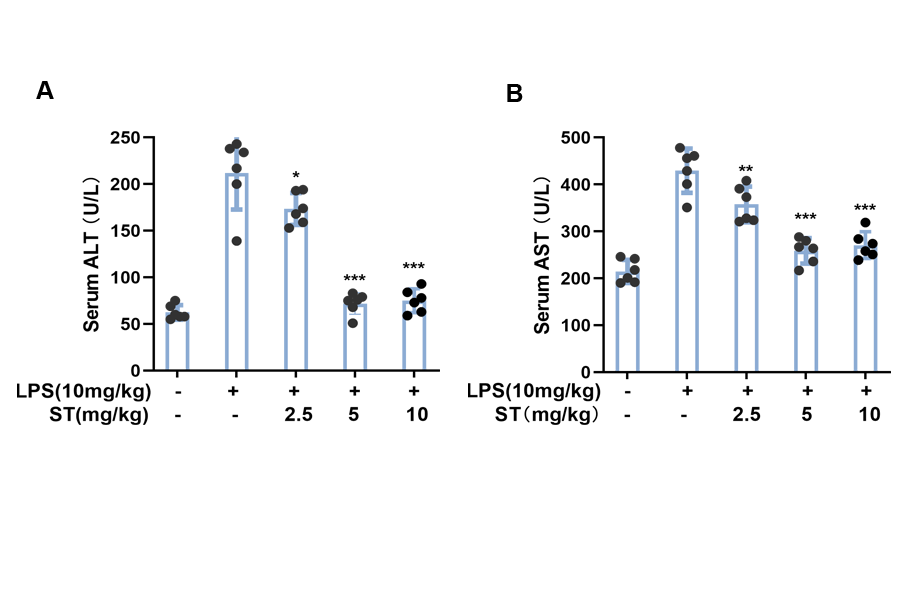

Supplement: Supplementary file 9 [file Image1.TIF]

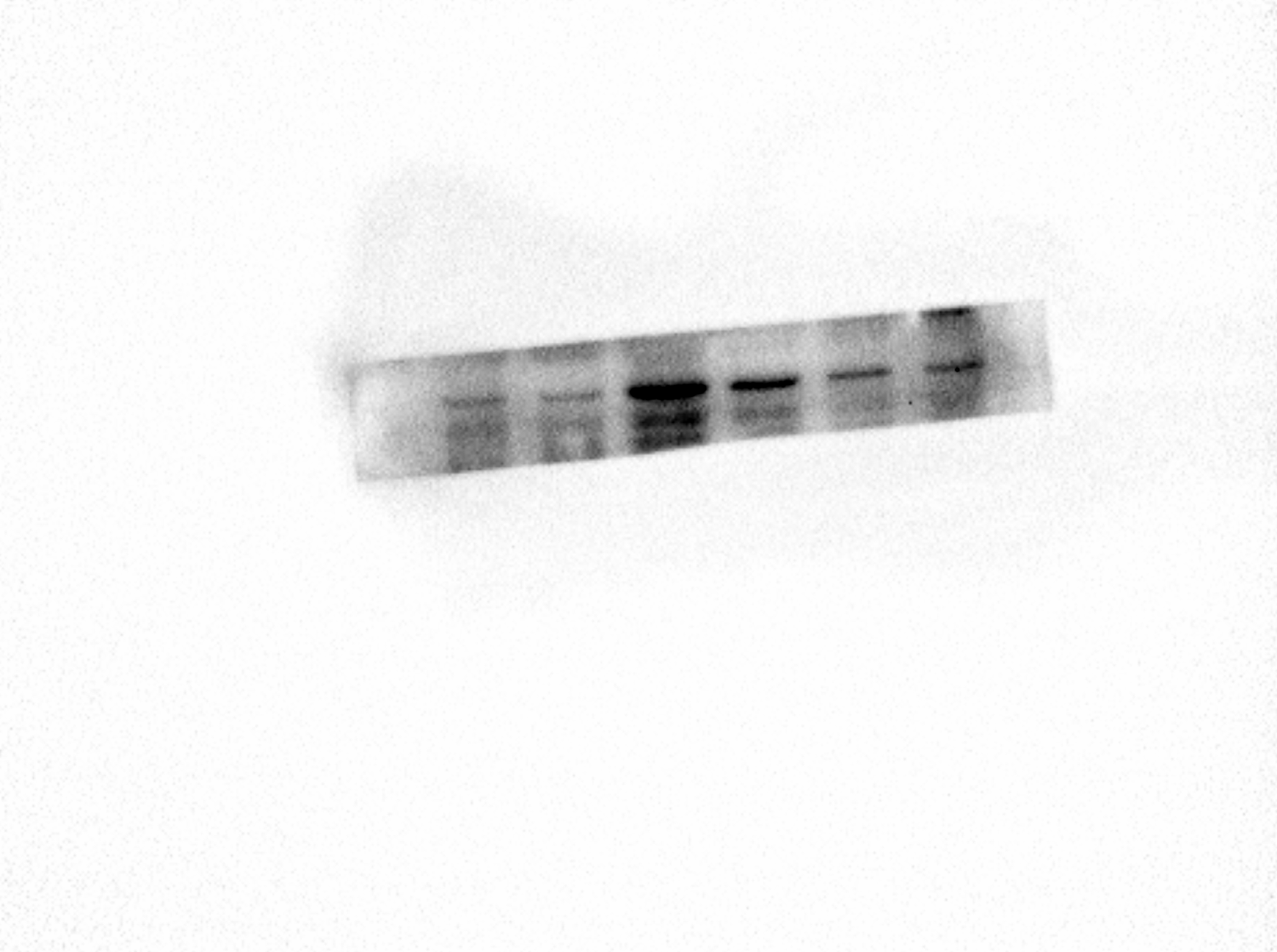

Supplement: Supplementary file 10 [file DataSheet10.ZIP › F1/CAS11.tif]

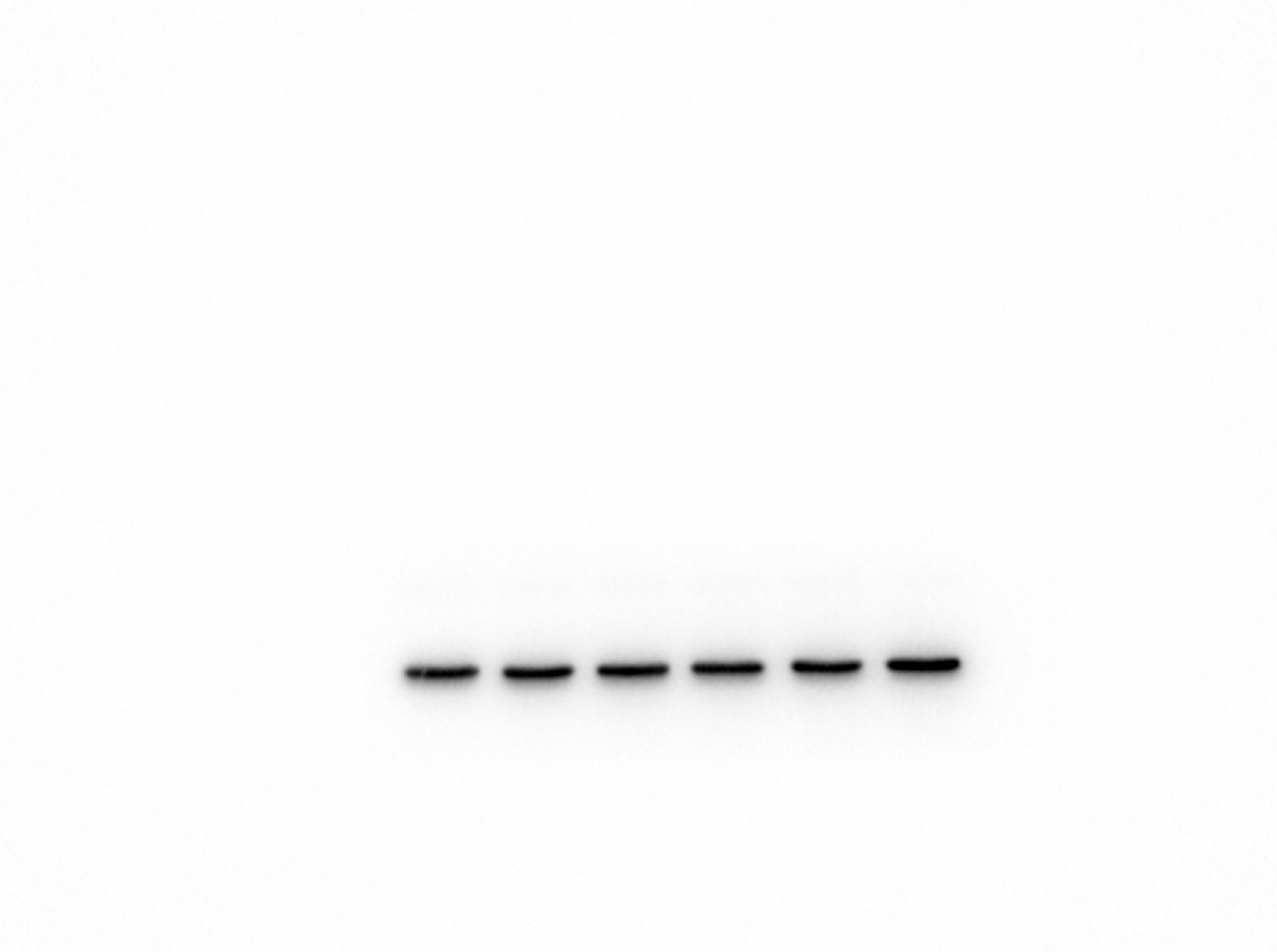

Supplement: Supplementary file 10 [file DataSheet10.ZIP › F1/GAPDH.tif]

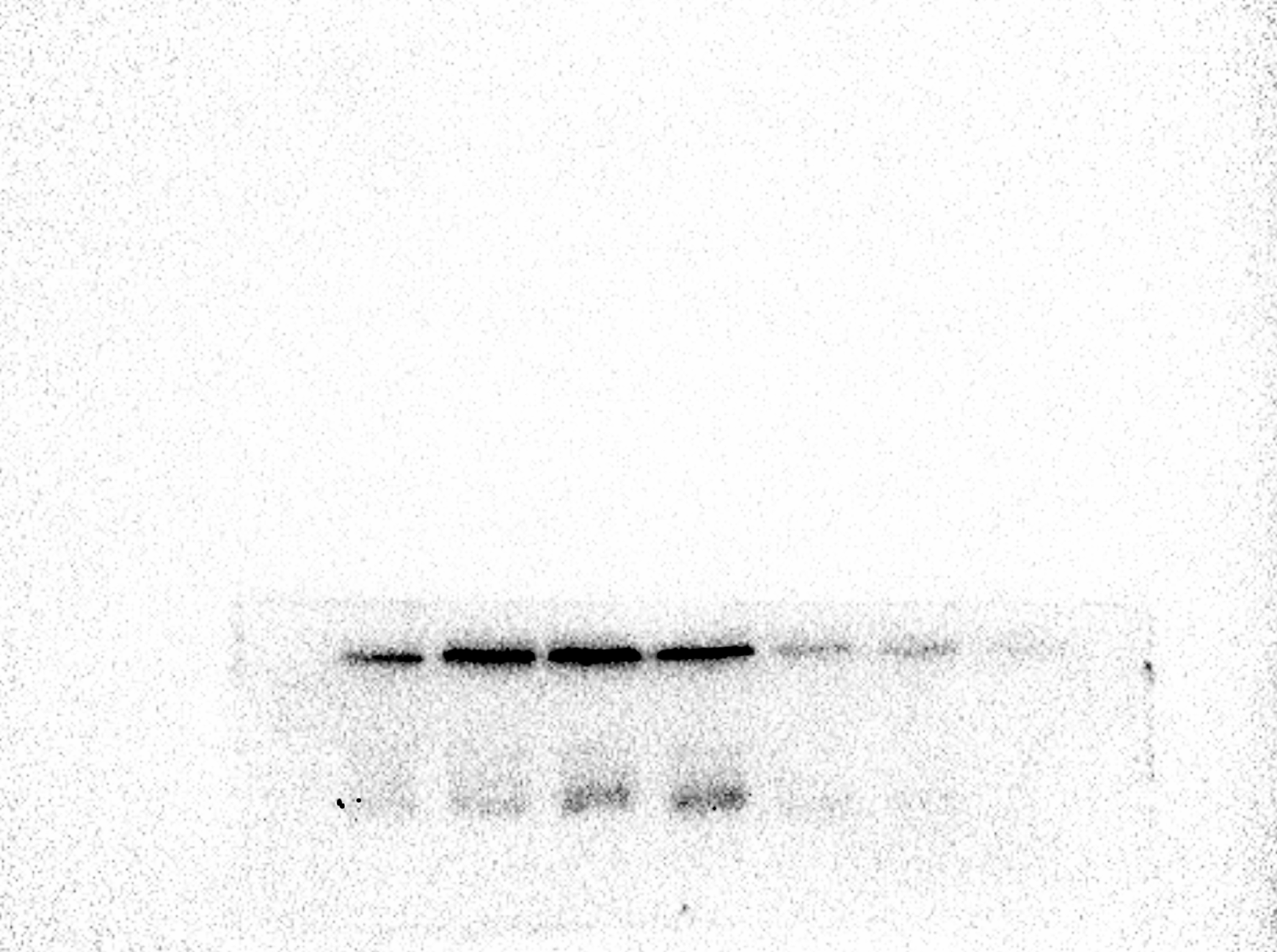

Supplement: Supplementary file 10 [file DataSheet10.ZIP › F1/GSDMD.tif]

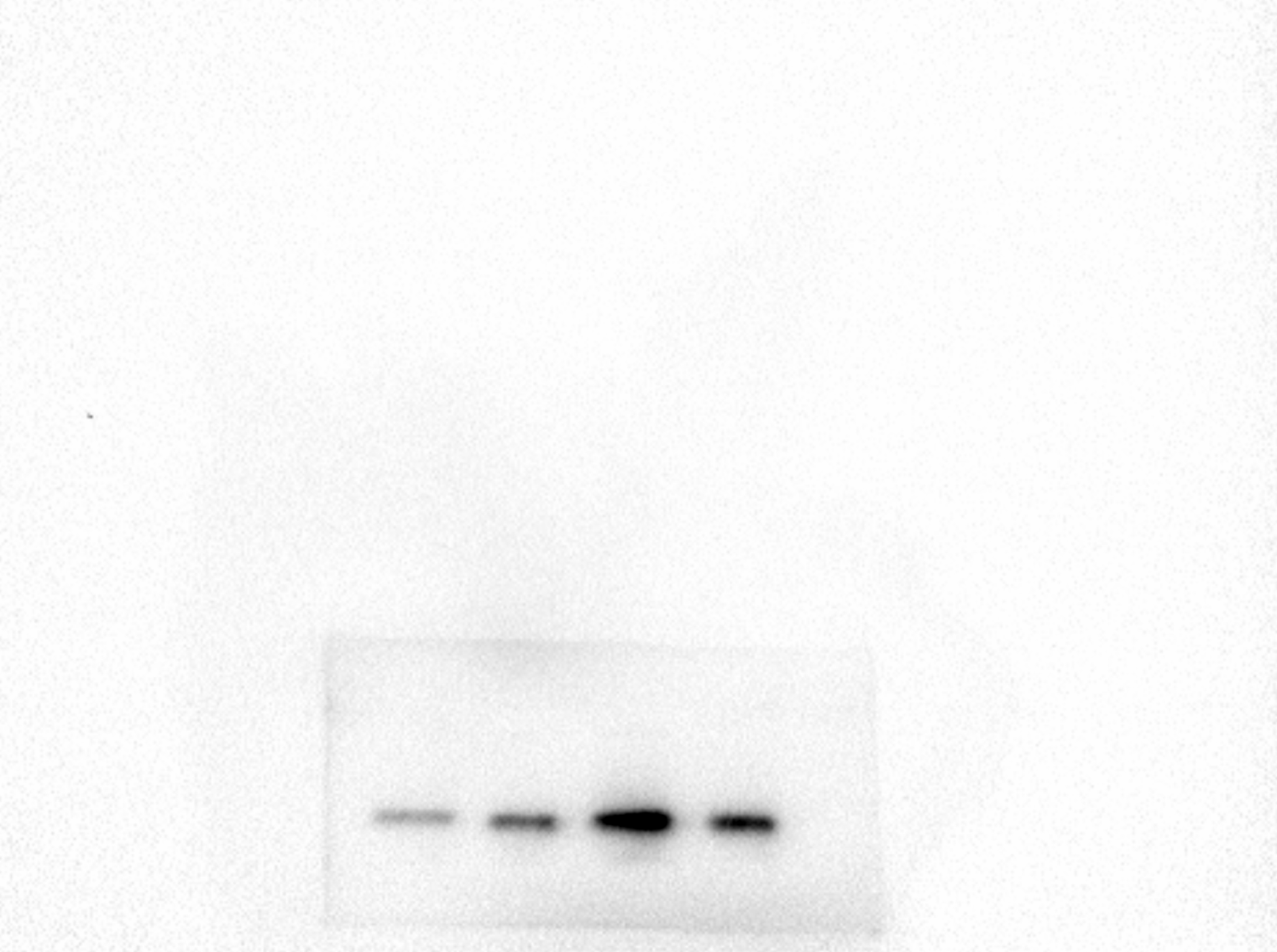

Supplement: Supplementary file 10 [file DataSheet10.ZIP › F4/ASC.tif]
