# Supplementary figures and images for: Samotolisib Attenuates Acute Liver Injury Through Inhibiting Caspase-11-Mediated Pyroptosis Via Regulating E3 Ubiquitin Ligase Nedd4 (part 2 of 2)
Source: Front Pharmacol. 2021 Aug 13;12:726198. doi: 10.3389/fphar.2021.726198 (PMC8414251; doi:10.3389/fphar.2021.726198)

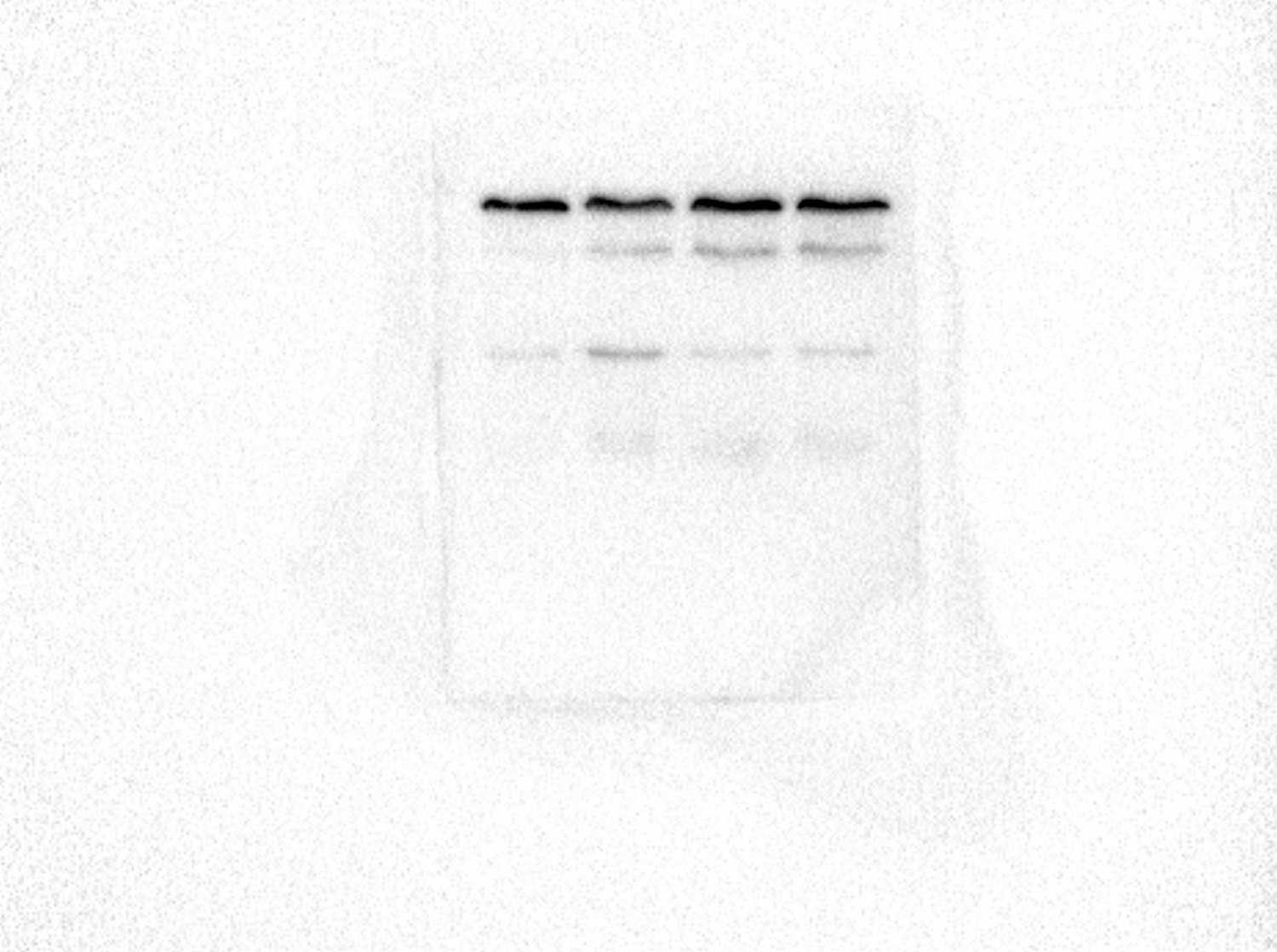

Supplement: Supplementary file 10 [file DataSheet10.ZIP › F4/C1.tif]

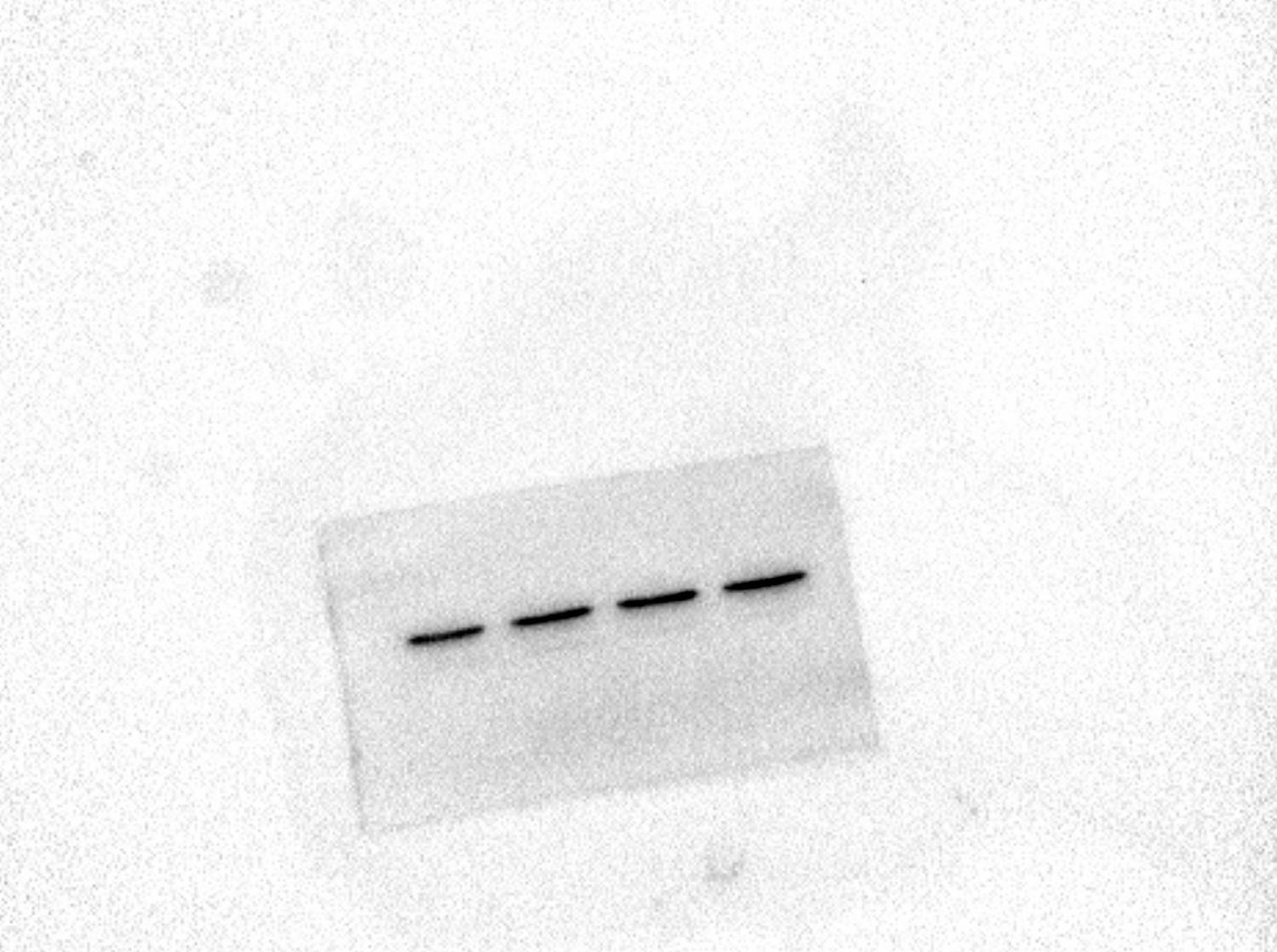

Supplement: Supplementary file 10 [file DataSheet10.ZIP › F4/F4 GAPDH.tif]

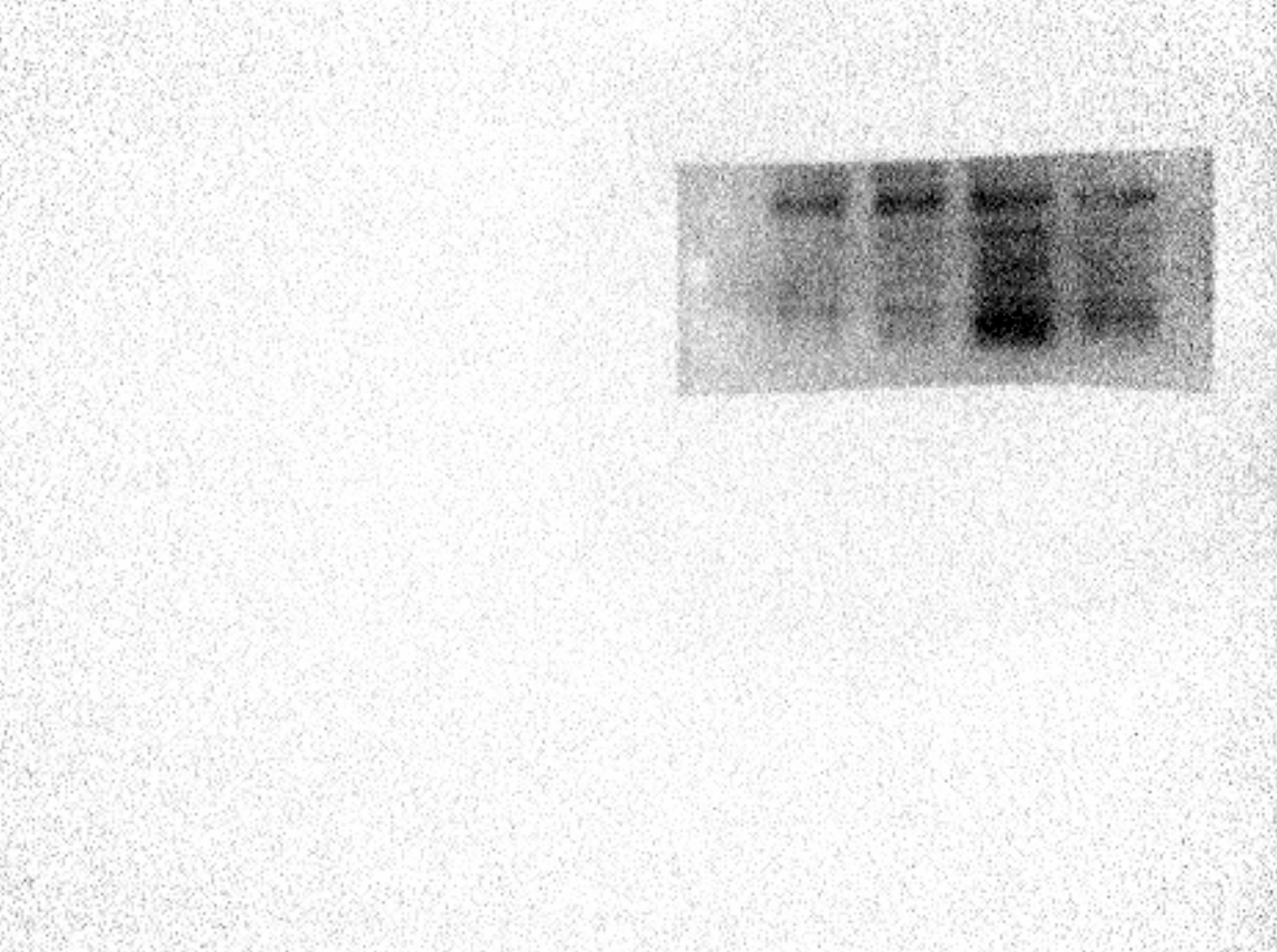

Supplement: Supplementary file 10 [file DataSheet10.ZIP › F4/GSDMD.tif]

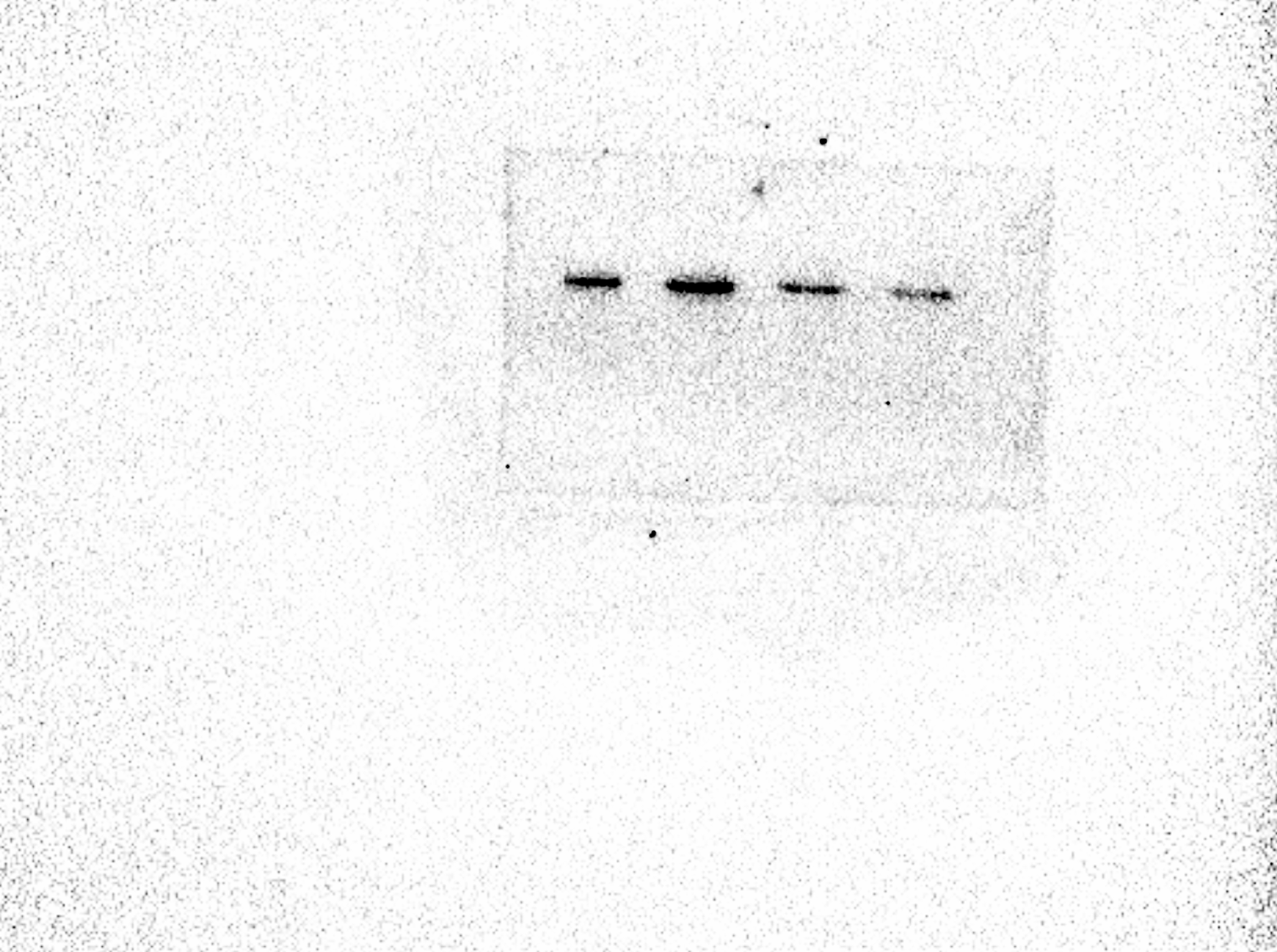

Supplement: Supplementary file 10 [file DataSheet10.ZIP › F4/IL Ia┬.tif]

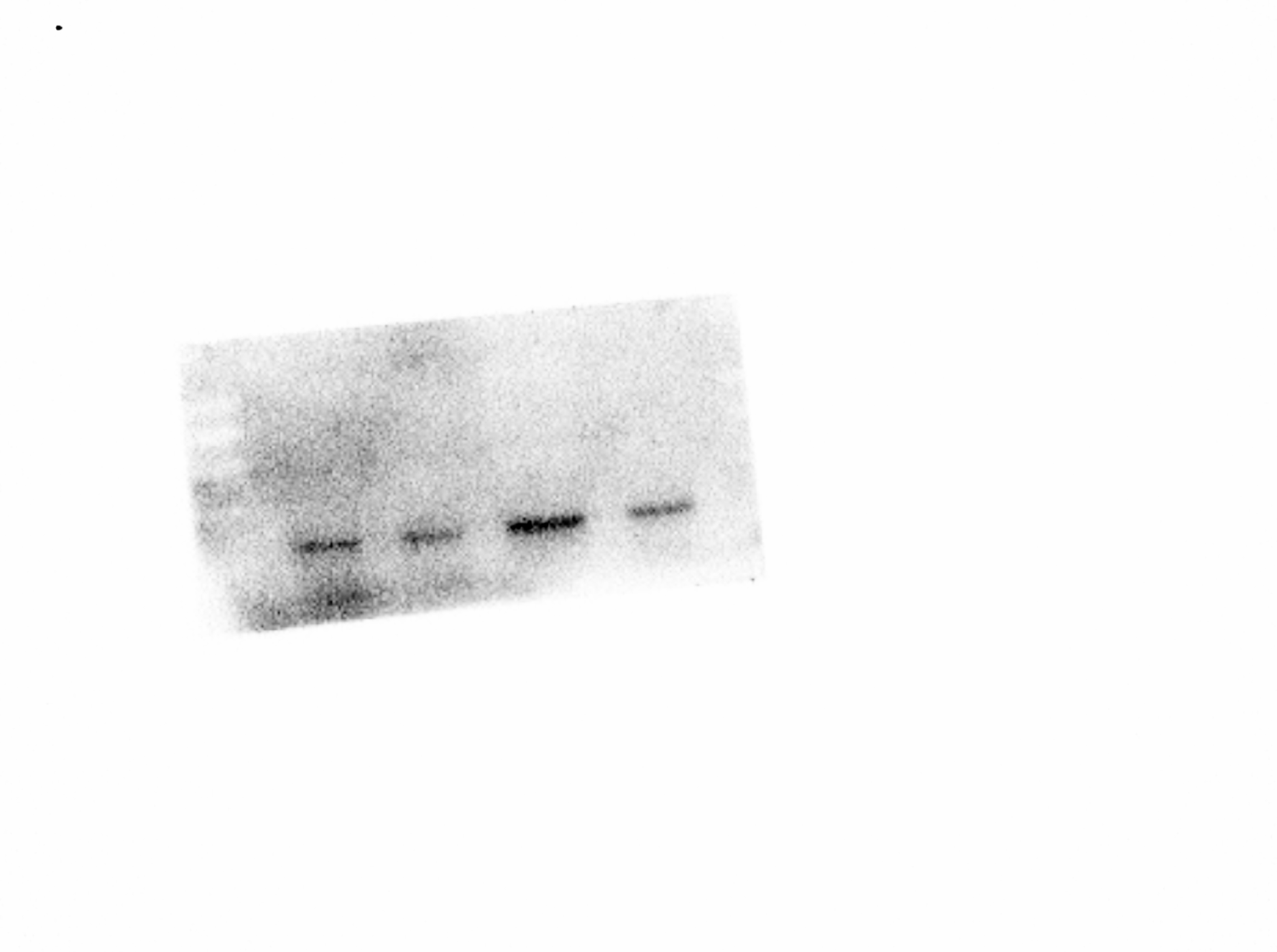

Supplement: Supplementary file 10 [file DataSheet10.ZIP › F4/IL-18.tif]

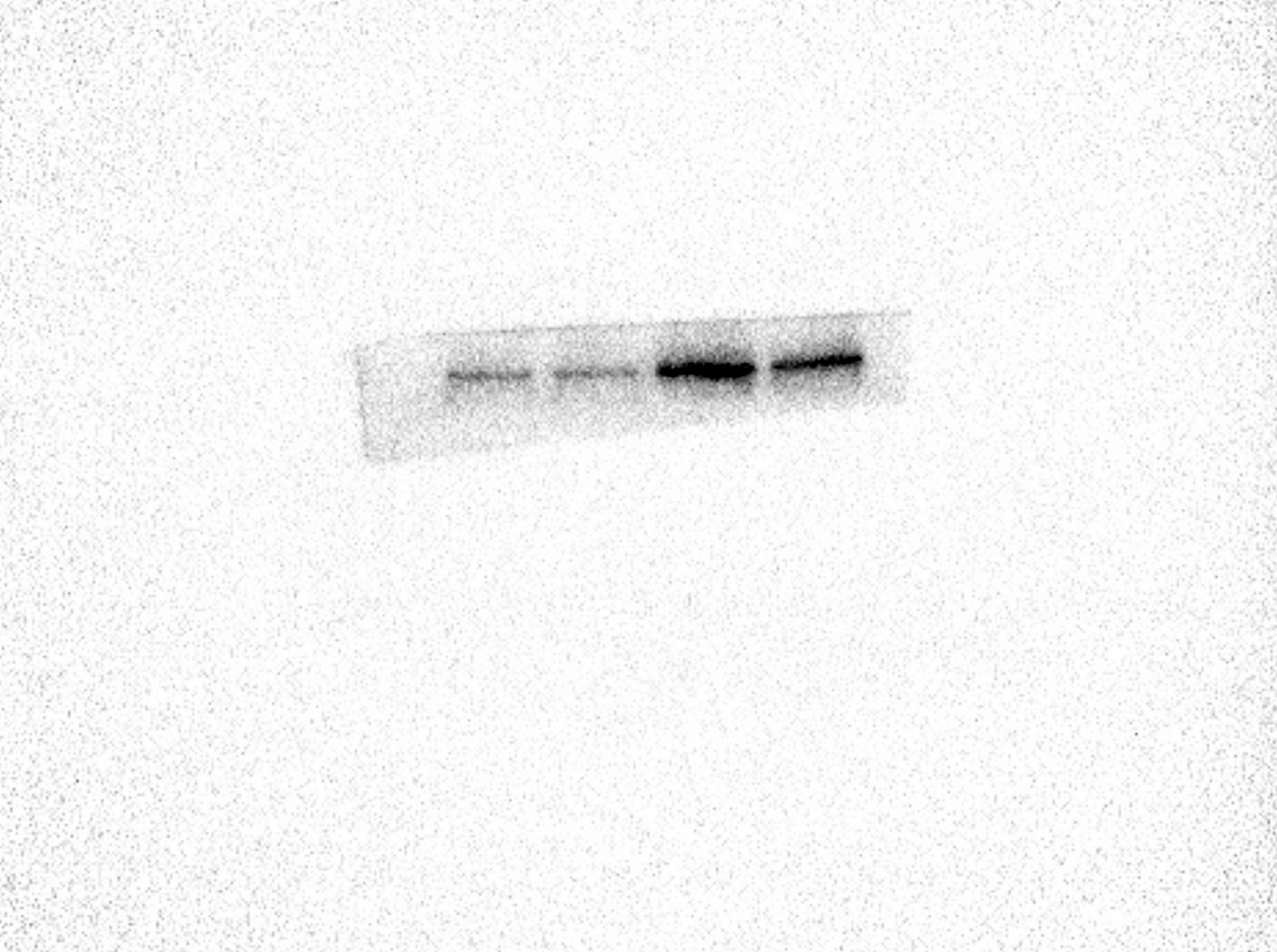

Supplement: Supplementary file 10 [file DataSheet10.ZIP › F4/NLRP3.tif]

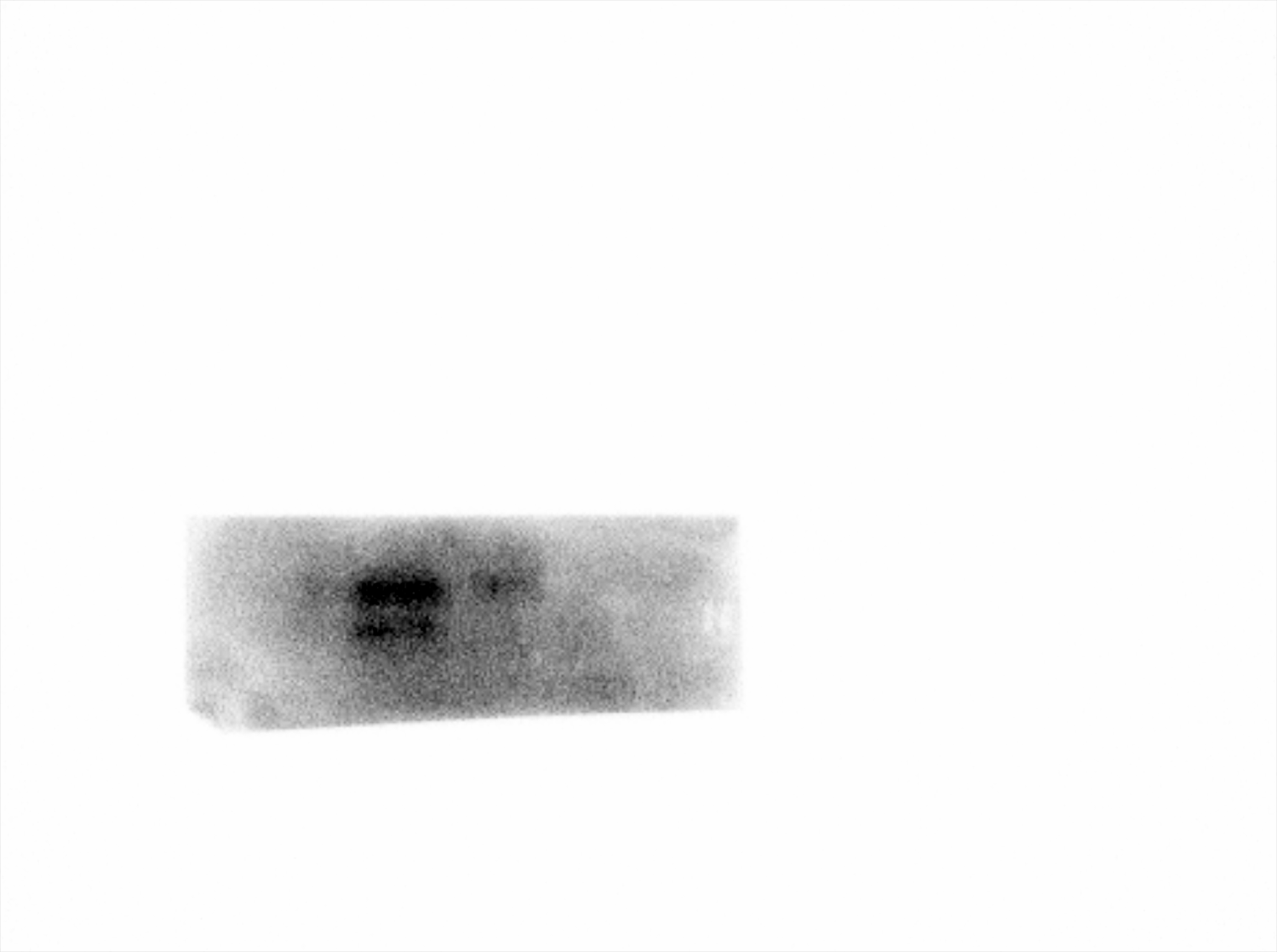

Supplement: Supplementary file 10 [file DataSheet10.ZIP › F5 CO-IP/C11 B.tif]

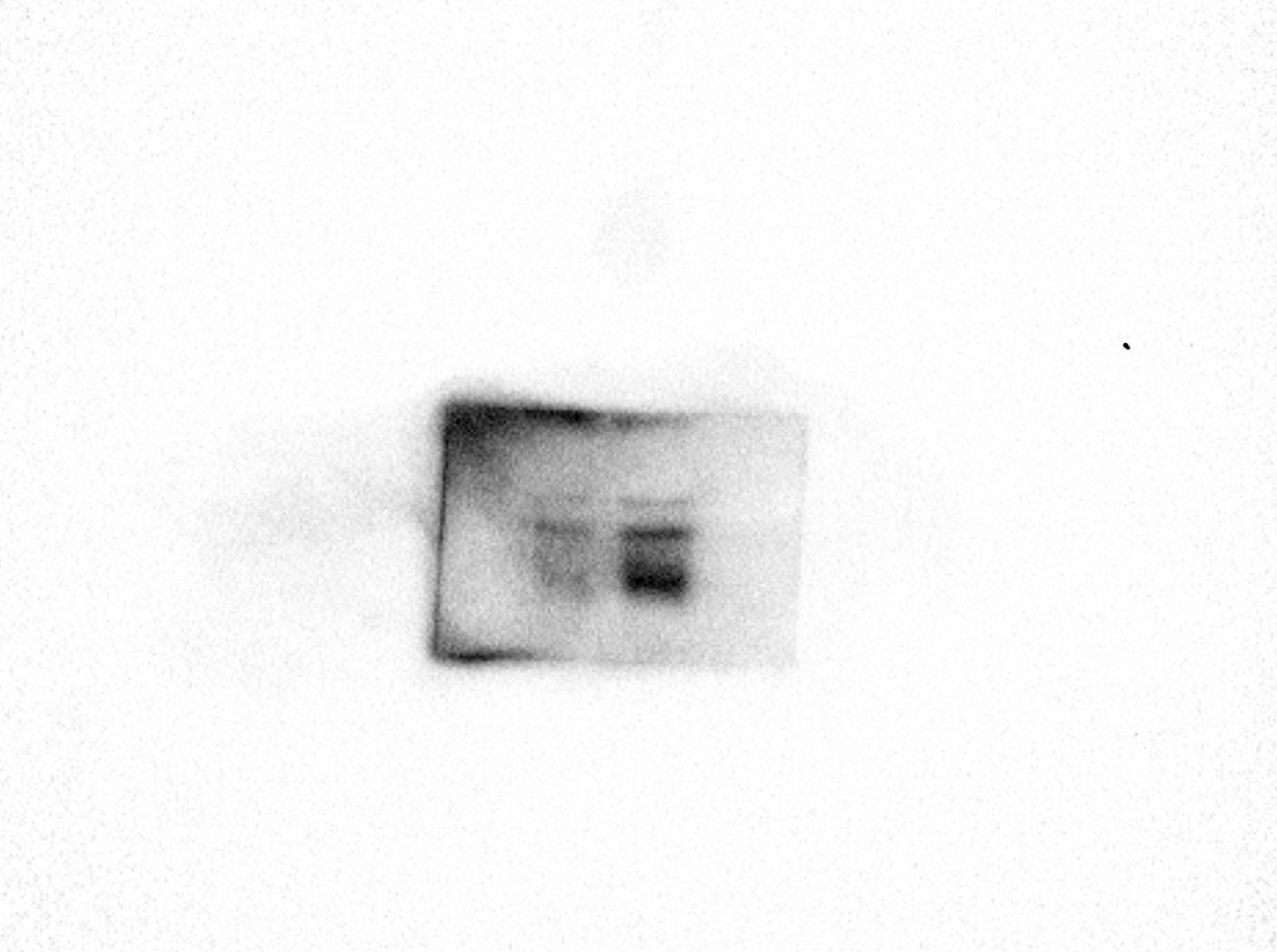

Supplement: Supplementary file 10 [file DataSheet10.ZIP › F5 CO-IP/c11 c.tif]

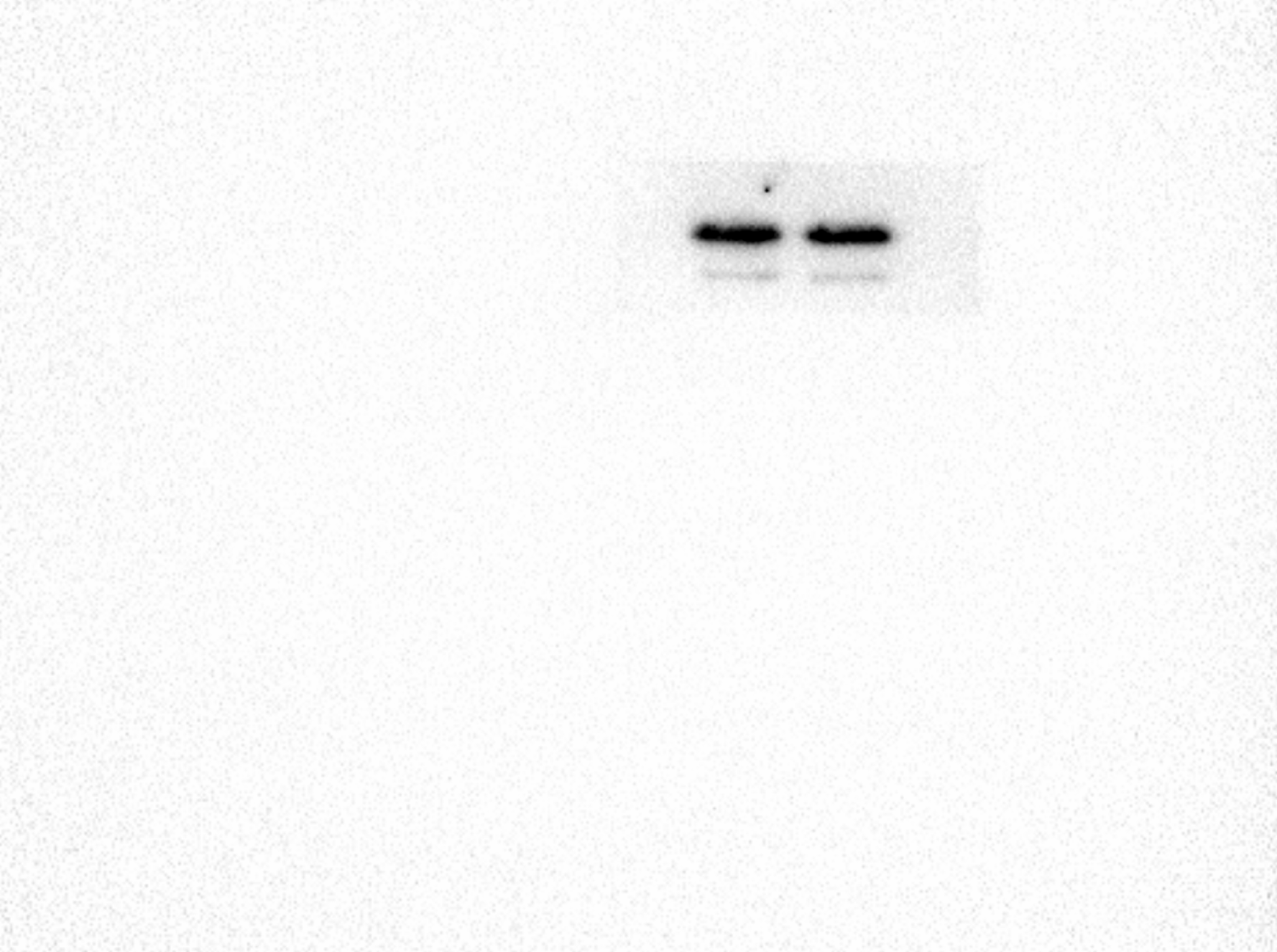

Supplement: Supplementary file 10 [file DataSheet10.ZIP › F5 CO-IP/GAPDH B.tif]

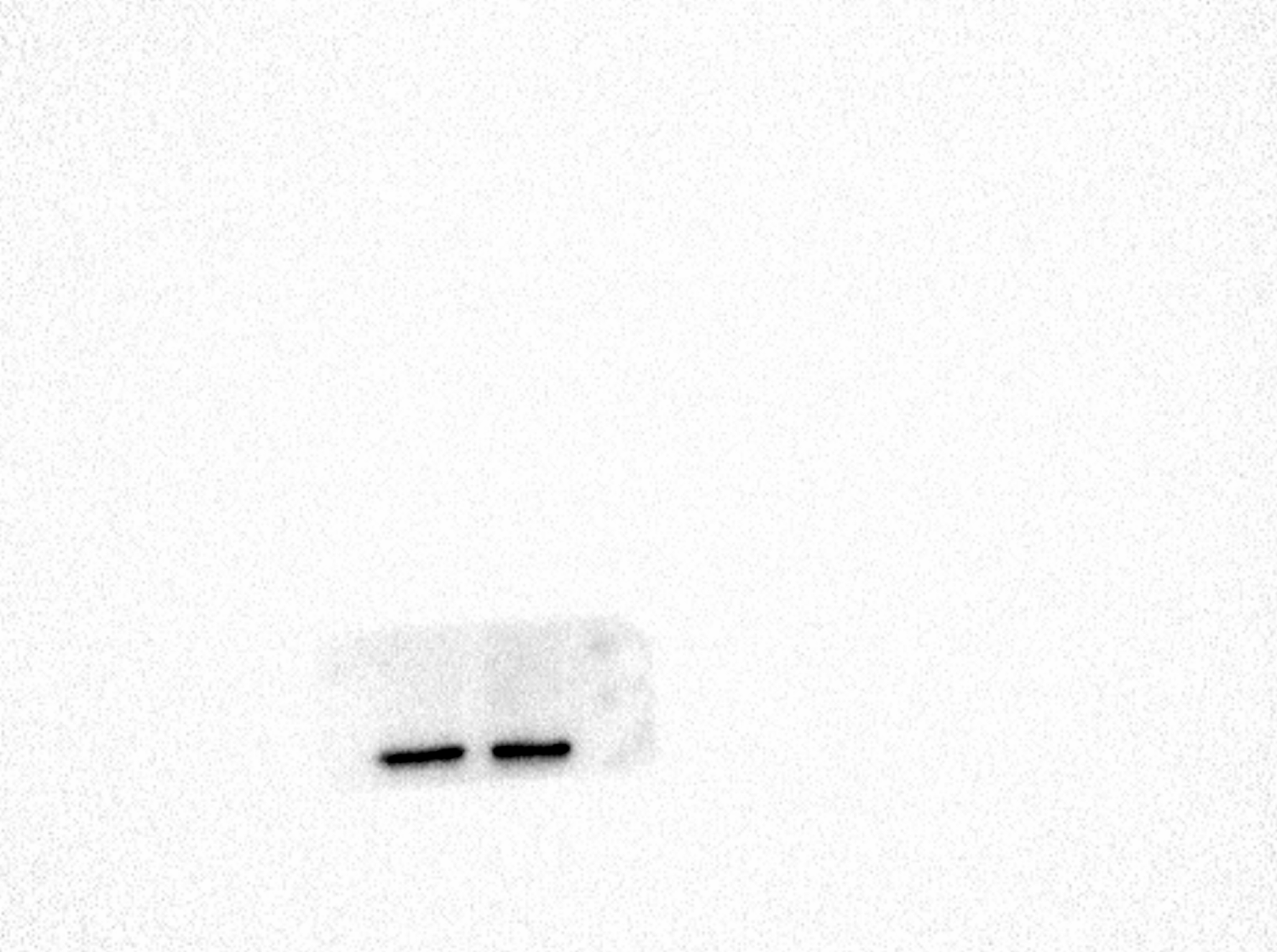

Supplement: Supplementary file 10 [file DataSheet10.ZIP › F5 CO-IP/GAPDH C.tif]

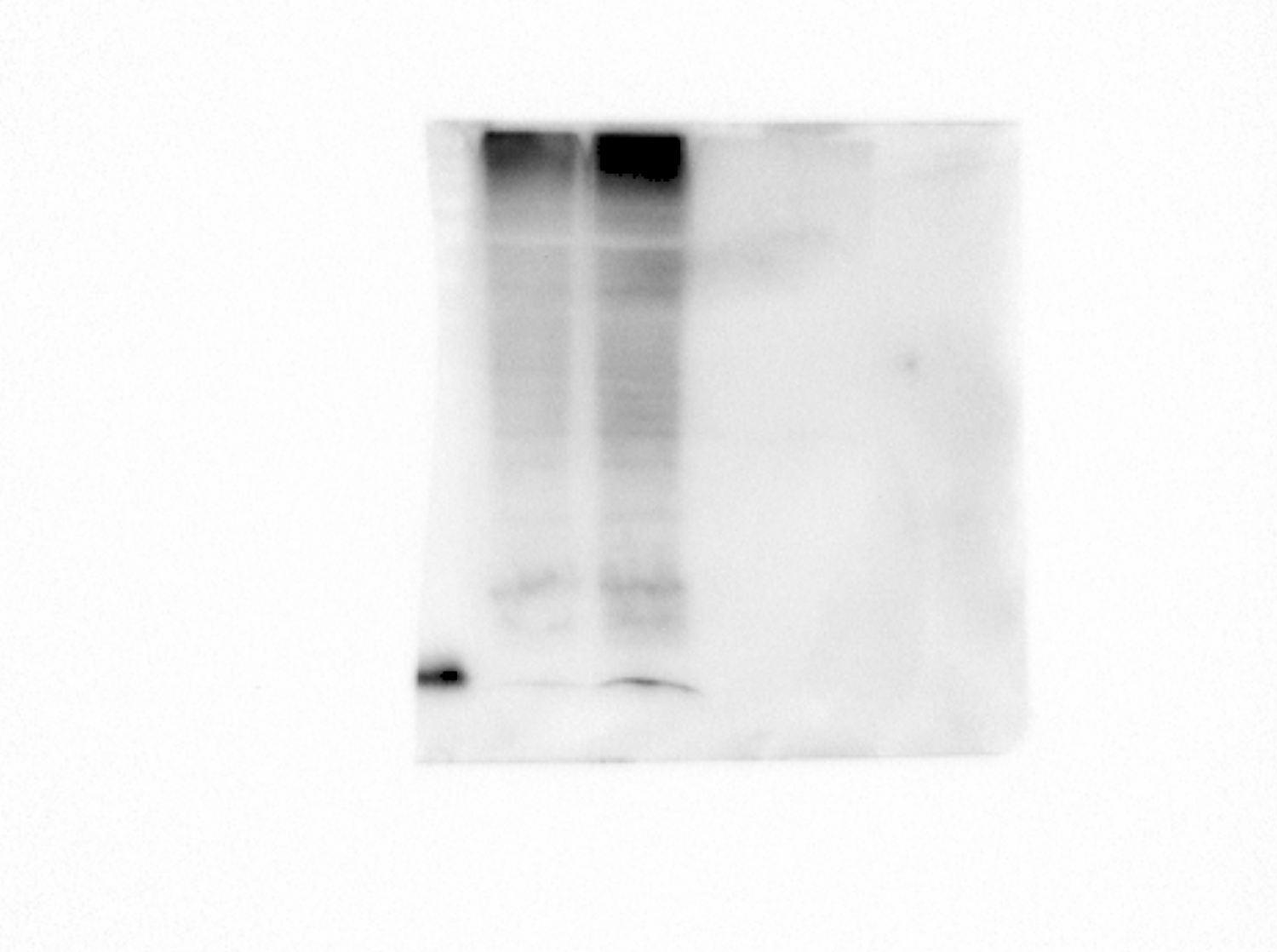

Supplement: Supplementary file 10 [file DataSheet10.ZIP › F5 CO-IP/UB B.tif]

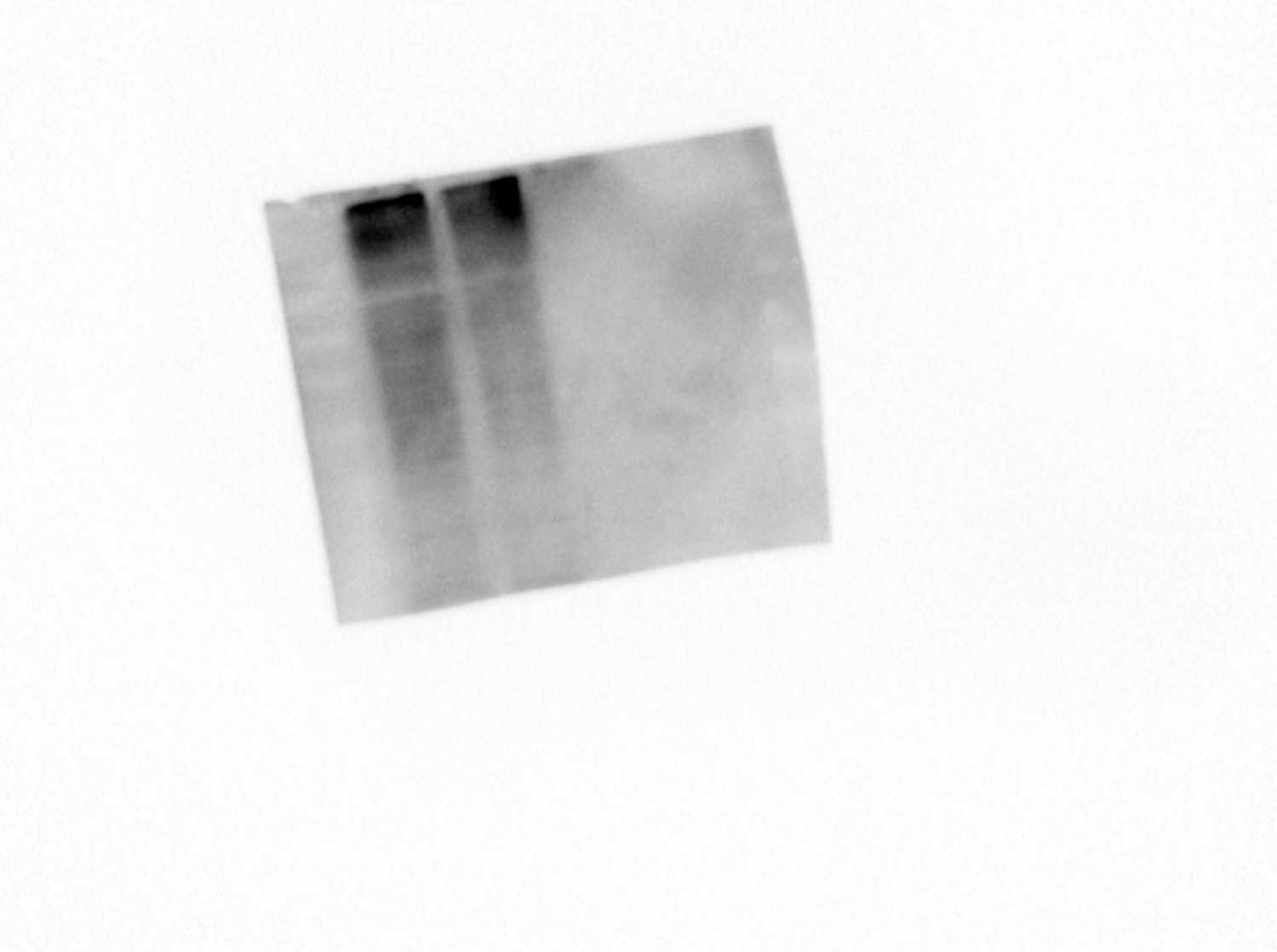

Supplement: Supplementary file 10 [file DataSheet10.ZIP › F5 CO-IP/UB C.tif]

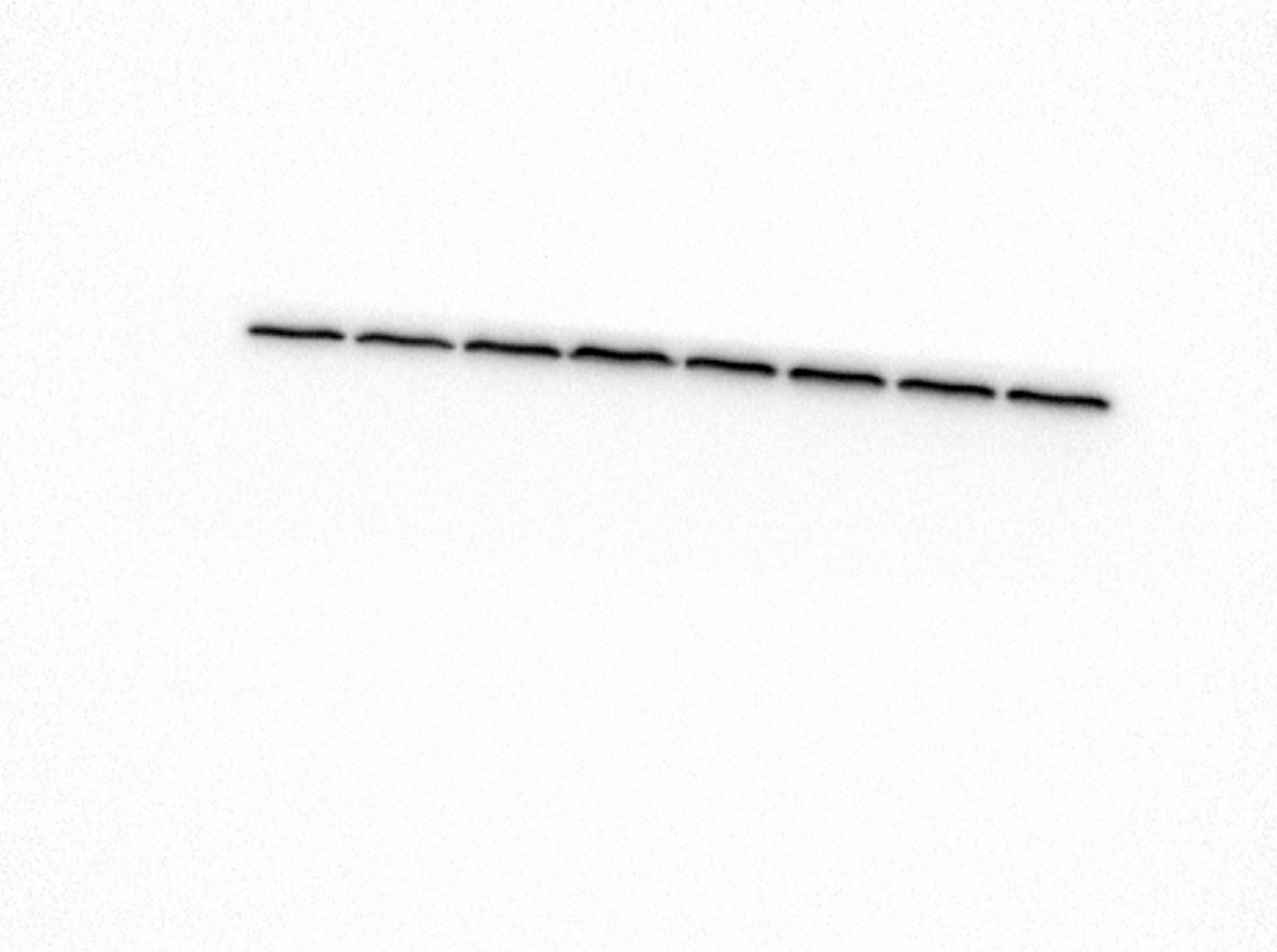

Supplement: Supplementary file 10 [file DataSheet10.ZIP › F5/AKT.tif]

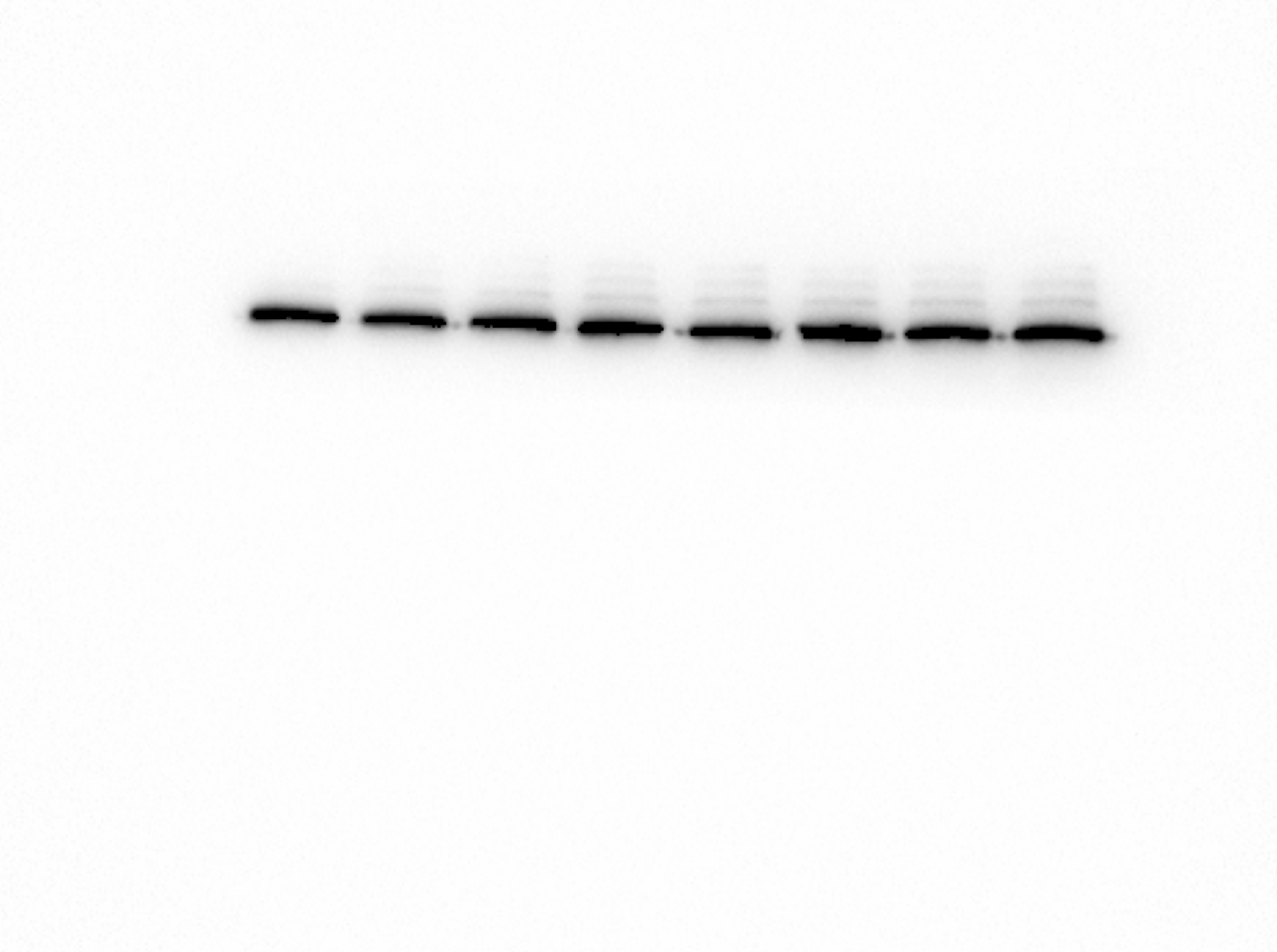

Supplement: Supplementary file 10 [file DataSheet10.ZIP › F5/GAPDH.tif]

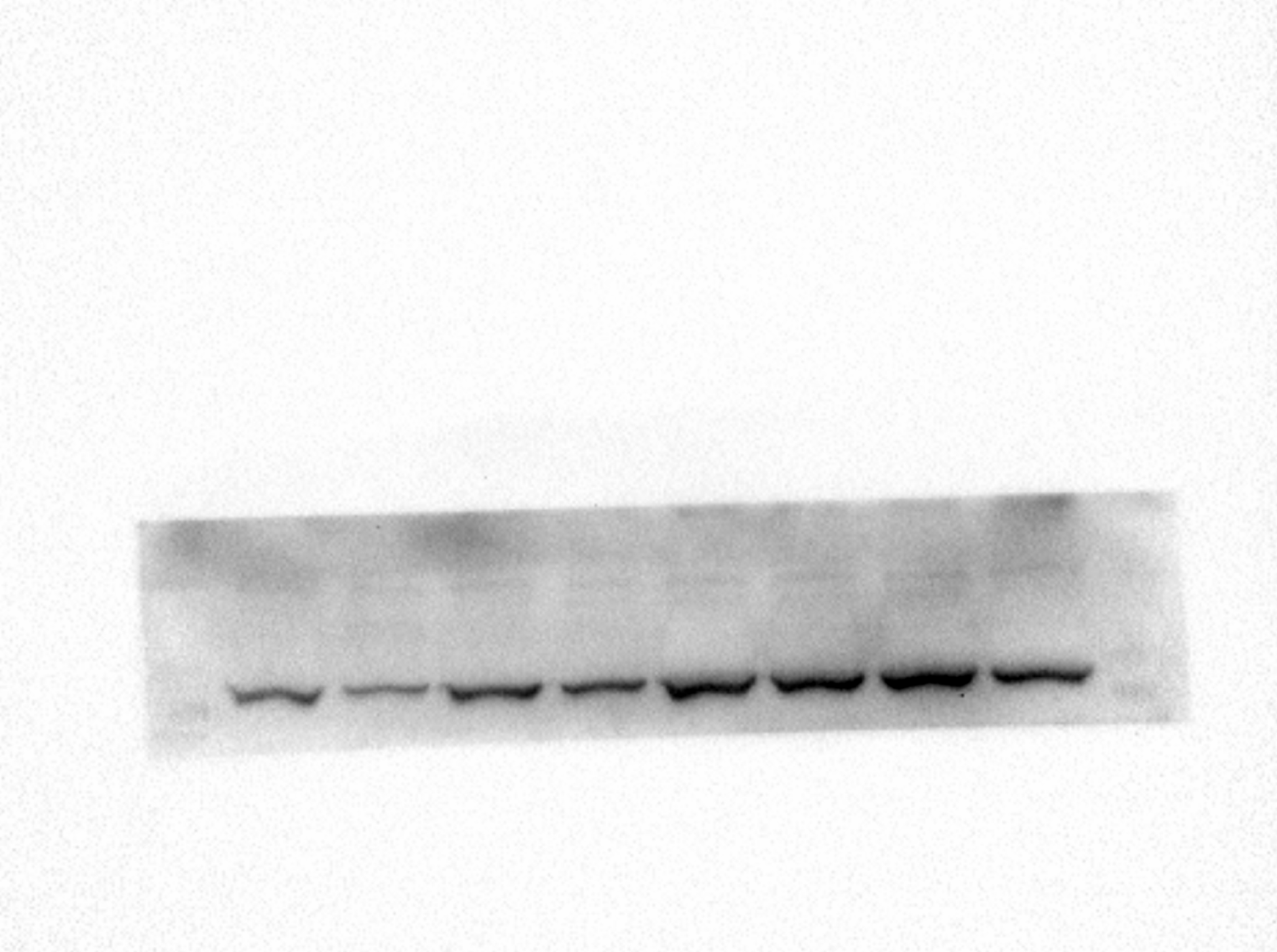

Supplement: Supplementary file 10 [file DataSheet10.ZIP › F5/P-AKT.tif]

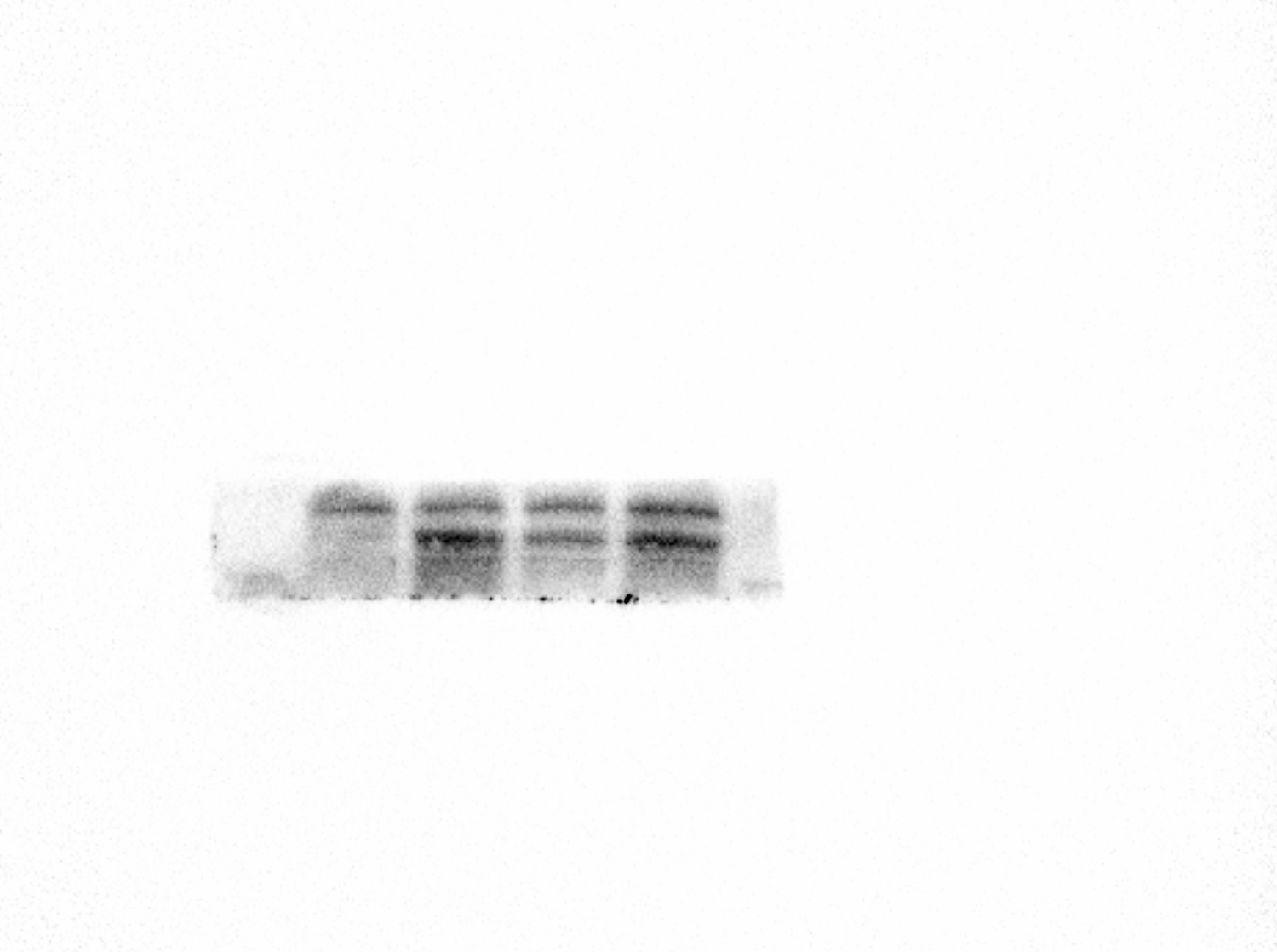

Supplement: Supplementary file 10 [file DataSheet10.ZIP › F6 CO-IP/CAS11.tif]

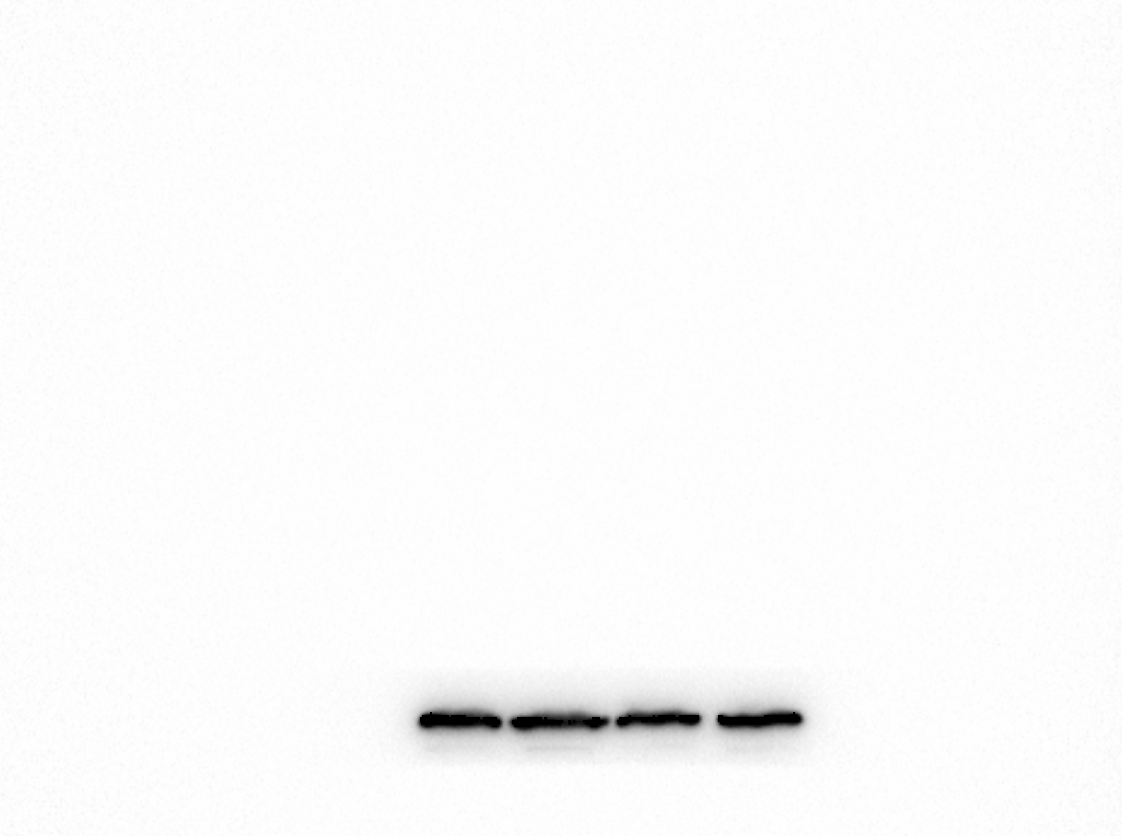

Supplement: Supplementary file 10 [file DataSheet10.ZIP › F6 CO-IP/GAPDH.tif]

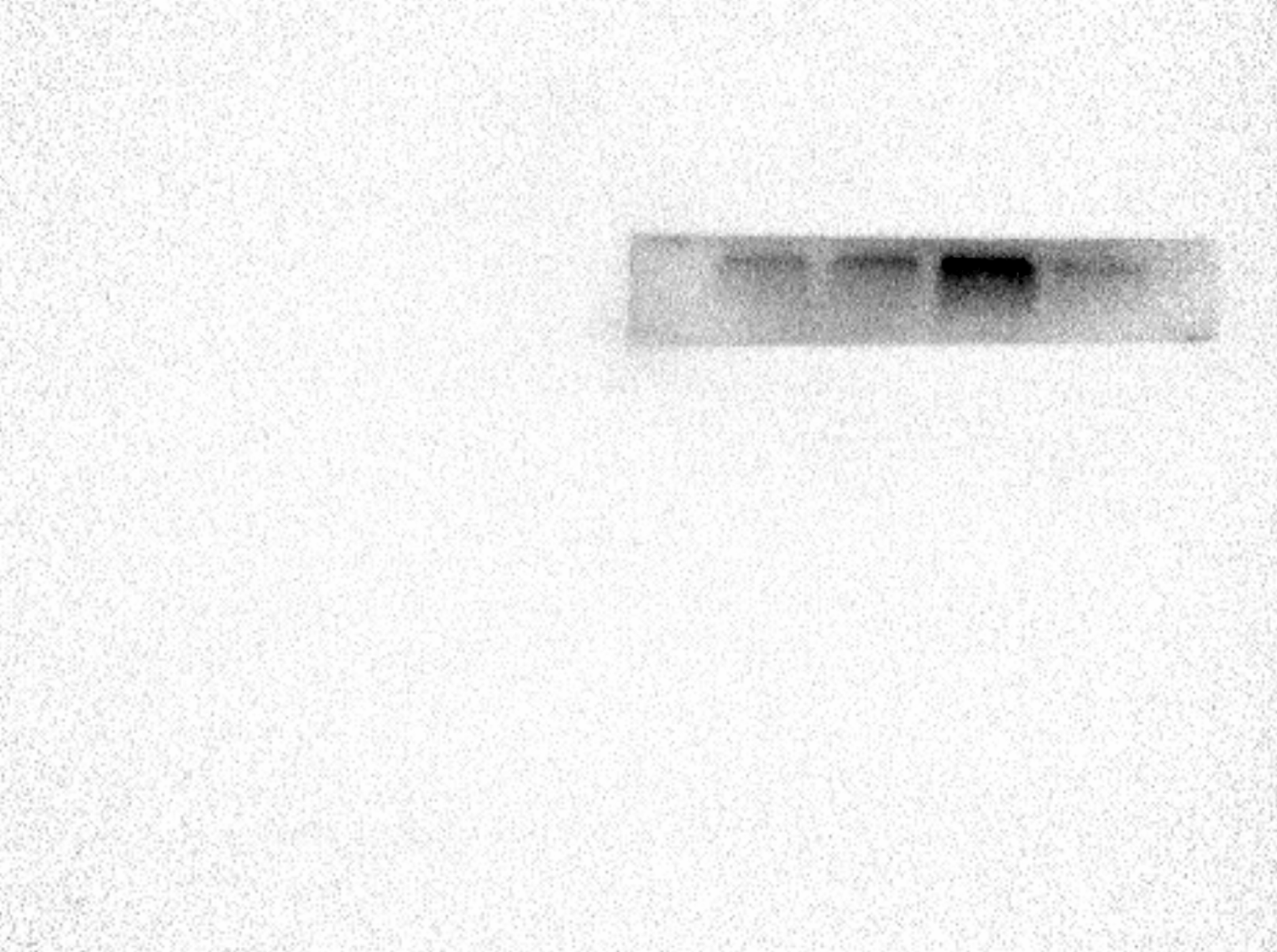

Supplement: Supplementary file 10 [file DataSheet10.ZIP › F6 CO-IP/N4.tif]

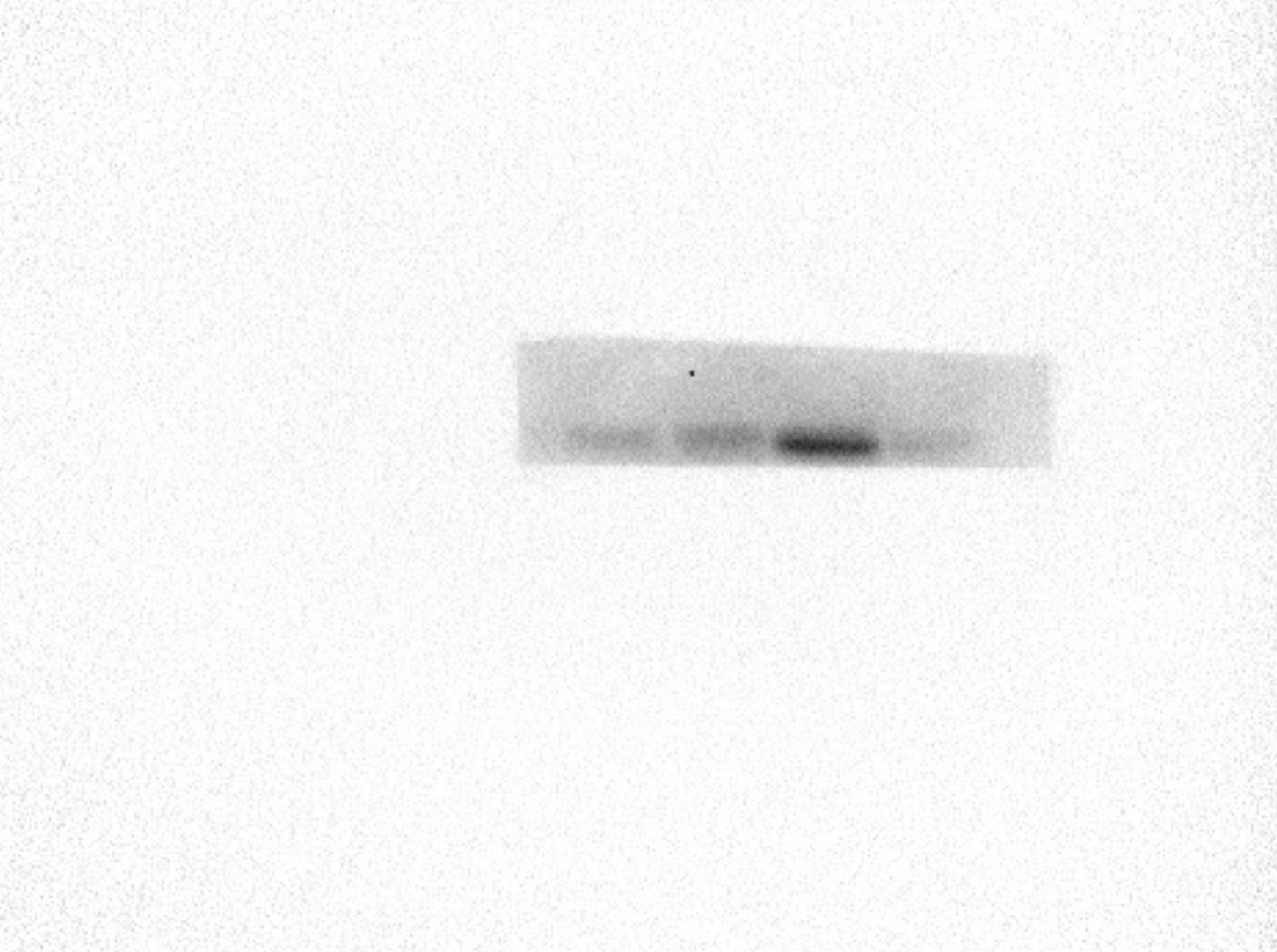

Supplement: Supplementary file 10 [file DataSheet10.ZIP › F6 CO-IP/NEDD4.tif]

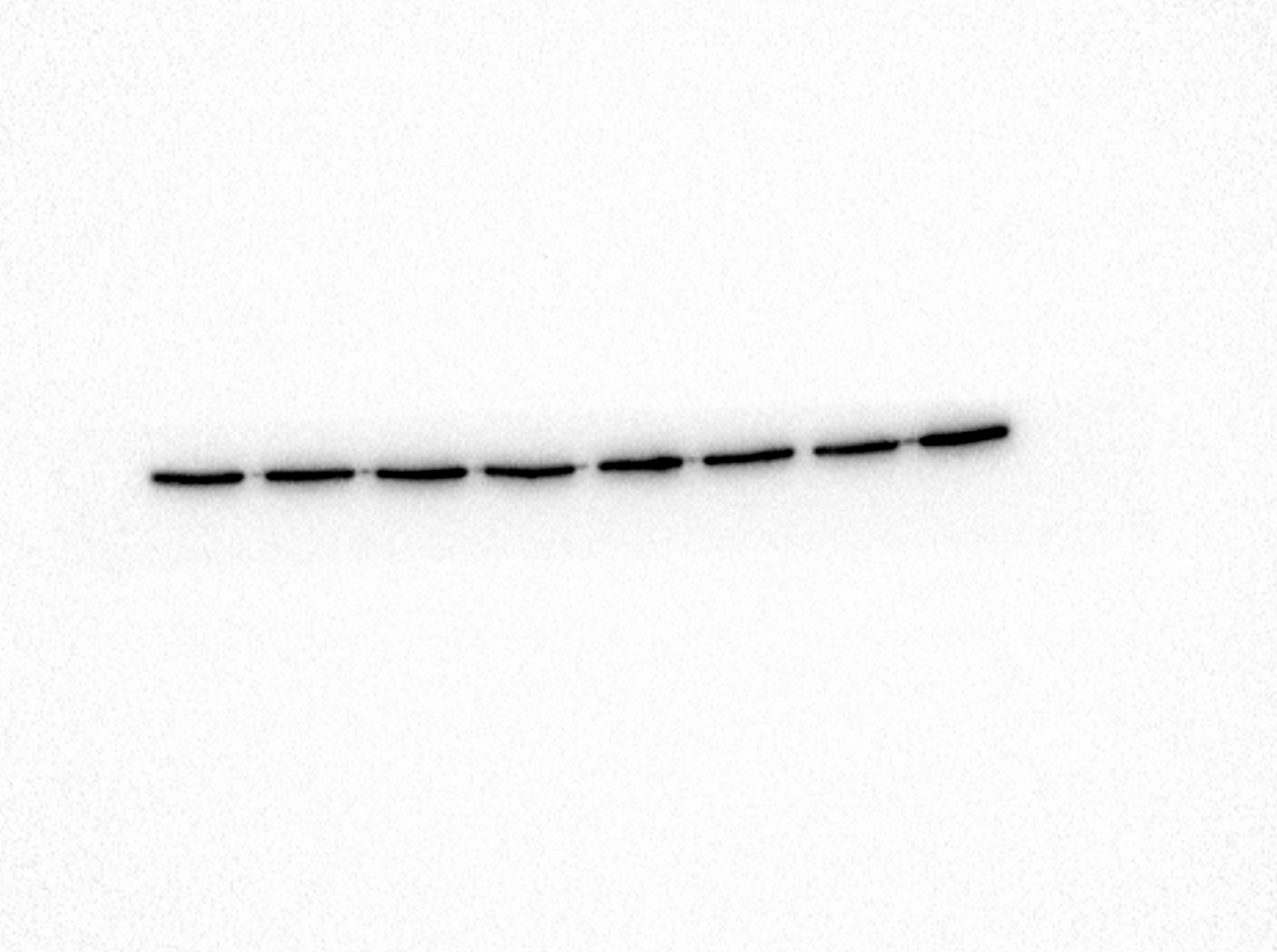

Supplement: Supplementary file 10 [file DataSheet10.ZIP › F6/GAPDH.tif]

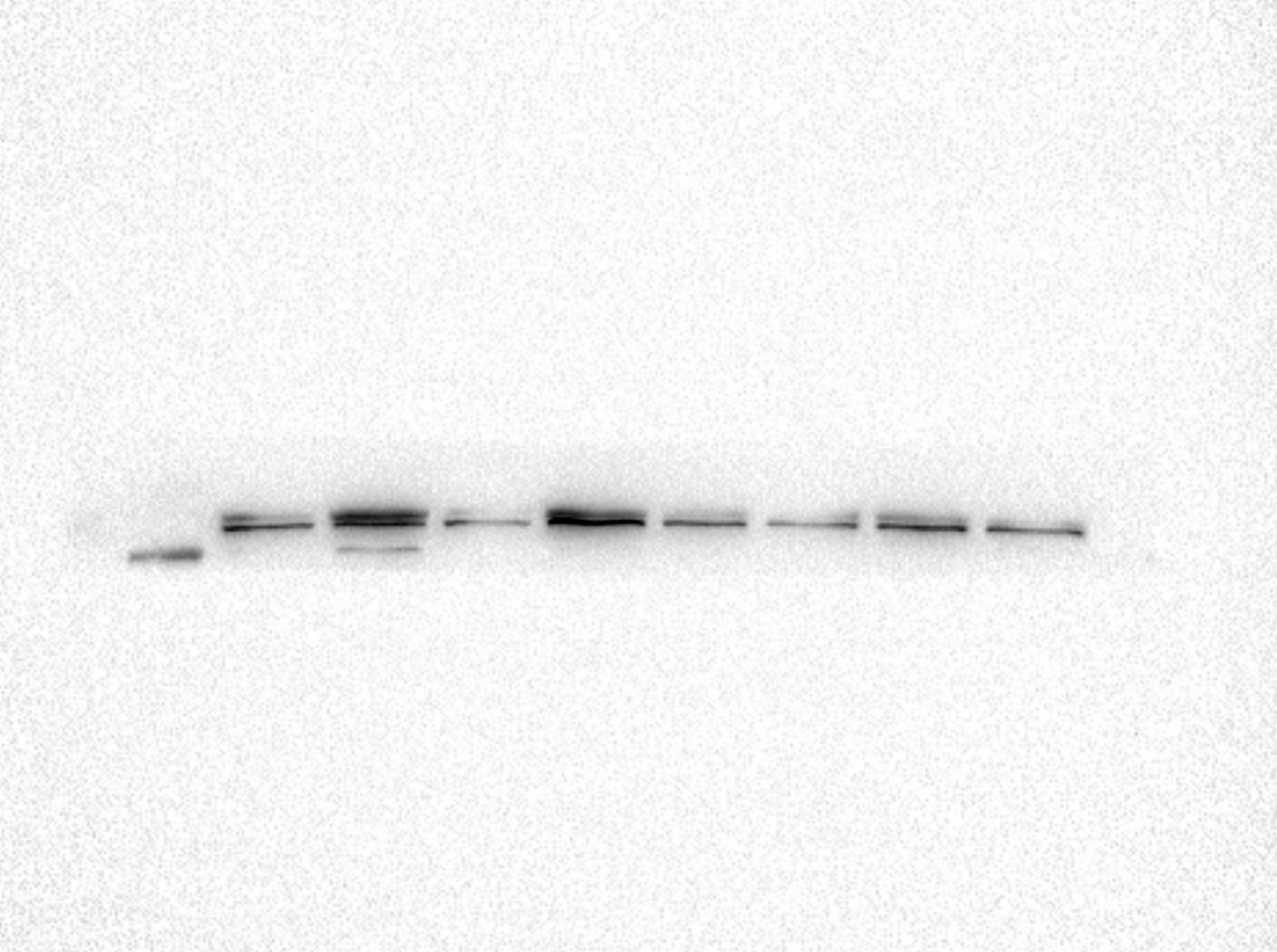

Supplement: Supplementary file 10 [file DataSheet10.ZIP › F6/NEDD4.tif]

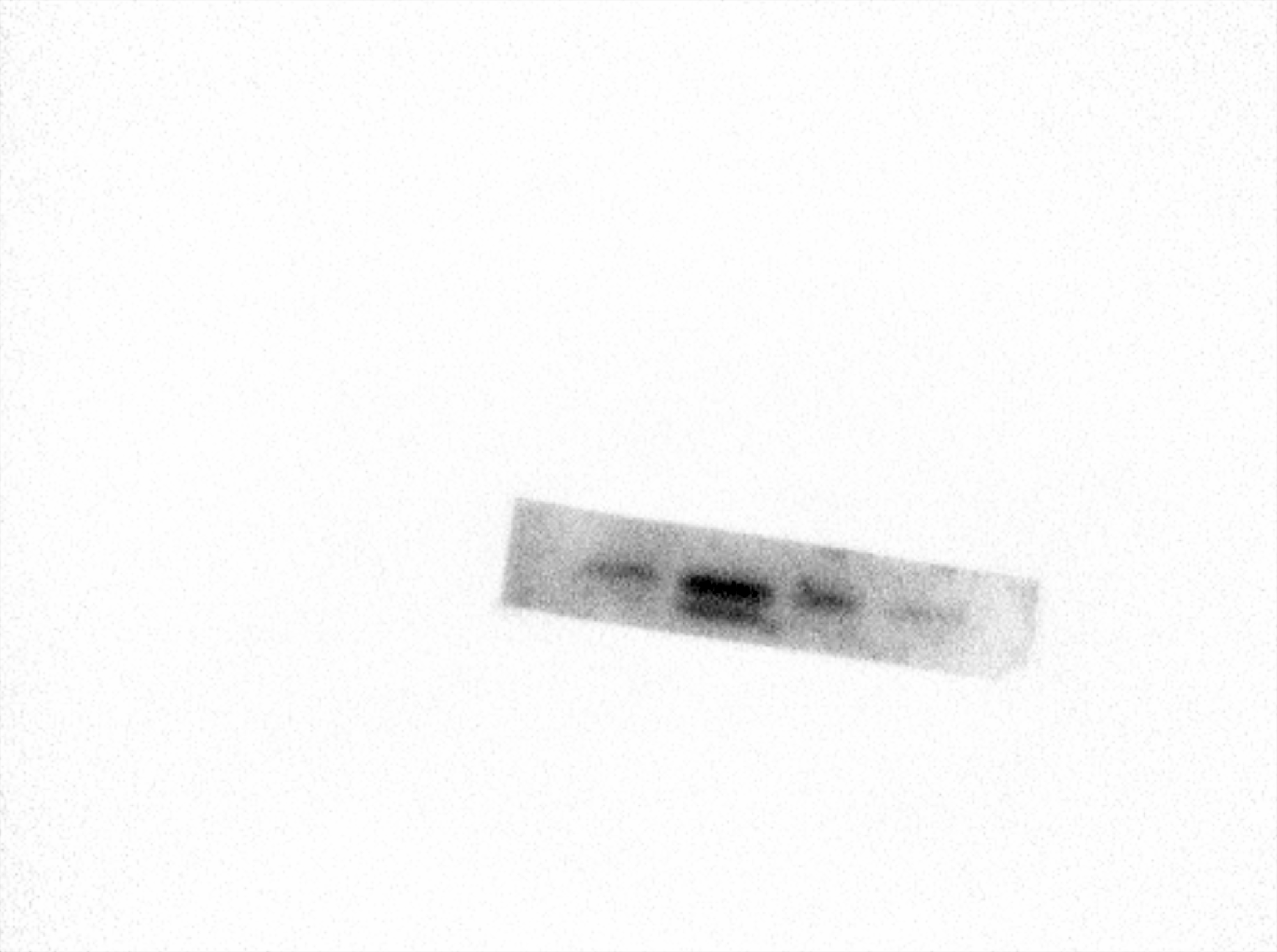

Supplement: Supplementary file 10 [file DataSheet10.ZIP › SF1/C11.tif]

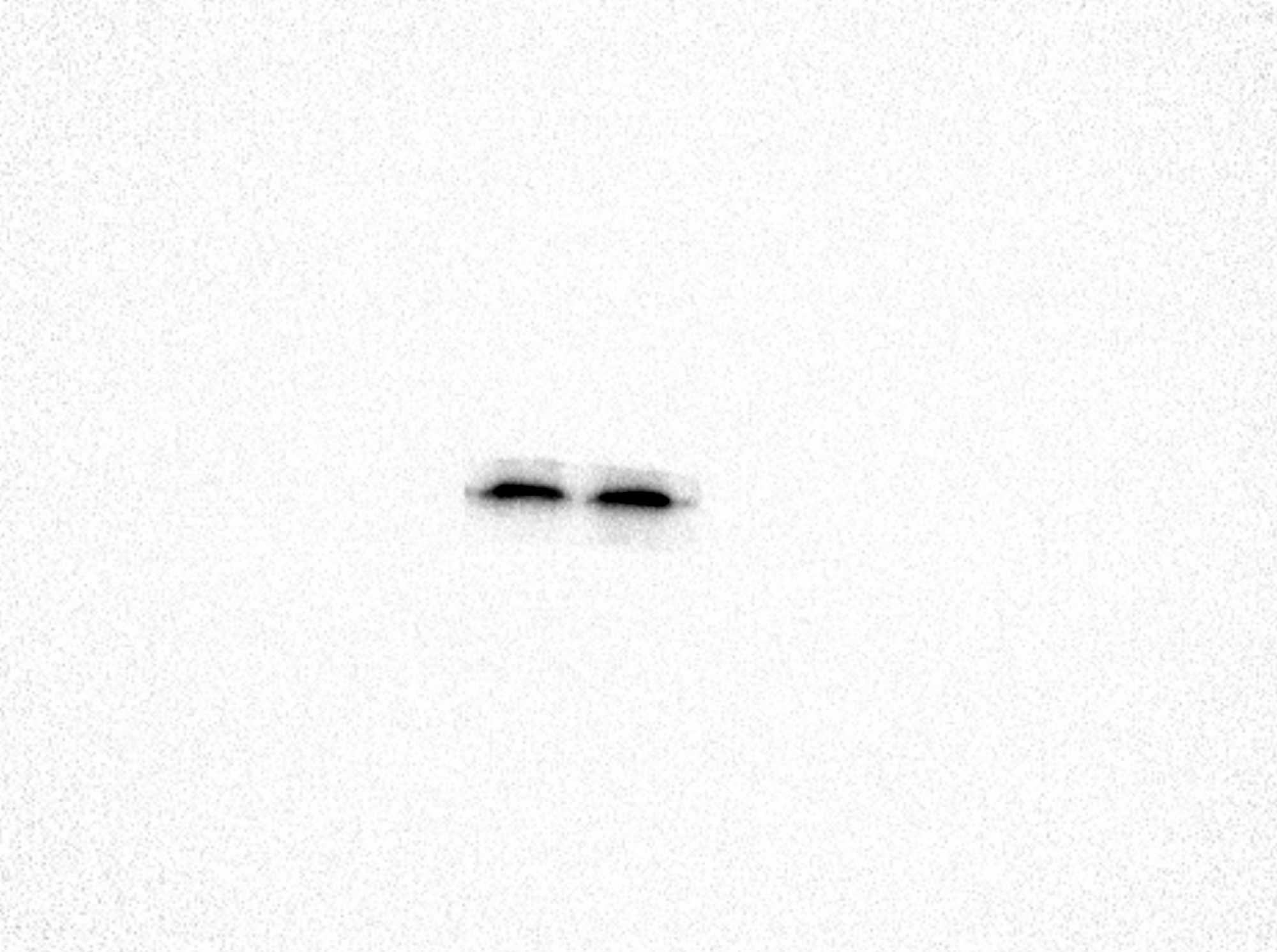

Supplement: Supplementary file 10 [file DataSheet10.ZIP › SF1/GAPDH.tif]

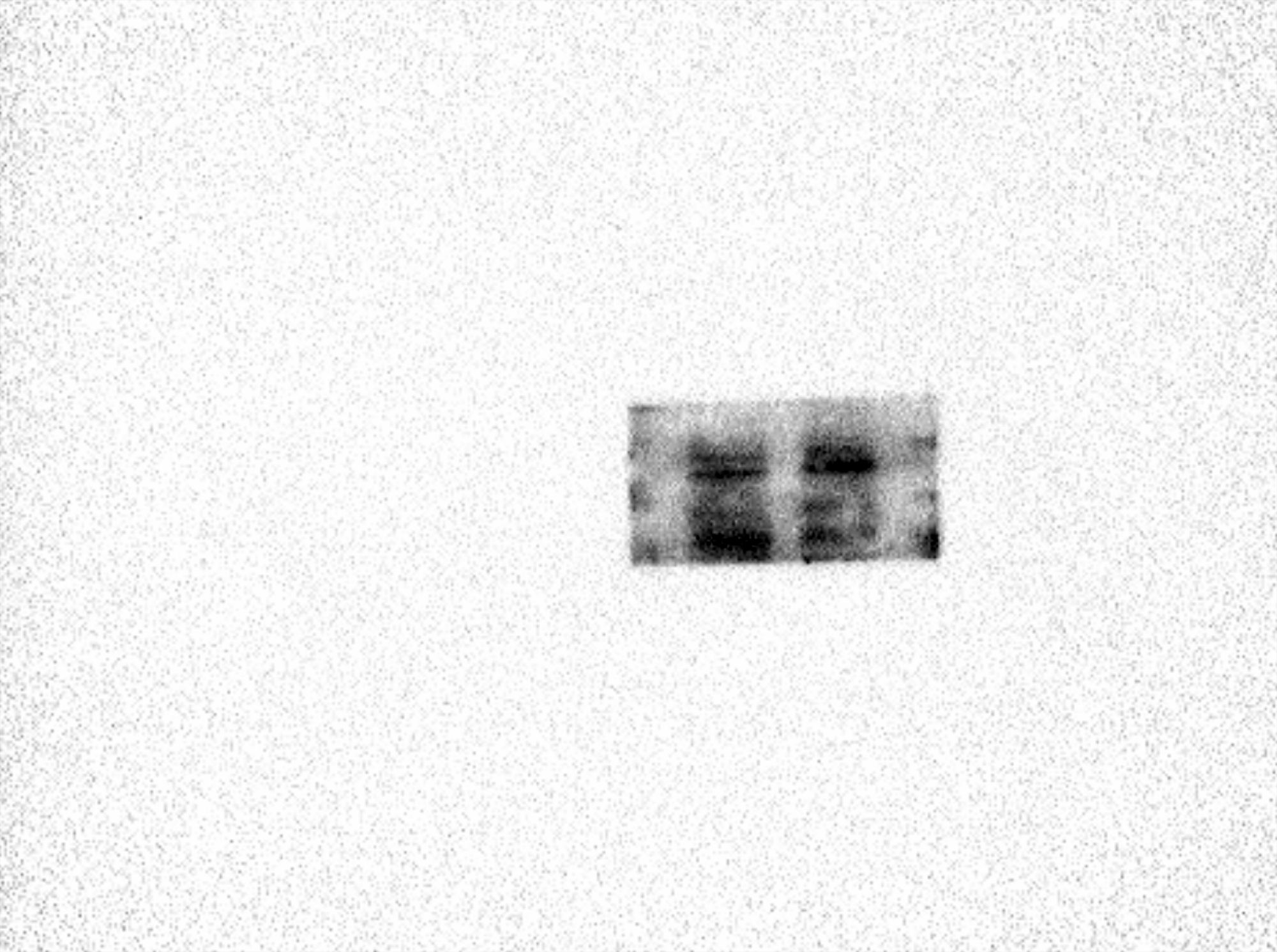

Supplement: Supplementary file 10 [file DataSheet10.ZIP › SF1/GSDMD.tif]

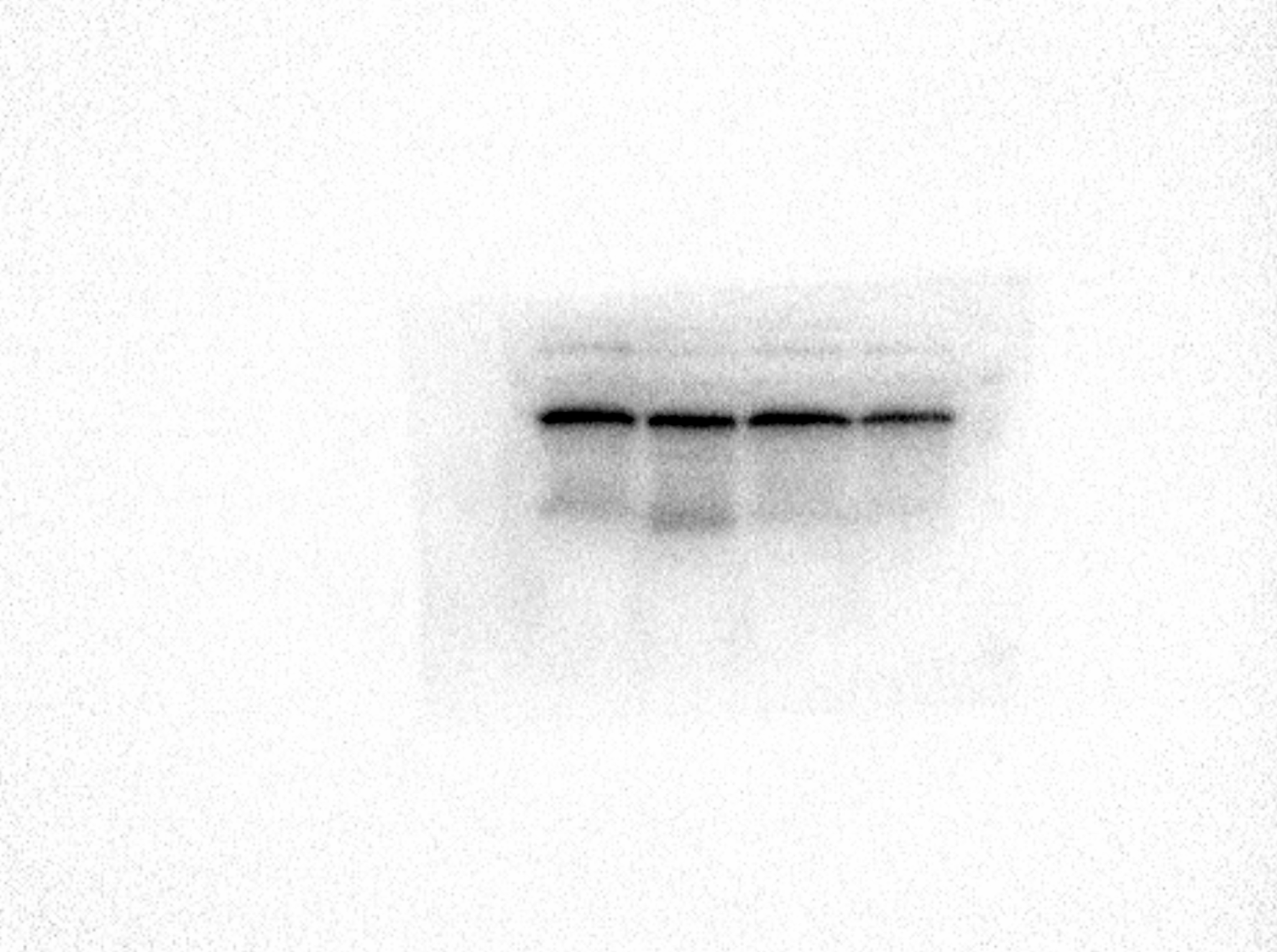

Supplement: Supplementary file 10 [file DataSheet10.ZIP › SF3/ATG5.tif]

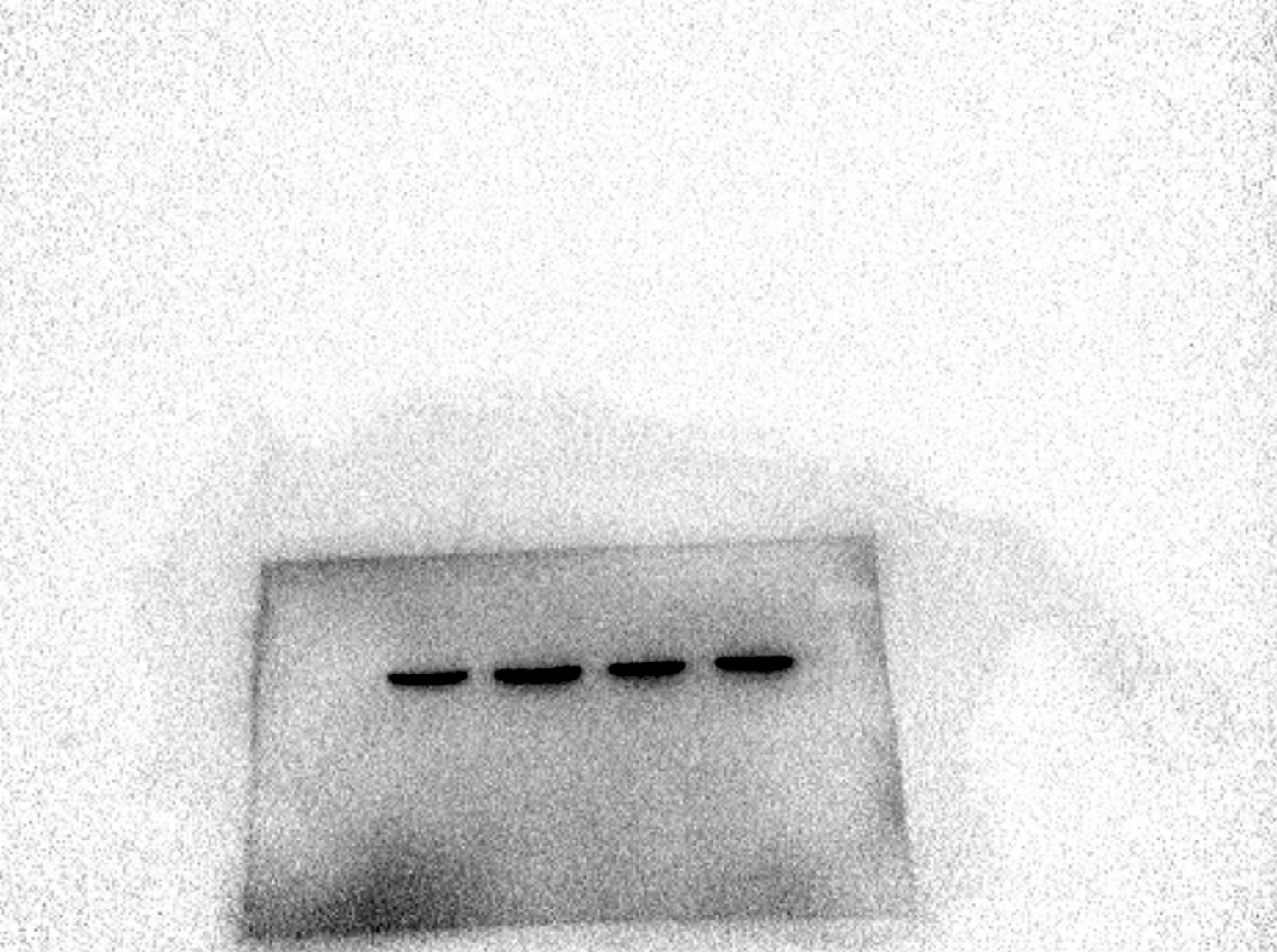

Supplement: Supplementary file 10 [file DataSheet10.ZIP › SF3/BECLIN-1.tif]

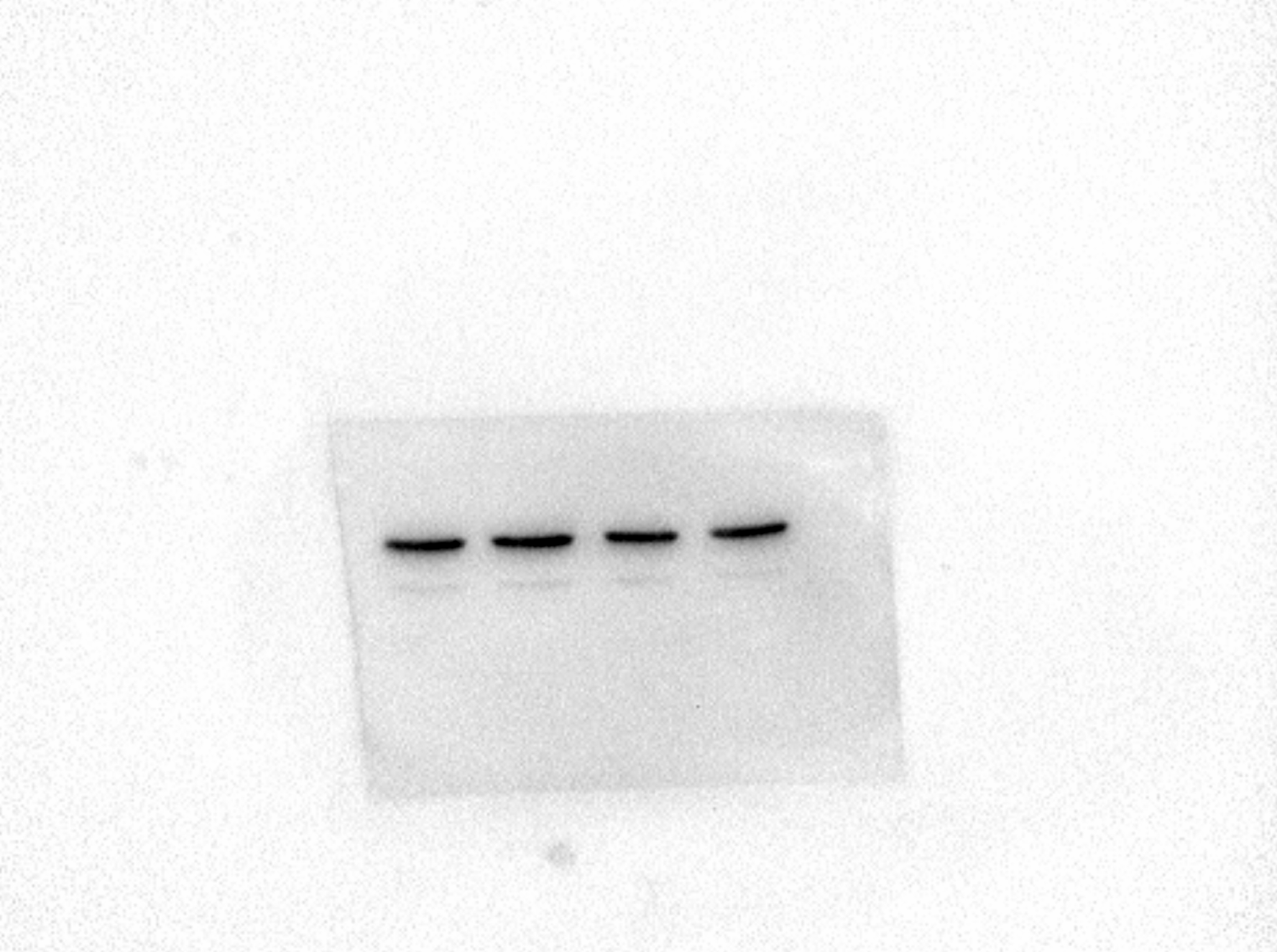

Supplement: Supplementary file 10 [file DataSheet10.ZIP › SF3/GAPDH.tif]

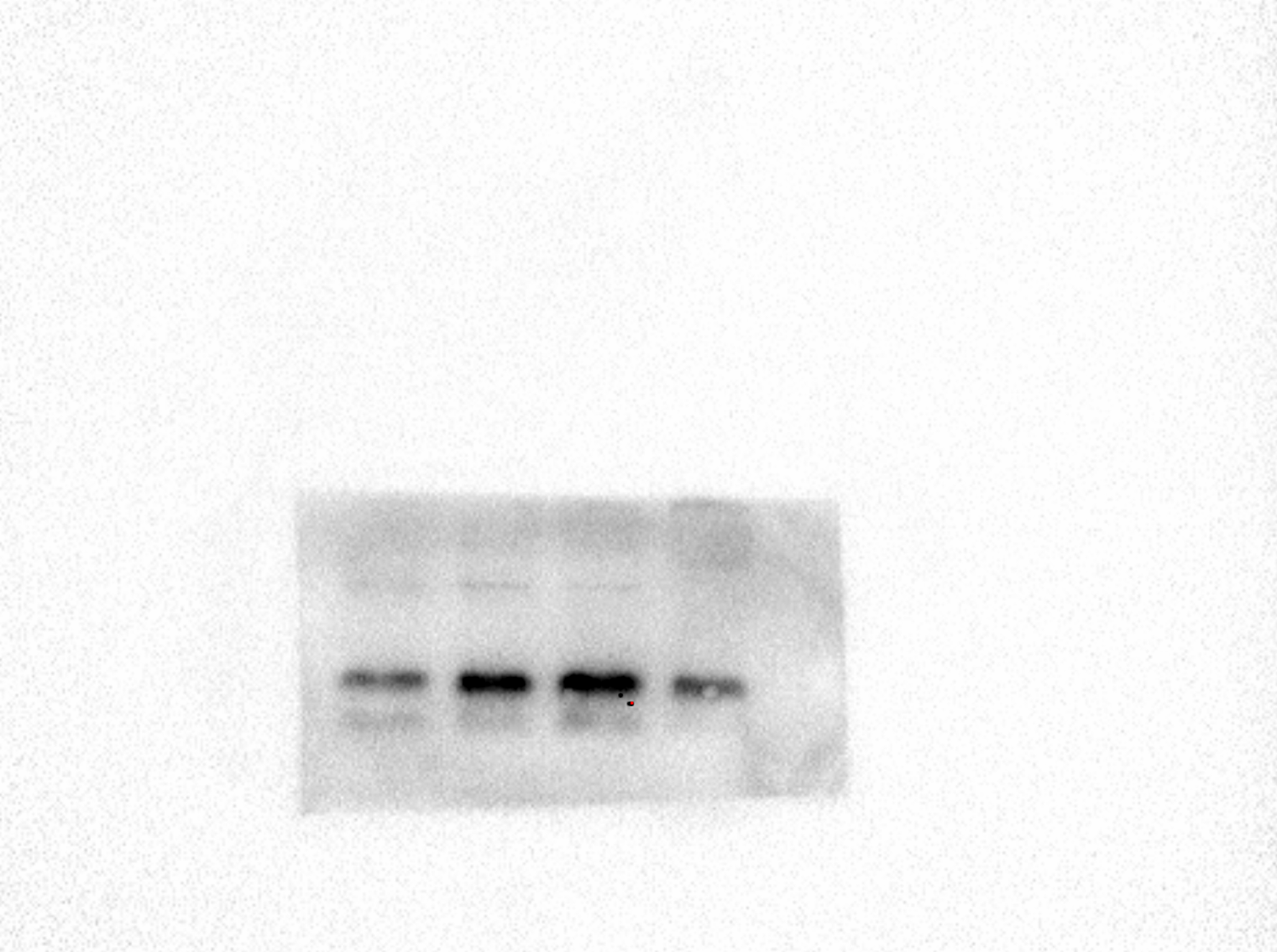

Supplement: Supplementary file 10 [file DataSheet10.ZIP › SF3/LC3.tif]

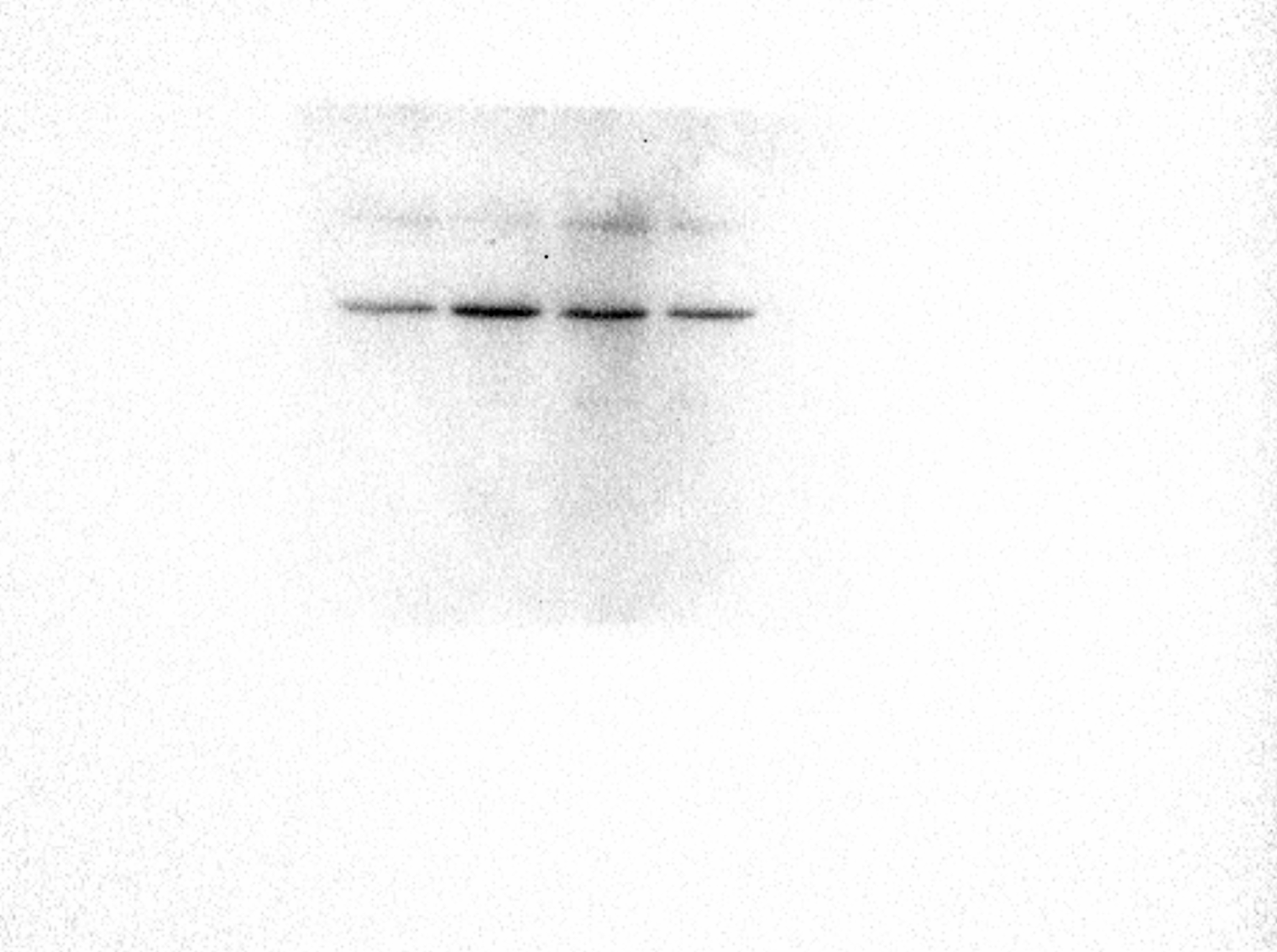

Supplement: Supplementary file 10 [file DataSheet10.ZIP › SF3/P62.tif]

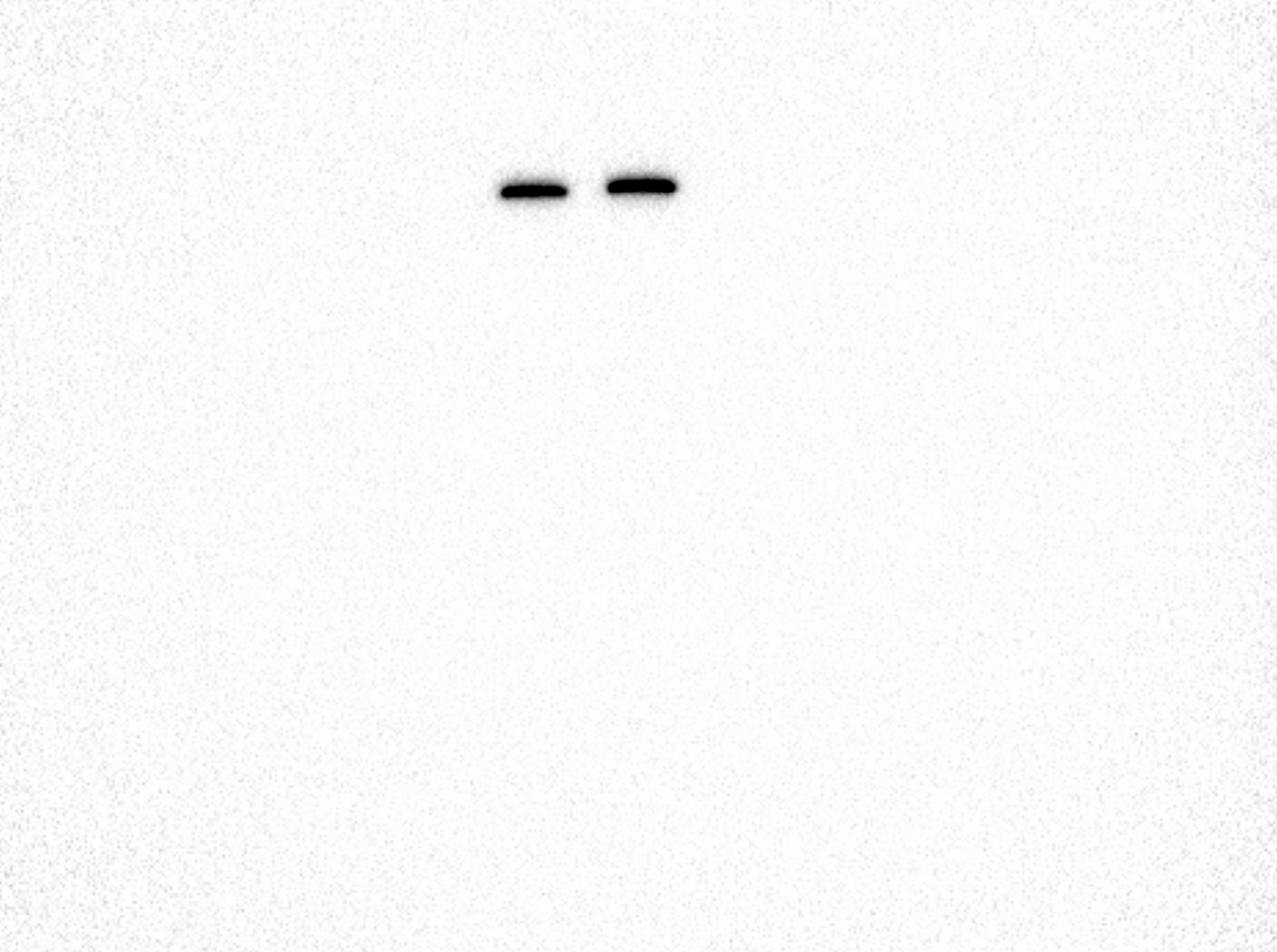

Supplement: Supplementary file 10 [file DataSheet10.ZIP › SF3/Sirtuin 1.tif]

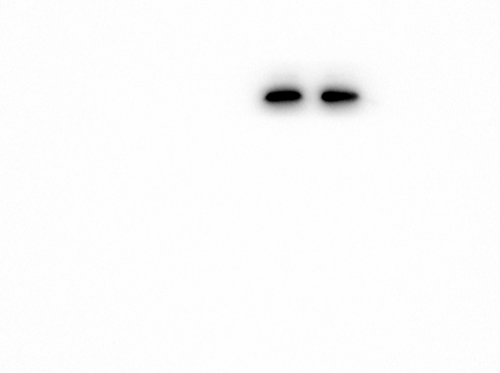

Supplement: Supplementary file 10 [file DataSheet10.ZIP › SF3/GAPDH2.tif]

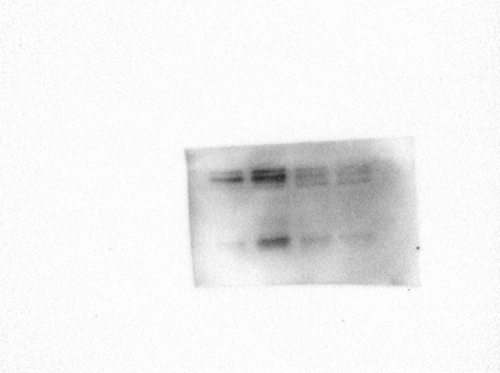

Supplement: Supplementary file 10 [file DataSheet10.ZIP › F4/caspase-11.tif]

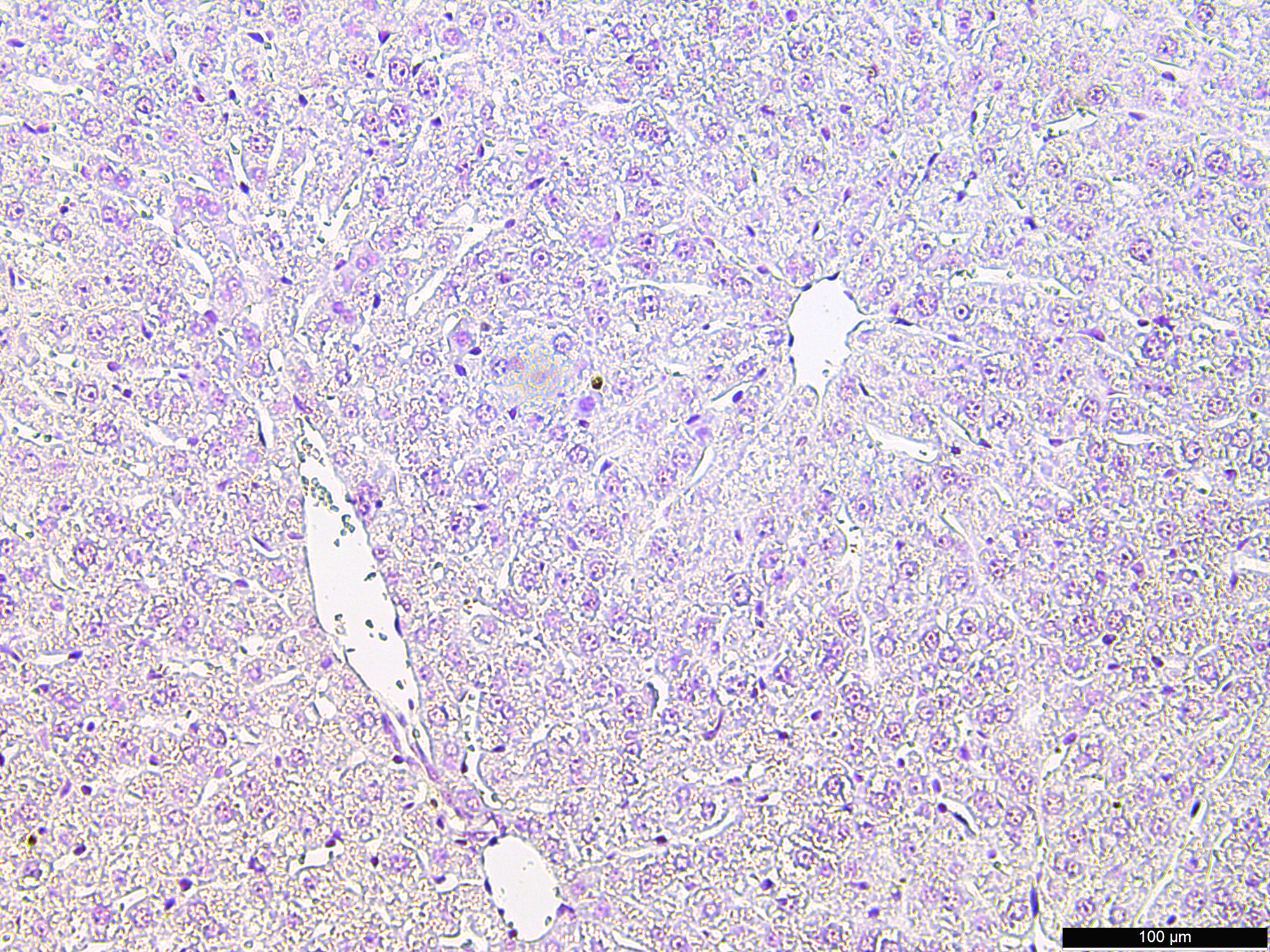

Supplement: Supplementary file 11 [file DataSheet6.ZIP › CN.tif]

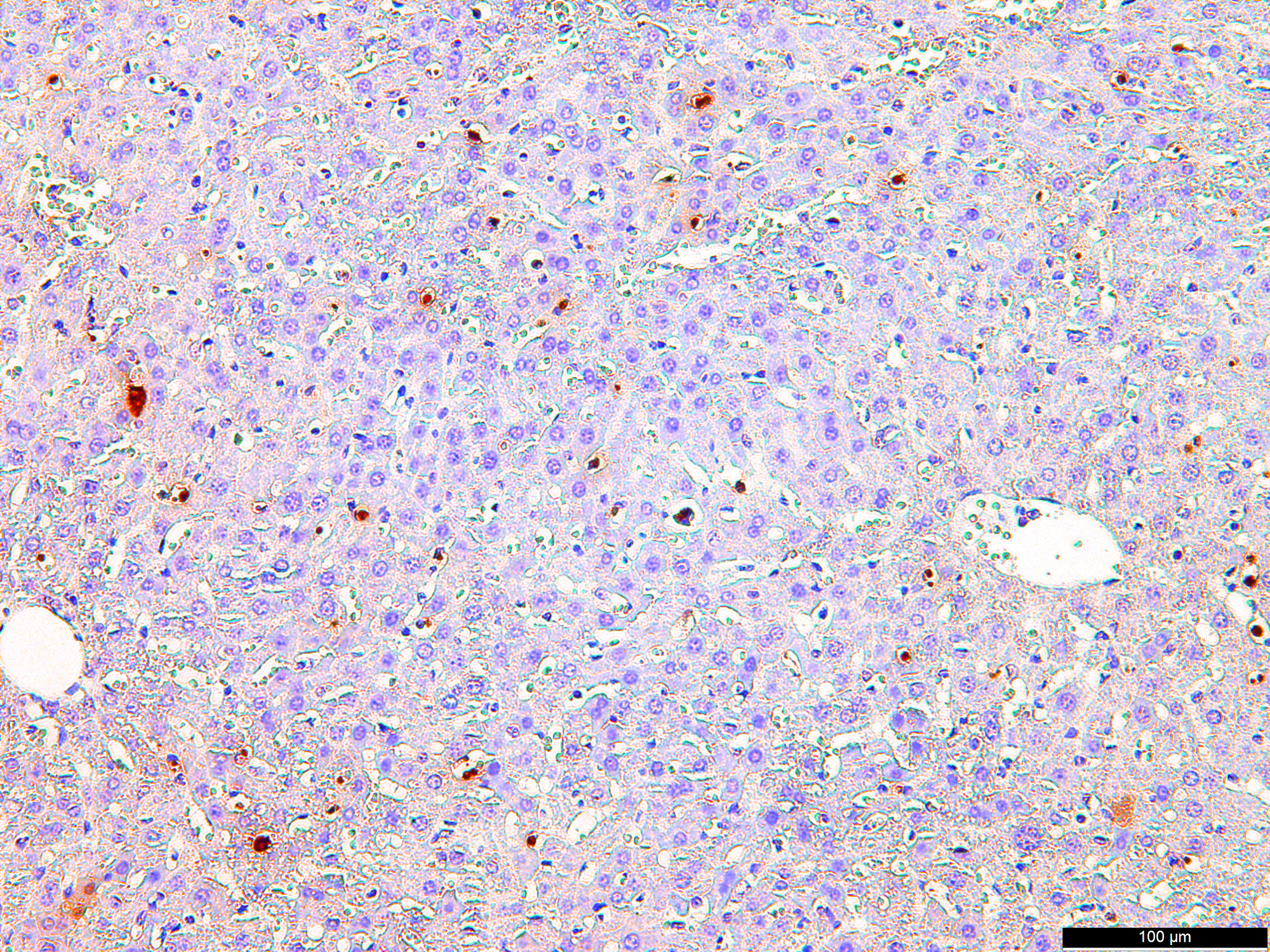

Supplement: Supplementary file 11 [file DataSheet6.ZIP › LPS.tif]

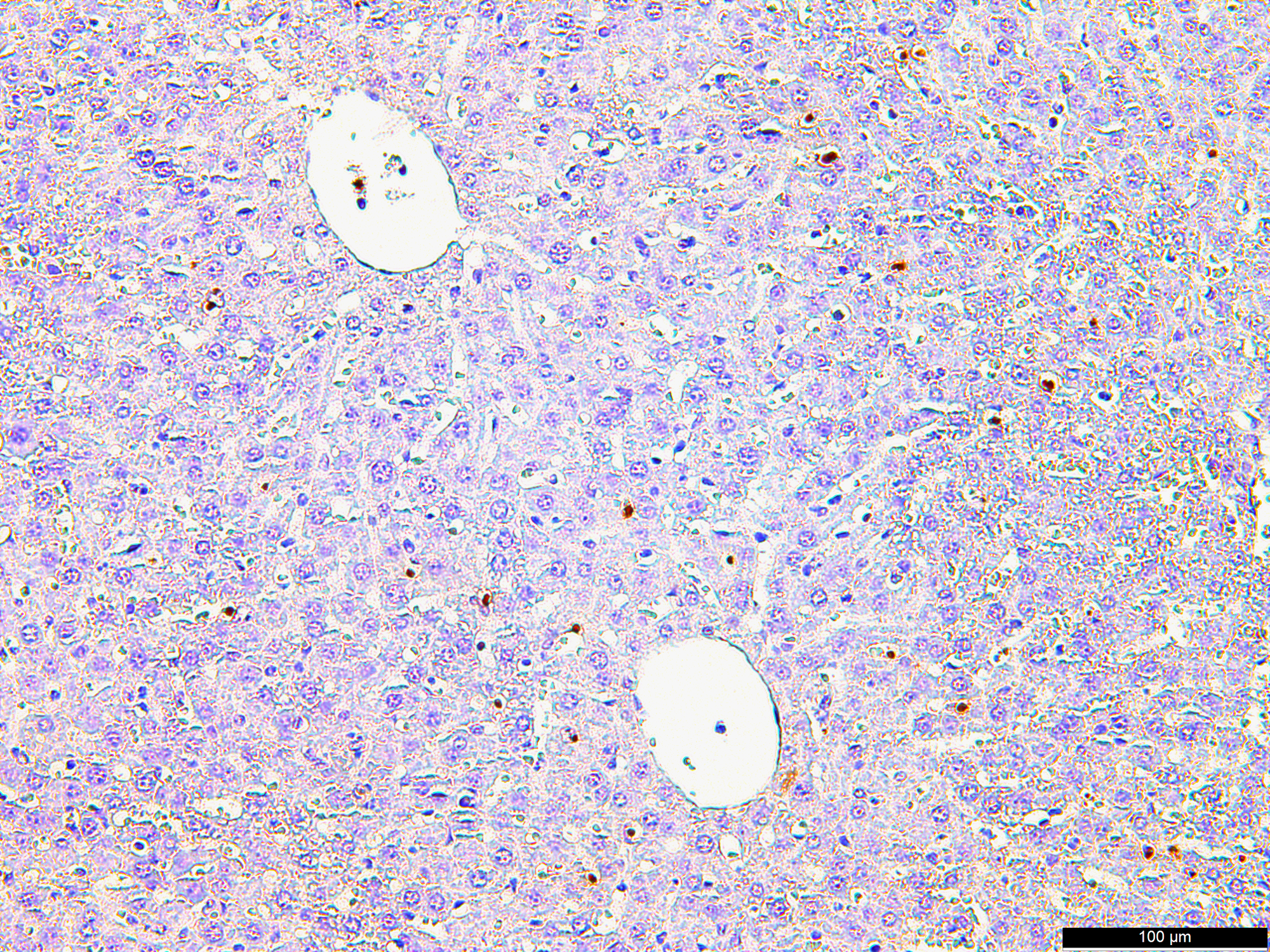

Supplement: Supplementary file 11 [file DataSheet6.ZIP › ST+LPS.tif]

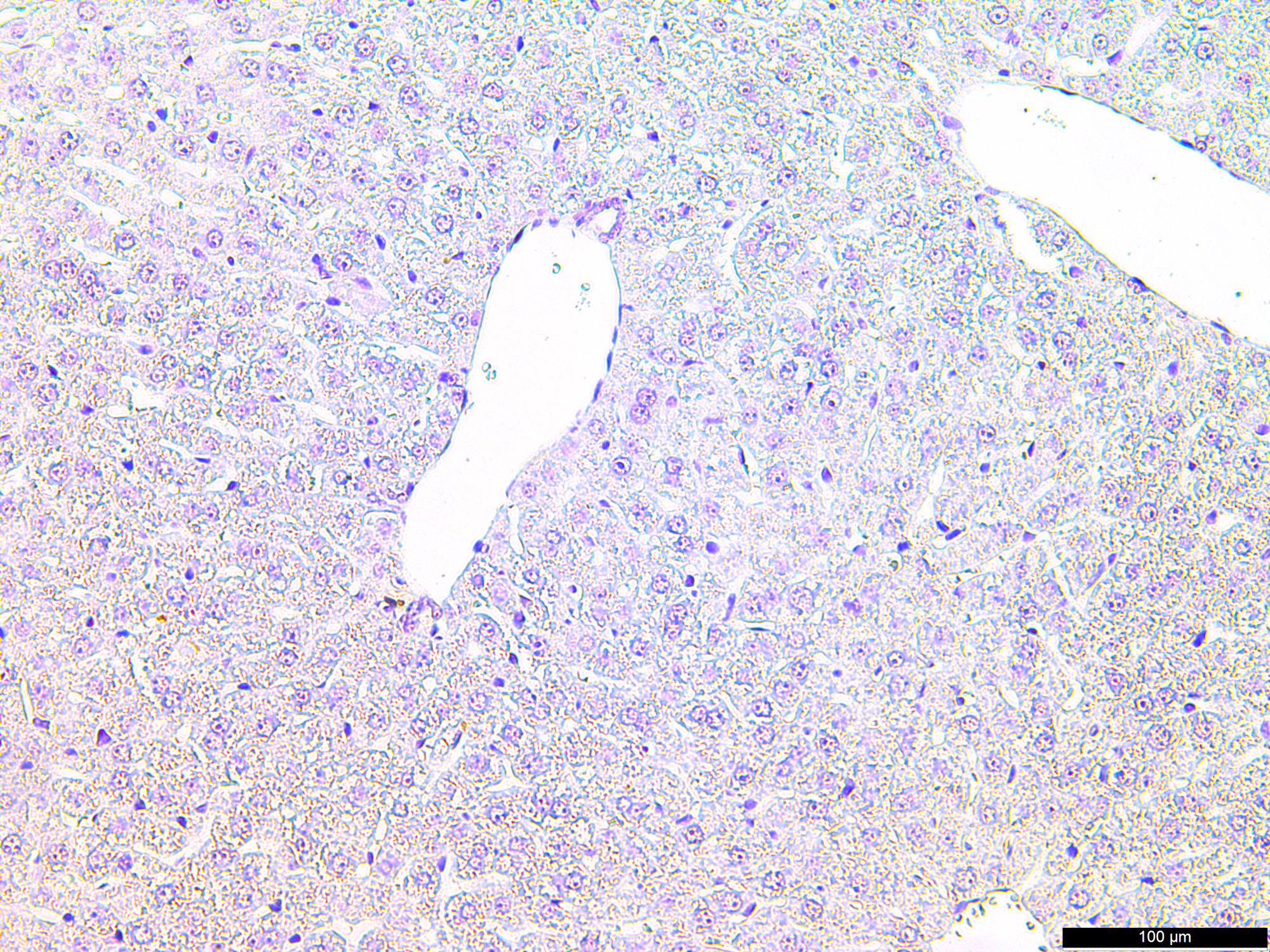

Supplement: Supplementary file 11 [file DataSheet6.ZIP › ST.tif]

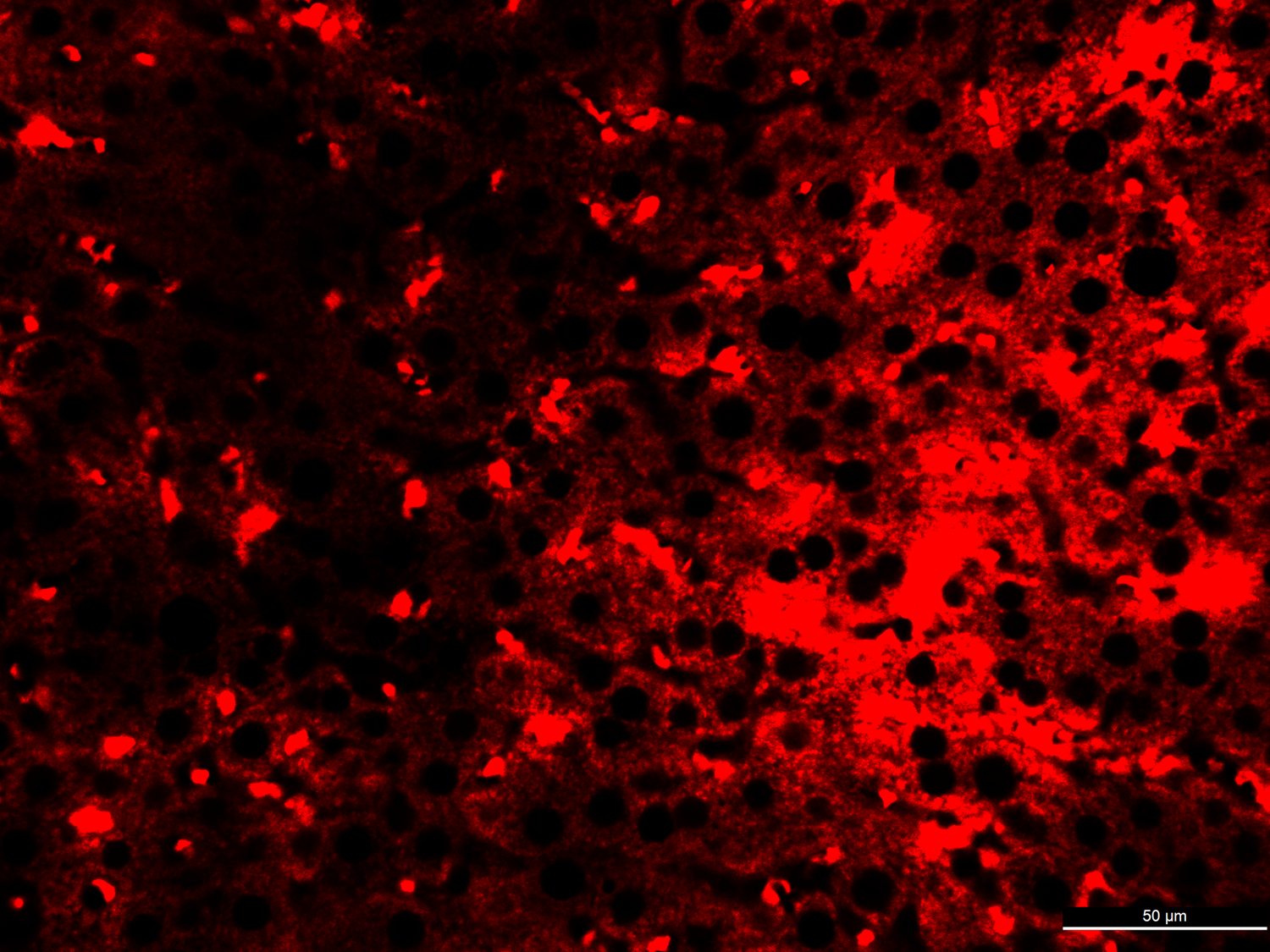

Supplement: Supplementary file 12 [file DataSheet2.ZIP › LPS/LPS (2).tif]

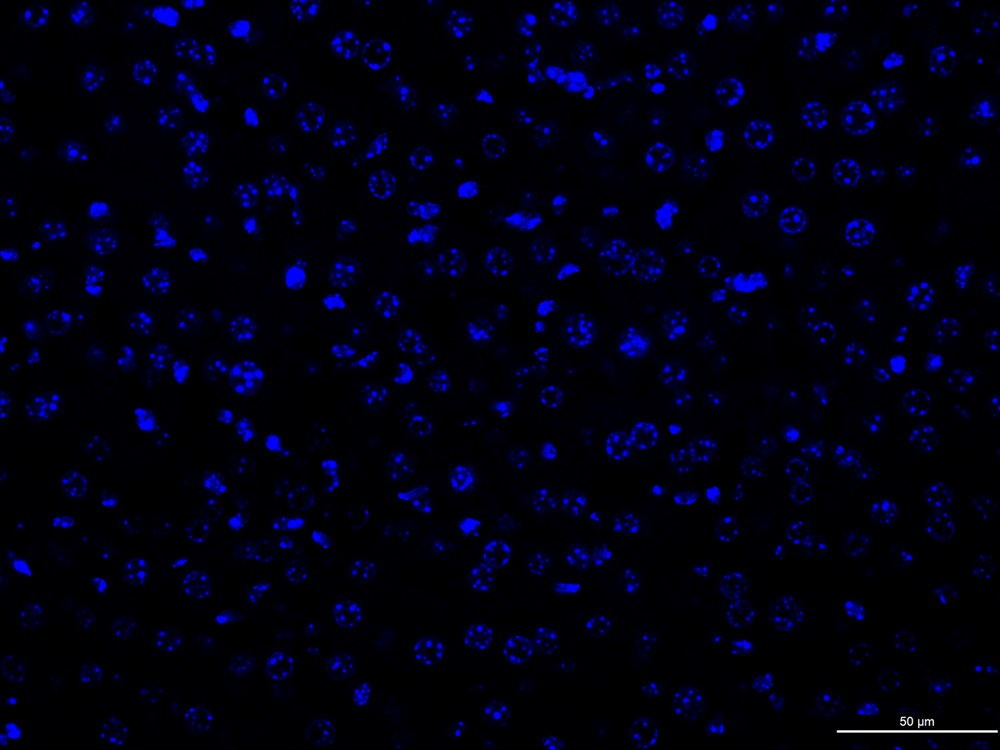

Supplement: Supplementary file 12 [file DataSheet2.ZIP › LPS/LPS (3).tif]

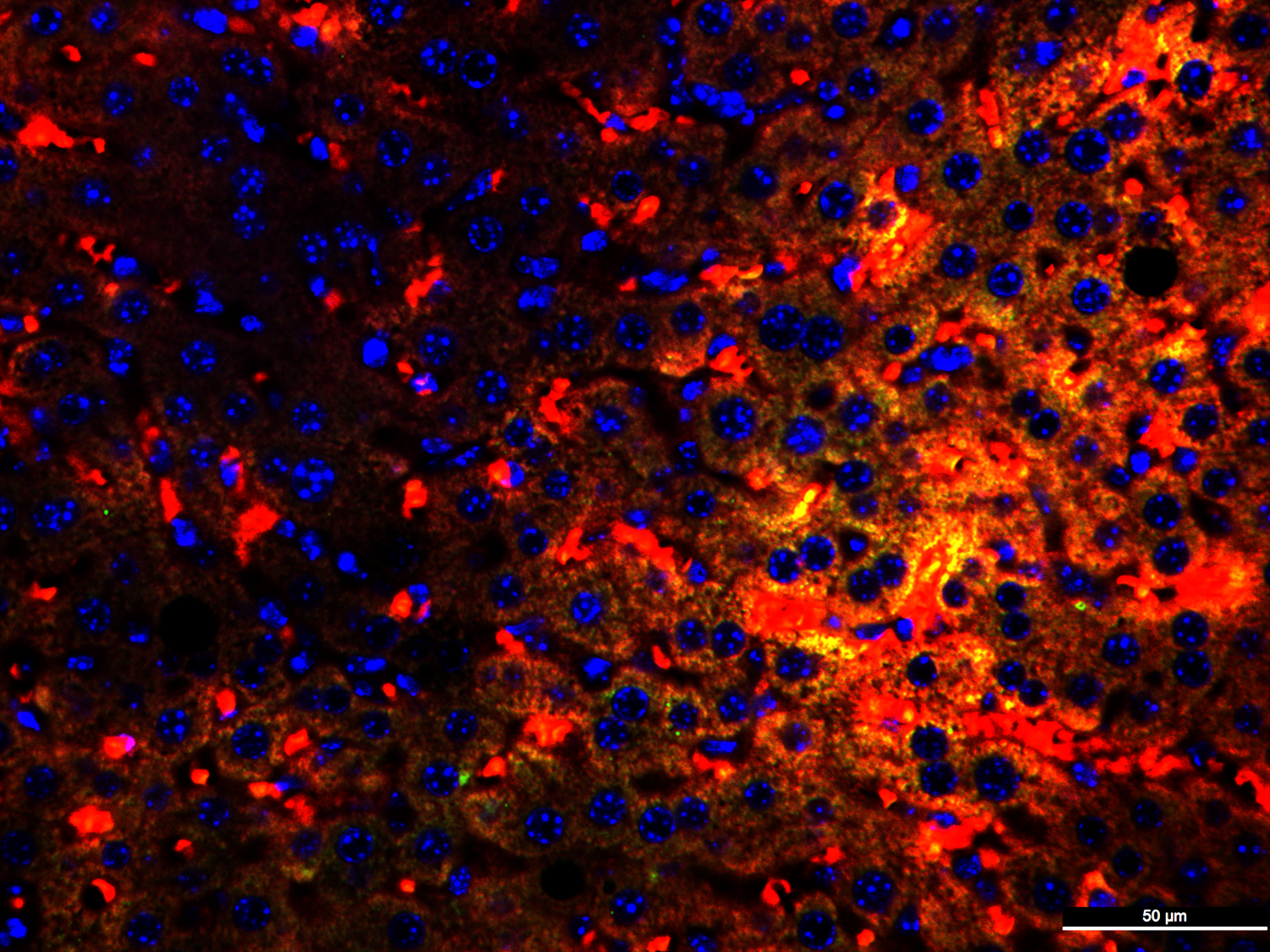

Supplement: Supplementary file 12 [file DataSheet2.ZIP › LPS/LPS(4).tif]

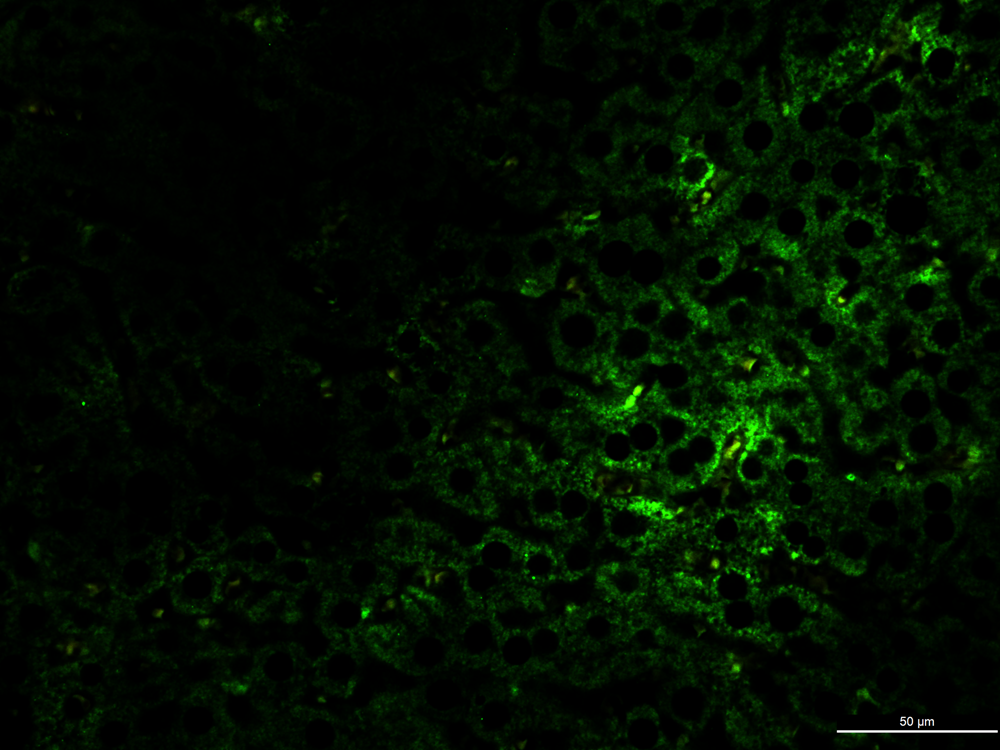

Supplement: Supplementary file 12 [file DataSheet2.ZIP › LPS/LPS.tif]

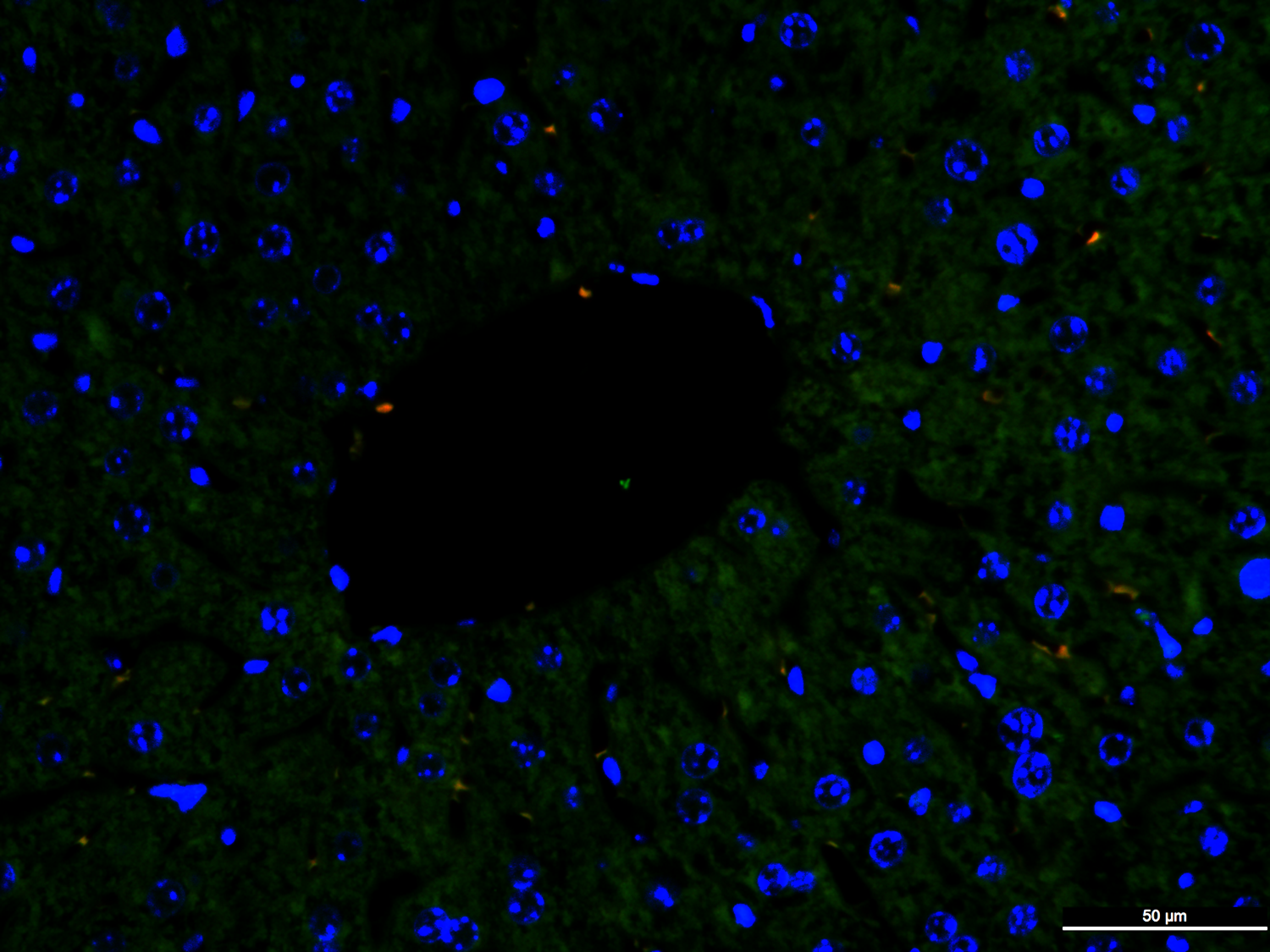

Supplement: Supplementary file 12 [file DataSheet2.ZIP › NC/N.tif]

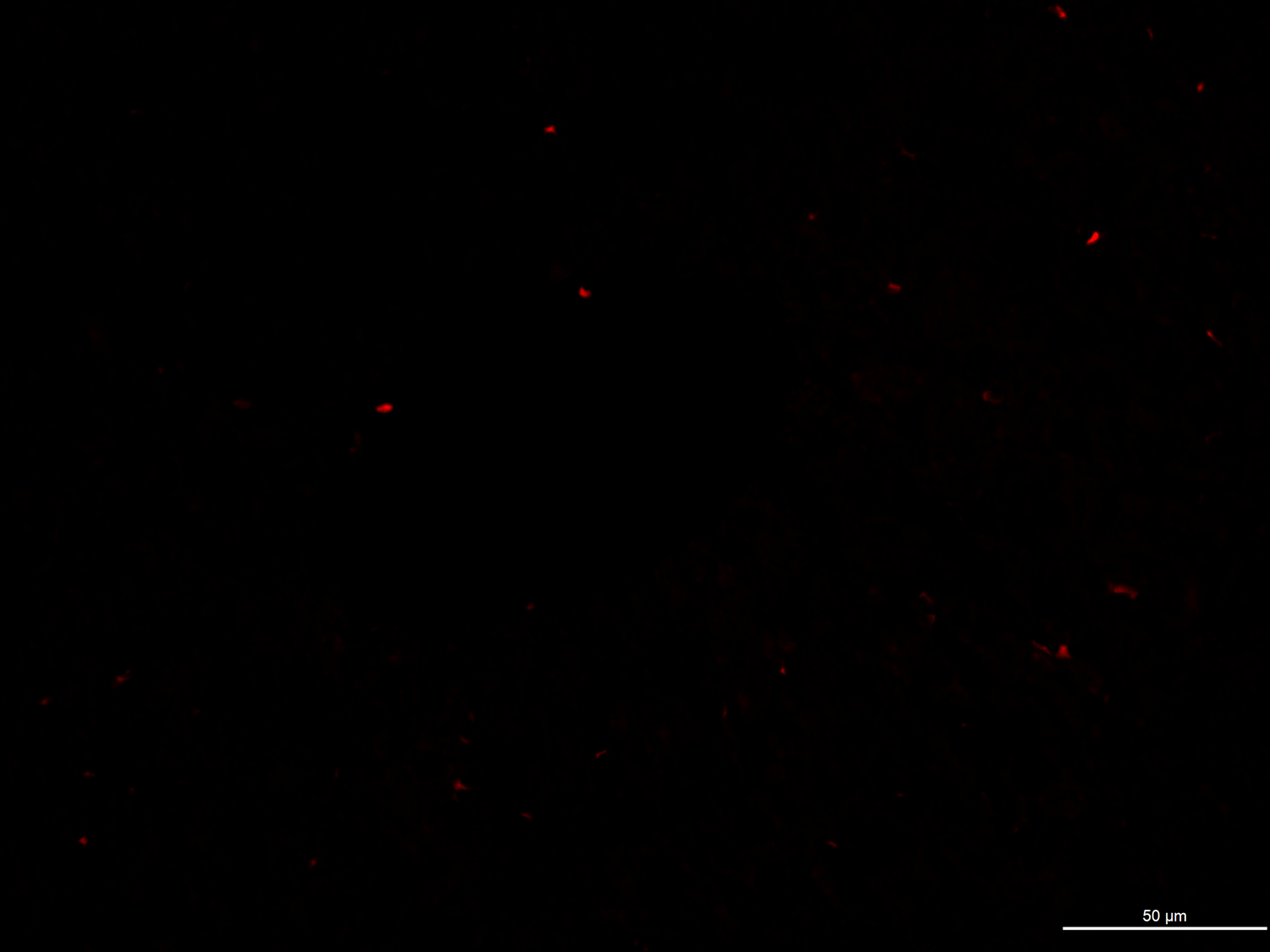

Supplement: Supplementary file 12 [file DataSheet2.ZIP › NC/NC (2).tif]

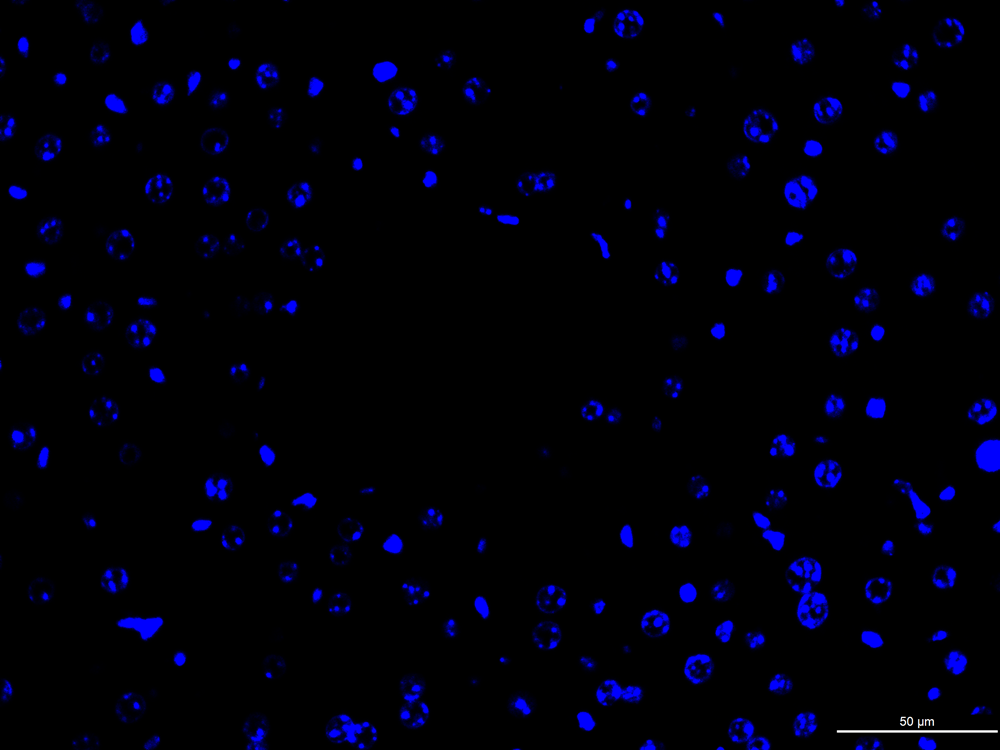

Supplement: Supplementary file 12 [file DataSheet2.ZIP › NC/NC (3).tif]

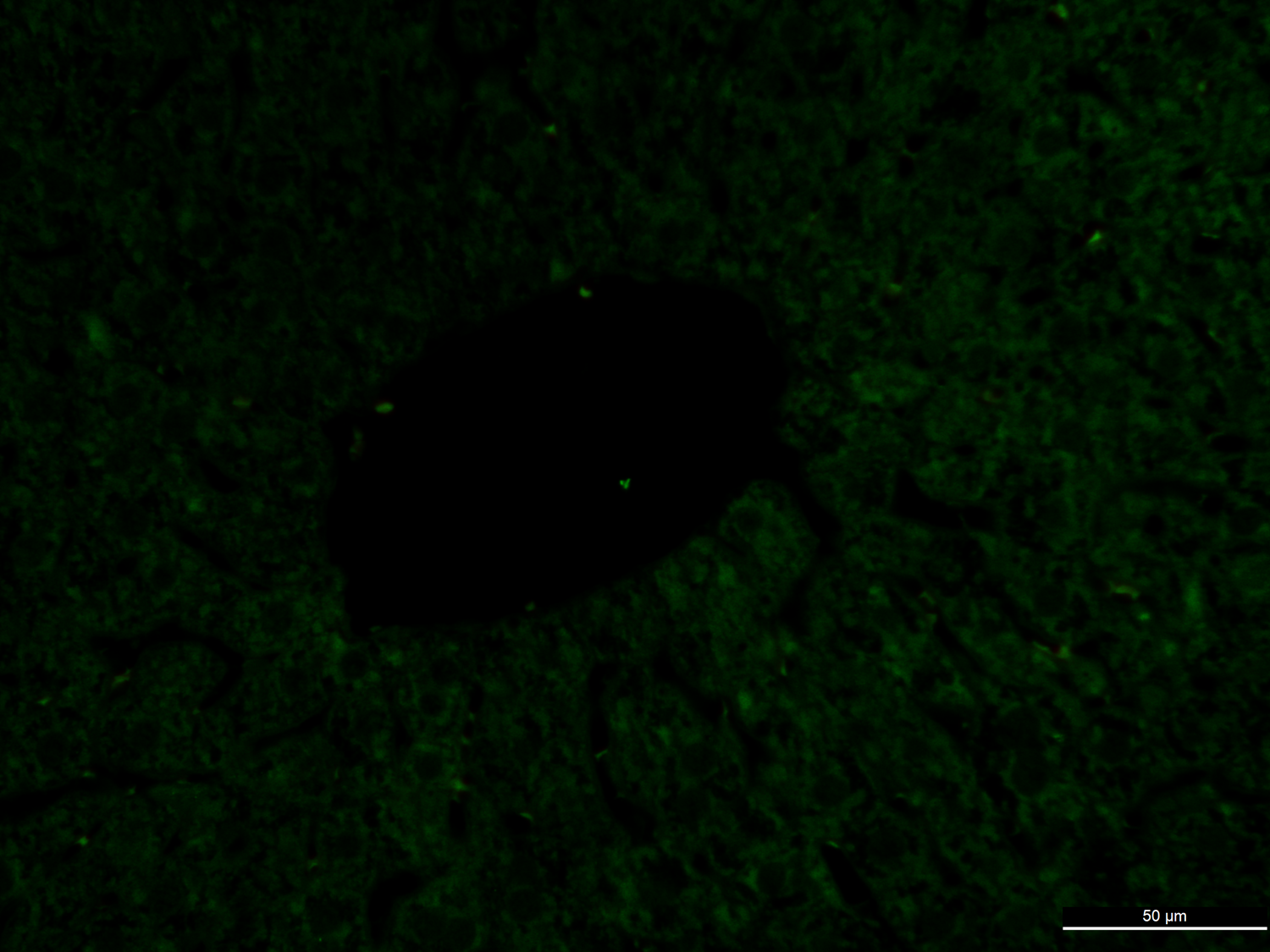

Supplement: Supplementary file 12 [file DataSheet2.ZIP › NC/NC.tif]

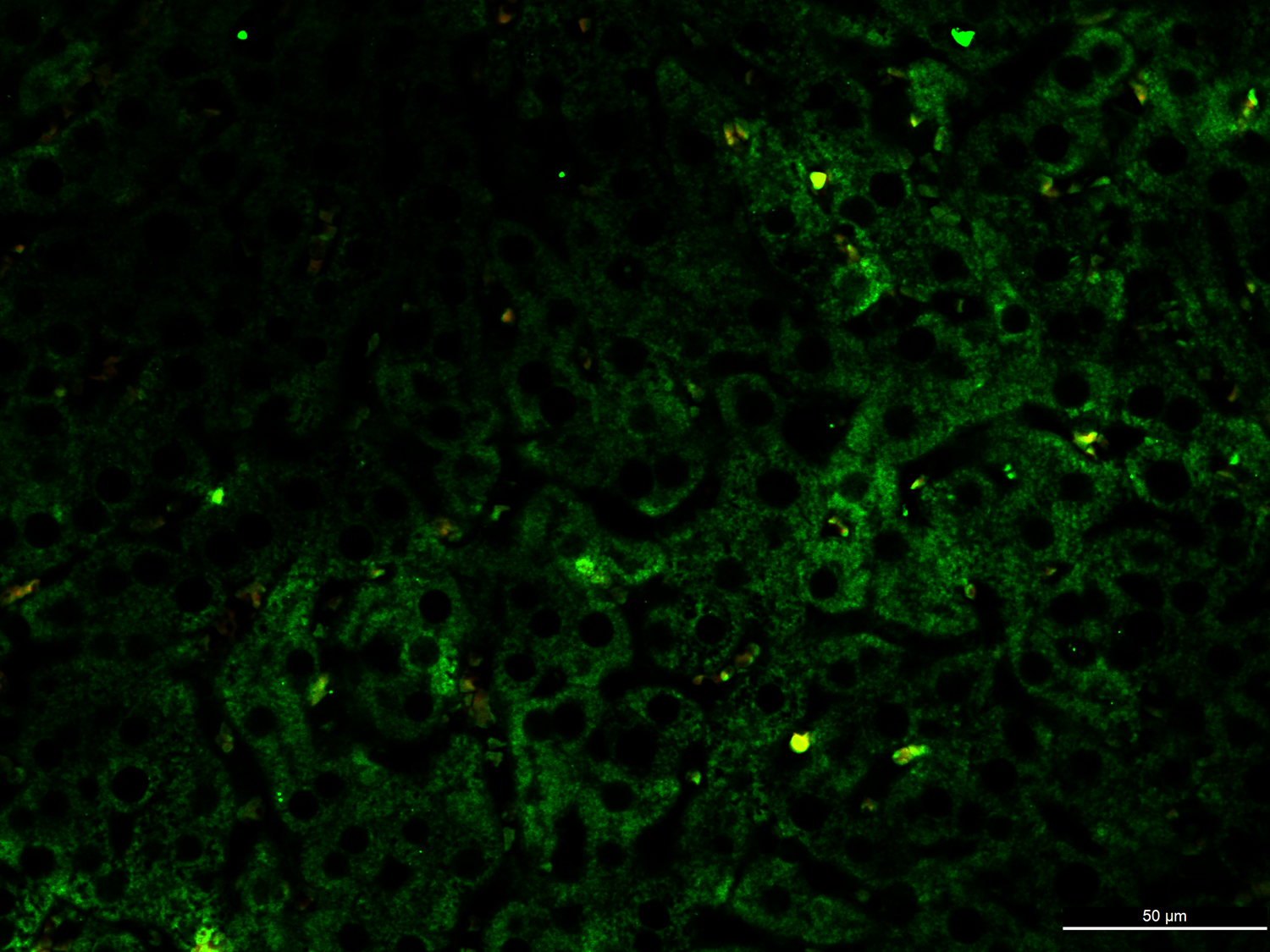

Supplement: Supplementary file 12 [file DataSheet2.ZIP › ST+IGF+LPS/SLG (2).tif]

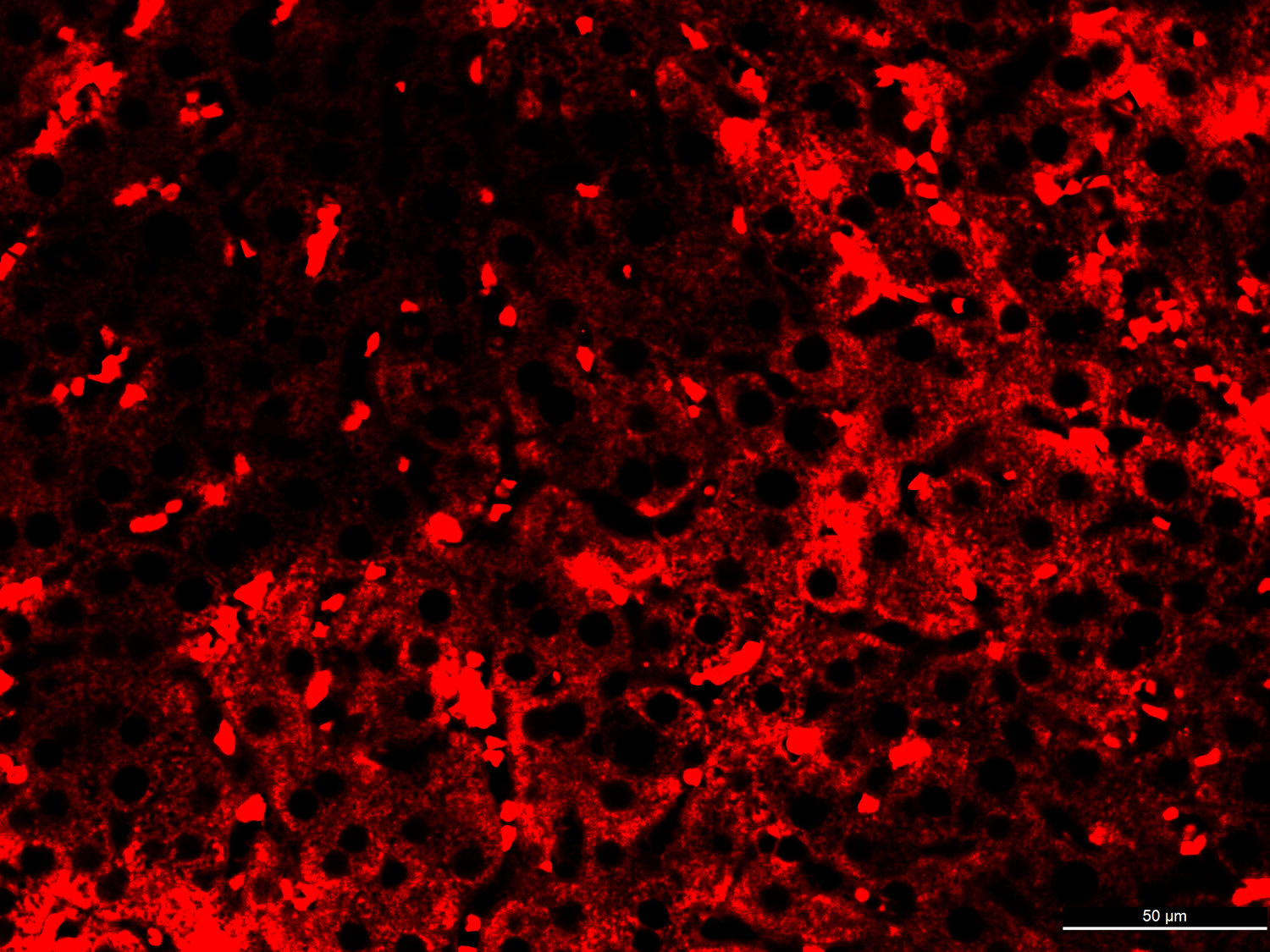

Supplement: Supplementary file 12 [file DataSheet2.ZIP › ST+IGF+LPS/SLG (3).tif]

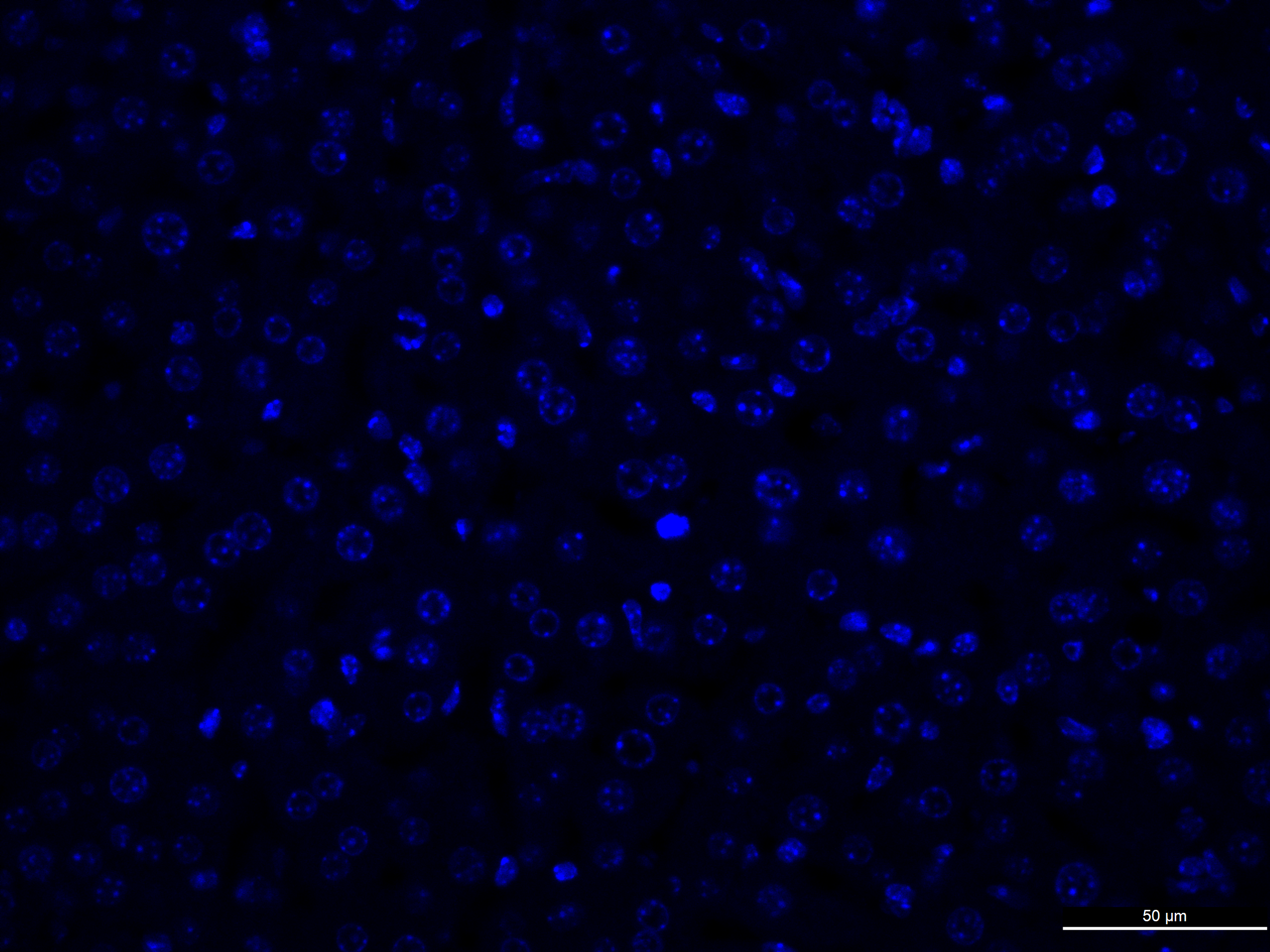

Supplement: Supplementary file 12 [file DataSheet2.ZIP › ST+IGF+LPS/SLG (4).tif]

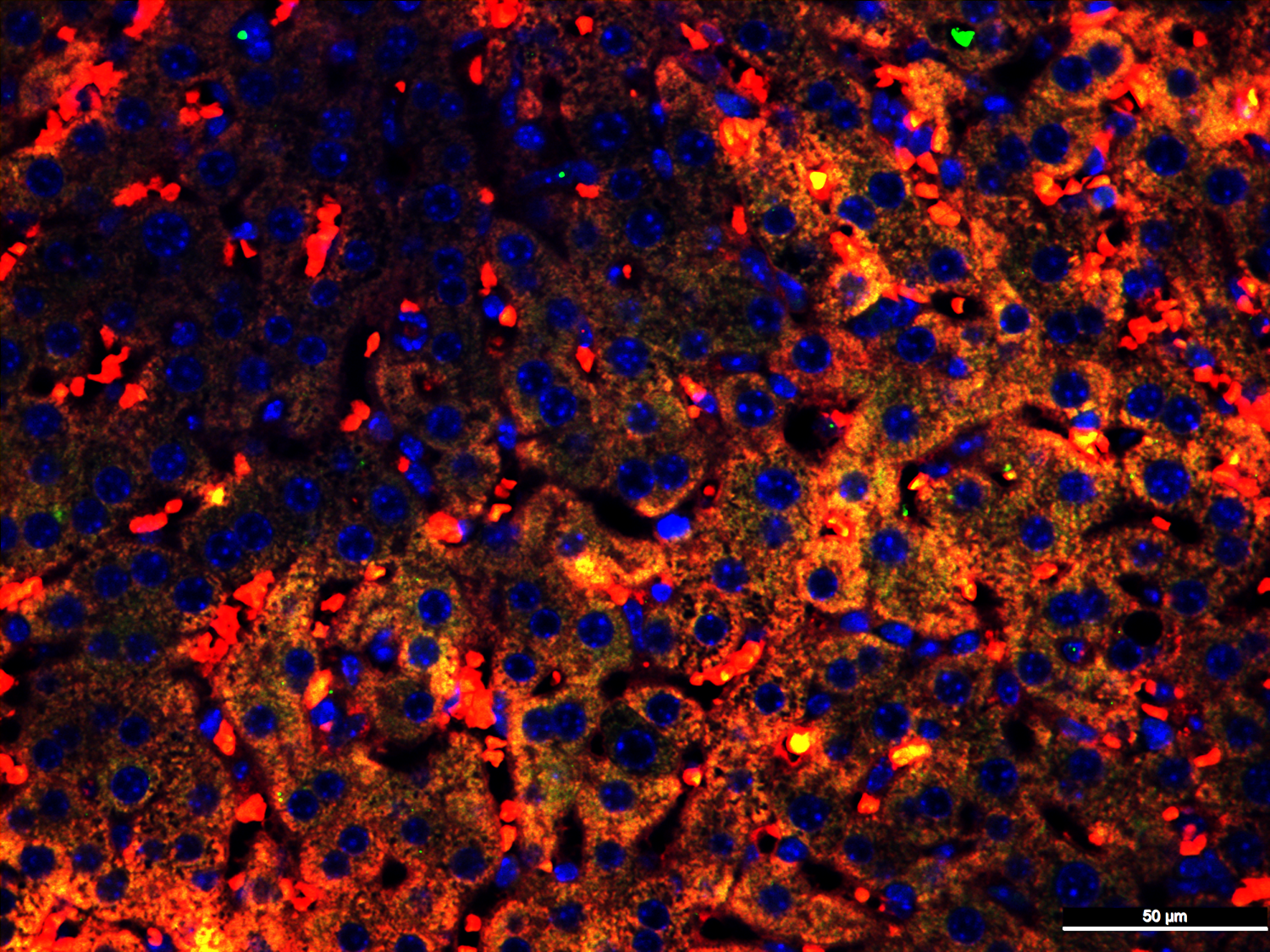

Supplement: Supplementary file 12 [file DataSheet2.ZIP › ST+IGF+LPS/SLG.tif]

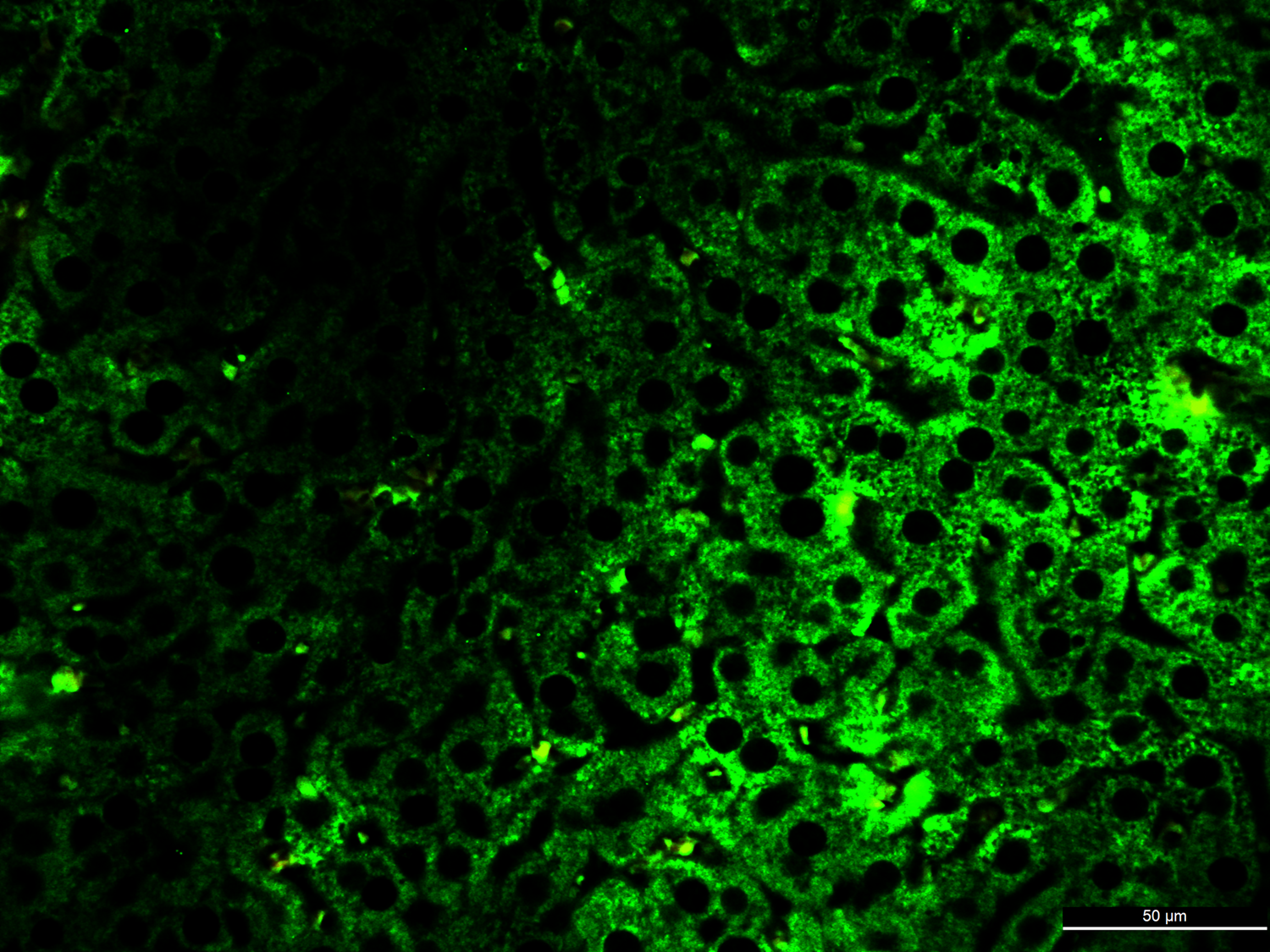

Supplement: Supplementary file 12 [file DataSheet2.ZIP › ST+LPS/ST LPS.tif]

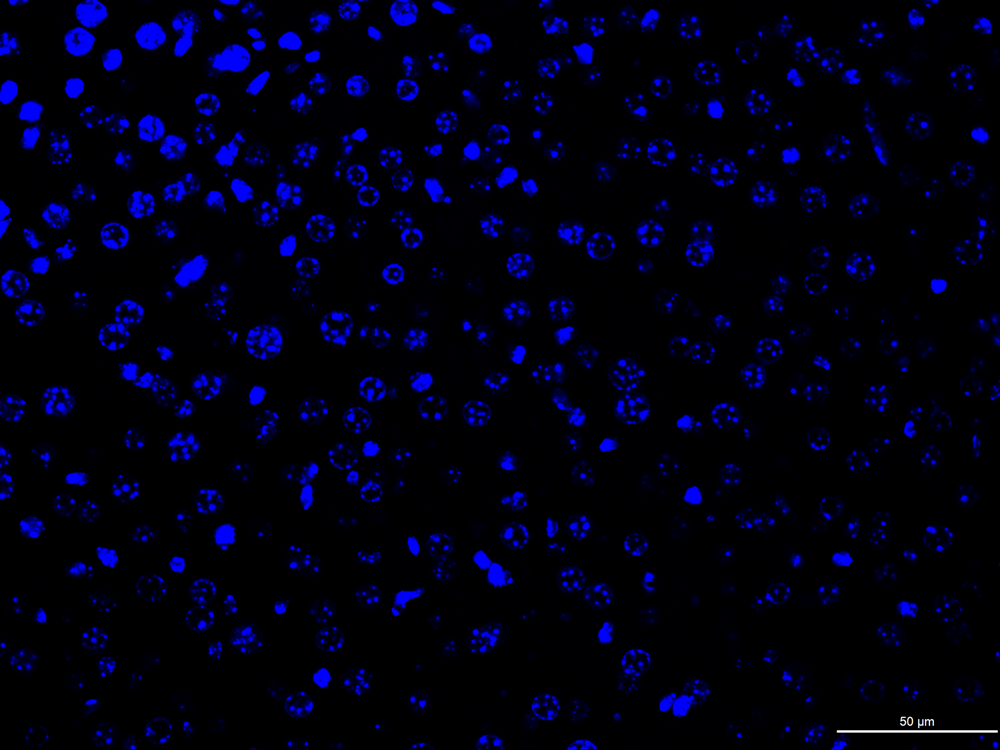

Supplement: Supplementary file 12 [file DataSheet2.ZIP › ST+LPS/ST+ LPS.tif]

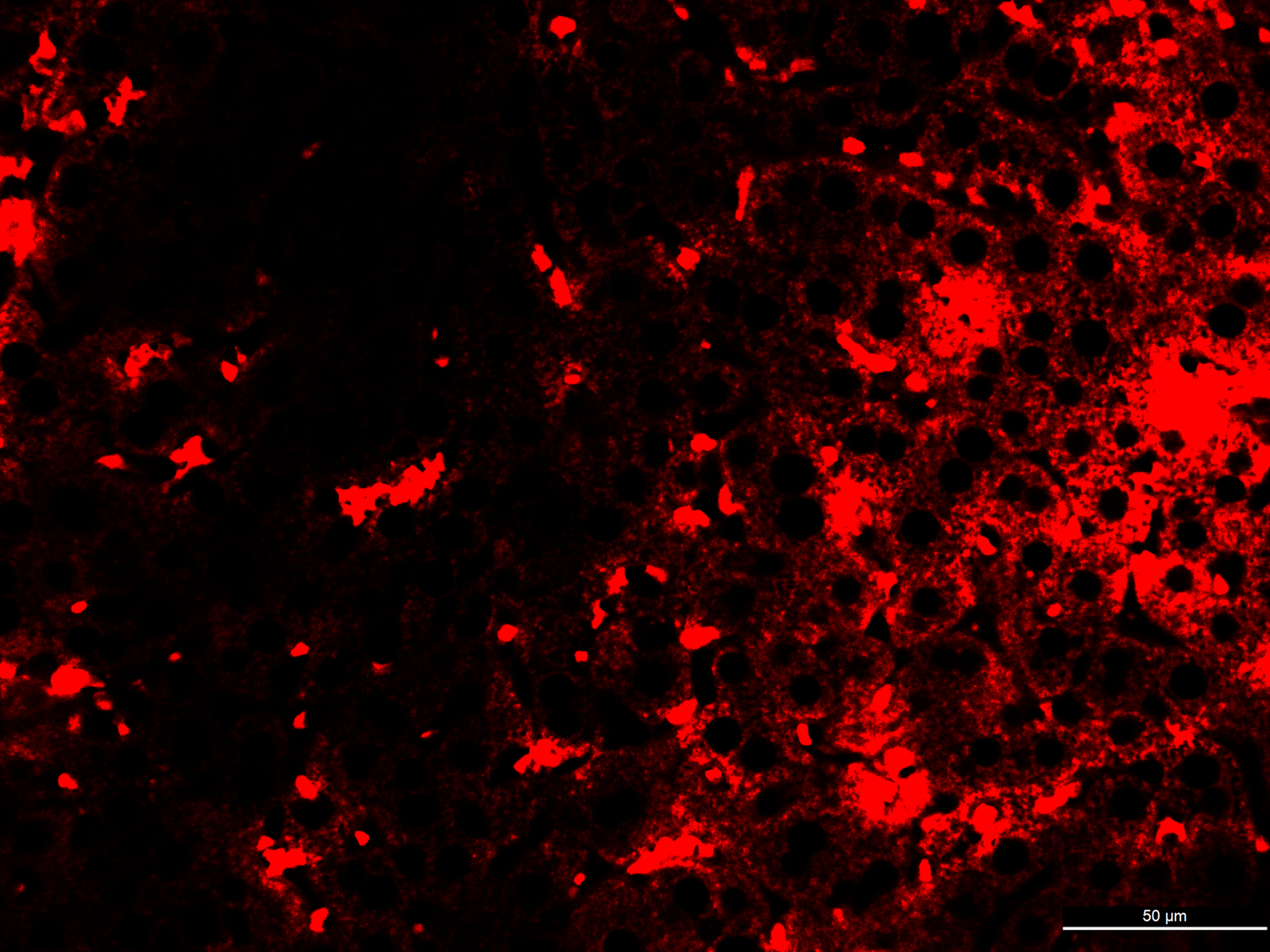

Supplement: Supplementary file 12 [file DataSheet2.ZIP › ST+LPS/ST+LPS 1.tif]

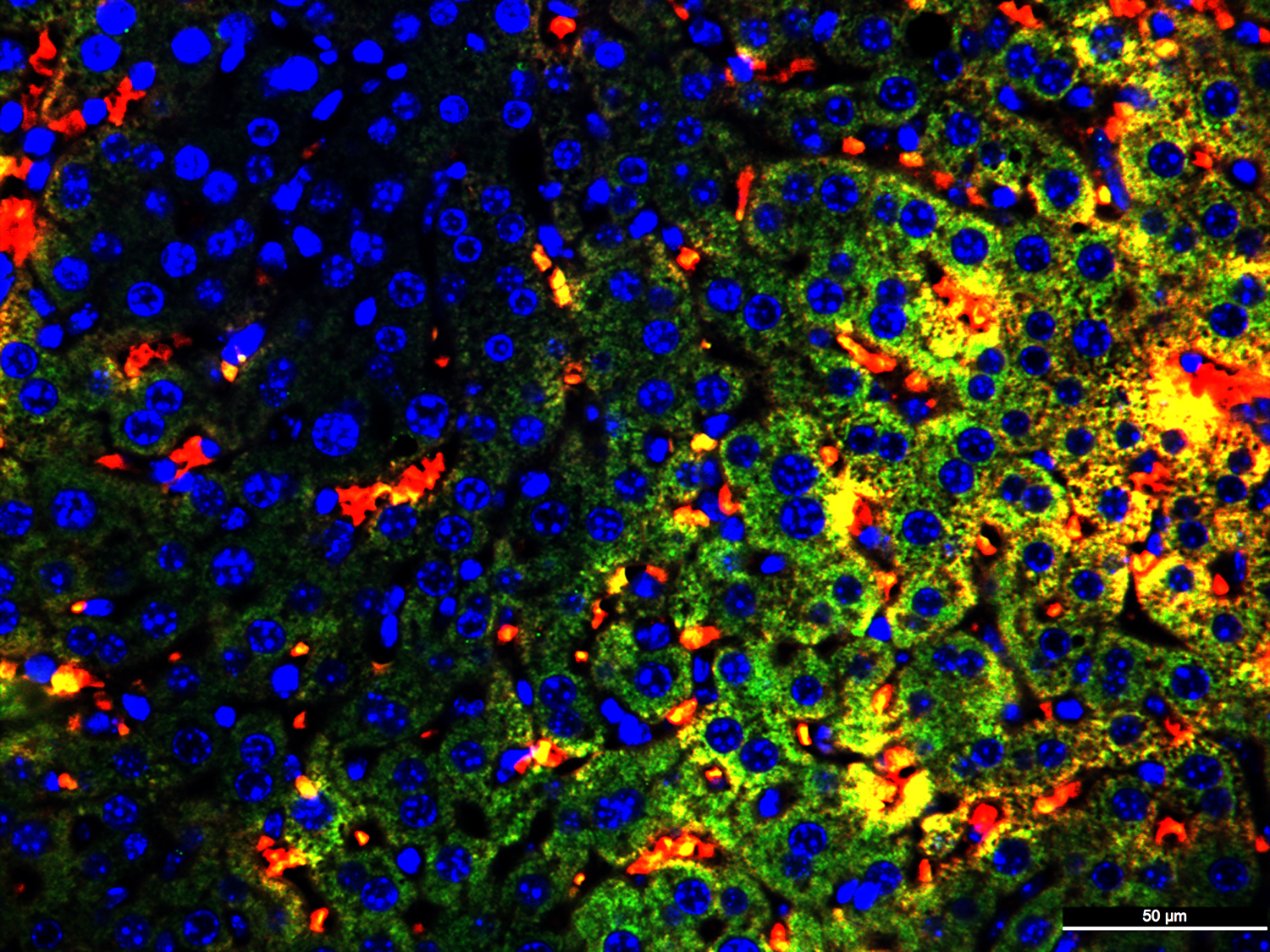

Supplement: Supplementary file 12 [file DataSheet2.ZIP › ST+LPS/ST+LPS.tif]

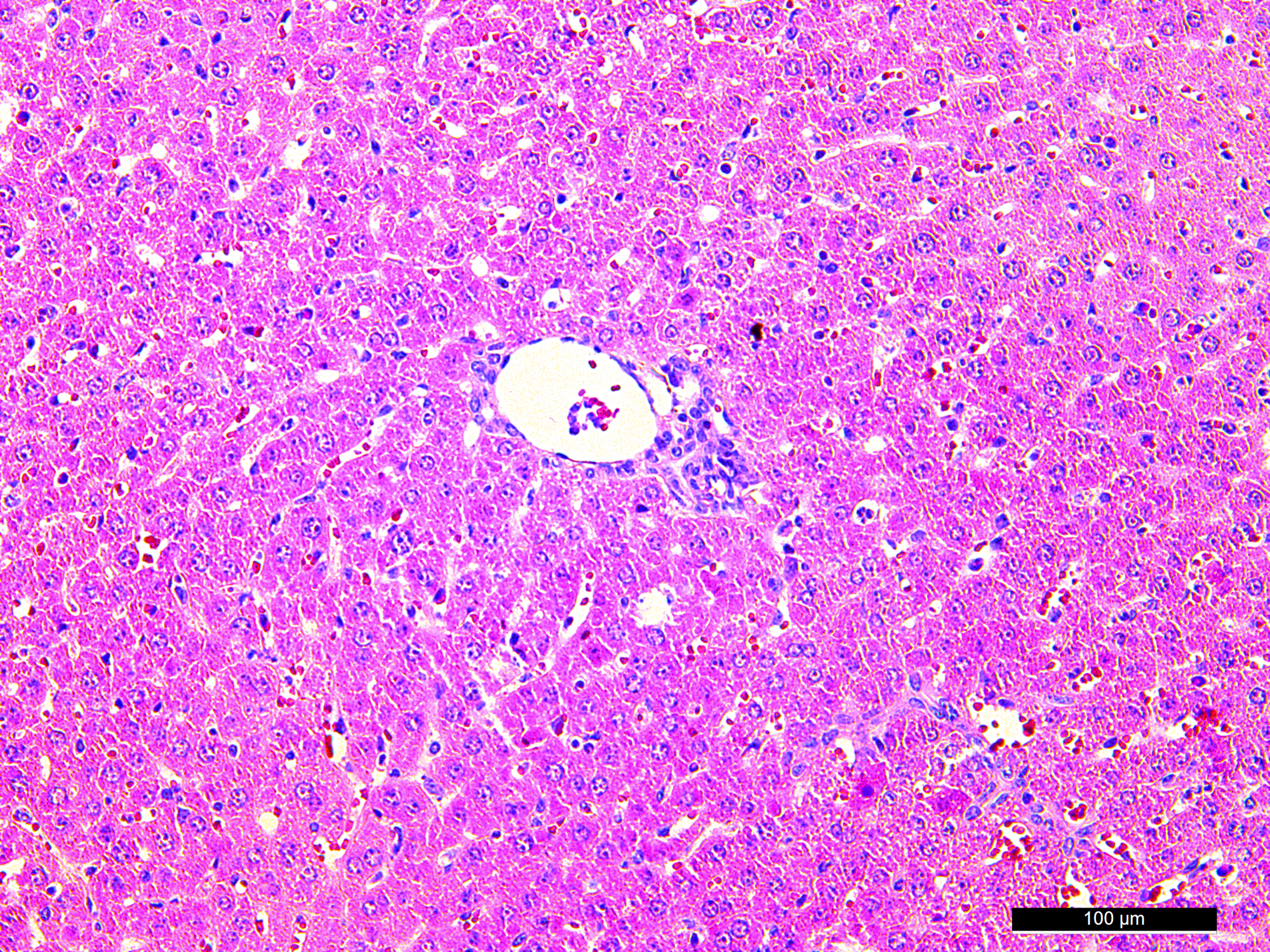

Supplement: Supplementary file 13 [file DataSheet5.ZIP › LPS.tif]

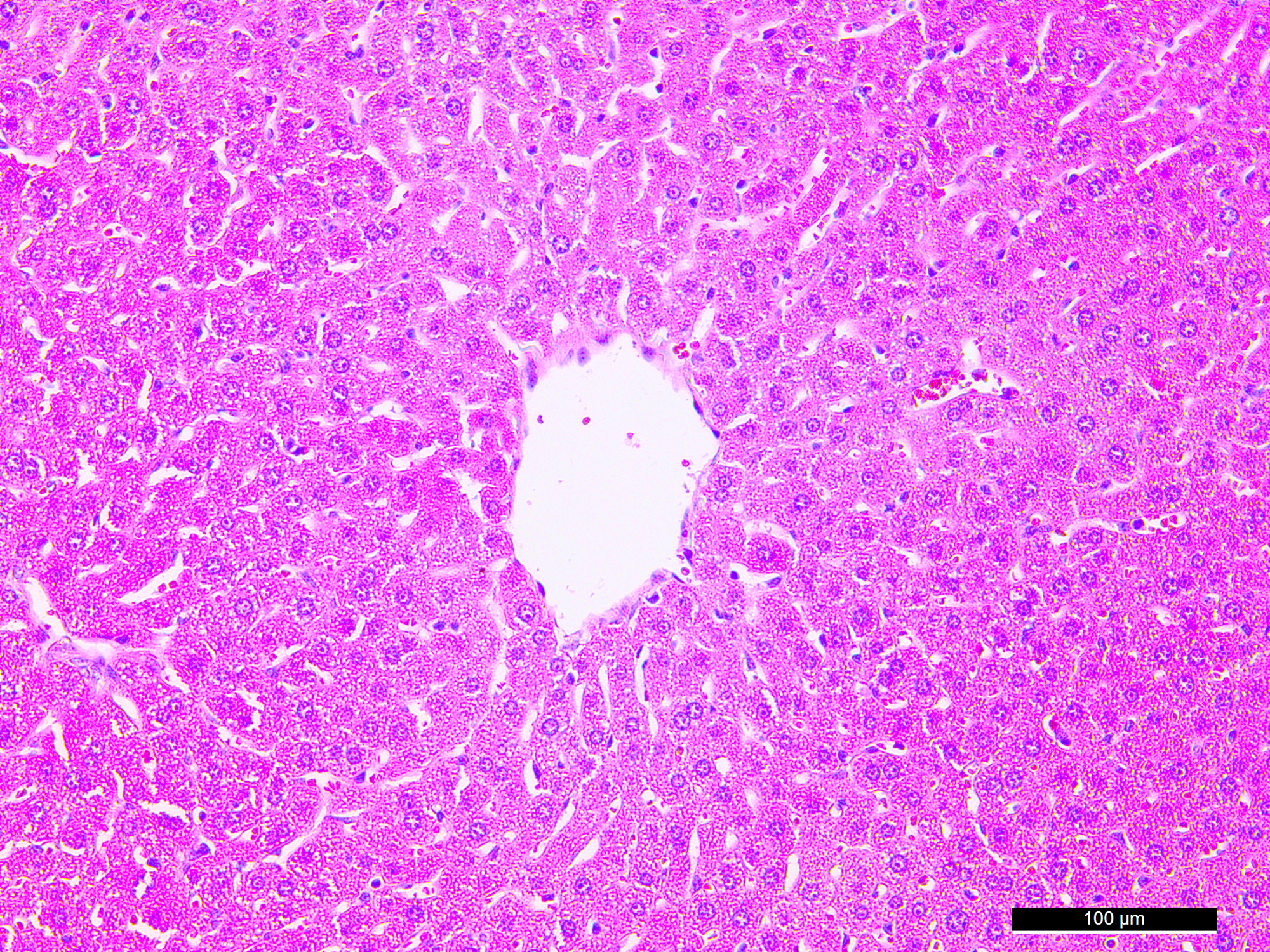

Supplement: Supplementary file 13 [file DataSheet5.ZIP › NC.tif]

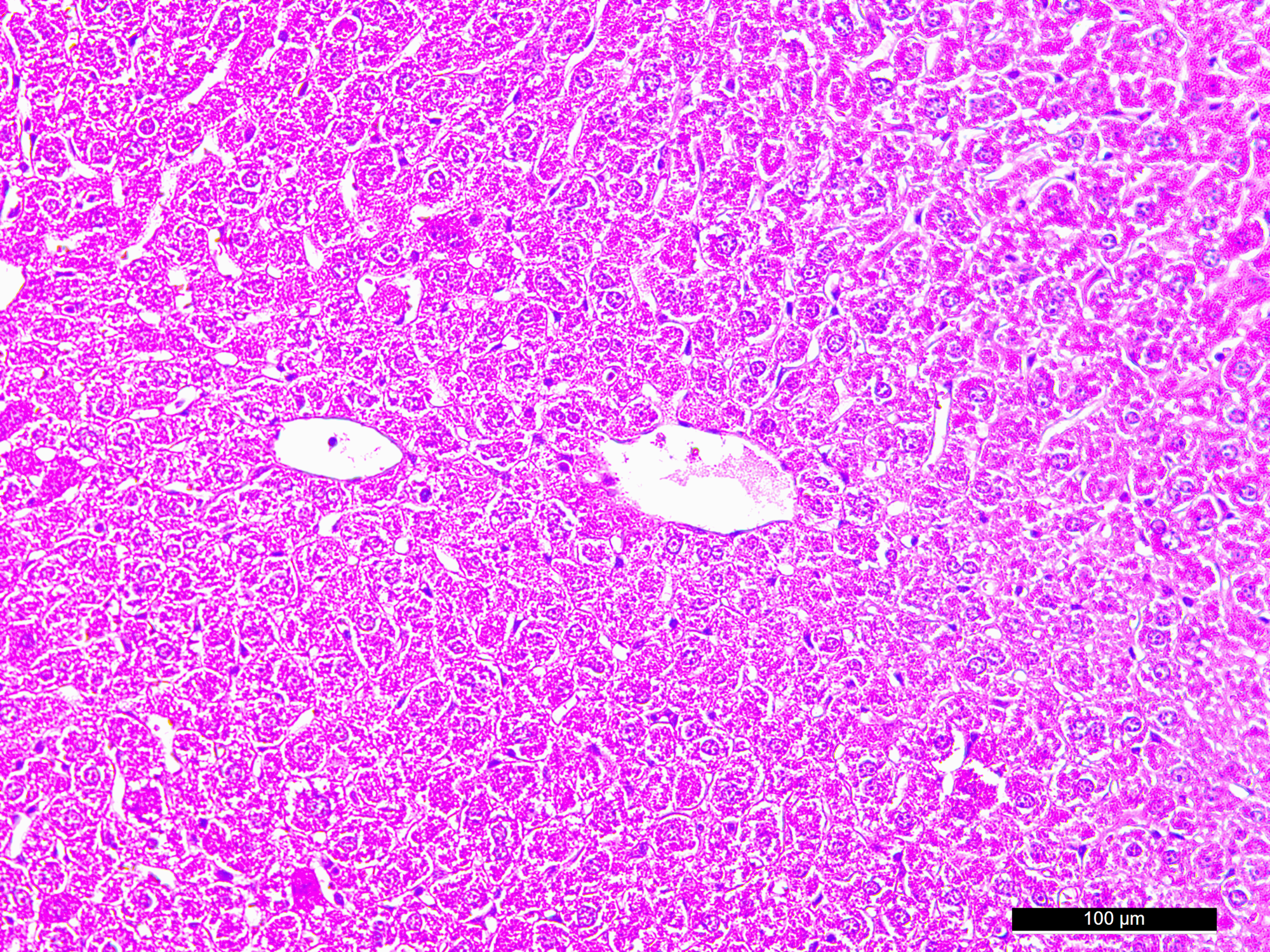

Supplement: Supplementary file 13 [file DataSheet5.ZIP › ST+LPS.tif]

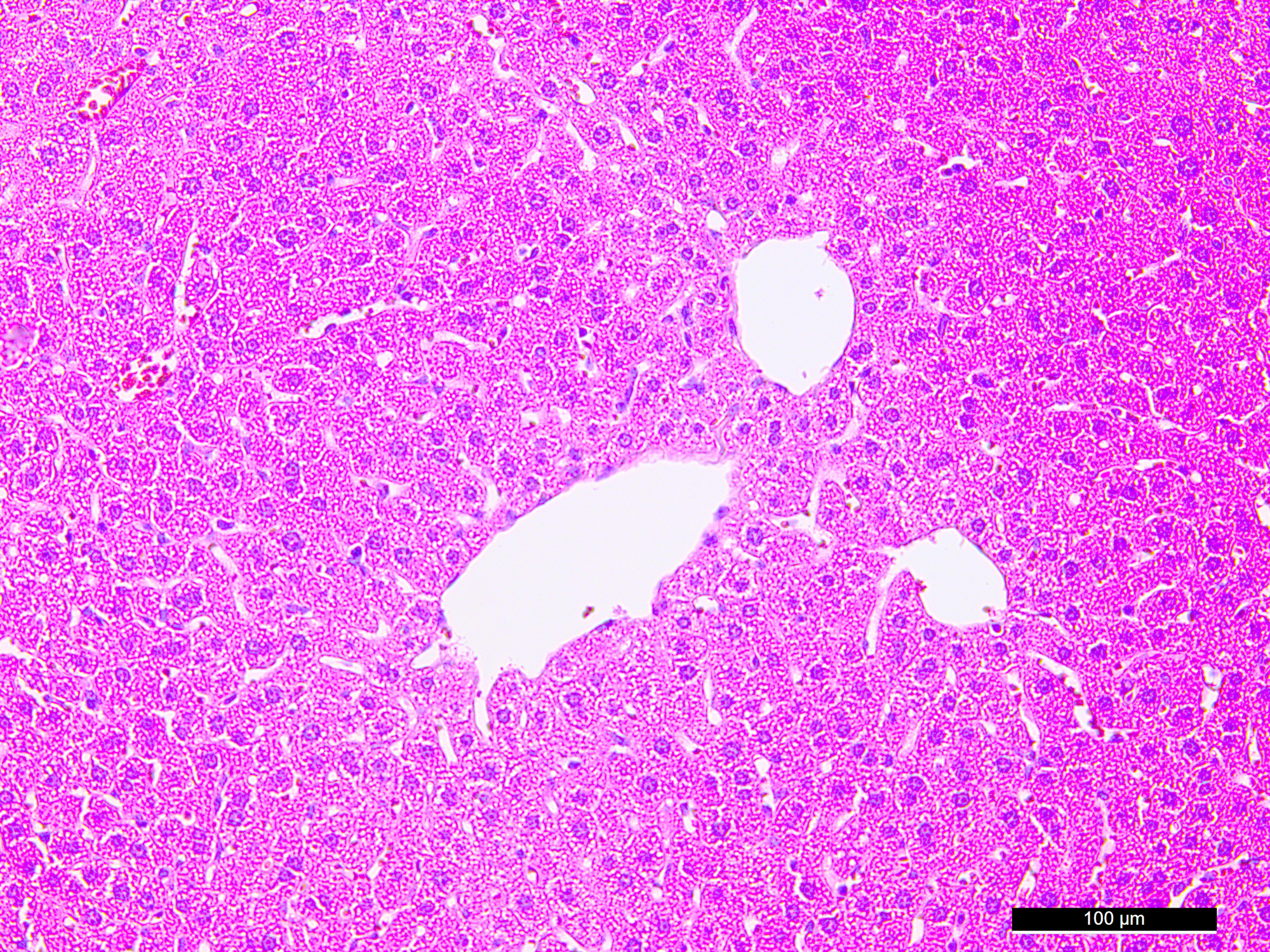

Supplement: Supplementary file 13 [file DataSheet5.ZIP › ST.tif]

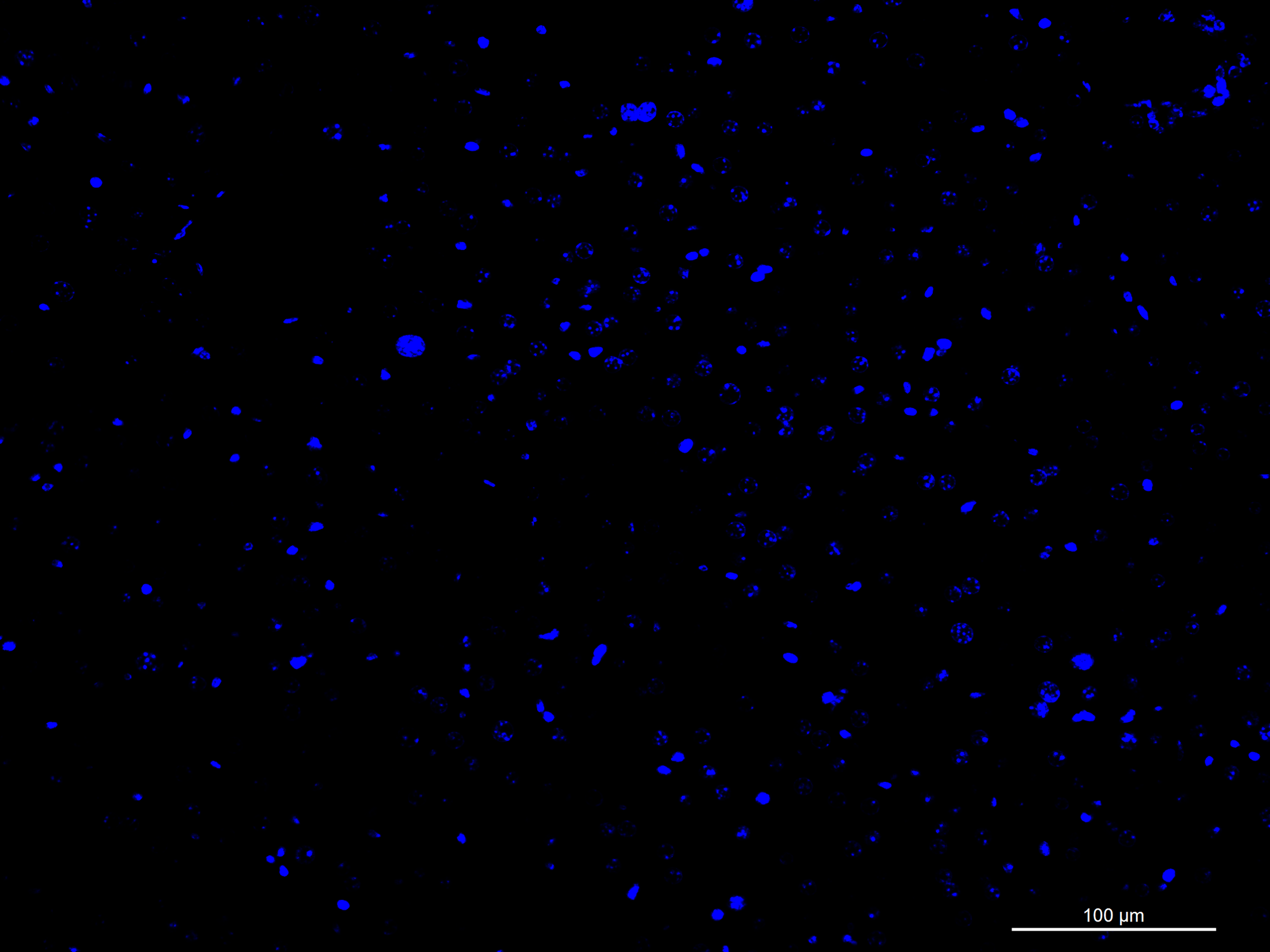

Supplement: Supplementary file 14 [file DataSheet7.ZIP › neutrophils con/NC (2).tif]

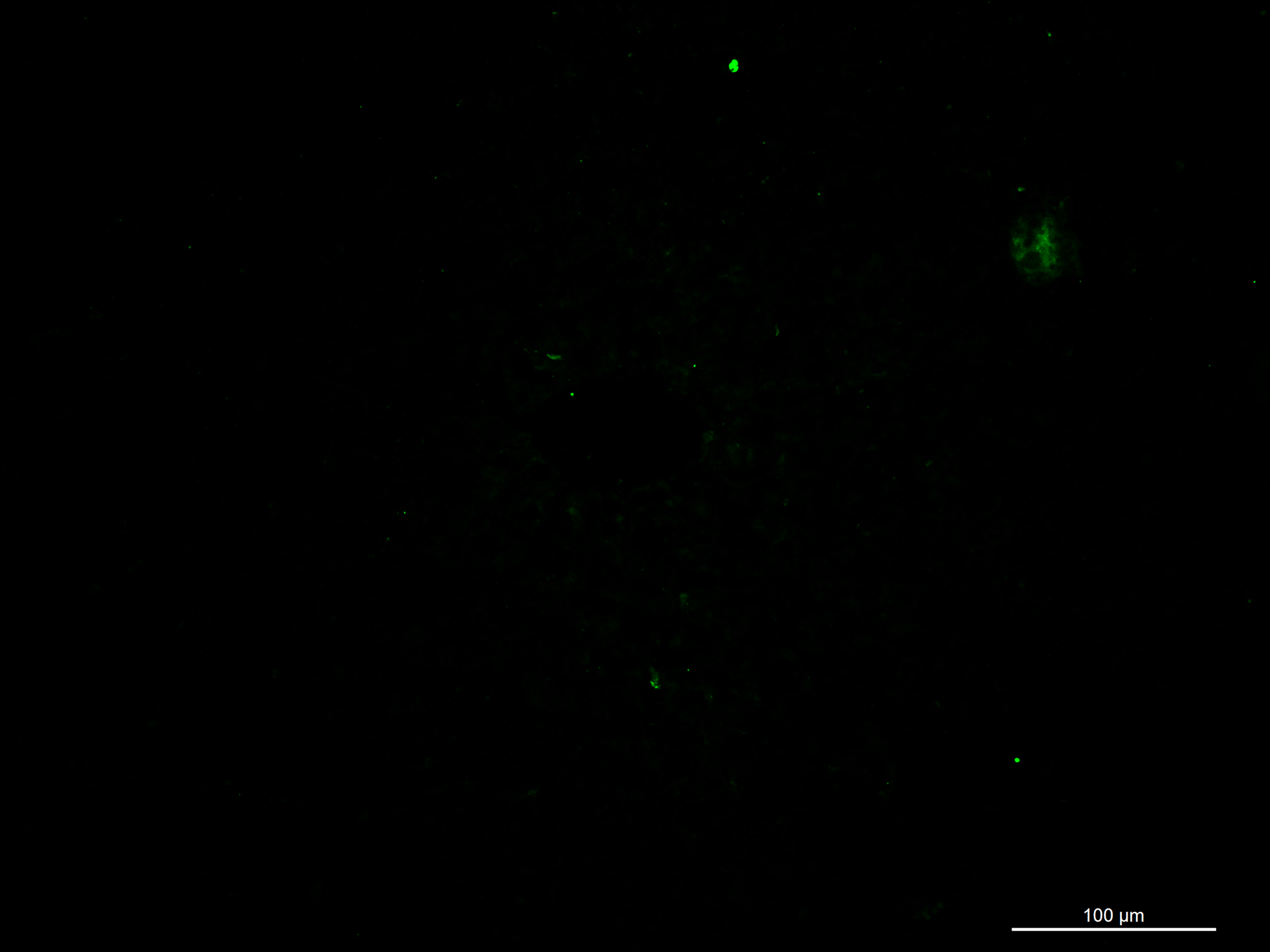

Supplement: Supplementary file 14 [file DataSheet7.ZIP › neutrophils con/NC (3).tif]

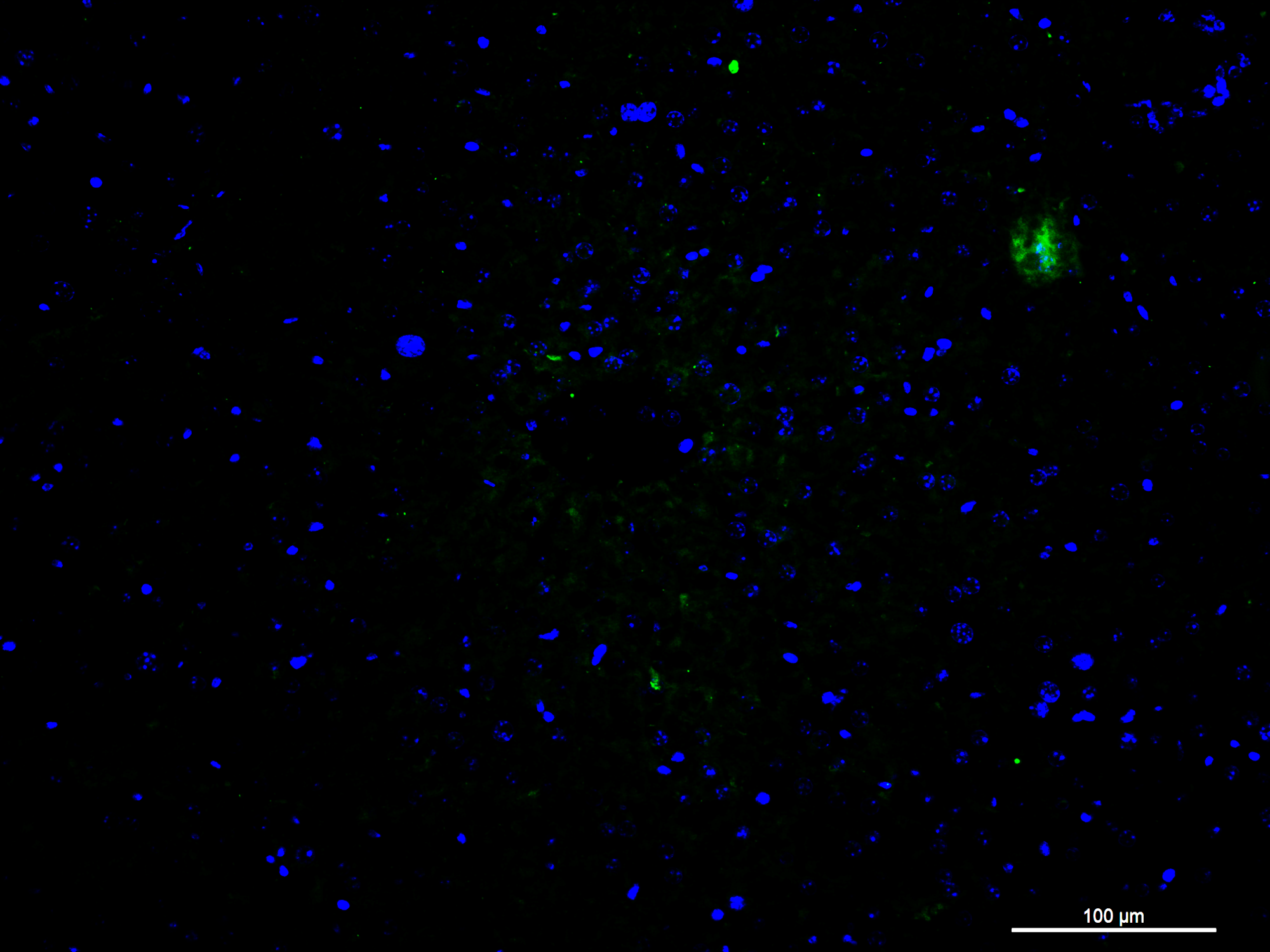

Supplement: Supplementary file 14 [file DataSheet7.ZIP › neutrophils con/NC.tif]

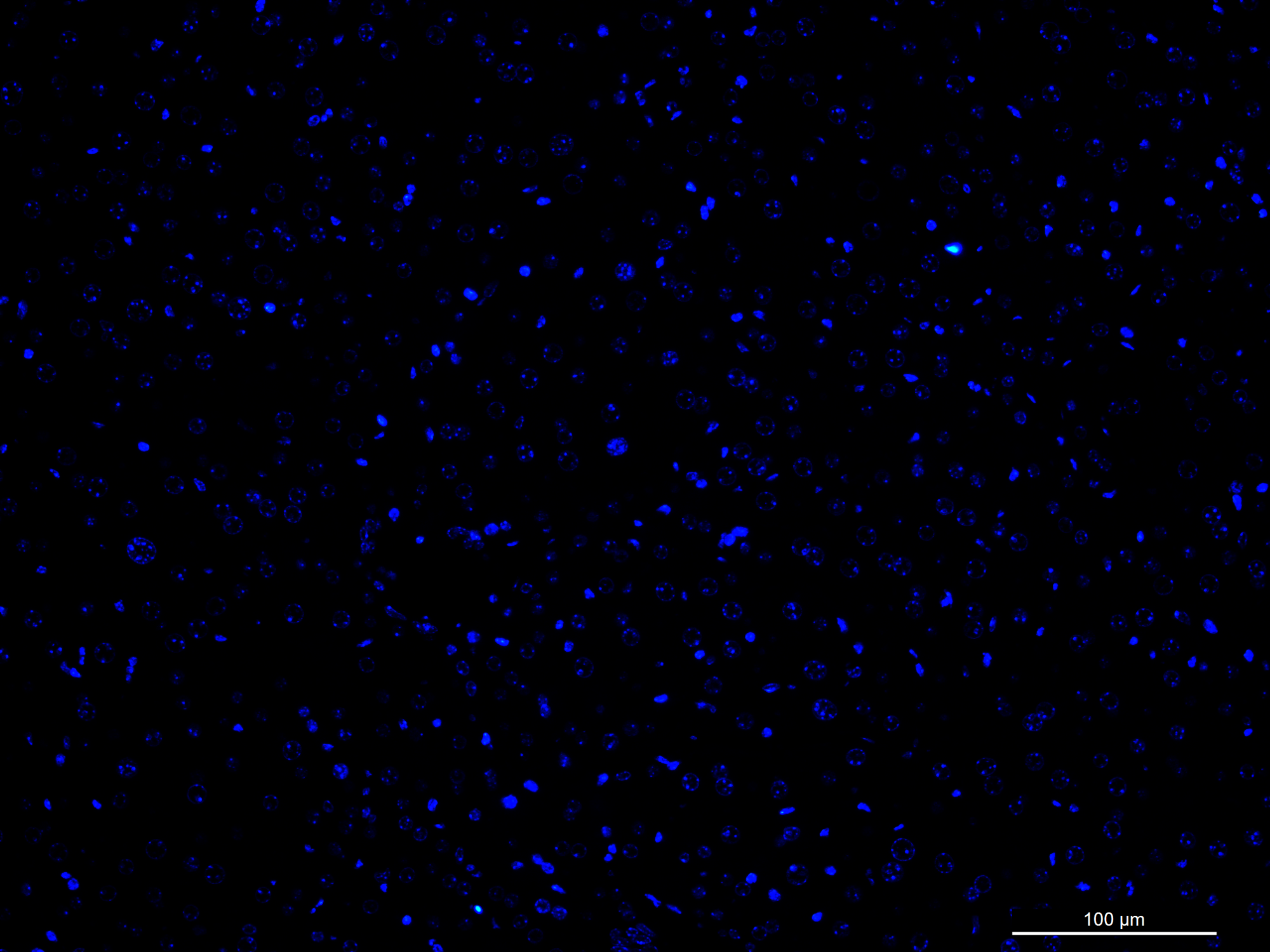

Supplement: Supplementary file 14 [file DataSheet7.ZIP › neutrophils LPS/LPS (2).tif]

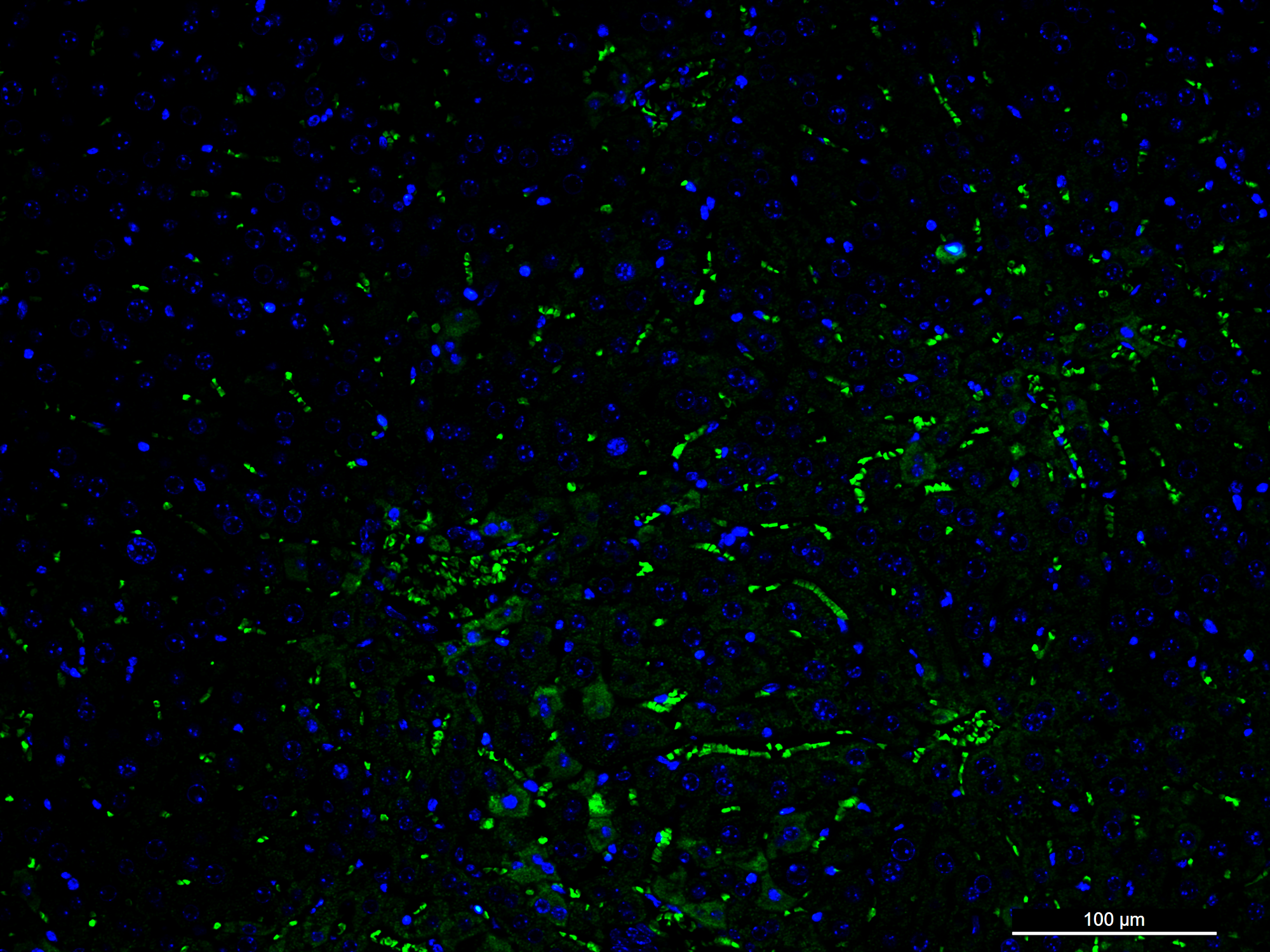

Supplement: Supplementary file 14 [file DataSheet7.ZIP › neutrophils LPS/LPS (3).tif]

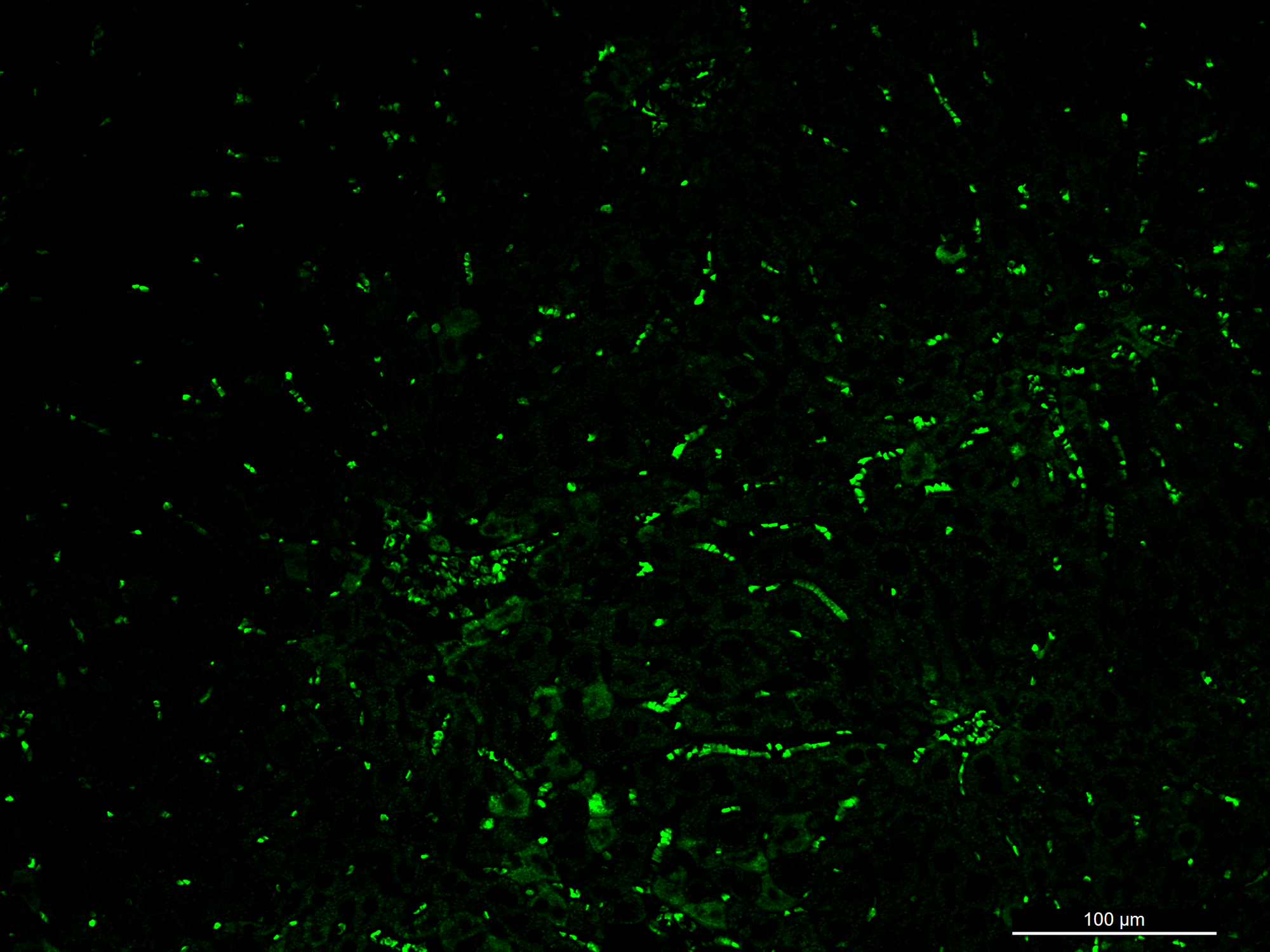

Supplement: Supplementary file 14 [file DataSheet7.ZIP › neutrophils LPS/LPS.tif]

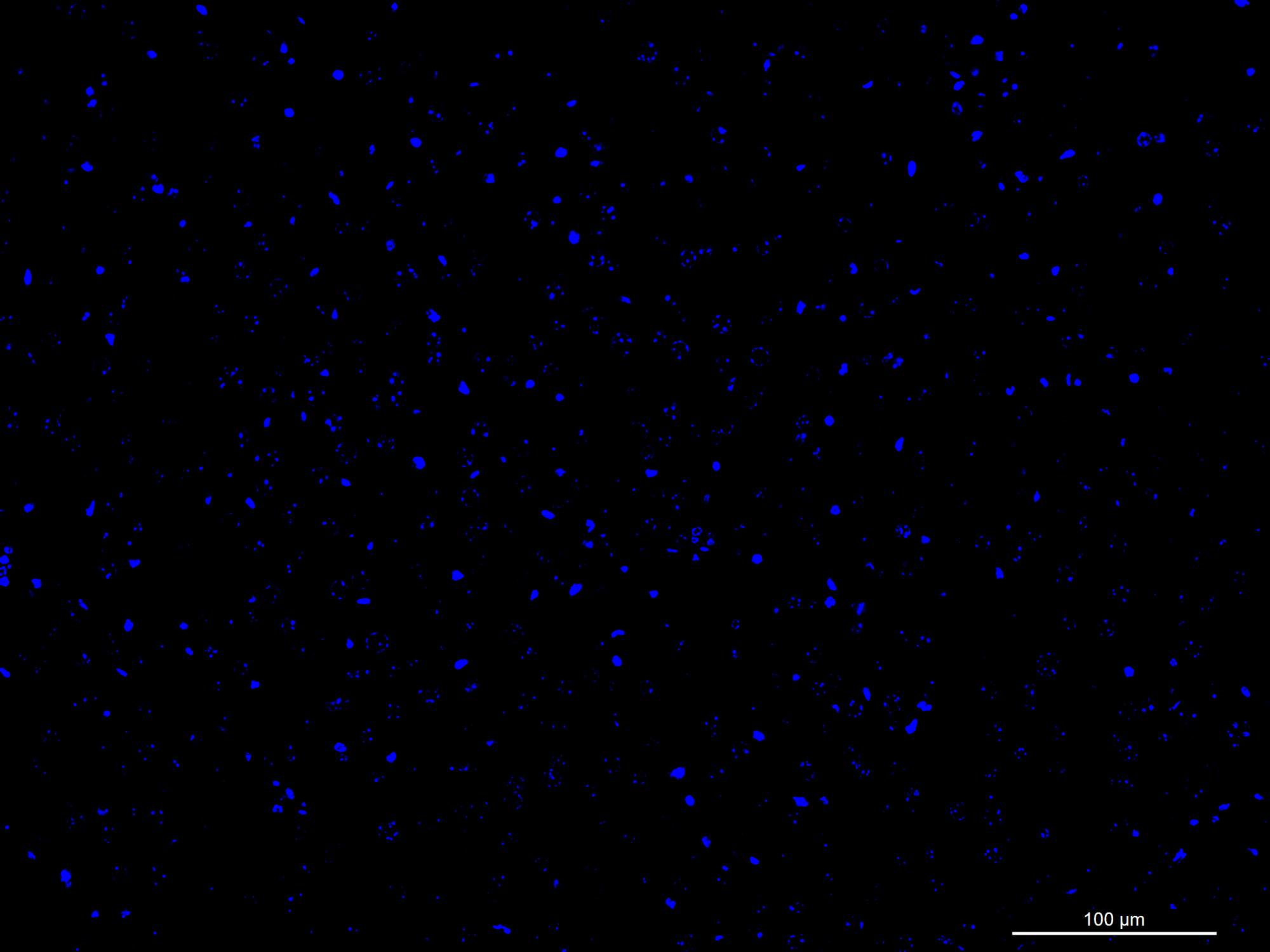

Supplement: Supplementary file 14 [file DataSheet7.ZIP › neutrophils ST+LPS/ST+LPS (2).tif]

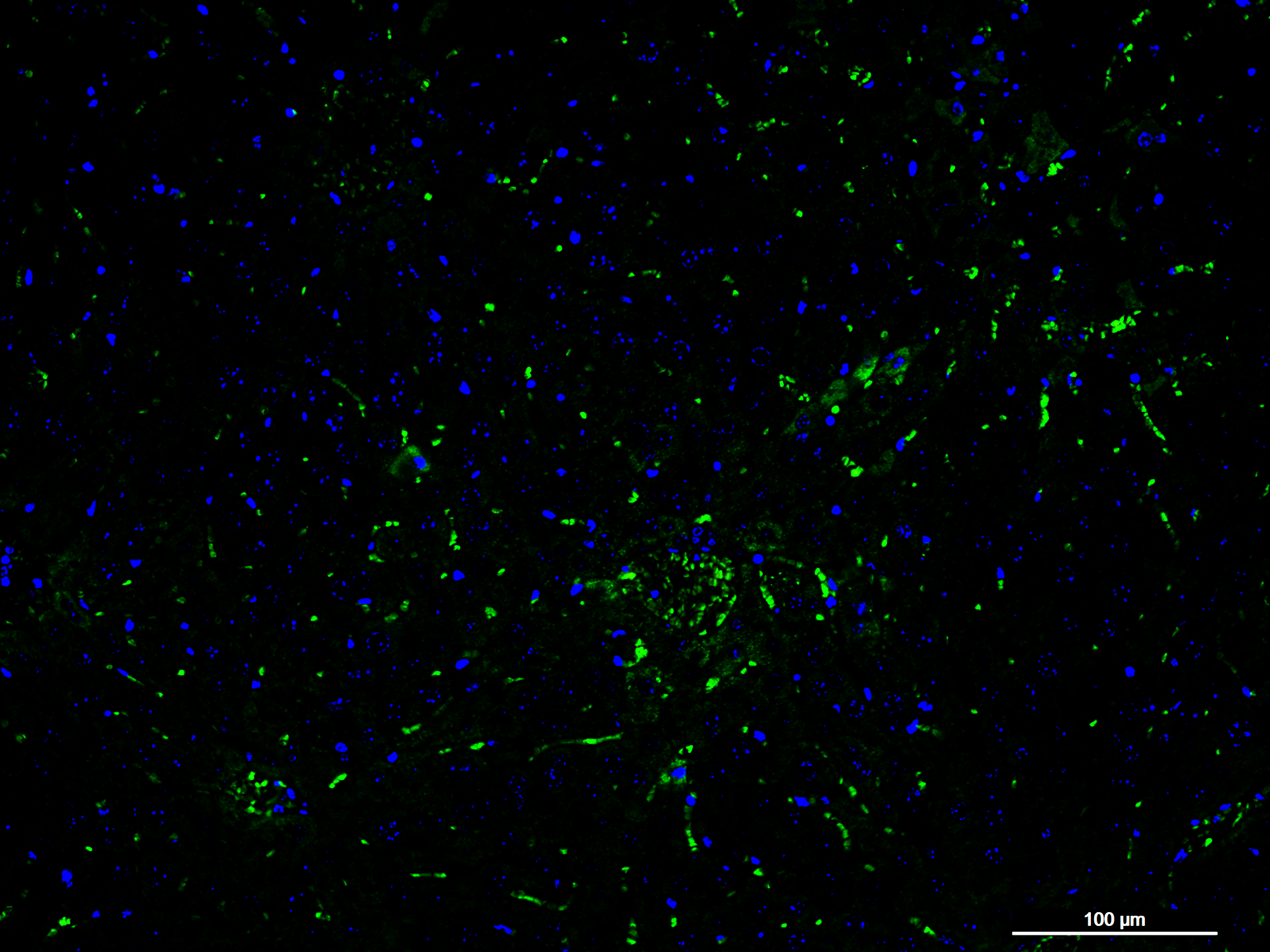

Supplement: Supplementary file 14 [file DataSheet7.ZIP › neutrophils ST+LPS/ST+LPS (3).tif]

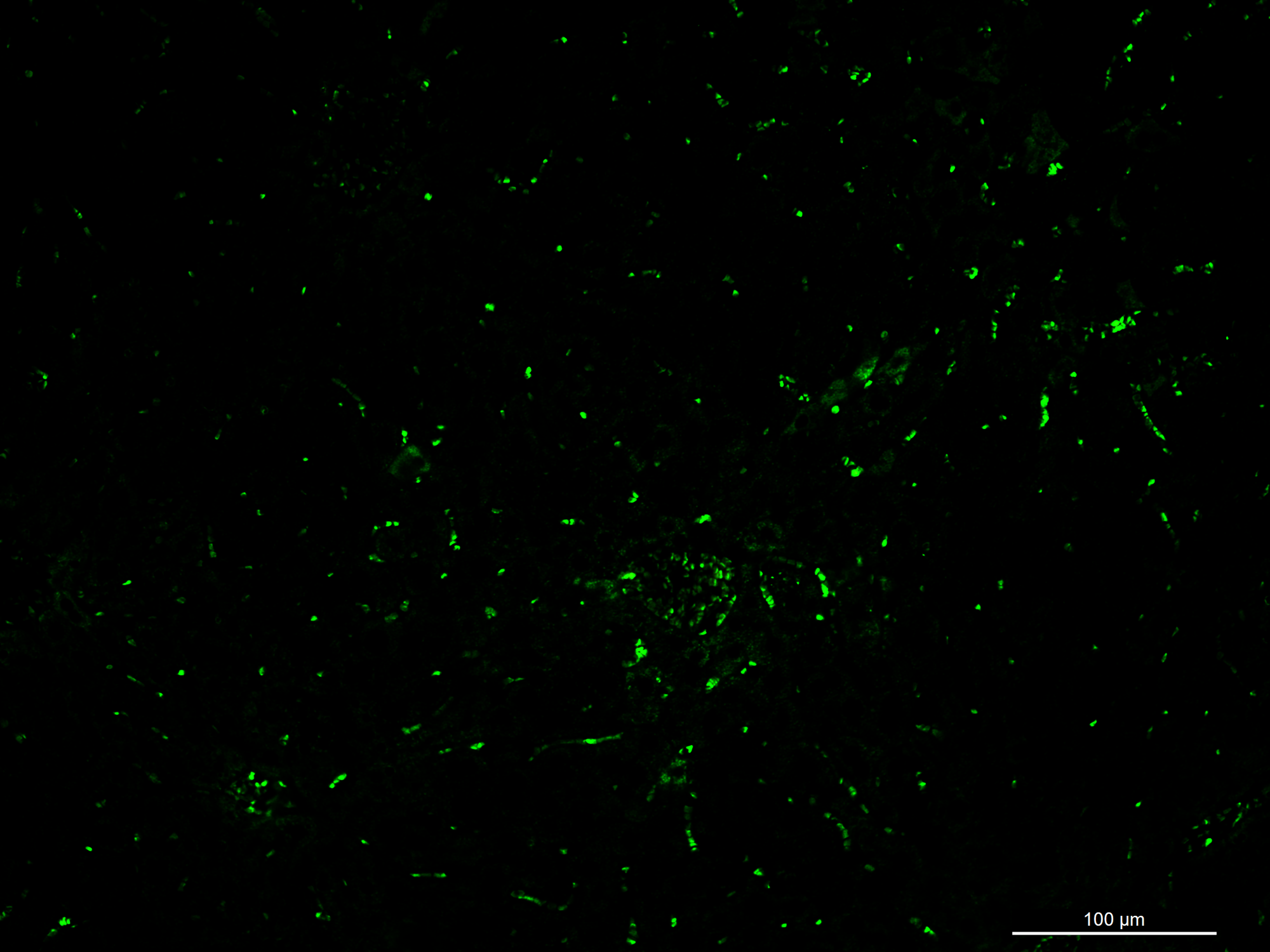

Supplement: Supplementary file 14 [file DataSheet7.ZIP › neutrophils ST+LPS/ST+LPS.tif]

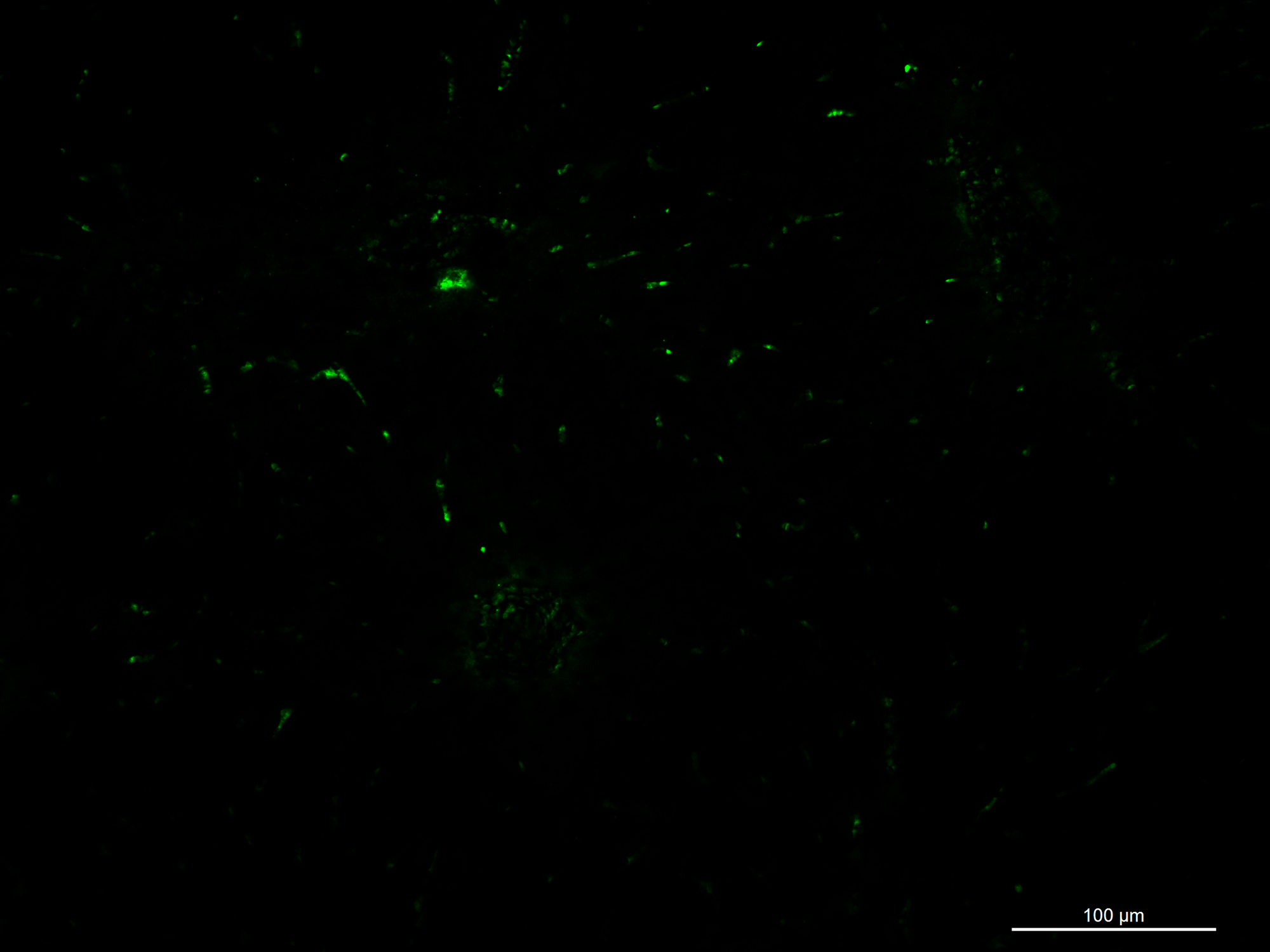

Supplement: Supplementary file 14 [file DataSheet7.ZIP › neutrophils ST/ST (2).tif]

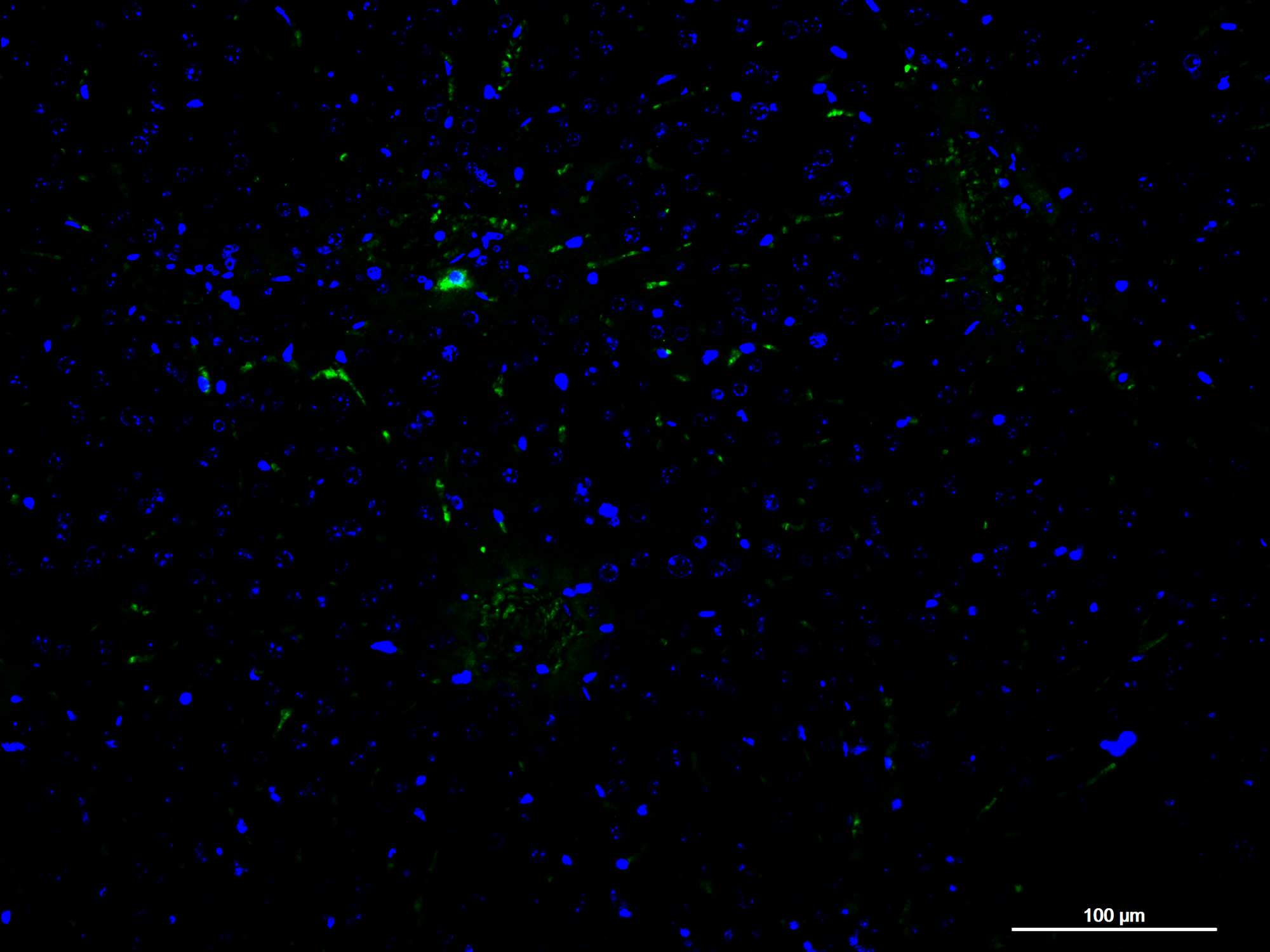

Supplement: Supplementary file 14 [file DataSheet7.ZIP › neutrophils ST/ST (3).tif]

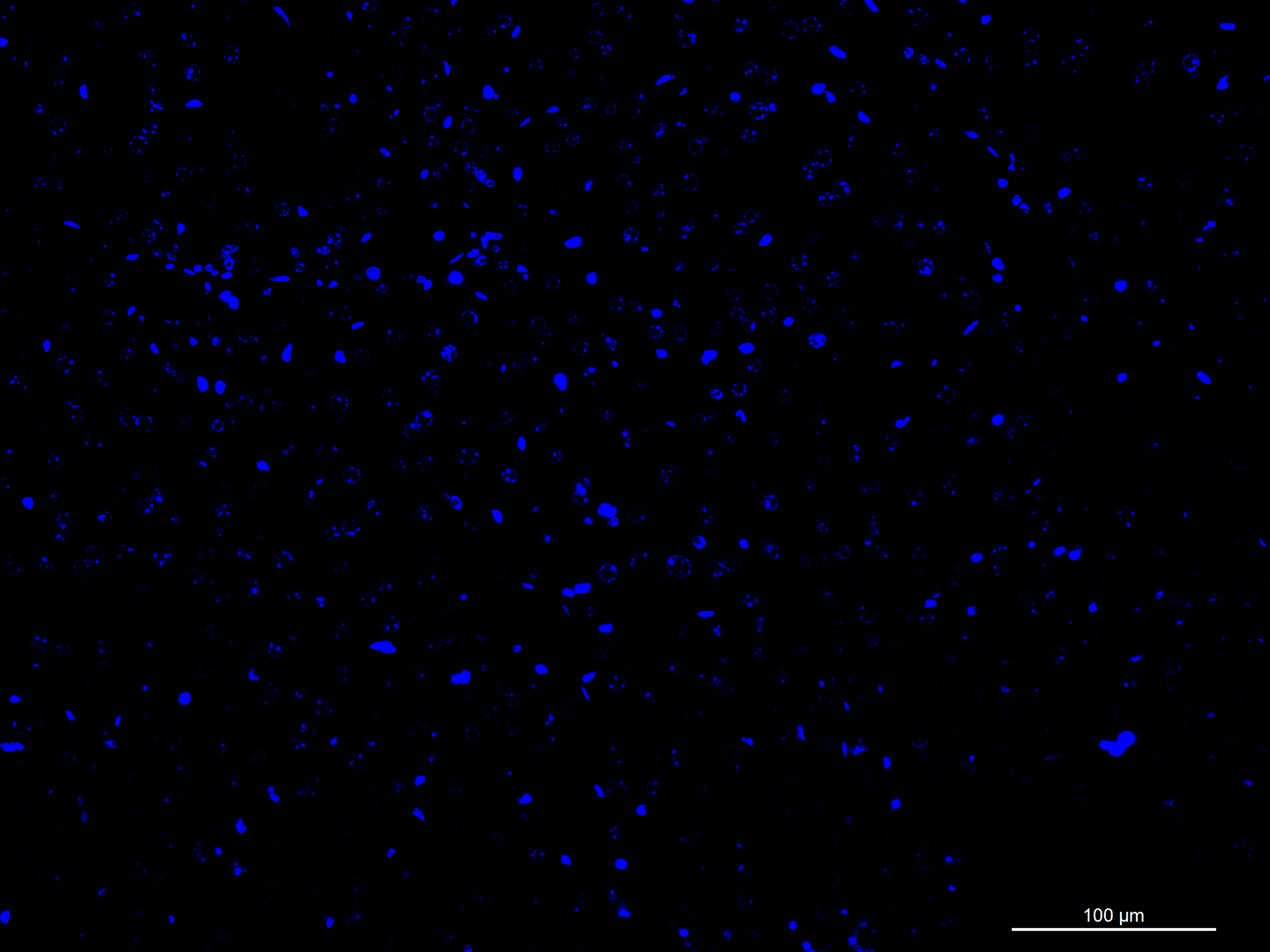

Supplement: Supplementary file 14 [file DataSheet7.ZIP › neutrophils ST/ST.tif]
